# Data set of dissolved major and trace elements from the lacustrine systems of Clearwater Mesa, Antarctica

**DOI:** 10.1016/j.dib.2020.105438

**Published:** 2020-03-19

**Authors:** Karina L. Lecomte, Cecilia V. Echegoyen, Paula A. Vignoni, Kateřina Kopalová, Tyler J. Kohler, Silvia H. Coria, Juan M. Lirio

**Affiliations:** a Centro de Investigaciones en Ciencias de la Tierra (CICTERRA), CONICET/Universidad Nacional de Córdoba, Av. Vélez Sarsfield, 1611, X5016CGA Córdoba, Argentina; b Facultad de Ciencias Exactas Físicas y Naturales, Universidad Nacional de Córdoba, Av. Vélez Sarsfield, 1611, X5016CGA Córdoba, Argentina; c Institute of Geosciences, Potsdam University, Karl-Liebknecht-Straße. 24-25, 14476, Potsdam-Golm, Germany; d Climate Dynamics and Landscape Evolution, German Research Centre for Geoscience GFZ, Telegrafenberg, 14473, Potsdam, Germany; e Department of Ecology, Faculty of Science, Charles University, Viničná 7, 128 44, Praha 2, Czech Republic; f Instituto Antártico Argentino, 25 de Mayo 1143, San Martín, Prov. Buenos Aires, Argentina

**Keywords:** Water chemistry, Pristine environments, Dissolved trace elements, High latitude lakes

## Abstract

This article presents analytical observations on physicochemical parameters and major and trace element concentrations of water, ice, and sediment samples from the lake systems of Clearwater Mesa (CWM), northeast Antarctic Peninsula. Geochemical analyses include inductively coupled plasma mass spectrometry (ICP-MS) for cations and trace elements and ion chromatography for anions. Some figures are included (i.e. Piper and Gibbs diagrams) which indicate water classification type and rock-water interactions in CWM, respectively. It also contains PHREEQC software output, listing the chemical speciation for dissolved elements, Saturation Indexes (SI), and modelling outputs. Each lake SI are also illustrated in a figure. Finally, total organic and inorganic carbon (TOC and TIC, respectively) were determined for bottom lake sediments and marginal salt samples. This information will be useful for future research assessing the impacts of anthropogenic pollution and the effects of climate change, providing insights into naturally occurring geochemical processes in a pristine environment, and evaluating geochemical behaviour of dissolved elements in high-latitude hydrological systems. These data correspond to the research article “Dissolved major and trace geochemical dynamics in Antarctic Lacustrine Systems” [1].

Specifications table

**Table:** 

Subject	Earth and Planetary Sciences
Specific subject area	Earth-Surface Geochemical Processes
Type of data	Table Figure Other (Software Output)
How data were acquired	Major cations and trace elements were determined by inductively coupled plasma mass spectrometry (PerkinElmer Sciex ELAN 9000 ICP/MS, PerkinElmer Nexion, Thermo icapQ or Agilent 7700, ActLabs, Canada). Chloride and sulphate were determined by chemically suppressed ion chromatography with conductivity detection (Thermo, model Constametric 3500, with Dionex suppressor and IonPac AS22 Dionex column -4 × 250 mm- for anions, CICTERRA-UNC, Argentina). *In situ* measurements include: alkalinity (volumetric methods), pH and Eh (Hach digital detector), electrical conductivity, temperature, and total dissolved solids (TDS, Hach conductivimeter). TIC and TOC values were determined in sediment samples following Loss on ignition method (LOI, Heiri et al. 2001). Chemical information was processed with PHREEQC Interactive software (U.S. Geological Survey) [2]. Sediment texture was determined with a particle analyser (Horiba LA-950, LabGEO, CICTERRA-UNC, Argentina).
Data format	Raw analyzed Other (Software Output)
Parameters for data collection	Samples were collected following standard protocols [3]. Parameters for data collection are: 1-Temperature, pH, redox potential, alkalinity, electrical conductivity, and TDS measured *in situ*. 2- Dissolved major and trace element concentrations, in filtered and acidified samples. 3-TIC and TOC in lyophilized sediments. 4- Grain size characterization in sediments with removed carbonates and organic matter.
Description of sample collection	72 samples were collected (2 ice, 35 lake water, 34 lake sediment, and one salt sample) from 35 different lakes and ponds during January 2015. Clean polythene bottles were used to collect and transport samples to each specific laboratory. Water samples were collected and stabilized (filtered in the field through a 0.22 µm pore size filter and acidified to pH<2) according to established protocols, and stored in a refrigerator (<4 °C) until analysis. Sediment samples were collected from the margins of lakes using a standard scoop sampler and stored in plastic bags.
Data source location	Clearwater Mesa (James Ross Island, northeast Antarctic Peninsula).
Data accessibility	Data are presented with this article.
Related research article	Karina L. Lecomte, Paula A. Vignoni, Cecilia V. Echegoyen, Pia Santolaya, Kateřina Kopalová, Tyler J. Kohler, Matěj Roman, Silvia H. Coria, Juan M. Lirio, Dissolved major and trace geochemical dynamics in Antarctic lacustrine systems, *Chemosphere,* (2020) 240, 124,938 DOI: https://doi.org/10.1016/j.chemosphere.2019.124938

## Value of the data

• Given the logistical constraints associated with collecting information from this unique and scarcely known high latitude environment, these observations present an opportunity for hydrological and geochemical research without the need for resampling.
• This information can be helpful for multidisciplinary researchers, such as environmentalists, biologists, physics, and geologists, and can be readily incorporated into meta-analyses.
• This information can be used to identify and analyse processes controlling high latitude lake chemistry through geochemical diagrams and models, and compare them with other environments.
• The present information contributes to a growing database that can serve as a baseline in environmental change and pollution studies (physicochemical and trace element) for Antarctic systems.

## 1 Data description

**Fig. 1:**
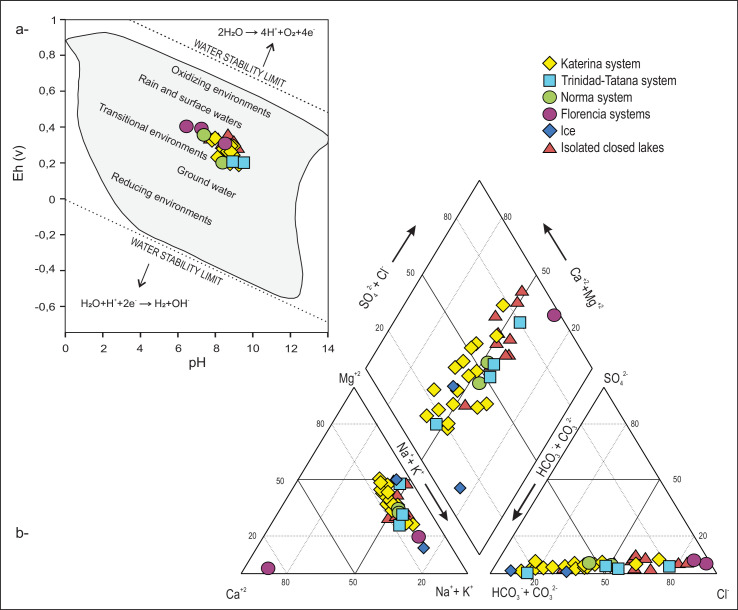
a) pH-Eh diagram; b) Piper diagram from Clearwater Mesa lake and ice water samples.

Water, ice, and sediment samples from 35 different lakes and ponds were collected during the austral summer (15–29 January 2015) on Clearwater Mesa (CWM), located on James Ross Island, northeast Antarctic Peninsula [1]. Fig. 1a shows the different hydrogeochemical environments defined according to the relationship between pH vs. Eh (adapted from [4]). Water ionic classification is shown in Fig. 1b with a Piper diagram [5].

**Fig. 2:**
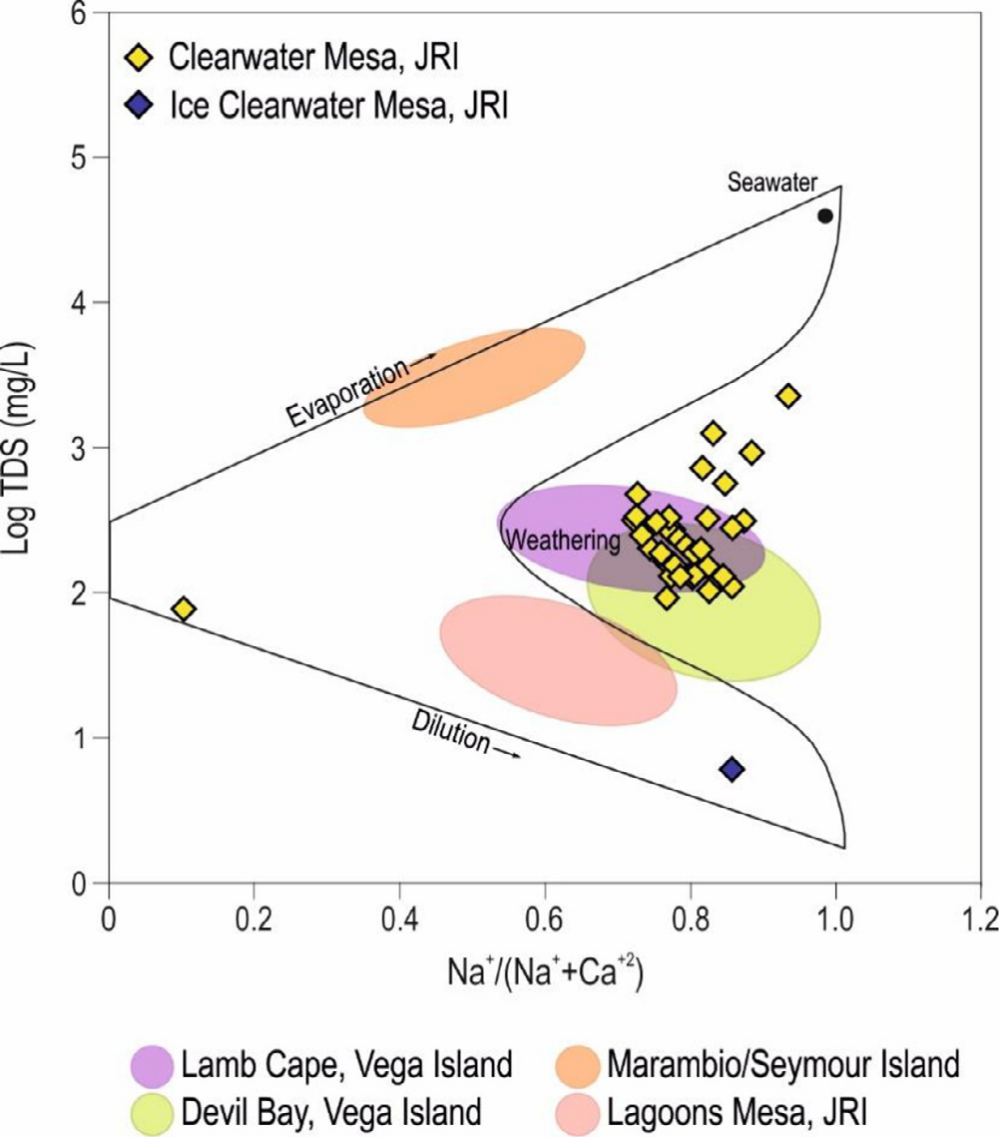
Gibbs Diagram of Clearwater Mesa samples.

Physico-chemical parameters and major ion concentrations were determined for 37 lake and ice samples, and are presented in Table 1. This table also indicates lake area; Mg^2+^/Ca^2+^ and Na^+^/(Na^+^+Ca^2+^) ratios; and sediment TOC and TIC. Fig. 2 shows TDS values vs. Na^+^/(Na^+^+Ca^2+^) ratios in a Gibbs diagram [6]. Table 2 displays the hydrochemical statistical values for each hydrogeological system.

**Table 1:** Sampling points’ location and lake's phisical and chemical characteristics. It is included TIC and TOC data for CWM lakes.

System	Lake	Jan 2015	Surface	pH	Eh	T	Conduc	TDS	T Alk	Mg^+2^	Ca^+2^	Na^+^	K^+^	HCO_3_^−^	CO_3_^−2^	Cl^−^	SO_4_^−2^	Balance	TOC	TIC	Na/ Na+Ca	Mg/Ca
			m^2^		m V	°C	μS cm^−1^	Mg L^−1^											%		meq *L* ^−^ ^1^	
Isolated closed lakes	Adriana	23	18,010	8.66	315.1	10.5	4490.0	2240	156.2	94.9	12.3	178.4	3.6	156.2	0.0	455.3	37.4	0.3	2.4	0.4	0.9	12.9
	Adru	20	1840	9.00	369.9	12.6	260.0	131.1	43.4	7.1	6.5	27.1	5.2	42.0	1.4	45.0	6.4	2	2.0	0.3	0.8	1.8
	Andrea	18	20,630	7.69	394.9	7.7	935.0	470.0	156.5	24.7	29.7	79.5	2.6	156.5	0.0	152.5	7.4	0.4	4.8	0.3	0.7	1.4
	Joanna	24	5260	8.52	341.0	16.5	312.0	156.9	42.3	9.6	6.7	23.1	1.3	42.3	0.0	44.2	8.9	0.6	2.0	0.2	0.7	2.4
	Juanita	23	5680	9.14	346.2	9.4	654.0	327.0	209.5	17.6	17.7	59.6	1.3	186.7	22.8	62.0	5.5	−6.8	1.5	0.6	0.7	1.7
	María	24	2510	8.34	361.3	15.2	267.0	135.5	50.4	7.3	5.6	23.2	1.0	50.4	0.0	34.3	4.4	0.5	3.0	0.3	0.8	2.2
	Martina	24	820	8.60	360.9	16.6	2490.0	1240	121.4	90.5	38.4	190.0	4.2	121.4	0.0	521.3	44.9	0.3	1.2	0.5	0.8	3.9
	Natasha	17	13,300	8.58	410.4	1.4	1126.0	564.0	144.4	49.5	14.2	79.1	2.9	140.8	3.6	204.4	7.0	−0.3	1.3	0.3	0.8	5.8
	Natasha Ice	18	–	6.52	455.4	–	609.0	305.0	250.5	37.9	8.8	59.9	1.5	250.5	0.0	71.2	2.0	0.3	–	–		
	Soledad	24	5030	8.72	310.8	15.7	263.0	129.3	28.3	4.9	3.2	17.5	0.8	28.3	0.0	27.5	4.2	0.4	2.6	0.5	0.8	2.6
	Tamara	23	21,600	7.90	394.0	8.6	300.0	150.0	41.1	9.0	6.2	29.1	0.8	41.1	0.0	52.5	7.8	0.4	1.9	0.3	0.8	2.4
Katerina	Adela	25	18,010	8.84	353.0	15.5	392.0	197.2	151.2	12.7	7.8	34.4	1.3	133.2	18.0	20.9	7.5	−8.8	–	–	0.8	2.7
	Alejandra	27	27,480	8.82	277.0	15.5	367.0	184.6	100.8	12.9	9.5	30.1	1.0	88.8	12.0	44.3	6.8	−6.2	1.6	0.5	0.7	2.3
	Claudina	27	30,520	8.76	304.7	16.8	255.0	128.3	101.6	8.2	6.4	23.6	0.8	98.0	3.6	12.5	2.8	−2.6	2.0	0.3	0.8	2.1
	Graciela	25	30,070	8.74	308.9	18.5	603.0	302.0	58.3	24.1	15.0	46.0	1.7	45.1	13.2	128.6	17.6	−4.1	2.2	0.2	0.7	2.7
	Ileana	21	6630	7.77	385.2	14.3	255.0	128.5	34.6	7.4	5.3	18.1	1.0	34.6	0.0	36.0	3.7	0.7	2.3	0.3	0.7	2.3
	Karina	22	20,760	7.92	399.3	11.3	328.0	164.0	142.5	15.8	7.3	23.8	1.0	142.5	0.0	11.8	1.4	0.4	2.4	0.2	0.7	3.6
	Katerina	21	126,950	8.37	342.4	14.1	496.0	248.0	191.1	25.5	11.9	32.8	1.2	191.1	0.0	31.2	5.1	0.3	2.5	0.4	0.7	3.6
																			4.4	0.4		
																			14.3	0.4		
	Linda	25	20,550	8.73	305.9	18.5	651.0	326.0	157.7	28.2	18.3	49.0	2.0	142.1	15.6	95.7	16.2	−4.2	2.3	0.3	0.7	2.6
	Ludmila	25	10,000	8.67	335.2	15.3	203.0	102.3	65.4	5.8	4.9	23.3	0.7	65.4	0.0	22.0	1.8	0.3	2.3	0.2	0.8	2.0
	Marta	28	2740	8.78	274.9	18.5	415.0	208.0	103.8	14.7	9.3	36.1	1.4	95.4	8.4	53.9	7.6	−3.8	2.2	0.4	0.8	2.6
	Nora	28	65,720	8.75	275.6	15.9	465.0	233.0	131.2	22.2	9.6	35.0	1.3	120.4	10.8	60.0	7.8	−4.1	1.8	0.3	0.8	3.9
	Paula	28	14,890	9.21	263.3	17.2	403.0	201.0	150.3	16.4	12.7	37.1	1.8	132.3	18.0	45.3	7.3	−7.1	1.8	0.3	0.7	2.2
	Sandra	28	1830	8.04	300.6	12.0	291.0	111.6	59.5	5.5	4.1	24.4	0.8	59.5	0.0	24.0	3.3	0.4	–	–	0.8	2.2
	Sara	28	6630	9.10	262.8	17.6	533.0	267.0	127.1	25.9	12.5	43.6	1.7	117.5	9.6	88.7	10.7	−2.9	2.3	0.4	0.8	3.5
	Silvia	21	37,350	8.75	341.5	13.0	630.0	315.0	197.7	29.4	17.8	46.4	1.8	197.7	0.0	64.0	13.4	0.4	1.6	0.2	0.7	2.8
	Susan	22	20,130	7.92	398.3	11.3	181.0	91.2	69.2	7.7	4.1	13.6	0.8	69.2	0.0	9.1	1.7	0.4	2.4	0.3	0.7	3.1
	Valentina	28	3910	9.22	263.3	19.1	506.0	253.0	180.9	18.4	12.6	43.5	1.9	170.1	10.8	39.5	6.3	−3.8	2.3	0.3	0.8	2.4
Trinidad-Tatana	Esther	29	14,080	9.43	271.2	16.3	302.0	152.1	61.5	6.7	7.7	27.1	0.8	51.9	9.6	41.5	4.6	−6.9	2.7	0.3	0.8	1.5
	Tatana	29	5350	9.02	275.5	16.0	1427.0	715.0	304.7	44.8	32.4	145.0	2.6	295.1	9.6	229.8	14.1	−1.1	0.9	0.6	0.8	2.3
	Argentina	29	16,820	9.32	279.2	13.3	642.0	321.0	42.2	15.7	10.7	50.1	1.1	31.4	10.8	118.0	7.8	−4	–	–	0.8	2.4
	Trinidad	29	65,370	8.85	277.5	13.0	1823.0	912.0	775.9	87.6	20.0	154.0	3.0	763.9	12.0	83.1	1.8	−1.1	1.6	0.3	0.9	7.3
Norma	Joaquina	15	2870	8.33	270.5	8.1	367.0	184.7	109.9	13.3	9.6	39.9	1.4	109.9	0.0	46.9	8.9	0.4	1.7	0.3	0.8	2.3
	Norma	15	610	7.31	412.9	6.8	248.0	125.5	57.2	8.8	5.9	24.2	0.8	57.2	0.0	36.9	4.5	0.3	0.8	0.1	0.8	2.5
Florencia	Cecilia	18	6860	8.47	378.3	7.4	556.0	278.0	6.8	8.4	8.8	53.3	2.3	6.8	0.0	116.0	8.5	−0.8	2.2	0.3	0.8	1.6
	Blancmange Glacier	18	–	6.40	458.8	–	11.7	5.9	54.9	1.7	2.7	16.5	0.6	54.9	0.0	2.7	0.6	0.3	–	–	0.8	1.0
	Florencia	18	∼132,000	7.22	446.8	2.0	152.9	76.7	3.4	0.2	13.0	1.5	0.3	3.4	0.0	22.2	2.4	−0.5	2.2	0.3	0.1	0.0

**Table 2:** Statistical values for each hydrogeological system's physical and chemical characteristics.

System		pH	Eh	T	Conduc.	TDS	T Alk	Mg^+2^	Ca^+2^	Na^+^	K^+^	HCO_3_^−^	CO_3_^−2^	Cl^−^	SO_4_^−2^	TOC	TIC	Na/(Na+Ca)	Mg/Ca
			m V	°C	μS cm^−1^	mg L^−1^										%		meq *L* ^−^ ^1^	
Isolated closed lakes	Maximum	9.1	455	16.6	4490	2240	250.5	94.9	38.4	190	5.2	250.5	22.8	521	44.9	4.8	0.6	0.93	12.9
	Minimum	6.5	311	1.4	260	129.3	28.3	4.9	3.2	17.5	0.8	28.3	0.0	27.5	2.0	1.2	0.2	0.70	1.4
	Mean	8.3	369	11.4	1064	531.7	113.1	32.1	13.6	69.7	2.3	110.6	2.5	152	12.4	2.3	0.4	0.80	3.7
	Median	8.6	361	11.6	609	305.0	121.4	17.6	8.8	59.6	1.5	121.4	0.0	62.0	7.0	2.0	0.3	0.79	2.4
Katerina	Maximum	9.2	399	19.1	651	326.0	197.7	29.4	18.3	49.0	2.0	197.7	18.0	129	17.6	14.3	0.5	0.84	3.9
	Minimum	7.8	263	11.3	181	91.2	34.6	5.5	4.1	13.6	0.7	34.6	0.0	9.1	1.4	1.6	0.2	0.69	2.0
	Mean	8.6	317	15.6	410	203.6	119.0	16.5	9.9	33.0	1.3	111.9	7.1	46.3	7.1	3.0	0.3	0.75	2.7
	Median	8.8	306	15.5	403	201.0	127.1	15.8	9.5	34.4	1.3	117.5	8.4	39.5	6.8	2.3	0.3	0.75	2.6
Trinidad-Tatana	Maximum	9.4	279	16.3	1823	912.0	775.9	87.6	32.4	154	3.0	763.9	12.0	230	14.1	2.7	0.6	0.87	7.3
	Minimum	8.9	271	13.0	302	152.1	42.2	6.7	7.7	27.1	0.8	31.4	9.6	41.5	1.8	0.9	0.3	0.75	1.5
	Mean	9.2	276	14.7	1049	525.0	296.1	38.7	17.7	94.1	1.9	285.6	10.5	118	7.1	1.7	0.4	0.81	3.4
	Median	9.2	277	14.7	1035	518.0	183.1	30.3	15.4	97.6	1.9	173.5	10.2	101	6.2	1.6	0.3	0.80	2.4
Norma	Maximum	8.3	413	8.1	367	184.7	109.9	13.3	9.6	39.9	1.4	109.9	0.0	46.9	8.9	1.7	0.3	0.78	2.5
	Minimum	7.3	271	6.8	248	125.5	57.2	8.8	5.9	24.2	0.8	57.2	0.0	36.9	4.5	0.8	0.1	0.78	2.3
	Mean	7.8	342	7.5	308	155.1	83.6	11.1	7.8	32.1	1.1	83.6	0.0	41.9	6.7	1.3	0.2	0.78	2.4
	Median	7.8	342	7.5	308	155.1	83.6	11.1	7.8	32.1	1.1	83.6	0.0	41.9	6.7	1.3	0.2	0.78	2.4
Florencia	Maximum	8.5	459	7.4	556	278.0	54.9	8.4	13.0	53.3	2.3	54.9	0.0	116	8.5	2.2	0.3	0.84	1.6
	Minimum	6.4	378	2.0	12	5.9	3.4	0.2	2.7	1.5	0.3	3.4	0.0	2.7	0.6	2.2	0.3	0.09	0.0
	Mean	7.4	428	4.7	240	120.2	21.7	3.4	8.2	23.8	1.1	21.7	0.0	47.0	3.8	2.2	0.3	0.59	0.9
	Median	7.2	447	4.7	153	76.7	6.8	1.7	8.8	16.5	0.6	6.8	0.0	22.2	2.4	2.2	0.3	0.84	1.0
CWM lakes	Maximum	9.4	459	19.1	4490	2240	775.9	94.9	38.4	190	5.2	763.9	22.8	521	44.9	14.3	0.6	0.93	12.9
	Minimum	6.4	263	1.4	12	5.9	3.4	0.2	2.7	1.5	0.3	3.4	0.0	2.7	0.6	0.8	0.1	0.09	0.0
	Mean	8.4	338	13.2	654	326.5	126.6	22.2	11.6	49.7	1.6	121.4	5.1	85.3	8.4	2.5	0.3	0.76	2.9
	Median	8.7	341	14.3	403	201.0	103.8	14.7	9.5	35.0	1.3	98.0	0.0	45.3	6.8	2.2	0.3	0.77	2.4

**Fig. 3:**
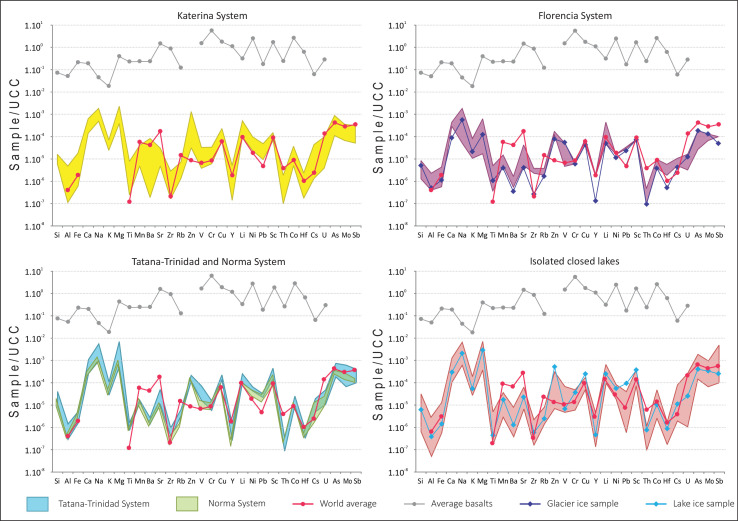
Upper Continental Crust normalized spidergram. World average [8] and James Ross Island Volcanic Group average [9] are added for comparison.

Table 3 presents dissolved trace element concentrations. They were normalized to the Upper Continental Crust [7] and a spidergram is shown in Fig. 3, where World average [8] and the mean James Ross Island Volcanic Group geochemical composition have been indicated [9]. Table 4 shows statistical values for differentiated hydrogeological system's trace elements.

**Table 3:** Trace element determined in the 2015 sampling campaing and JRIVG mean values (in ppm) are from concentrations reported by Košler et al., 2009.

Sample	Si	Al		Fe	Ti	Mn	Ba	Sr	Zr	Rb	Zn	V	Cr	Cu
	mg L^−1^				µg L^−1^									
Detection limit (µg L^−1^)	200	2		10	0.1	0.1	0.1	0.04	0.01	0.005	0.5	0.1	0.5	0.2
Adela	4.80	0.10		0.21	5.60	17.40	2.80	5.21	0.21	0.63	8.70	1.30	2.70	5.50
Adriana	0.20	0.01		0.03	0.70	1.70	0.50	20.40	0.08	0.35	0.50	1.30	4.00	3.90
Adru	3.60	0.06		0.03	8.20	3.60	0.20	2.79	0.09	0.81	16.20	1.60	0.50	2.00
Alejandra	4.30	0.01		0.02	1.70	3.60	0.30	2.89	0.04	0.21	6.20	0.70	0.50	1.70
Andrea	4.10	0.00		0.04	2.20	5.60	0.20	22.30	0.16	0.47	6.10	7.30	1.40	1.60
Argentina	3.80	0.12		0.19	7.00	9.50	1.40	4.04	0.19	0.50	11.00	2.60	0.50	3.60
Cecilia	2.60	0.03		0.02	1.50	3.20	0.30	15.00	0.73	0.43	8.90	5.70	0.60	1.10
Claudina	2.50	0.01		0.02	1.00	2.80	0.10	2.45	0.03	0.14	2.20	0.50	0.50	1.60
Ester	3.40	0.09		0.12	3.60	10.10	1.60	3.99	0.08	0.37	8.60	2.20	0.70	3.50
Blancmange Glacier	1.60	0.04		0.04	4.50	2.40	0.20	1.45	0.05	0.19	5.50	6.00	0.50	1.10
Florencia	0.40	0.19		0.15	20.90	9.10	0.90	1.81	0.44	0.21	12.10	0.50	0.50	2.00
Graciela	3.30	0.13		0.19	8.20	11.70	2.60	6.55	0.16	0.50	12.10	1.00	0.70	3.40
Ileana	2.80	0.10		0.26	7.60	7.70	1.50	2.87	0.10	0.30	6.10	0.60	0.60	2.40
Joanna	4.20	0.08		0.12	5.10	13.60	1.70	4.67	0.37	0.64	21.30	0.60	1.20	2.80
Joaquina	6.00	0.03		0.07	3.10	5.60	0.60	4.35	0.07	0.17	10.90	1.60	1.00	1.90
Juanita	5.70	0.01		0.02	2.00	4.30	0.20	5.40	0.06	0.25	6.80	2.70	0.60	1.30
Karina	2.80	0.07		0.13	3.80	7.90	4.32	3.59	0.10	0.21	5.80	0.50	0.60	2.90
Katerina	3.00	0.10		0.19	6.10	14.00	2.60	7.21	0.11	0.26	6.90	0.70	0.70	2.50
Linda	4.70	0.32		0.59	22.50	15.60	2.10	7.60	0.40	0.66	11.10	1.70	1.00	4.40
Ludmila	3.60	0.05		0.07	2.50	7.70	1.10	1.63	0.03	0.26	9.90	0.80	0.80	3.10
Maria	3.80	0.07		0.06	3.00	11.70	2.10	3.19	0.11	0.42	12.90	0.50	0.80	2.70
Marta	4.40	0.09		0.09	6.60	5.80	1.30	5.80	0.13	0.26	4.00	2.00	0.80	1.90
Martina	9.70	0.18		0.11	5.80	18.10	4.40	21.10	0.22	0.91	14.00	1.70	0.50	4.20
Natasha	1.90	0.03		0.05	1.80	10.40	0.70	7.98	0.11	0.28	37.00	0.70	3.00	6.30
Natasha Ice	3.80	0.22		0.45	20.50	21.70	1.50	12.90	0.42	0.53	14.70	1.70	0.90	4.00
Nora	3.00	0.09		0.14	6.30	8.50	1.50	7.12	0.11	0.30	2.90	1.30	0.50	2.00
Norma	2.60	0.03		0.13	2.70	11.80	0.90	2.61	0.03	0.12	11.10	0.70	0.50	2.20
Paula	4.40	0.40		0.62	30.50	23.60	2.30	9.96	0.53	0.78	91.50	3.20	1.00	4.10
Sandra	3.50	0.06		0.03	2.40	8.40	2.00	2.96	0.04	0.31	8.50	0.70	0.60	2.00
Sara	4.10	0.14		0.16	5.70	17.70	4.60	9.64	0.09	0.43	13.80	1.60	0.80	4.00
Silvia	2.90	0.02		0.03	2.10	5.30	0.30	7.36	0.03	0.29	4.90	0.80	0.60	2.50
Soledad	2.70	0.17		0.25	12.90	11.90	1.80	2.39	0.16	0.39	10.90	1.00	0.60	2.70
Susan	1.80	0.20		0.14	3.90	11.20	3.20	3.04	0.06	0.34	11.50	0.40	0.50	2.70
Tamara	2.40	0.02		0.03	1.50	7.40	0.80	3.88	0.03	0.16	6.30	0.60	0.80	1.70
Tatana	12.60	0.10		0.11	7.00	8.80	1.60	17.90	0.16	0.61	9.00	7.80	1.30	5.60
Trinidad	2.70	0.03		0.06	3.20	8.30	0.80	15.80	0.18	0.71	15.20	1.80	1.10	3.50
Valentina	4.70	0.16		0.18	10.20	12.80	2.20	8.85	0.20	0.41	4.40	3.40	0.60	2.30
Basalts mean values	22.22	4.06		7.40	917.80	139.41	126.88	505.63	163.50	13.60	–	160.38	458.13	43.39

Sample	Y	Li		Ni	Pb	*Sc*	Th	Co	Hf	Cs	U	As	Mo	Sb
	µg L^−1^													
Detection limit (µg L^−1^)	0.003		1	0.3	0.01	1	0.001	0.005	0.001	0.001	0.001	0.03	0.1	0.01
Adela	0.06		7.00	2.10	0.22	1.00	0.00	0.55	0.00	0.10	0.08	0.60	0.30	0.04
Adriana	0.01		5.00	1.10	0.01	1.00	0.00	0.04	0.00	0.21	0.44	2.80	1.40	0.05
Adru	0.00		3.00	0.90	0.34	2.00	0.00	0.14	0.00	0.03	0.01	0.47	0.10	0.98
Alejandra	0.00		2.00	1.40	0.45	2.00	0.00	0.10	0.00	0.20	0.06	0.44	0.30	0.02
Andrea	0.01		2.00	1.60	0.51	2.00	0.00	0.16	0.00	0.10	0.14	0.55	0.30	0.02
Argentina	0.03		2.00	1.90	0.38	2.00	0.00	0.32	0.01	0.09	0.04	0.42	0.20	0.08
Cecilia	0.04		1.00	0.70	0.50	1.00	0.01	0.07	0.03	0.05	0.05	0.28	0.20	0.02
Claudina	0.00		2.00	1.10	0.16	1.00	0.00	0.08	0.00	0.02	0.04	0.40	0.20	0.01
Ester	0.01		3.00	2.70	0.40	2.00	0.00	0.43	0.00	0.02	0.03	0.27	0.10	0.02
Blancmange Glacier	0.00		1.00	0.50	0.40	1.00	0.00	0.07	0.00	0.02	0.04	0.28	0.20	0.01
Florencia	0.04		9.00	0.70	0.58	1.00	0.00	0.17	0.01	0.02	0.01	0.04	0.10	0.02
Graciela	0.03		9.00	1.90	0.69	2.00	0.01	0.38	0.01	0.02	0.15	0.58	0.30	0.02
Ileana	0.04		2.00	2.00	0.53	1.00	0.00	0.24	0.00	0.02	0.04	0.45	0.10	0.02
Joanna	0.02		11.00	1.90	0.45	2.00	0.00	0.40	0.00	0.02	0.02	0.28	0.20	0.04
Joaquina	0.01		2.00	2.10	0.49	3.00	0.00	0.22	0.00	0.06	0.07	0.51	0.40	0.03
Juanita	0.00		6.00	0.80	0.20	3.00	0.00	0.06	0.00	0.01	0.06	0.80	0.30	0.02
Karina	0.01		4.00	1.40	0.28	1.00	0.00	0.26	0.00	0.02	0.06	0.33	0.10	0.01
Katerina	0.02		5.00	1.80	0.26	1.00	0.00	0.36	0.00	0.03	0.10	0.60	0.20	0.03
Linda	0.10		10.00	2.50	0.52	2.00	0.01	0.40	0.01	0.03	0.23	0.79	0.30	0.04
Ludmila	0.00		5.00	1.10	0.37	2.00	0.00	0.23	0.00	0.01	0.01	0.17	0.10	0.01
Maria	0.00		13.00	1.20	0.35	2.00	0.00	0.34	0.00	0.03	0.02	0.22	0.10	0.02
Marta	0.01		3.00	1.30	0.23	2.00	0.01	0.27	0.00	0.02	0.06	0.60	0.30	0.03
Martina	0.03		13.00	2.80	0.36	5.00	0.00	0.81	0.01	0.07	0.73	1.37	1.20	0.05
Natasha	0.01		5.00	2.40	1.57	5.00	0.01	0.17	0.01	0.05	0.07	0.60	0.50	0.05
Natasha Ice	0.10		6.00	3.60	0.66	2.00	0.01	0.33	0.01	0.13	0.27	1.17	0.40	0.05
Nora	0.01		5.00	1.50	0.25	1.00	0.01	0.28	0.00	0.01	0.08	0.50	0.20	0.03
Norma	0.00		2.00	1.10	0.23	1.00	0.00	0.21	0.00	0.01	0.02	0.36	0.20	0.02
Paula	0.10		8.00	2.20	0.77	2.00	0.02	0.60	0.01	0.03	0.17	1.17	0.30	0.06
Sandra	0.00		3.00	1.00	0.24	2.00	0.00	0.37	0.00	0.01	0.03	0.25	0.10	0.03
Sara	0.11		5.00	2.30	0.55	2.00	0.00	0.36	0.00	0.02	0.12	0.83	0.40	0.04
Silvia	0.00		7.00	1.80	0.22	1.00	0.00	0.08	0.00	0.07	0.26	0.73	0.30	0.04
Soledad	0.04		12.00	1.30	0.45	1.00	0.00	0.55	0.00	0.37	0.02	0.26	0.10	0.04
Susan	0.02		4.00	4.00	0.55	1.00	0.01	0.30	0.00	0.09	0.02	0.23	0.10	0.03
Tamara	0.00		5.00	1.50	0.51	1.00	0.00	0.14	0.00	0.02	0.00	0.48	0.20	0.02
Tatana	0.01		3.00	2.60	0.56	6.00	0.00	0.33	0.00	0.09	0.14	1.09	0.90	0.05
Trinidad	0.01		5.00	2.90	0.38	2.00	0.00	0.17	0.00	0.05	0.13	0.97	0.40	0.04
Valentina	0.03		6.00	1.60	0.28	2.00	0.01	0.44	0.01	0.01	0.27	1.27	0.50	0.06
Basalts mean values	23.94		6.19	108.38	2.94	0.00	22.78	2.58	43.78	3.54	0.13	0.97	–	–

**Table 4:** Statistical values for each hydrogeological system's trace elements.

System		Si	Al	Fe	Ti	Mn	Ba	Sr	Zr	Rb	Zn	V	Cr	Cu	Y	Li	Ni	Pb	Sc	Th	Co	Hf	Cs	U	As	Mo	Sb
		Mg L^−1^			µg L^−1^																						
Isolated closed lakes	Max	9.7	0.2	0.3	12.9	18.1	4.4	22.3	0.4	0.9	37.0	7.3	4.0	6.3	0.042	13.0	2.8	1.6	5.0	0.008	0.8	0.006	0.37	0.73	2.80	1.4	0.98
	Min	0.2	0.0	0.0	0.70	1.7	0.2	2.4	0.0	0.2	0.5	0.5	0.5	1.3	0.003	2.0	0.8	0.0	1.0	0.001	0.0	0.001	0.01	0.00	0.22	0.1	0.02
	Mean	3.8	0.1	0.1	4.32	8.8	1.3	9.4	0.1	0.5	13.2	1.8	1.3	2.9	0.013	7.5	1.6	0.5	2.4	0.003	0.3	0.003	0.09	0.15	0.78	0.4	0.13
	Median	3.7	0.0	0.0	2.60	8.9	0.8	5.0	0.1	0.4	11.9	1.2	0.8	2.7	0.008	5.5	1.4	0.4	2.0	0.002	0.2	0.003	0.04	0.04	0.52	0.3	0.04
Katerina	Max	4.8	0.4	0.6	30.5	23.6	4.6	10.0	0.5	0.8	91.5	3.4	2.7	5.5	0.109	10.0	4.0	0.8	2.0	0.019	0.6	0.013	0.20	0.27	1.27	0.5	0.06
	Min	1.8	0.0	0.0	1.00	2.8	0.1	1.6	0.0	0.1	2.2	0.4	0.5	1.6	0.003	2.0	1.0	0.2	1.0	0.001	0.1	0.001	0.01	0.01	0.17	0.1	0.01
	Mean	3.6	0.1	0.2	7.45	10.7	2.0	5.6	0.1	0.4	12.4	1.2	0.8	2.9	0.033	5.1	1.8	0.4	1.5	0.005	0.3	0.004	0.04	0.10	0.58	0.2	0.03
	Median	3.5	0.1	0.1	5.70	8.5	2.1	5.8	0.1	0.3	6.9	0.8	0.6	2.5	0.020	5.0	1.8	0.3	2.0	0.004	0.3	0.003	0.02	0.08	0.58	0.3	0.03
Trinidad-Tatana	Max	12.6	0.1	0.2	7.00	10.1	1.6	17.9	0.2	0.7	15.2	7.8	1.3	5.6	0.033	5.0	2.9	0.6	6.0	0.004	0.4	0.005	0.09	0.14	1.09	0.9	0.08
	Min	2.7	0.0	0.1	3.20	8.3	0.8	4.0	0.1	0.4	8.6	1.8	0.5	3.5	0.006	2.0	1.9	0.4	2.0	0.001	0.2	0.002	0.02	0.03	0.27	0.1	0.02
	Mean	5.6	0.1	0.1	5.20	9.2	1.4	10.4	0.2	0.5	11.0	3.6	0.9	4.1	0.014	3.3	2.5	0.4	3.0	0.003	0.3	0.004	0.06	0.08	0.69	0.4	0.05
	Median	3.6	0.1	0.1	5.30	9.2	1.5	9.9	0.2	0.6	10.0	2.4	0.9	3.6	0.008	3.0	2.7	0.4	2.0	0.003	0.3	0.004	0.07	0.08	0.70	0.3	0.05
Norma	Max	6.0	0.0	0.1	3.10	11.8	0.9	4.4	0.1	0.2	11.1	1.6	1.0	2.2	0.006	2.0	2.1	0.5	3.0	0.002	0.2	0.003	0.06	0.07	0.51	0.4	0.03
	Min	2.6	0.0	0.1	2.70	5.6	0.6	2.6	0.0	0.1	10.9	0.7	0.5	1.9	0.003	2.0	1.1	0.2	1.0	0.002	0.2	0.002	0.01	0.02	0.36	0.2	0.02
	Mean	4.3	0.0	0.1	2.90	8.7	0.8	3.5	0.1	0.1	11.0	1.2	0.8	2.1	0.005	2.0	1.6	0.4	2.0	0.002	0.2	0.003	0.03	0.04	0.44	0.3	0.03
	Median	4.3	0.0	0.1	2.90	8.7	0.8	3.5	0.1	0.1	11.0	1.2	0.8	2.1	0.005	2.0	1.6	0.4	2.0	0.002	0.2	0.003	0.03	0.04	0.44	0.3	0.03
Florencia	Max	2.6	0.2	0.2	20.9	9.1	0.9	15.0	0.7	0.4	12.1	6.0	0.6	2.0	0.042	9.0	0.7	0.6	1.0	0.005	0.2	0.033	0.05	0.05	0.28	0.2	0.02
	Min	0.4	0.0	0.0	1.50	2.4	0.2	1.5	0.1	0.2	5.5	0.5	0.5	1.1	0.003	1.0	0.5	0.4	1.0	0.001	0.1	0.003	0.02	0.01	0.04	0.1	0.01
	Mean	1.5	0.1	0.1	8.97	4.9	0.5	6.1	0.4	0.3	8.8	4.1	0.5	1.4	0.027	3.7	0.6	0.5	1.0	0.002	0.1	0.016	0.03	0.03	0.20	0.2	0.02
	Median	1.6	0.0	0.0	4.50	3.2	0.3	1.8	0.4	0.2	8.9	5.7	0.5	1.1	0.037	1.0	0.7	0.5	1.0	0.001	0.1	0.011	0.02	0.04	0.28	0.2	0.02
CWM lakes	Max	12.6	0.4	0.6	30.5	23.6	4.6	22.3	0.7	0.9	91.5	7.8	4.0	6.3	0.109	13.0	4.0	1.6	6.0	0.019	0.8	0.033	0.37	0.73	2.80	1.4	0.98
	Min	0.2	0.0	0.0	0.70	1.7	0.1	1.5	0.0	0.1	0.5	0.4	0.5	1.1	0.003	1.0	0.5	0.0	1.0	0.001	0.0	0.001	0.01	0.00	0.04	0.1	0.01
	Mean	3.7	0.1	0.1	6.59	9.7	1.5	7.3	0.2	0.4	12.1	1.9	0.9	2.9	0.025	5.3	1.8	0.4	1.9	0.004	0.3	0.005	0.06	0.11	0.63	0.3	0.06
	Median	3.5	0.1	0.1	4.50	8.8	1.5	5.2	0.1	0.4	9.0	1.3	0.7	2.7	0.013	5.0	1.6	0.4	2.0	0.002	0.3	0.003	0.03	0.06	0.50	0.2	0.03

**Fig. 4:**
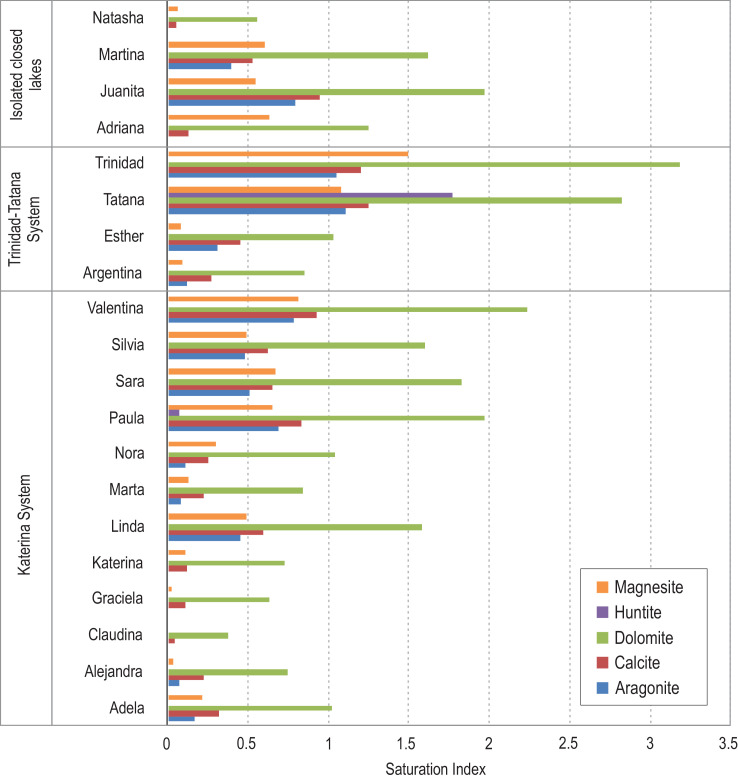
Mineral Saturation Index (SI) calculated with PHREEQC.

PHREEQC models are presented in the Software Output, which is included in the *Supplementary Material*. It comprises ion speciation, Saturation Indexes, and geochemical models (i.e. mixing modelling between Blancmange Glacier and Cecilia samples and inverse modelling between Blancmange Glacier and Florencia samples). Finally, Fig. 4 illustrates those minerals that are prone to precipitate due to their positive Saturation Index (SI).

## 2 Experimental design, materials, and methods

### 2.1 Sampling and analyses

Water and sediment samples were collected from 35 different lakes and ponds on Clearwater Mesa during the 2015 Antarctic summer field campaign following standard protocols [3]. Two ice samples (i.e., Blancmange Glacier and Lake Natasha-ice), were also collected, along with a precipitated salt sample from the margin of Lake Andrea.

Water temperature, pH, redox potential, electrical conductivity, TDS, and alkalinity were measured at each waterbody *in situ*. Redox potential and pH were measured with a Hach digital detector, while temperature, TDS, and electrical conductivity were measured using a digital Hach conductivimeter. Alkalinity was measured as CaCO_3_ by end point titration in the field, and using a 0.16 N H_2_SO_4_ solution until pH = 4.5. For the determination of anions, major cations, and trace elements, samples were vacuum-filtered in the field with 0.22 µm pore size cellulose filters (HA-type, Millipore Corp.). An aliquot was stored in polyethylene bottles at 4 °C for the determination of chloride and sulphate by chemically suppressed ion chromatography with conductivity detection. The other aliquot was acidified (pH< 2) with concentrated, redistilled and ultrapure HNO_3_ (Sigma-Aldrich) for the analytical determination of major and trace elements by inductively coupled plasma-mass spectrometry (ICP-MS, Activation Laboratories Ltd., Ancaster, Ontario, Canada). Major and trace elements concentrations were validated using NIST (National Institute of Standards and Technology) 1640 and Riverine Water Reference Materials for Trace Metals certified by the National Research Council of Canada (SRLS-4), and detection limits are reported in the corresponding tables. Statistical parameters (i.e. mean and median) were calculated for each hydrogeological system's trace elements and physical and chemical characteristics.

### 2.2 Sediment analysis

Bottom lake sediment samples were collected from 32 shallow lakes using a standard scoop sampler. Lake Katerina was sampled 3 times in order to characterize spatial variability in this large lake. Particular attention was paid to collect the sediment/water interface. Samples were skimmed from the benthic surface (top 2–3 cm), stored in plastic bags, and refrigerated until analysis.

TOC and TIC was determined in sediment samples using the loss on ignition method (LOI; [10]). Prior to their analysis by LOI, they were partitioned and lyophilized in order to remove water by sublimation. To prepare the samples, sediment was first ground to obtain a homogeneous sample. A quantity of 2 g was then weighed on a high-precision analytical balance (Precisa XR 2058- DR) and placed in numbered crucibles. Each sample was weighed to obtain the initial weight. The organic matter content was determined by calculating the difference in mass between sediment samples dried to a constant weight at 105 °C, and after furnacing at 550 °C for 4 h. Carbonate content was calculated as the mass loss after burning the LOI residue at 950 °C for 2 h [10].

### 2.3 Geochemical modelling

Chemical information was processed with PHREEQC [2] constructed using the AQUACHEM PHREEQC interface. These programs were used to calculate the SI for different mineral phases, element speciation in the given conditions (i.e. the element distribution in all the possible dissolved chemical species that can be found in the samples), and geochemical weathering and mixing models. Software outputs are presented in the *Supplementary Material*. The SI is the ratio between the ion activity product for the given material and the reaction constant at a given temperature. When SI > 0, it means that the solution is supersaturated with respect to the mineral phase and may therefore precipitate, whereas when SI < 0 the solution is below saturation of the specified mineral.

Geochemical modelling uses the interaction of cations and anions as a function of temperature, redox potential, pH, and ionic strength. Inverse modelling was performed to quantify weathering processes occurring, first between connected lakes Esther and Tatana, and second between Blancmange Glacier and Lake Florencia. Inverse modelling begins with known initial and final solutions, and available mineral phases which attempts to quantify the processes that lead to the final solution chemistry (e.g., [11]). In the process, all possible combinations of dissolution and/or precipitation reactions explaining chemical changes observed between the two solutions and the mineral phases are reconstructed [12]. Models were performed under equilibrium with O_2 (g)_ given that these are surficial hydrological systems. Finally, three mixing models with different proportions of Blancmange Glacier and Lake Cecilia solutions (i.e., one model where both the glacier and lake contribute 50% of the water, one where 20% of the water is from the glacier and 80% is from Lake Cecilia, and one where 80% of the water is from the glacier and 20% is from Lake Cecilia) were made to verify eventual mixing processes.

## Appendix Supplementary materials

Image, application 1
