# Supplementary material for: Data set of dissolved major and trace elements from the lacustrine systems of Clearwater Mesa, Antarctica
Source: Data Brief. 2020 Mar 19;30:105438. doi: 10.1016/j.dib.2020.105438 (PMC7150500; doi:10.1016/j.dib.2020.105438)
Supplement: Supplementary file 1 [file mmc1.pdf]

```
   Input file: C:\Program Files (x86)\AquaChem\PHRin.tmp
  Output file: C:\Program Files (x86)\AquaChem\PHRout.tmp
Database file: C:\Users\Usuario\Documents\AquaChem\PHREEQC\phreeqc.dat

------------------
Reading data base.
------------------

      SOLUTION_MASTER_SPECIES
      SOLUTION_SPECIES
      PHASES
      EXCHANGE_MASTER_SPECIES
      EXCHANGE_SPECIES
      SURFACE_MASTER_SPECIES
      SURFACE_SPECIES
      RATES
      END
------------------------------------
Reading input data for simulation 1.
------------------------------------

      SELECTED_OUTPUT
      file  C:\Program Files (x86)\AquaChem\PHRSelOut.tmp
      reset true
      molalities  HCO3- CO3-2
      TITLE <Enter step description>
      SOLUTION 36 4HFlorencia-1
            units mg/L
            pH    7.0 #value missing
            pe    4
            temp   4.9
            Ca     2.7
            Mg     1.65
            Na     16.5
            K      .6
            Cl     2.708
            S(6)   .56 as SO4
            F      .081
            Ba     .0002
            Cu     1.1  ug/l
            Mn     .0024
            Si     1600 as SiO2
            Zn     5.5  ug/l
            Alkalinity         54.9  as HCO3
      SOLUTION 9 4HNatasha-1
            units mg/L
            pH    7.0 #value missing
            pe    4
            temp   20
            Ca     8.8
            Mg     37.9
            Na     59.9
            K      1.49
            Cl     71.241
            S(6)   2.019 as SO4
            F      .102
            Ba     .0007
            Cu     6.3  ug/l
            Mn     .0104
            Si     1900 as SiO2
            Zn     37   ug/l
            Alkalinity         250.5  as HCO3
      SOLUTION 12 4LAdela-1
            units mg/L
            pH    7.0 #value missing
            pe    4
            temp   15.5
```

```
      Ca     7.8
      Mg     12.7
      Na     34.4
      K      1.3
      Cl     20.869
      S(6)   7.55 as SO4
      F      .096
      Ba     .0028
      Cu     5.5  ug/l
      Mn     .0174
      Si     4800 as SiO2
      Zn     8.7  ug/l
      Alkalinity         206.4093  as HCO3
SOLUTION 1 4LAdriana-1
      units mg/L
      pH    7.0 #value missing
      pe    4
      temp   10.5
      Ca     12.3
      Mg     94.9
      Na     178.3834
      K      3.62
      Cl     455.268
      S(6)   37.43 as SO4
      Ba     .0005
      Cu     3.9  ug/l
      Mn     .0017
      Si     200 as SiO2
      Zn     .5   ug/l
      Alkalinity         156.16  as HCO3
SOLUTION 2 4LAdru-1
      units mg/L
      pH    7.0 #value missing
      pe    4
      temp   12.6
      Ca     6.5
      Mg     7.14
      Na     27.1
      K      5.24
      Cl     44.972
      S(6)   6.37 as SO4
      F      .056
      Ba     .0002
      Cu     2    ug/l
      Mn     .0036
      Si     3600 as SiO2
      Zn     16.2 ug/l
      Alkalinity         47.61272  as HCO3
SOLUTION 13 4LAlejandra-1
      units mg/L
      pH    7.0 #value missing
      pe    4
      temp   15.5
      Ca     9.5
      Mg     12.9
      Na     30.1
      K      1
      Cl     44.2515
      S(6)   6.8 as SO4
      F      .125
      Ba     .0003
      Cu     1.7  ug/l
      Mn     .0036
      Si     4300 as SiO2
      Zn     6.2  ug/l
      Alkalinity         137.5662  as HCO3
SOLUTION 3 4LAndrea-1
```

```
      units mg/L
      pH    7.0 #value missing
      pe    4
      temp   7.7
      Ca     29.7
      Mg     24.7
      Na     79.5
      K      2.63
      Cl     152.4915
      S(6)   7.37 as SO4
      F      .153
      Ba     .0002
      Cu     1.6  ug/l
      Mn     .0056
      Si     4100 as SiO2
      Zn     6.1  ug/l
      Alkalinity         156.5  as HCO3
SOLUTION 31 4LArgentina-1
      units mg/L
      pH    7.0 #value missing
      pe    4
      temp   13.3
      Ca     10.7
      Mg     15.7
      Na     50.1
      K      1.13
      Cl     118
      S(6)   7.79 as SO4
      F      .113
      Ba     .0014
      Cu     3.6  ug/l
      Mn     9.500001E-03
      Si     3800 as SiO2
      Zn     11   ug/l
      Alkalinity         75.29561  as HCO3
SOLUTION 35 4LCecilia-1
      units mg/L
      pH    7.0 #value missing
      pe    4
      temp   7.4
      Ca     8.8
      Mg     8.43
      Na     53.3
      K      2.31
      Cl     115.963
      S(6)   8.52 as SO4
      F      .123
      Ba     .0003
      Cu     1.1  ug/l
      Mn     .0032
      Si     2600 as SiO2
      Zn     8.9  ug/l
      Alkalinity         6.83  as HCO3
SOLUTION 14 4LClaudina-1
      units mg/L
      pH    7.0 #value missing
      pe    4
      temp   16.8
      Ca     6.4
      Mg     8.17
      Na     23.6
      K      .81
      Cl     12.526
      S(6)   2.8 as SO4
      F      .115
      Ba     .0001
      Cu     1.6  ug/l
```

```
      Mn     .0028
      Si     2500 as SiO2
      Zn     2.2  ug/l
      Alkalinity         112.6419  as HCO3
SOLUTION 29 4LEsther-1
      units mg/L
      pH    7.0 #value missing
      pe    4
      temp   16.3
      Ca     7.7
      Mg     6.71
      Na     27.1
      K      .8
      Cl     41.47
      S(6)   4.57 as SO4
      F      .3415
      Ba     .0016
      Cu     3.5  ug/l
      Mn     .0101
      Si     3400 as SiO2
      Zn     8.6  ug/l
      Alkalinity         90.90498  as HCO3
SOLUTION 37 4LFlorencia-1
      units mg/L
      pH    7.0 #value missing
      pe    4
      temp   2
      Ca     13
      Mg     .23
      Na     1.5
      K      .3
      Cl     22.196
      S(6)   2.44 as SO4
      F      .137
      Ba     .0009
      Cu     2    ug/l
      Mn     .0091
      Si     400 as SiO2
      Zn     12.1 ug/l
      Alkalinity         3.42  as HCO3
SOLUTION 15 4LGraciela-1
      units mg/L
      pH    7.0 #value missing
      pe    4
      temp   18.5
      Ca     15
      Mg     24.1
      Na     46
      K      1.68
      Cl     128.584
      S(6)   17.59 as SO4
      F      .125
      Ba     .0026
      Cu     3.4  ug/l
      Mn     .0117
      Si     3300 as SiO2
      Zn     12.1 ug/l
      Alkalinity         98.76685  as HCO3
SOLUTION 16 4LIleana-1
      units mg/L
      pH    7.0 #value missing
      pe    4
      temp   14.3
      Ca     5.3
      Mg     7.39
      Na     18.1
      K      .97
```

```
      Cl     36.036
      S(6)   3.67 as SO4
      F      .053
      Ba     .0015
      Cu     2.4  ug/l
      Mn     .0077
      Si     2800 as SiO2
      Zn     6.1  ug/l
      Alkalinity         34.58  as HCO3
SOLUTION 4 4LJoanna-1
      units mg/L
      pH    7.0 #value missing
      pe    4
      temp   16.5
      Ca     6.7
      Mg     9.55
      Na     23.1
      K      1.34
      Cl     44.157
      S(6)   8.91 as SO4
      F      .083
      Ba     .0017
      Cu     2.8  ug/l
      Mn     .0136
      Si     4200 as SiO2
      Zn     21.3 ug/l
      Alkalinity         42.3  as HCO3
SOLUTION 33 4LJoaquina-1
      units mg/L
      pH    7.0 #value missing
      pe    4
      temp   8.1
      Ca     9.6
      Mg     13.3
      Na     39.9
      K      1.38
      Cl     46.874
      S(6)   8.9 as SO4
      F      .129
      Ba     .0006
      Cu     1.9  ug/l
      Mn     .0056
      Si     6000 as SiO2
      Zn     10.9 ug/l
      Alkalinity         109.89  as HCO3
SOLUTION 5 4LJuanita-1
      units mg/L
      pH    7.0 #value missing
      pe    4
      temp   9.4
      Ca     17.7
      Mg     17.6
      Na     59.6
      K      1.26
      Cl     62.006
      S(6)   5.46 as SO4
      Ba     .0002
      Cu     1.3  ug/l
      Mn     4.300001E-03
      Si     5700 as SiO2
      Zn     6.8  ug/l
      Alkalinity         279.4318  as HCO3
SOLUTION 17 4LKarina-1
      units mg/L
      pH    7.0 #value missing
      pe    4
      temp   11.3
```

```
      Ca     7.3
      Mg     15.8
      Na     23.8
      K      .96
      Cl     11.828
      S(6)   1.39 as SO4
      F      .11
      Ba     .0432
      Cu     2.9  ug/l
      Mn     7.900001E-03
      Si     2800 as SiO2
      Zn     5.8  ug/l
      Alkalinity         142.5  as HCO3
SOLUTION 18 4LKaterina-1
      units mg/L
      pH    7.0 #value missing
      pe    4
      temp   14.1
      Ca     11.9
      Mg     25.5
      Na     32.8
      K      1.24
      Cl     31.208
      S(6)   5.08 as SO4
      F      .093
      Ba     .0026
      Cu     2.5  ug/l
      Mn     .014
      Si     3000 as SiO2
      Zn     6.9  ug/l
      Alkalinity         191.1  as HCO3
SOLUTION 19 4LLinda-1
      units mg/L
      pH    7.0 #value missing
      pe    4
      temp   18.5
      Ca     18.3
      Mg     28.2
      Na     49
      K      1.96
      Cl     95.703
      S(6)   16.15 as SO4
      F      .113
      Ba     .0021
      Cu     4.4  ug/l
      Mn     .0156
      Si     4700 as SiO2
      Zn     11.1 ug/l
      Alkalinity         205.5281  as HCO3
SOLUTION 20 4LLudmila-1
      units mg/L
      pH    7.0 #value missing
      pe    4
      temp   15.3
      Ca     4.9
      Mg     5.75
      Na     23.3
      K      .68
      Cl     22.038
      S(6)   1.84 as SO4
      F      .041
      Ba     .0011
      Cu     3.1  ug/l
      Mn     .0077
      Si     3600 as SiO2
      Zn     9.9  ug/l
      Alkalinity         65.35999  as HCO3
```

```
SOLUTION 6 4LMaría-1
      units mg/L
      pH    7.0 #value missing
      pe    4
      temp   15.2
      Ca     5.6
      Mg     7.26
      Na     23.2
      K      1.02
      Cl     34.282
      S(6)   4.45 as SO4
      Ba     .0021
      Cu     2.7  ug/l
      Mn     .0117
      Si     3800 as SiO2
      Zn     12.9 ug/l
      Alkalinity         50.41  as HCO3
SOLUTION 21 4LMarta-1
      units mg/L
      pH    7.0 #value missing
      pe    4
      temp   18.5
      Ca     9.3
      Mg     14.7
      Na     36.1
      K      1.41
      Cl     53.948
      S(6)   7.59 as SO4
      F      .089
      Ba     .0013
      Cu     1.9  ug/l
      Mn     .0058
      Si     4400 as SiO2
      Zn     4    ug/l
      Alkalinity         129.5844  as HCO3
SOLUTION 7 4LMartina-1
      units mg/L
      pH    7.0 #value missing
      pe    4
      temp   16.6
      Ca     38.4
      Mg     90.5
      Na     190.017
      K      4.18
      Cl     521.273
      S(6)   44.85 as SO4
      Ba     .0044
      Cu     4.2  ug/l
      Mn     .0181
      Si     9700 as SiO2
      Zn     14   ug/l
      Alkalinity         121.39  as HCO3
SOLUTION 8 4LNatasha-1
      units mg/L
      pH    7.0 #value missing
      pe    4
      temp   1.4
      Ca     14.2
      Mg     49.5
      Na     79.1
      K      2.88
      Cl     204.433
      S(6)   7.05 as SO4
      Ba     .0015
      Cu     4    ug/l
      Mn     .0217
      Si     3800 as SiO2
```

```
      Zn     14.7 ug/l
      Alkalinity         155.4419  as HCO3
SOLUTION 22 4LNora-1
      units mg/L
      pH    7.0 #value missing
      pe    4
      temp   15.9
      Ca     9.6
      Mg     22.2
      Na     35
      K      1.31
      Cl     59.985
      S(6)   7.77 as SO4
      F      .098
      Ba     .0015
      Cu     2    ug/l
      Mn     .0085
      Si     3000 as SiO2
      Zn     2.9  ug/l
      Alkalinity         164.3656  as HCO3
SOLUTION 34 4LNorma-1
      units mg/L
      pH    7.0 #value missing
      pe    4
      temp   6.8
      Ca     5.9
      Mg     8.83
      Na     24.2
      K      .75
      Cl     36.946
      S(6)   4.48 as SO4
      F      .097
      Ba     .0009
      Cu     2.2  ug/l
      Mn     .0118
      Si     2600 as SiO2
      Zn     11.1 ug/l
      Alkalinity         57.23  as HCO3
SOLUTION 23 4LPaula-1
      units mg/L
      pH    7.0 #value missing
      pe    4
      temp   17.2
      Ca     12.7
      Mg     16.4
      Na     37.1
      K      1.8
      Cl     45.262
      S(6)   7.27 as SO4
      Ba     .0023
      Cu     4.1  ug/l
      Mn     .0236
      Si     4400 as SiO2
      Zn     91.5 ug/l
      Alkalinity         205.5093  as HCO3
SOLUTION 24 4LSandra-1
      units mg/L
      pH    7.0 #value missing
      pe    4
      temp   12
      Ca     4.1
      Mg     5.51
      Na     24.4
      K      .78
      Cl     23.962
      S(6)   3.29 as SO4
      F      .099
```

```
      Ba      .002
      Cu      2    ug/l
      Mn      .0084
      Si      3500 as SiO2
      Zn      8.5  ug/l
      Alkalinity         59.48  as HCO3
SOLUTION 25 4LSara-1
      units mg/L
      pH    7.0 #value missing
      pe    4
      temp   17.6
      Ca      12.5
      Mg      25.9
      Na      43.6
      K       1.71
      Cl      88.728
      S(6)   10.65 as SO4
      F       .135
      Ba      .0046
      Cu      4    ug/l
      Mn      .0177
      Si      4100 as SiO2
      Zn      13.8 ug/l
      Alkalinity         156.575  as HCO3
SOLUTION 26 4LSilvia-1
      units mg/L
      pH    7.0 #value missing
      pe    4
      temp   13
      Ca      17.8
      Mg      29.4
      Na      46.4
      K       1.77
      Cl      64.02
      S(6)   13.41 as SO4
      F       .043
      Ba      .0003
      Cu      2.5  ug/l
      Mn      .0053
      Si      2900 as SiO2
      Zn      4.9  ug/l
      Alkalinity         197.68  as HCO3
SOLUTION 10 4LSoledad-1
      units mg/L
      pH    7.0 #value missing
      pe    4
      temp   15.7
      Ca      3.2
      Mg      4.92
      Na      17.5
      K       .75
      Cl      27.484
      S(6)   4.19 as SO4
      F       .079
      Ba      .0018
      Cu      2.7  ug/l
      Mn      .0119
      Si      2700 as SiO2
      Zn      10.9 ug/l
      Alkalinity         28.26  as HCO3
SOLUTION 27 4LSusan-1
      units mg/L
      pH    7.0 #value missing
      pe    4
      temp   11.3
      Ca      4.1
      Mg      7.65
```

```
      Na     13.6
      K      .82
      Cl     9.08
      S(6)   1.7 as SO4
      F      .087
      Ba     .0032
      Cu     2.7  ug/l
      Mn     .0112
      Si     1800 as SiO2
      Zn     11.5 ug/l
      Alkalinity         69.18  as HCO3
SOLUTION 11 4LTamara-1
      units mg/L
      pH    7.0 #value missing
      pe    4
      temp   8.6
      Ca     6.2
      Mg     9.02
      Na     29.1
      K      .76
      Cl     52.532
      S(6)   7.79 as SO4
      Ba     .0008
      Cu     1.7  ug/l
      Mn     .0074
      Si     2400 as SiO2
      Zn     6.3  ug/l
      Alkalinity         41.08  as HCO3
SOLUTION 30 4LTatana-1
      units mg/L
      pH    7.0 #value missing
      pe    4
      temp   16
      Ca     32.4
      Mg     44.8
      Na     145
      K      2.63
      Cl     229.76
      S(6)   14.05 as SO4
      Ba     .0016
      Cu     5.6  ug/l
      Mn     8.800001E-03
      Si     12600 as SiO2
      Zn     9    ug/l
      Alkalinity         334.095  as HCO3
SOLUTION 32 4LTrinidad-1
      units mg/L
      pH    7.0 #value missing
      pe    4
      temp   13
      Ca     20
      Mg     87.6
      Na     154
      K      3.03
      Cl     83.077
      S(6)   1.84 as SO4
      F      .11
      Ba     .0008
      Cu     3.5  ug/l
      Mn     8.300001E-03
      Si     2700 as SiO2
      Zn     15.2 ug/l
      Alkalinity         812.7361  as HCO3
SOLUTION 28 4LValentina-1
      units mg/L
      pH    7.0 #value missing
      pe    4
```

```
            temp   19.1
            Ca     12.6
            Mg     18.4
            Na     43.5
            K      1.89
            Cl     39.526
            S(6)   6.32 as SO4
            F      .125
            Ba     .0022
            Cu     2.3  ug/l
            Mn     .0128
            Si     4700 as SiO2
            Zn     4.4  ug/l
            Alkalinity         214.0256  as HCO3
      end
-----
TITLE
-----

 <Enter step description>

-------------------------------------------
Beginning of initial solution calculations.
-------------------------------------------

Initial solution 1.     4LAdriana-1

-----------------------------Solution composition------------------------------

      Elements           Molality       Moles

      Alkalinity       2.562e-003  2.562e-003
      Ba               3.645e-009  3.645e-009
      Ca               3.072e-004  3.072e-004
      Cl               1.286e-002  1.286e-002
      Cu               6.144e-008  6.144e-008
      K                9.268e-005  9.268e-005
      Mg               3.908e-003  3.908e-003
      Mn               3.098e-008  3.098e-008
      Na               7.768e-003  7.768e-003
      S(6)             3.901e-004  3.901e-004
      Si               3.332e-003  3.332e-003
      Zn               7.657e-009  7.657e-009

----------------------------Description of solution----------------------------

                                       pH  =   7.000
                                       pe  =   4.000
                        Activity of water  =   0.999
                           Ionic strength  =  2.041e-002
                       Mass of water (kg)  =  1.000e+000
                    Total carbon (mol/kg)  =  3.178e-003
                       Total CO2 (mol/kg)  =  3.178e-003
                      Temperature (deg C)  =  10.500
                  Electrical balance (eq)  =  9.283e-005
 Percent error, 100*(Cat-|An|)/(Cat+|An|)  =   0.29
                               Iterations  =   9
                                  Total H  = 1.110283e+002
                                  Total O  = 5.553002e+001

----------------------------Distribution of species----------------------------

                                                    Log       Log         Log
   Species                   Molality    Activity  Molality  Activity     Gamma

   H+                      1.122e-007  1.000e-007    -6.950    -7.000    -0.050
   OH-                     3.518e-008  3.056e-008    -7.454    -7.515    -0.061
```

	H2O	5.551e+001	9.995e-001	1.744	-0.000	0.000
Ba	3.645e-009					
	Ba+2	3.429e-009	2.016e-009	-8.465	-8.696	-0.231
	BaSO4	1.844e-010	1.853e-010	-9.734	-9.732	0.002
	BaHCO3+	3.050e-011	2.661e-011	-10.516	-10.575	-0.059
	BaCO3	5.556e-013	5.582e-013	-12.255	-12.253	0.002
	BaOH+	7.824e-016	6.827e-016	-15.107	-15.166	-0.059
C(4)	3.178e-003					
	HCO3-	2.478e-003	2.174e-003	-2.606	-2.663	-0.057
	CO2	6.222e-004	6.252e-004	-3.206	-3.204	0.002
	MgHCO3+	6.332e-005	5.525e-005	-4.198	-4.258	-0.059
	NaHCO3	8.247e-006	8.285e-006	-5.084	-5.082	0.002
	CaHCO3+	4.113e-006	3.608e-006	-5.386	-5.443	-0.057
	MgCO3	1.234e-006	1.240e-006	-5.909	-5.907	0.002
	CO3-2	1.210e-006	7.172e-007	-5.917	-6.144	-0.227
	CaCO3	1.724e-007	1.732e-007	-6.763	-6.761	0.002
	NaCO3-	4.809e-008	4.196e-008	-7.318	-7.377	-0.059
	MnHCO3+	3.398e-009	2.965e-009	-8.469	-8.528	-0.059
	ZnHCO3+	1.081e-009	9.431e-010	-8.966	-9.025	-0.059
	MnCO3	8.678e-010	8.719e-010	-9.062	-9.060	0.002
	ZnCO3	4.908e-010	4.931e-010	-9.309	-9.307	0.002
	BaHCO3+	3.050e-011	2.661e-011	-10.516	-10.575	-0.059
	Zn(CO3)2-2	1.304e-011	7.561e-012	-10.885	-11.121	-0.237
	BaCO3	5.556e-013	5.582e-013	-12.255	-12.253	0.002
Ca	3.072e-004					
	Ca+2	2.974e-004	1.762e-004	-3.527	-3.754	-0.227
	CaSO4	5.564e-006	5.590e-006	-5.255	-5.253	0.002
	CaHCO3+	4.113e-006	3.608e-006	-5.386	-5.443	-0.057
	CaCO3	1.724e-007	1.732e-007	-6.763	-6.761	0.002
	CaOH+	3.349e-010	2.922e-010	-9.475	-9.534	-0.059
	CaHSO4+	3.217e-012	2.807e-012	-11.492	-11.552	-0.059
Cl	1.286e-002					
	Cl-	1.286e-002	1.118e-002	-1.891	-1.952	-0.061
	MnCl+	7.985e-010	6.968e-010	-9.098	-9.157	-0.059
	ZnCl+	6.066e-011	5.293e-011	-10.217	-10.276	-0.059
	MnCl2	3.383e-012	3.399e-012	-11.471	-11.469	0.002
	ZnCl2	5.798e-013	5.826e-013	-12.237	-12.235	0.002
	MnCl3-	1.199e-014	1.046e-014	-13.921	-13.980	-0.059
	ZnCl3-	7.640e-015	6.666e-015	-14.117	-14.176	-0.059
	ZnCl4-2	5.707e-017	3.309e-017	-16.244	-16.480	-0.237
Cu(1)	8.205e-010					
	Cu+	8.205e-010	7.086e-010	-9.086	-9.150	-0.064
Cu(2)	6.062e-008					
	Cu(OH)2	3.234e-008	3.249e-008	-7.490	-7.488	0.002
	Cu+2	2.598e-008	1.557e-008	-7.585	-7.808	-0.222
	CuOH+	1.786e-009	1.556e-009	-8.748	-8.808	-0.060
	CuSO4	5.221e-010	5.246e-010	-9.282	-9.280	0.002
	Cu(OH)3-	2.242e-014	1.957e-014	-13.649	-13.708	-0.059
	Cu(OH)4-2	6.730e-020	3.902e-020	-19.172	-19.409	-0.237
H(0)	1.640e-025					
	H2	8.201e-026	8.240e-026	-25.086	-25.084	0.002
K	9.268e-005					
	K+	9.259e-005	8.049e-005	-4.033	-4.094	-0.061
	KSO4-	9.109e-008	7.949e-008	-7.041	-7.100	-0.059
	KOH	2.776e-012	2.789e-012	-11.557	-11.555	0.002
Mg	3.908e-003					
	Mg+2	3.778e-003	2.264e-003	-2.423	-2.645	-0.222
	MgSO4	6.541e-005	6.572e-005	-4.184	-4.182	0.002
	MgHCO3+	6.332e-005	5.525e-005	-4.198	-4.258	-0.059
	MgCO3	1.234e-006	1.240e-006	-5.909	-5.907	0.002
	MgOH+	2.377e-008	2.074e-008	-7.624	-7.683	-0.059
Mn(2)	3.098e-008					
	Mn+2	2.554e-008	1.531e-008	-7.593	-7.815	-0.222
	MnHCO3+	3.398e-009	2.965e-009	-8.469	-8.528	-0.059
	MnCO3	8.678e-010	8.719e-010	-9.062	-9.060	0.002
	MnCl+	7.985e-010	6.968e-010	-9.098	-9.157	-0.059
	MnSO4	3.714e-010	3.732e-010	-9.430	-9.428	0.002

```
   MnCl2                  3.383e-012  3.399e-012   -11.471   -11.469     0.002
   MnOH+                  1.301e-012  1.135e-012   -11.886   -11.945    -0.059
   MnCl3-                 1.199e-014  1.046e-014   -13.921   -13.980    -0.059
Mn(3)            1.741e-030
   Mn+3                   1.741e-030  5.107e-031   -29.759   -30.292    -0.533
Na               7.768e-003
   Na+                    7.753e-003  6.777e-003    -2.111    -2.169    -0.058
   NaHCO3                 8.247e-006  8.285e-006    -5.084    -5.082     0.002
   NaSO4-                 6.481e-006  5.655e-006    -5.188    -5.248    -0.059
   NaCO3-                 4.809e-008  4.196e-008    -7.318    -7.377    -0.059
   NaOH                   4.454e-010  4.475e-010    -9.351    -9.349     0.002
O(0)             0.000e+000
   O2                     0.000e+000  0.000e+000   -47.133   -47.131     0.002
S(6)             3.901e-004
   SO4-2                  3.125e-004  1.834e-004    -3.505    -3.737    -0.232
   MgSO4                  6.541e-005  6.572e-005    -4.184    -4.182     0.002
   NaSO4-                 6.481e-006  5.655e-006    -5.188    -5.248    -0.059
   CaSO4                  5.564e-006  5.590e-006    -5.255    -5.253     0.002
   KSO4-                  9.109e-008  7.949e-008    -7.041    -7.100    -0.059
   HSO4-                  1.519e-009  1.325e-009    -8.818    -8.878    -0.059
   CuSO4                  5.221e-010  5.246e-010    -9.282    -9.280     0.002
   MnSO4                  3.714e-010  3.732e-010    -9.430    -9.428     0.002
   BaSO4                  1.844e-010  1.853e-010    -9.734    -9.732     0.002
   ZnSO4                  1.311e-010  1.317e-010    -9.882    -9.880     0.002
   CaHSO4+                3.217e-012  2.807e-012   -11.492   -11.552    -0.059
   Zn(SO4)2-2             3.808e-013  2.208e-013   -12.419   -12.656    -0.237
Si               3.332e-003
   H4SiO4                 3.329e-003  3.345e-003    -2.478    -2.476     0.002
   H3SiO4-                3.243e-006  2.829e-006    -5.489    -5.548    -0.059
   H2SiO4-2               1.185e-012  6.872e-013   -11.926   -12.163    -0.237
Zn               7.657e-009
   Zn+2                   5.862e-009  3.446e-009    -8.232    -8.463    -0.231
   ZnHCO3+                1.081e-009  9.431e-010    -8.966    -9.025    -0.059
   ZnCO3                  4.908e-010  4.931e-010    -9.309    -9.307     0.002
   ZnSO4                  1.311e-010  1.317e-010    -9.882    -9.880     0.002
   ZnCl+                  6.066e-011  5.293e-011   -10.217   -10.276    -0.059
   ZnOH+                  1.362e-011  1.188e-011   -10.866   -10.925    -0.059
   Zn(CO3)2-2             1.304e-011  7.561e-012   -10.885   -11.121    -0.237
   Zn(OH)2                4.313e-012  4.334e-012   -11.365   -11.363     0.002
   ZnCl2                  5.798e-013  5.826e-013   -12.237   -12.235     0.002
   Zn(SO4)2-2             3.808e-013  2.208e-013   -12.419   -12.656    -0.237
   ZnCl3-                 7.640e-015  6.666e-015   -14.117   -14.176    -0.059
   Zn(OH)3-               1.570e-016  1.370e-016   -15.804   -15.863    -0.059
   ZnCl4-2                5.707e-017  3.309e-017   -16.244   -16.480    -0.237
   Zn(OH)4-2              3.742e-022  2.170e-022   -21.427   -21.664    -0.237

------------------------------Saturation indices-------------------------------

      Phase               SI  log IAP  log KT

      Anhydrite        -3.16   -7.49   -4.34  CaSO4
      Aragonite        -1.64   -9.90   -8.26  CaCO3
      Barite           -2.20  -12.43  -10.24  BaSO4
      Calcite          -1.49   -9.90   -8.41  CaCO3
      Chalcedony        1.25   -2.48   -3.73  SiO2
      Chrysotile       -4.97   29.11   34.09  Mg3Si2O5(OH)4
      CO2(g)           -1.93   -3.20   -1.28  CO2
      Dolomite         -1.95  -18.69  -16.74  CaMg(CO3)2
      Gypsum           -2.90   -7.49   -4.59  CaSO4:2H2O
      H2(g)           -22.00  -25.08   -3.08  H2
      H2O(g)           -1.90   -0.00    1.90  H2O
      Halite           -5.67   -4.12    1.55  NaCl
      Hausmannite     -24.25   40.55   64.80  Mn3O4
      Manganite        -8.16   17.18   25.34  MnOOH
      O2(g)           -44.24  -47.13   -2.89  O2
      Pyrochroite      -9.02    6.18   15.20  Mn(OH)2
      Pyrolusite      -15.64   28.18   43.82  MnO2
```

Quartz	1.73	-2.48	-4.20	SiO2
Rhodochrosite	-2.88	-13.96	-11.08	MnCO3
Sepiolite	-0.88	15.28	16.16	Mg2Si3O7.5OH:3H2O
Sepiolite(d)	-3.38	15.28	18.66	Mg2Si3O7.5OH:3H2O
SiO2(a)	0.36	-2.48	-2.84	SiO2
Smithsonite	-4.77	-14.61	-9.84	ZnCO3
Talc	1.03	24.16	23.14	Mg3Si4O10(OH)2
Willemite	-7.98	8.60	16.58	Zn2SiO4
Witherite	-6.21	-14.84	-8.63	BaCO3
Zn(OH)2(e)	-5.96	5.54	11.50	Zn(OH)2

Initial solution 2.   4LAdru-1

-----------------------------Solution composition------------------------------

Elements	Molality	Moles
Alkalinity	7.832e-004	7.832e-004
Ba	1.462e-009	1.462e-009
Ca	1.628e-004	1.628e-004
Cl	1.273e-003	1.273e-003
Cu	3.159e-008	3.159e-008
F	2.959e-006	2.959e-006
K	1.345e-004	1.345e-004
Mg	2.948e-004	2.948e-004
Mn	6.577e-008	6.577e-008
Na	1.183e-003	1.183e-003
S(6)	6.656e-005	6.656e-005
Si	6.014e-002	6.014e-002
Zn	2.488e-007	2.488e-007

----------------------------Description of solution----------------------------

pH	=	7.000
pe	=	4.000
Activity of water	=	0.999
Ionic strength	=	2.718e-003
Mass of water (kg)	=	1.000e+000
Total carbon (mol/kg)	=	9.117e-004
Total CO2 (mol/kg)	=	9.117e-004
Temperature (deg C)	=	12.600
Electrical balance (eq)	=	4.100e-005
Percent error, 100*(Cat-|An|)/(Cat+|An|)	=	0.93
Iterations	=	9
Total H	=	1.112537e+002
Total O	=	5.574959e+001

----------------------------Distribution of species----------------------------

Species		Molality	Activity	Log Molality	Log Activity	Log Gamma
H+		1.053e-007	1.000e-007	-6.977	-7.000	-0.023
OH-		3.883e-008	3.670e-008	-7.411	-7.435	-0.025
H2O		5.551e+001	9.989e-001	1.744	-0.000	0.000
Ba	1.462e-009					
Ba+2		1.427e-009	1.144e-009	-8.845	-8.941	-0.096
BaSO4		2.899e-011	2.901e-011	-10.538	-10.538	0.000
BaHCO3+		5.355e-012	5.063e-012	-11.271	-11.296	-0.024
BaCO3		1.099e-013	1.100e-013	-12.959	-12.959	0.000
BaOH+		4.096e-016	3.873e-016	-15.388	-15.412	-0.024
C(4)	9.117e-004					
HCO3-		7.205e-004	6.820e-004	-3.142	-3.166	-0.024
CO2		1.876e-004	1.877e-004	-3.727	-3.727	0.000
MgHCO3+		1.899e-006	1.796e-006	-5.721	-5.746	-0.024
CaHCO3+		9.253e-007	8.758e-007	-6.034	-6.058	-0.024
NaHCO3		4.287e-007	4.290e-007	-6.368	-6.368	0.000

CO3-2	2.972e-007	2.386e-007	-6.527	-6.622	-0.095
MgCO3	4.398e-008	4.401e-008	-7.357	-7.356	0.000
CaCO3	4.288e-008	4.290e-008	-7.368	-7.368	0.000
ZnHCO3+	1.609e-008	1.521e-008	-7.794	-7.818	-0.024
ZnCO3	8.428e-009	8.433e-009	-8.074	-8.074	0.000
MnHCO3+	3.159e-009	2.987e-009	-8.500	-8.525	-0.024
NaCO3-	2.737e-009	2.588e-009	-8.563	-8.587	-0.024
MnCO3	9.307e-010	9.313e-010	-9.031	-9.031	0.000
Zn(CO3)2-2	5.383e-011	4.302e-011	-10.269	-10.366	-0.097
BaHCO3+	5.355e-012	5.063e-012	-11.271	-11.296	-0.024
BaCO3	1.099e-013	1.100e-013	-12.959	-12.959	0.000
Ca	1.628e-004				
Ca+2	1.607e-004	1.289e-004	-3.794	-3.890	-0.096
CaSO4	1.152e-006	1.153e-006	-5.938	-5.938	0.000
CaHCO3+	9.253e-007	8.758e-007	-6.034	-6.058	-0.024
CaCO3	4.288e-008	4.290e-008	-7.368	-7.368	0.000
CaF+	2.422e-009	2.290e-009	-8.616	-8.640	-0.024
CaOH+	2.261e-010	2.138e-010	-9.646	-9.670	-0.024
CaHSO4+	6.241e-013	5.901e-013	-12.205	-12.229	-0.024
Cl	1.273e-003				
Cl-	1.273e-003	1.203e-003	-2.895	-2.920	-0.025
ZnCl+	3.430e-010	3.243e-010	-9.465	-9.489	-0.024
MnCl+	2.548e-010	2.409e-010	-9.594	-9.618	-0.024
ZnCl2	3.877e-013	3.879e-013	-12.412	-12.411	0.000
MnCl2	1.264e-013	1.265e-013	-12.898	-12.898	0.000
ZnCl3-	5.125e-016	4.846e-016	-15.290	-15.315	-0.024
MnCl3-	4.435e-017	4.193e-017	-16.353	-16.377	-0.024
ZnCl4-2	3.301e-019	2.638e-019	-18.481	-18.579	-0.097
Cu(1)	4.458e-010				
Cu+	4.458e-010	4.209e-010	-9.351	-9.376	-0.025
Cu(2)	3.115e-008				
Cu(OH)2	1.885e-008	1.887e-008	-7.725	-7.724	0.000
Cu+2	1.125e-008	9.050e-009	-7.949	-8.043	-0.094
CuOH+	9.562e-010	9.040e-010	-9.019	-9.044	-0.024
CuSO4	8.541e-011	8.547e-011	-10.068	-10.068	0.000
Cu(OH)3-	1.201e-014	1.136e-014	-13.920	-13.945	-0.024
Cu(OH)4-2	2.832e-020	2.263e-020	-19.548	-19.645	-0.097
F	2.959e-006				
F-	2.919e-006	2.758e-006	-5.535	-5.559	-0.025
MgF+	3.565e-008	3.371e-008	-7.448	-7.472	-0.024
CaF+	2.422e-009	2.290e-009	-8.616	-8.640	-0.024
NaF	1.774e-009	1.775e-009	-8.751	-8.751	0.000
HF	3.326e-010	3.328e-010	-9.478	-9.478	0.000
MnF+	9.916e-013	9.375e-013	-12.004	-12.028	-0.024
HF2-	3.317e-015	3.137e-015	-14.479	-14.504	-0.024
SiF6-2	1.656e-032	1.323e-032	-31.781	-31.878	-0.097
H(0)	1.610e-025				
H2	8.048e-026	8.053e-026	-25.094	-25.094	0.000
K	1.345e-004				
K+	1.345e-004	1.271e-004	-3.871	-3.896	-0.025
KSO4-	3.812e-008	3.604e-008	-7.419	-7.443	-0.024
KOH	4.399e-012	4.402e-012	-11.357	-11.356	0.000
Mg	2.948e-004				
Mg+2	2.908e-004	2.338e-004	-3.536	-3.631	-0.095
MgSO4	1.986e-006	1.987e-006	-5.702	-5.702	0.000
MgHCO3+	1.899e-006	1.796e-006	-5.721	-5.746	-0.024
MgCO3	4.398e-008	4.401e-008	-7.357	-7.356	0.000
MgF+	3.565e-008	3.371e-008	-7.448	-7.472	-0.024
MgOH+	2.789e-009	2.637e-009	-8.555	-8.579	-0.024
Mn(2)	6.577e-008				
Mn+2	6.108e-008	4.914e-008	-7.214	-7.309	-0.094
MnHCO3+	3.159e-009	2.987e-009	-8.500	-8.525	-0.024
MnCO3	9.307e-010	9.313e-010	-9.031	-9.031	0.000
MnSO4	3.451e-010	3.453e-010	-9.462	-9.462	0.000
MnCl+	2.548e-010	2.409e-010	-9.594	-9.618	-0.024
MnOH+	4.648e-012	4.394e-012	-11.333	-11.357	-0.024
MnF+	9.916e-013	9.375e-013	-12.004	-12.028	-0.024

```
   MnCl2              1.264e-013  1.265e-013   -12.898   -12.898     0.000
   MnCl3-             4.435e-017  4.193e-017   -16.353   -16.377    -0.024
Mn(3)            3.800e-030
   Mn+3               3.800e-030  2.295e-030   -29.420   -29.639    -0.219
Na               1.183e-003
   Na+                1.183e-003  1.118e-003    -2.927    -2.951    -0.024
   NaHCO3             4.287e-007  4.290e-007    -6.368    -6.368     0.000
   NaSO4-             2.763e-007  2.612e-007    -6.559    -6.583    -0.024
   NaCO3-             2.737e-009  2.588e-009    -8.563    -8.587    -0.024
   NaF                1.774e-009  1.775e-009    -8.751    -8.751     0.000
   NaOH               7.377e-011  7.382e-011   -10.132   -10.132     0.000
O(0)             0.000e+000
   O2                 0.000e+000  0.000e+000   -46.369   -46.368     0.000
S(6)             6.656e-005
   SO4-2              6.310e-005  5.058e-005    -4.200    -4.296    -0.096
   MgSO4              1.986e-006  1.987e-006    -5.702    -5.702     0.000
   CaSO4              1.152e-006  1.153e-006    -5.938    -5.938     0.000
   NaSO4-             2.763e-007  2.612e-007    -6.559    -6.583    -0.024
   KSO4-              3.812e-008  3.604e-008    -7.419    -7.443    -0.024
   ZnSO4              1.900e-009  1.901e-009    -8.721    -8.721     0.000
   HSO4-              4.026e-010  3.806e-010    -9.395    -9.419    -0.024
   MnSO4              3.451e-010  3.453e-010    -9.462    -9.462     0.000
   CuSO4              8.541e-011  8.547e-011   -10.068   -10.068     0.000
   BaSO4              2.899e-011  2.901e-011   -10.538   -10.538     0.000
   Zn(SO4)2-2         1.080e-012  8.635e-013   -11.966   -12.064    -0.097
   CaHSO4+            6.241e-013  5.901e-013   -12.205   -12.229    -0.024
Si               6.014e-002
   H4SiO4             6.008e-002  6.012e-002    -1.221    -1.221     0.000
   H3SiO4-            5.869e-005  5.549e-005    -4.231    -4.256    -0.024
   H2SiO4-2           1.980e-011  1.582e-011   -10.703   -10.801    -0.097
   SiF6-2             1.656e-032  1.323e-032   -31.781   -31.878    -0.097
Zn               2.488e-007
   Zn+2               2.209e-007  1.771e-007    -6.656    -6.752    -0.096
   ZnHCO3+            1.609e-008  1.521e-008    -7.794    -7.818    -0.024
   ZnCO3              8.428e-009  8.433e-009    -8.074    -8.074     0.000
   ZnSO4              1.900e-009  1.901e-009    -8.721    -8.721     0.000
   ZnOH+              7.691e-010  7.271e-010    -9.114    -9.138    -0.024
   ZnCl+              3.430e-010  3.243e-010    -9.465    -9.489    -0.024
   Zn(OH)2            2.224e-010  2.225e-010    -9.653    -9.653     0.000
   Zn(CO3)2-2         5.383e-011  4.302e-011   -10.269   -10.366    -0.097
   Zn(SO4)2-2         1.080e-012  8.635e-013   -11.966   -12.064    -0.097
   ZnCl2              3.877e-013  3.879e-013   -12.412   -12.411     0.000
   Zn(OH)3-           7.434e-015  7.029e-015   -14.129   -14.153    -0.024
   ZnCl3-             5.125e-016  4.846e-016   -15.290   -15.315    -0.024
   ZnCl4-2            3.301e-019  2.638e-019   -18.481   -18.579    -0.097
   Zn(OH)4-2          1.392e-020  1.113e-020   -19.856   -19.954    -0.097

------------------------------Saturation indices-------------------------------

      Phase                SI log IAP  log KT

      Anhydrite         -3.85   -8.19   -4.33  CaSO4
      Aragonite         -2.24  -10.51   -8.27  CaCO3
      Barite            -3.05  -13.24  -10.19  BaSO4
      Calcite           -2.09  -10.51   -8.42  CaCO3
      Chalcedony         2.48   -1.22   -3.70  SiO2
      Chrysotile        -5.14   28.66   33.80  Mg3Si2O5(OH)4
      CO2(g)            -2.42   -3.73   -1.31  CO2
      Dolomite          -3.98  -20.77  -16.79  CaMg(CO3)2
      Fluorite          -4.25  -15.01  -10.76  CaF2
      Gypsum            -3.60   -8.19   -4.59  CaSO4:2H2O
      H2(g)            -22.00  -25.09   -3.09  H2
      H2O(g)            -1.85   -0.00    1.84  H2O
      Halite            -7.42   -5.87    1.55  NaCl
      Hausmannite      -22.16   42.07   64.23  Mn3O4
      Manganite         -7.65   17.69   25.34  MnOOH
      O2(g)            -43.47  -46.37   -2.90  O2
```

Pyrochroite	-8.51	6.69	15.20	Mn(OH)2
Pyrolusite	-14.76	28.69	43.45	MnO2
Quartz	2.95	-1.22	-4.17	SiO2
Rhodochrosite	-2.85	-13.93	-11.08	MnCO3
Sepiolite	0.97	17.08	16.10	Mg2Si3O7.5OH:3H2O
Sepiolite(d)	-1.58	17.08	18.66	Mg2Si3O7.5OH:3H2O
SiO2(a)	1.60	-1.22	-2.82	SiO2
Smithsonite	-3.51	-13.37	-9.86	ZnCO3
Talc	3.35	26.22	22.87	Mg3Si4O10(OH)2
Willemite	-3.12	13.28	16.39	Zn2SiO4
Witherite	-6.95	-15.56	-8.61	BaCO3
Zn(OH)2(e)	-4.25	7.25	11.50	Zn(OH)2

Initial solution 3.   4LAndrea-1

----------------------------Solution composition----------------------------

Elements	Molality	Moles
Alkalinity	2.577e-003	2.577e-003
Ba	1.463e-009	1.463e-009
Ca	7.444e-004	7.444e-004
Cl	4.321e-003	4.321e-003
Cu	2.529e-008	2.529e-008
F	8.090e-006	8.090e-006
K	6.757e-005	6.757e-005
Mg	1.021e-003	1.021e-003
Mn	1.024e-007	1.024e-007
Na	3.474e-003	3.474e-003
S(6)	7.707e-005	7.707e-005
Si	6.855e-002	6.855e-002
Zn	9.374e-008	9.374e-008

----------------------------Description of solution----------------------------

pH	=	7.000
pe	=	4.000
Activity of water	=	0.999
Ionic strength	=	8.806e-003
Mass of water (kg)	=	1.000e+000
Total carbon (mol/kg)	=	3.212e-003
Total CO2 (mol/kg)	=	3.212e-003
Temperature (deg C)	=	7.700
Electrical balance (eq)	=	1.228e-005
Percent error, 100*(Cat-|An|)/(Cat+|An|)	=	0.09
Iterations	=	9
Total H	=	1.112891e+002
Total O	=	5.578967e+001

----------------------------Distribution of species----------------------------

Species		Molality	Activity	Log Molality	Log Activity	Log Gamma
H+		1.088e-007	1.000e-007	-6.964	-7.000	-0.036
OH-		2.616e-008	2.375e-008	-7.582	-7.624	-0.042
H2O		5.551e+001	9.986e-001	1.744	-0.001	0.000
Ba	1.463e-009					
Ba+2		1.426e-009	9.833e-010	-8.846	-9.007	-0.161
BaSO4		2.293e-011	2.298e-011	-10.640	-10.639	0.001
BaHCO3+		1.361e-011	1.238e-011	-10.866	-10.907	-0.041
BaCO3		2.463e-013	2.468e-013	-12.609	-12.608	0.001
BaOH+		3.659e-016	3.327e-016	-15.437	-15.478	-0.041
C(4)	3.212e-003					
HCO3-		2.482e-003	2.264e-003	-2.605	-2.645	-0.040
CO2		6.937e-004	6.951e-004	-3.159	-3.158	0.001
MgHCO3+		1.932e-005	1.757e-005	-4.714	-4.755	-0.041

CaHCO3+		1.087e-005	9.916e-006	-4.964	-5.004	-0.040
NaHCO3		4.012e-006	4.020e-006	-5.397	-5.396	0.001
CO3-2		9.933e-007	6.875e-007	-6.003	-6.163	-0.160
CaCO3		4.675e-007	4.685e-007	-6.330	-6.329	0.001
MgCO3		3.478e-007	3.485e-007	-6.459	-6.458	0.001
NaCO3-		1.761e-008	1.601e-008	-7.754	-7.796	-0.041
ZnHCO3+		1.528e-008	1.390e-008	-7.816	-7.857	-0.041
MnHCO3+		1.308e-008	1.190e-008	-7.883	-7.924	-0.041
ZnCO3		6.675e-009	6.689e-009	-8.176	-8.175	0.001
MnCO3		3.214e-009	3.221e-009	-8.493	-8.492	0.001
Zn(CO3)2-2		1.437e-010	9.832e-011	-9.842	-10.007	-0.165
BaHCO3+		1.361e-011	1.238e-011	-10.866	-10.907	-0.041
BaCO3		2.463e-013	2.468e-013	-12.609	-12.608	0.001
Ca	7.444e-004					
Ca+2		7.291e-004	5.044e-004	-3.137	-3.297	-0.160
CaHCO3+		1.087e-005	9.916e-006	-4.964	-5.004	-0.040
CaSO4		3.945e-006	3.953e-006	-5.404	-5.403	0.001
CaCO3		4.675e-007	4.685e-007	-6.330	-6.329	0.001
CaF+		2.228e-008	2.026e-008	-7.652	-7.693	-0.041
CaOH+		9.192e-010	8.359e-010	-9.037	-9.078	-0.041
CaHSO4+		2.133e-012	1.940e-012	-11.671	-11.712	-0.041
Cl	4.321e-003					
Cl-		4.321e-003	3.923e-003	-2.364	-2.406	-0.042
MnCl+		1.036e-009	9.426e-010	-8.984	-9.026	-0.041
ZnCl+		2.519e-010	2.291e-010	-9.599	-9.640	-0.041
MnCl2		1.611e-012	1.614e-012	-11.793	-11.792	0.001
ZnCl2		8.724e-013	8.742e-013	-12.059	-12.058	0.001
ZnCl3-		3.790e-015	3.447e-015	-14.421	-14.463	-0.041
MnCl3-		1.918e-015	1.744e-015	-14.717	-14.758	-0.041
ZnCl4-2		8.567e-018	5.860e-018	-17.067	-17.232	-0.165
Cu(1)	3.354e-010					
Cu+		3.354e-010	3.036e-010	-9.474	-9.518	-0.043
Cu(2)	2.496e-008					
Cu(OH)2		1.428e-008	1.431e-008	-7.845	-7.844	0.001
Cu+2		9.866e-009	6.868e-009	-8.006	-8.163	-0.157
CuOH+		7.546e-010	6.858e-010	-9.122	-9.164	-0.041
CuSO4		5.747e-011	5.759e-011	-10.241	-10.240	0.001
Cu(OH)3-		9.468e-015	8.610e-015	-14.024	-14.065	-0.041
Cu(OH)4-2		2.508e-020	1.716e-020	-19.601	-19.766	-0.165
F	8.090e-006					
F-		7.799e-006	7.079e-006	-5.108	-5.150	-0.042
MgF+		2.555e-007	2.323e-007	-6.593	-6.634	-0.041
CaF+		2.228e-008	2.026e-008	-7.652	-7.693	-0.041
NaF		1.284e-008	1.286e-008	-7.891	-7.891	0.001
HF		7.852e-010	7.868e-010	-9.105	-9.104	0.001
MnF+		3.176e-012	2.888e-012	-11.498	-11.539	-0.041
HF2-		1.976e-014	1.797e-014	-13.704	-13.745	-0.041
SiF6-2		1.042e-029	7.128e-030	-28.982	-29.147	-0.165
H(0)	1.697e-025					
H2		8.483e-026	8.500e-026	-25.071	-25.071	0.001
K	6.757e-005					
K+		6.755e-005	6.134e-005	-4.170	-4.212	-0.042
KSO4-		1.604e-008	1.458e-008	-7.795	-7.836	-0.041
KOH		2.120e-012	2.124e-012	-11.674	-11.673	0.001
Mg	1.021e-003					
Mg+2		9.960e-004	6.929e-004	-3.002	-3.159	-0.158
MgHCO3+		1.932e-005	1.757e-005	-4.714	-4.755	-0.041
MgSO4		4.709e-006	4.719e-006	-5.327	-5.326	0.001
MgCO3		3.478e-007	3.485e-007	-6.459	-6.458	0.001
MgF+		2.555e-007	2.323e-007	-6.593	-6.634	-0.041
MgOH+		5.261e-009	4.784e-009	-8.279	-8.320	-0.041
Mn(2)	1.024e-007					
Mn+2		8.471e-008	5.897e-008	-7.072	-7.229	-0.157
MnHCO3+		1.308e-008	1.190e-008	-7.883	-7.924	-0.041
MnCO3		3.214e-009	3.221e-009	-8.493	-8.492	0.001
MnCl+		1.036e-009	9.426e-010	-8.984	-9.026	-0.041
MnSO4		3.437e-010	3.444e-010	-9.464	-9.463	0.001

```
   MnOH+                3.725e-012  3.387e-012   -11.429   -11.470    -0.041
   MnF+                 3.176e-012  2.888e-012   -11.498   -11.539    -0.041
   MnCl2                1.611e-012  1.614e-012   -11.793   -11.792     0.001
   MnCl3-               1.918e-015  1.744e-015   -14.717   -14.758    -0.041
Mn(3)           2.930e-030
   Mn+3                 2.930e-030  1.247e-030   -29.533   -29.904    -0.371
Na              3.474e-003
   Na+                  3.469e-003  3.158e-003    -2.460    -2.501    -0.041
   NaHCO3               4.012e-006  4.020e-006    -5.397    -5.396     0.001
   NaSO4-               7.223e-007  6.568e-007    -6.141    -6.183    -0.041
   NaCO3-               1.761e-008  1.601e-008    -7.754    -7.796    -0.041
   NaF                  1.284e-008  1.286e-008    -7.891    -7.891     0.001
   NaOH                 2.079e-010  2.084e-010    -9.682    -9.681     0.001
O(0)            0.000e+000
   O2                   0.000e+000  0.000e+000   -48.168   -48.167     0.001
S(6)            7.707e-005
   SO4-2                6.768e-005  4.662e-005    -4.170    -4.331    -0.162
   MgSO4                4.709e-006  4.719e-006    -5.327    -5.326     0.001
   CaSO4                3.945e-006  3.953e-006    -5.404    -5.403     0.001
   NaSO4-               7.223e-007  6.568e-007    -6.141    -6.183    -0.041
   KSO4-                1.604e-008  1.458e-008    -7.795    -7.836    -0.041
   ZnSO4                4.617e-010  4.627e-010    -9.336    -9.335     0.001
   HSO4-                3.517e-010  3.199e-010    -9.454    -9.495    -0.041
   MnSO4                3.437e-010  3.444e-010    -9.464    -9.463     0.001
   CuSO4                5.747e-011  5.759e-011   -10.241   -10.240     0.001
   BaSO4                2.293e-011  2.298e-011   -10.640   -10.639     0.001
   CaHSO4+              2.133e-012  1.940e-012   -11.671   -11.712    -0.041
   Zn(SO4)2-2           2.953e-013  2.020e-013   -12.530   -12.695    -0.165
Si              6.855e-002
   H4SiO4               6.849e-002  6.863e-002    -1.164    -1.163     0.001
   H3SiO4-              5.660e-005  5.147e-005    -4.247    -4.288    -0.041
   H2SiO4-2             1.467e-011  1.003e-011   -10.834   -10.999    -0.165
   SiF6-2               1.042e-029  7.128e-030   -28.982   -29.147    -0.165
Zn              9.374e-008
   Zn+2                 7.072e-008  4.876e-008    -7.150    -7.312    -0.161
   ZnHCO3+              1.528e-008  1.390e-008    -7.816    -7.857    -0.041
   ZnCO3                6.675e-009  6.689e-009    -8.176    -8.175     0.001
   ZnSO4                4.617e-010  4.627e-010    -9.336    -9.335     0.001
   ZnCl+                2.519e-010  2.291e-010    -9.599    -9.640    -0.041
   ZnOH+                1.458e-010  1.326e-010    -9.836    -9.878    -0.041
   Zn(CO3)2-2           1.437e-010  9.832e-011    -9.842   -10.007    -0.165
   Zn(OH)2              6.109e-011  6.122e-011   -10.214   -10.213     0.001
   ZnCl2                8.724e-013  8.742e-013   -12.059   -12.058     0.001
   Zn(SO4)2-2           2.953e-013  2.020e-013   -12.530   -12.695    -0.165
   ZnCl3-               3.790e-015  3.447e-015   -14.421   -14.463    -0.041
   Zn(OH)3-             2.126e-015  1.933e-015   -14.673   -14.714    -0.041
   ZnCl4-2              8.567e-018  5.860e-018   -17.067   -17.232    -0.165
   Zn(OH)4-2            4.473e-021  3.060e-021   -20.349   -20.514    -0.165

------------------------------Saturation indices-------------------------------

      Phase               SI log IAP  log KT

      Anhydrite        -3.29   -7.63   -4.34  CaSO4
      Aragonite        -1.21   -9.46   -8.25  CaCO3
      Barite           -3.04  -13.34  -10.30  BaSO4
      Calcite          -1.06   -9.46   -8.40  CaCO3
      Chalcedony        2.60   -1.16   -3.76  SiO2
      Chrysotile       -4.28   30.19   34.47  Mg3Si2O5(OH)4
      CO2(g)           -1.92   -3.16   -1.23  CO2
      Dolomite         -2.12  -18.78  -16.66  CaMg(CO3)2
      Fluorite         -2.77  -13.60  -10.83  CaF2
      Gypsum           -3.03   -7.63   -4.60  CaSO4:2H2O
      H2(g)           -22.00  -25.07   -3.07  H2
      H2O(g)           -1.99   -0.00    1.99  H2O
      Halite           -6.45   -4.91    1.54  NaCl
      Hausmannite     -23.26   42.31   65.57  Mn3O4
```

Manganite	-7.57	17.77	25.34	MnOOH
O2(g)	-45.29	-48.17	-2.88	O2
Pyrochroite	-8.43	6.77	15.20	Mn(OH)2
Pyrolusite	-15.55	28.77	44.32	MnO2
Quartz	3.09	-1.16	-4.25	SiO2
Rhodochrosite	-2.33	-13.39	-11.07	MnCO3
Sepiolite	1.95	18.19	16.24	Mg2Si3O7.5OH:3H2O
Sepiolite(d)	-0.47	18.19	18.66	Mg2Si3O7.5OH:3H2O
SiO2(a)	1.70	-1.16	-2.86	SiO2
Smithsonite	-3.67	-13.47	-9.80	ZnCO3
Talc	4.38	27.87	23.49	Mg3Si4O10(OH)2
Willemite	-4.62	12.21	16.84	Zn2SiO4
Witherite	-6.52	-15.17	-8.65	BaCO3
Zn(OH)2(e)	-4.81	6.69	11.50	Zn(OH)2

Initial solution 4.   4LJoanna-1

-----------------------------Solution composition------------------------------

Elements	Molality	Moles
Alkalinity	6.962e-004	6.962e-004
Ba	1.243e-008	1.243e-008
Ca	1.679e-004	1.679e-004
Cl	1.251e-003	1.251e-003
Cu	4.425e-008	4.425e-008
F	4.388e-006	4.388e-006
K	3.442e-005	3.442e-005
Mg	3.945e-004	3.945e-004
Mn	2.486e-007	2.486e-007
Na	1.009e-003	1.009e-003
S(6)	9.315e-005	9.315e-005
Si	7.021e-002	7.021e-002
Zn	3.273e-007	3.273e-007

----------------------------Description of solution----------------------------

pH	=	7.000
pe	=	4.000
Activity of water	=	0.999
Ionic strength	=	2.780e-003
Mass of water (kg)	=	1.000e+000
Total carbon (mol/kg)	=	7.635e-004
Total CO2 (mol/kg)	=	7.635e-004
Temperature (deg C)	=	16.500
Electrical balance (eq)	=	3.181e-005
Percent error, 100*(Cat-|An|)/(Cat+|An|)	=	0.74
Iterations	=	9
Total H	=	1.112938e+002
Total O	=	5.578956e+001

----------------------------Distribution of species----------------------------

Species		Molality	Activity	Log Molality	Log Activity	Log Gamma
H+		1.054e-007	1.000e-007	-6.977	-7.000	-0.023
OH-		5.404e-008	5.102e-008	-7.267	-7.292	-0.025
H2O		5.551e+001	9.987e-001	1.744	-0.001	0.000
Ba	1.243e-008					
Ba+2		1.205e-008	9.628e-009	-7.919	-8.016	-0.098
BaSO4		3.353e-010	3.356e-010	-9.475	-9.474	0.000
BaHCO3+		4.327e-011	4.087e-011	-10.364	-10.389	-0.025
BaCO3		9.395e-013	9.401e-013	-12.027	-12.027	0.000
BaOH+		3.449e-015	3.258e-015	-14.462	-14.487	-0.025
C(4)	7.635e-004					
HCO3-		6.119e-004	5.787e-004	-3.213	-3.238	-0.024

CO2		1.478e-004	1.479e-004	-3.830	-3.830	0.000
MgHCO3+		2.164e-006	2.044e-006	-5.665	-5.690	-0.025
CaHCO3+		8.830e-007	8.351e-007	-6.054	-6.078	-0.024
NaHCO3		3.100e-007	3.102e-007	-6.509	-6.508	0.000
CO3-2		2.802e-007	2.242e-007	-6.553	-6.649	-0.097
MgCO3		5.836e-008	5.839e-008	-7.234	-7.234	0.000
CaCO3		4.314e-008	4.316e-008	-7.365	-7.365	0.000
ZnHCO3+		1.805e-008	1.705e-008	-7.744	-7.768	-0.025
ZnCO3		1.046e-008	1.047e-008	-7.981	-7.980	0.000
MnHCO3+		1.017e-008	9.602e-009	-7.993	-8.018	-0.025
MnCO3		3.313e-009	3.315e-009	-8.480	-8.480	0.000
NaCO3-		2.709e-009	2.559e-009	-8.567	-8.592	-0.025
Zn(CO3)2-2		6.299e-011	5.016e-011	-10.201	-10.300	-0.099
BaHCO3+		4.327e-011	4.087e-011	-10.364	-10.389	-0.025
BaCO3		9.395e-013	9.401e-013	-12.027	-12.027	0.000
Ca	1.679e-004					
Ca+2		1.653e-004	1.322e-004	-3.782	-3.879	-0.097
CaSO4		1.689e-006	1.690e-006	-5.772	-5.772	0.000
CaHCO3+		8.830e-007	8.351e-007	-6.054	-6.078	-0.024
CaCO3		4.314e-008	4.316e-008	-7.365	-7.365	0.000
CaF+		4.039e-009	3.816e-009	-8.394	-8.418	-0.025
CaOH+		2.319e-010	2.191e-010	-9.635	-9.659	-0.025
CaHSO4+		9.511e-013	8.984e-013	-12.022	-12.047	-0.025
Cl	1.251e-003					
Cl-		1.251e-003	1.181e-003	-2.903	-2.928	-0.025
MnCl+		9.483e-010	8.958e-010	-9.023	-9.048	-0.025
ZnCl+		5.354e-010	5.057e-010	-9.271	-9.296	-0.025
ZnCl2		6.035e-013	6.039e-013	-12.219	-12.219	0.000
MnCl2		4.616e-013	4.619e-013	-12.336	-12.335	0.000
ZnCl3-		8.039e-016	7.594e-016	-15.095	-15.120	-0.025
MnCl3-		1.591e-016	1.502e-016	-15.798	-15.823	-0.025
ZnCl4-2		5.267e-019	4.194e-019	-18.278	-18.377	-0.099
Cu(1)	6.482e-010					
Cu+		6.482e-010	6.114e-010	-9.188	-9.214	-0.025
Cu(2)	4.361e-008					
Cu(OH)2		2.633e-008	2.635e-008	-7.580	-7.579	0.000
Cu+2		1.577e-008	1.264e-008	-7.802	-7.898	-0.096
CuOH+		1.337e-009	1.263e-009	-8.874	-8.899	-0.025
CuSO4		1.689e-010	1.690e-010	-9.772	-9.772	0.000
Cu(OH)3-		1.679e-014	1.586e-014	-13.775	-13.800	-0.025
Cu(OH)4-2		3.968e-020	3.160e-020	-19.401	-19.500	-0.099
F	4.388e-006					
F-		4.306e-006	4.065e-006	-5.366	-5.391	-0.025
MgF+		7.547e-008	7.129e-008	-7.122	-7.147	-0.025
CaF+		4.039e-009	3.816e-009	-8.394	-8.418	-0.025
NaF		2.228e-009	2.229e-009	-8.652	-8.652	0.000
HF		5.242e-010	5.246e-010	-9.280	-9.280	0.000
MnF+		5.542e-012	5.235e-012	-11.256	-11.281	-0.025
HF2-		8.035e-015	7.590e-015	-14.095	-14.120	-0.025
SiF6-2		1.353e-031	1.077e-031	-30.869	-30.968	-0.099
H(0)	1.544e-025					
H2		7.719e-026	7.724e-026	-25.112	-25.112	0.000
K	3.442e-005					
K+		3.440e-005	3.248e-005	-4.463	-4.488	-0.025
KSO4-		1.442e-008	1.362e-008	-7.841	-7.866	-0.025
KOH		1.124e-012	1.125e-012	-11.949	-11.949	0.000
Mg	3.945e-004					
Mg+2		3.882e-004	3.110e-004	-3.411	-3.507	-0.096
MgSO4		4.045e-006	4.047e-006	-5.393	-5.393	0.000
MgHCO3+		2.164e-006	2.044e-006	-5.665	-5.690	-0.025
MgF+		7.547e-008	7.129e-008	-7.122	-7.147	-0.025
MgCO3		5.836e-008	5.839e-008	-7.234	-7.234	0.000
MgOH+		5.419e-009	5.119e-009	-8.266	-8.291	-0.025
Mn(2)	2.486e-007					
Mn+2		2.322e-007	1.862e-007	-6.634	-6.730	-0.096
MnHCO3+		1.017e-008	9.602e-009	-7.993	-8.018	-0.025
MnCO3		3.313e-009	3.315e-009	-8.480	-8.480	0.000

```
   MnSO4                1.947e-009  1.948e-009    -8.711    -8.710     0.000
   MnCl+                9.483e-010  8.958e-010    -9.023    -9.048    -0.025
   MnOH+                2.479e-011  2.342e-011   -10.606   -10.630    -0.025
   MnF+                 5.542e-012  5.235e-012   -11.256   -11.281    -0.025
   MnCl2                4.616e-013  4.619e-013   -12.336   -12.335     0.000
   MnCl3-               1.591e-016  1.502e-016   -15.798   -15.823    -0.025
Mn(3)           2.677e-029
   Mn+3                 2.677e-029  1.603e-029   -28.572   -28.795    -0.223
Na              1.009e-003
   Na+                  1.009e-003  9.531e-004    -2.996    -3.021    -0.025
   NaSO4-               3.326e-007  3.142e-007    -6.478    -6.503    -0.025
   NaHCO3               3.100e-007  3.102e-007    -6.509    -6.508     0.000
   NaCO3-               2.709e-009  2.559e-009    -8.567    -8.592    -0.025
   NaF                  2.228e-009  2.229e-009    -8.652    -8.652     0.000
   NaOH                 6.285e-011  6.289e-011   -10.202   -10.201     0.000
O(0)            0.000e+000
   O2                   0.000e+000  0.000e+000   -44.981   -44.980     0.000
S(6)            9.315e-005
   SO4-2                8.707e-005  6.954e-005    -4.060    -4.158    -0.098
   MgSO4                4.045e-006  4.047e-006    -5.393    -5.393     0.000
   CaSO4                1.689e-006  1.690e-006    -5.772    -5.772     0.000
   NaSO4-               3.326e-007  3.142e-007    -6.478    -6.503    -0.025
   KSO4-                1.442e-008  1.362e-008    -7.841    -7.866    -0.025
   ZnSO4                3.563e-009  3.566e-009    -8.448    -8.448     0.000
   MnSO4                1.947e-009  1.948e-009    -8.711    -8.710     0.000
   HSO4-                5.985e-010  5.654e-010    -9.223    -9.248    -0.025
   BaSO4                3.353e-010  3.356e-010    -9.475    -9.474     0.000
   CuSO4                1.689e-010  1.690e-010    -9.772    -9.772     0.000
   Zn(SO4)2-2           2.708e-012  2.156e-012   -11.567   -11.666    -0.099
   CaHSO4+              9.511e-013  8.984e-013   -12.022   -12.047    -0.025
Si              7.021e-002
   H4SiO4               7.013e-002  7.017e-002    -1.154    -1.154     0.000
   H3SiO4-              8.012e-005  7.568e-005    -4.096    -4.121    -0.025
   H2SiO4-2             3.612e-011  2.876e-011   -10.442   -10.541    -0.099
   SiF6-2               1.353e-031  1.077e-031   -30.869   -30.968    -0.099
Zn              3.273e-007
   Zn+2                 2.929e-007  2.340e-007    -6.533    -6.631    -0.098
   ZnHCO3+              1.805e-008  1.705e-008    -7.744    -7.768    -0.025
   ZnCO3                1.046e-008  1.047e-008    -7.981    -7.980     0.000
   ZnSO4                3.563e-009  3.566e-009    -8.448    -8.448     0.000
   ZnOH+                1.397e-009  1.319e-009    -8.855    -8.880    -0.025
   ZnCl+                5.354e-010  5.057e-010    -9.271    -9.296    -0.025
   Zn(OH)2              2.936e-010  2.938e-010    -9.532    -9.532     0.000
   Zn(CO3)2-2           6.299e-011  5.016e-011   -10.201   -10.300    -0.099
   Zn(SO4)2-2           2.708e-012  2.156e-012   -11.567   -11.666    -0.099
   ZnCl2                6.035e-013  6.039e-013   -12.219   -12.219     0.000
   Zn(OH)3-             9.824e-015  9.280e-015   -14.008   -14.032    -0.025
   ZnCl3-               8.039e-016  7.594e-016   -15.095   -15.120    -0.025
   ZnCl4-2              5.267e-019  4.194e-019   -18.278   -18.377    -0.099
   Zn(OH)4-2            1.845e-020  1.469e-020   -19.734   -19.833    -0.099

------------------------------Saturation indices-------------------------------

      Phase               SI log IAP  log KT

      Anhydrite        -3.70   -8.04   -4.34  CaSO4
      Aragonite        -2.24  -10.53   -8.29  CaCO3
      Barite           -2.06  -12.17  -10.12  BaSO4
      Calcite          -2.09  -10.53   -8.44  CaCO3
      Chalcedony        2.50   -1.15   -3.65  SiO2
      Chrysotile       -4.11   29.17   33.28  Mg3Si2O5(OH)4
      CO2(g)           -2.47   -3.83   -1.36  CO2
      Dolomite         -3.80  -20.68  -16.89  CaMg(CO3)2
      Fluorite         -3.96  -14.66  -10.71  CaF2
      Gypsum           -3.45   -8.04   -4.58  CaSO4:2H2O
      H2(g)           -22.00  -25.11   -3.11  H2
      H2O(g)           -1.74   -0.00    1.74  H2O
```

Halite	-7.51	-5.95	1.56	NaCl
Hausmannite	-19.39	43.81	63.19	Mn3O4
Manganite	-7.07	18.27	25.34	MnOOH
O2(g)	-42.06	-44.98	-2.92	O2
Pyrochroite	-7.93	7.27	15.20	Mn(OH)2
Pyrolusite	-13.51	29.27	42.78	MnO2
Quartz	2.96	-1.15	-4.11	SiO2
Rhodochrosite	-2.28	-13.38	-11.10	MnCO3
Sepiolite	1.53	17.52	15.99	Mg2Si3O7.5OH:3H2O
Sepiolite(d)	-1.14	17.52	18.66	Mg2Si3O7.5OH:3H2O
SiO2(a)	1.63	-1.15	-2.78	SiO2
Smithsonite	-3.37	-13.28	-9.91	ZnCO3
Talc	4.47	26.87	22.40	Mg3Si4O10(OH)2
Willemite	-2.46	13.58	16.05	Zn2SiO4
Witherite	-6.08	-14.67	-8.59	BaCO3
Zn(OH)2(e)	-4.13	7.37	11.50	Zn(OH)2

Initial solution 5.   4LJuanita-1

----------------------------Solution composition------------------------------

Elements	Molality	Moles
Alkalinity	4.608e-003	4.608e-003
Ba	1.465e-009	1.465e-009
Ca	4.443e-004	4.443e-004
Cl	1.760e-003	1.760e-003
Cu	2.058e-008	2.058e-008
K	3.242e-005	3.242e-005
Mg	7.284e-004	7.284e-004
Mn	7.875e-008	7.875e-008
Na	2.608e-003	2.608e-003
S(6)	5.719e-005	5.719e-005
Si	9.545e-002	9.545e-002
Zn	1.047e-007	1.047e-007

----------------------------Description of solution----------------------------

```
                                       pH  =   7.000
                                       pe  =   4.000
                        Activity of water  =   0.998
                           Ionic strength  =  6.861e-003
                       Mass of water (kg)  =  1.000e+000
                    Total carbon (mol/kg)  =  5.735e-003
                       Total CO2 (mol/kg)  =  5.735e-003
                      Temperature (deg C)  =   9.400
                  Electrical balance (eq)  = -1.495e-003
 Percent error, 100*(Cat-|An|)/(Cat+|An|)  = -13.16
                               Iterations  =  11
                                  Total H  = 1.113987e+002
                                  Total O  = 5.590425e+001
```

----------------------------Distribution of species----------------------------

Species		Molality	Activity	Log Molality	Log Activity	Log Gamma
H+		1.079e-007	1.000e-007	-6.967	-7.000	-0.033
OH-		3.018e-008	2.768e-008	-7.520	-7.558	-0.038
H2O		5.551e+001	9.982e-001	1.744	-0.001	0.000
Ba	1.465e-009					
Ba+2		1.419e-009	1.016e-009	-8.848	-8.993	-0.145
BaHCO3+		2.673e-011	2.455e-011	-10.573	-10.610	-0.037
BaSO4		1.889e-011	1.892e-011	-10.724	-10.723	0.001
BaCO3		5.043e-013	5.051e-013	-12.297	-12.297	0.001
BaOH+		3.742e-016	3.436e-016	-15.427	-15.464	-0.037
C(4)	5.735e-003					

	HCO3-		4.474e-003	4.119e-003	-2.349	-2.385	-0.036
	CO2		1.214e-003	1.216e-003	-2.916	-2.915	0.001
	MgHCO3+		2.538e-005	2.330e-005	-4.596	-4.633	-0.037
	CaHCO3+		1.259e-005	1.159e-005	-4.900	-4.936	-0.036
	NaHCO3		5.532e-006	5.540e-006	-5.257	-5.256	0.001
	CO3-2		1.833e-006	1.316e-006	-5.737	-5.881	-0.144
	CaCO3		5.513e-007	5.522e-007	-6.259	-6.258	0.001
	MgCO3		4.978e-007	4.986e-007	-6.303	-6.302	0.001
	NaCO3-		2.783e-008	2.556e-008	-7.556	-7.593	-0.037
	ZnHCO3+		2.628e-008	2.414e-008	-7.580	-7.617	-0.037
	MnHCO3+		1.655e-008	1.520e-008	-7.781	-7.818	-0.037
	ZnCO3		1.220e-008	1.222e-008	-7.913	-7.913	0.001
	MnCO3		4.322e-009	4.329e-009	-8.364	-8.364	0.001
	Zn(CO3)2-2		4.837e-010	3.440e-010	-9.315	-9.463	-0.148
	BaHCO3+		2.673e-011	2.455e-011	-10.573	-10.610	-0.037
	BaCO3		5.043e-013	5.051e-013	-12.297	-12.297	0.001
Ca		4.443e-004					
	Ca+2		4.292e-004	3.081e-004	-3.367	-3.511	-0.144
	CaHCO3+		1.259e-005	1.159e-005	-4.900	-4.936	-0.036
	CaSO4		1.955e-006	1.958e-006	-5.709	-5.708	0.001
	CaCO3		5.513e-007	5.522e-007	-6.259	-6.258	0.001
	CaOH+		5.558e-010	5.104e-010	-9.255	-9.292	-0.037
	CaHSO4+		1.061e-012	9.743e-013	-11.974	-12.011	-0.037
Cl		1.760e-003					
	Cl-		1.760e-003	1.614e-003	-2.755	-2.792	-0.038
	MnCl+		2.965e-010	2.722e-010	-9.528	-9.565	-0.037
	ZnCl+		1.066e-010	9.785e-011	-9.972	-10.009	-0.037
	MnCl2		1.915e-013	1.918e-013	-12.718	-12.717	0.001
	ZnCl2		1.546e-013	1.548e-013	-12.811	-12.810	0.001
	ZnCl3-		2.766e-016	2.540e-016	-15.558	-15.595	-0.037
	MnCl3-		9.286e-017	8.528e-017	-16.032	-16.069	-0.037
	ZnCl4-2		2.536e-019	1.803e-019	-18.596	-18.744	-0.148
Cu(1)		2.790e-010					
	Cu+		2.790e-010	2.552e-010	-9.554	-9.593	-0.039
Cu(2)		2.031e-008					
	Cu(OH)2		1.179e-008	1.181e-008	-7.929	-7.928	0.001
	Cu+2		7.861e-009	5.672e-009	-8.105	-8.246	-0.142
	CuOH+		6.168e-010	5.662e-010	-9.210	-9.247	-0.037
	CuSO4		3.834e-011	3.840e-011	-10.416	-10.416	0.001
	Cu(OH)3-		7.733e-015	7.102e-015	-14.112	-14.149	-0.037
	Cu(OH)4-2		1.989e-020	1.414e-020	-19.701	-19.849	-0.148
H(0)		1.665e-025					
	H2		8.327e-026	8.340e-026	-25.080	-25.079	0.001
K		3.242e-005					
	K+		3.242e-005	2.973e-005	-4.489	-4.527	-0.038
	KSO4-		6.341e-009	5.823e-009	-8.198	-8.235	-0.037
	KOH		1.027e-012	1.029e-012	-11.988	-11.988	0.001
Mg		7.284e-004					
	Mg+2		6.996e-004	5.045e-004	-3.155	-3.297	-0.142
	MgHCO3+		2.538e-005	2.330e-005	-4.596	-4.633	-0.037
	MgSO4		2.870e-006	2.875e-006	-5.542	-5.541	0.001
	MgCO3		4.978e-007	4.986e-007	-6.303	-6.302	0.001
	MgOH+		4.503e-009	4.135e-009	-8.347	-8.384	-0.037
Mn(2)		7.875e-008					
	Mn+2		5.738e-008	4.140e-008	-7.241	-7.383	-0.142
	MnHCO3+		1.655e-008	1.520e-008	-7.781	-7.818	-0.037
	MnCO3		4.322e-009	4.329e-009	-8.364	-8.364	0.001
	MnCl+		2.965e-010	2.722e-010	-9.528	-9.565	-0.037
	MnSO4		1.995e-010	1.998e-010	-9.700	-9.699	0.001
	MnOH+		3.023e-012	2.776e-012	-11.520	-11.557	-0.037
	MnCl2		1.915e-013	1.918e-013	-12.718	-12.717	0.001
	MnCl3-		9.286e-017	8.528e-017	-16.032	-16.069	-0.037
Mn(3)		2.489e-030					
	Mn+3		2.489e-030	1.156e-030	-29.604	-29.937	-0.333
Na		2.608e-003					
	Na+		2.602e-003	2.392e-003	-2.585	-2.621	-0.037
	NaHCO3		5.532e-006	5.540e-006	-5.257	-5.256	0.001

NaSO4-		4.369e-007	4.012e-007	-6.360	-6.397	-0.037
NaCO3-		2.783e-008	2.556e-008	-7.556	-7.593	-0.037
NaOH		1.575e-010	1.577e-010	-9.803	-9.802	0.001
O(0)	0.000e+000					
O2		0.000e+000	0.000e+000	-47.537	-47.536	0.001
S(6)	5.719e-005					
SO4-2		5.192e-005	3.715e-005	-4.285	-4.430	-0.145
MgSO4		2.870e-006	2.875e-006	-5.542	-5.541	0.001
CaSO4		1.955e-006	1.958e-006	-5.709	-5.708	0.001
NaSO4-		4.369e-007	4.012e-007	-6.360	-6.397	-0.037
KSO4-		6.341e-009	5.823e-009	-8.198	-8.235	-0.037
ZnSO4		3.565e-010	3.571e-010	-9.448	-9.447	0.001
HSO4-		2.864e-010	2.630e-010	-9.543	-9.580	-0.037
MnSO4		1.995e-010	1.998e-010	-9.700	-9.699	0.001
CuSO4		3.834e-011	3.840e-011	-10.416	-10.416	0.001
BaSO4		1.889e-011	1.892e-011	-10.724	-10.723	0.001
CaHSO4+		1.061e-012	9.743e-013	-11.974	-12.011	-0.037
Zn(SO4)2-2		1.721e-013	1.224e-013	-12.764	-12.912	-0.148
Si	9.545e-002					
H4SiO4		9.537e-002	9.552e-002	-1.021	-1.020	0.001
H3SiO4-		8.397e-005	7.711e-005	-4.076	-4.113	-0.037
H2SiO4-2		2.418e-011	1.719e-011	-10.617	-10.765	-0.148
Zn	1.047e-007					
Zn+2		6.501e-008	4.655e-008	-7.187	-7.332	-0.145
ZnHCO3+		2.628e-008	2.414e-008	-7.580	-7.617	-0.037
ZnCO3		1.220e-008	1.222e-008	-7.913	-7.913	0.001
Zn(CO3)2-2		4.837e-010	3.440e-010	-9.315	-9.463	-0.148
ZnSO4		3.565e-010	3.571e-010	-9.448	-9.447	0.001
ZnOH+		1.592e-010	1.462e-010	-9.798	-9.835	-0.037
ZnCl+		1.066e-010	9.785e-011	-9.972	-10.009	-0.037
Zn(OH)2		5.829e-011	5.839e-011	-10.234	-10.234	0.001
Zn(SO4)2-2		1.721e-013	1.224e-013	-12.764	-12.912	-0.148
ZnCl2		1.546e-013	1.548e-013	-12.811	-12.810	0.001
Zn(OH)3-		2.007e-015	1.843e-015	-14.697	-14.734	-0.037
ZnCl3-		2.766e-016	2.540e-016	-15.558	-15.595	-0.037
ZnCl4-2		2.536e-019	1.803e-019	-18.596	-18.744	-0.148
Zn(OH)4-2		4.100e-021	2.916e-021	-20.387	-20.535	-0.148

------------------------------Saturation indices-------------------------------

Phase	SI	log IAP	log KT	
Anhydrite	-3.60	-7.94	-4.34	CaSO4
Aragonite	-1.14	-9.39	-8.25	CaCO3
Barite	-3.16	-13.42	-10.26	BaSO4
Calcite	-0.98	-9.39	-8.41	CaCO3
Chalcedony	2.72	-1.02	-3.74	SiO2
Chrysotile	-4.17	30.07	34.24	Mg3Si2O5(OH)4
CO2(g)	-1.65	-2.92	-1.26	CO2
Dolomite	-1.86	-18.57	-16.71	CaMg(CO3)2
Gypsum	-3.35	-7.94	-4.59	CaSO4:2H2O
H2(g)	-22.00	-25.08	-3.08	H2
H2O(g)	-1.94	-0.00	1.94	H2O
Halite	-6.96	-5.41	1.54	NaCl
Hausmannite	-23.25	41.85	65.10	Mn3O4
Manganite	-7.72	17.62	25.34	MnOOH
O2(g)	-44.65	-47.54	-2.89	O2
Pyrochroite	-8.58	6.62	15.20	Mn(OH)2
Pyrolusite	-15.40	28.62	44.01	MnO2
Quartz	3.20	-1.02	-4.22	SiO2
Rhodochrosite	-2.19	-13.26	-11.07	MnCO3
Sepiolite	2.15	18.35	16.19	Mg2Si3O7.5OH:3H2O
Sepiolite(d)	-0.31	18.35	18.66	Mg2Si3O7.5OH:3H2O
SiO2(a)	1.83	-1.02	-2.85	SiO2
Smithsonite	-3.39	-13.21	-9.82	ZnCO3
Talc	4.76	28.03	23.27	Mg3Si4O10(OH)2
Willemite	-4.36	12.32	16.68	Zn2SiO4

Witherite	-6.24	-14.87	-8.64	BaCO3
Zn(OH)2(e)	-4.83	6.67	11.50	Zn(OH)2

Initial solution 6. 4LMaría-1

----------------------------Solution composition----------------------------

Elements	Molality	Moles
Alkalinity	8.294e-004	8.294e-004
Ba	1.535e-008	1.535e-008
Ca	1.403e-004	1.403e-004
Cl	9.708e-004	9.708e-004
Cu	4.266e-008	4.266e-008
K	2.619e-005	2.619e-005
Mg	2.998e-004	2.998e-004
Mn	2.138e-007	2.138e-007
Na	1.013e-003	1.013e-003
S(6)	4.651e-005	4.651e-005
Si	6.349e-002	6.349e-002
Zn	1.981e-007	1.981e-007

----------------------------Description of solution----------------------------

pH	=	7.000
pe	=	4.000
Activity of water	=	0.999
Ionic strength	=	2.378e-003
Mass of water (kg)	=	1.000e+000
Total carbon (mol/kg)	=	9.482e-004
Total CO2 (mol/kg)	=	9.482e-004
Temperature (deg C)	=	15.200
Electrical balance (eq)	=	2.720e-005
Percent error, 100*(Cat-|An|)/(Cat+|An|)	=	0.72
Iterations	=	9
Total H	=	1.112671e+002
Total O	=	5.576303e+001

----------------------------Distribution of species----------------------------

Species		Molality	Activity	Log Molality	Log Activity	Log Gamma
H+		1.050e-007	1.000e-007	-6.979	-7.000	-0.021
OH-		4.830e-008	4.579e-008	-7.316	-7.339	-0.023
H2O		5.551e+001	9.989e-001	1.744	-0.000	0.000
Ba	1.535e-008					
Ba+2		1.507e-008	1.223e-008	-7.822	-7.913	-0.091
BaSO4		2.190e-010	2.191e-010	-9.660	-9.659	0.000
BaHCO3+		6.521e-011	6.186e-011	-10.186	-10.209	-0.023
BaCO3		1.396e-012	1.397e-012	-11.855	-11.855	0.000
BaOH+		4.364e-015	4.139e-015	-14.360	-14.383	-0.023
C(4)	9.482e-004					
HCO3-		7.566e-004	7.183e-004	-3.121	-3.144	-0.023
CO2		1.878e-004	1.879e-004	-3.726	-3.726	0.000
MgHCO3+		2.066e-006	1.959e-006	-5.685	-5.708	-0.023
CaHCO3+		9.035e-007	8.579e-007	-6.044	-6.067	-0.023
NaHCO3		3.879e-007	3.881e-007	-6.411	-6.411	0.000
CO3-2		3.313e-007	2.692e-007	-6.480	-6.570	-0.090
MgCO3		5.324e-008	5.327e-008	-7.274	-7.274	0.000
CaCO3		4.343e-008	4.346e-008	-7.362	-7.362	0.000
ZnHCO3+		1.352e-008	1.282e-008	-7.869	-7.892	-0.023
MnHCO3+		1.088e-008	1.032e-008	-7.963	-7.986	-0.023
ZnCO3		7.612e-009	7.617e-009	-8.118	-8.118	0.000
MnCO3		3.446e-009	3.448e-009	-8.463	-8.462	0.000
NaCO3-		3.046e-009	2.889e-009	-8.516	-8.539	-0.023
BaHCO3+		6.521e-011	6.186e-011	-10.186	-10.209	-0.023

	Zn(CO3)2-2	5.416e-011	4.384e-011	-10.266	-10.358	-0.092
	BaCO3	1.396e-012	1.397e-012	-11.855	-11.855	0.000
Ca	1.403e-004					
	Ca+2	1.386e-004	1.126e-004	-3.858	-3.948	-0.090
	CaHCO3+	9.035e-007	8.579e-007	-6.044	-6.067	-0.023
	CaSO4	7.302e-007	7.306e-007	-6.137	-6.136	0.000
	CaCO3	4.343e-008	4.346e-008	-7.362	-7.362	0.000
	CaOH+	1.968e-010	1.867e-010	-9.706	-9.729	-0.023
	CaHSO4+	4.041e-013	3.833e-013	-12.393	-12.416	-0.023
Cl	9.708e-004					
	Cl-	9.708e-004	9.205e-004	-3.013	-3.036	-0.023
	MnCl+	6.373e-010	6.045e-010	-9.196	-9.219	-0.023
	ZnCl+	2.369e-010	2.247e-010	-9.625	-9.648	-0.023
	MnCl2	2.427e-013	2.429e-013	-12.615	-12.615	0.000
	ZnCl2	2.078e-013	2.079e-013	-12.682	-12.682	0.000
	ZnCl3-	2.130e-016	2.021e-016	-15.672	-15.695	-0.023
	MnCl3-	6.491e-017	6.157e-017	-16.188	-16.211	-0.023
	ZnCl4-2	1.063e-019	8.602e-020	-18.974	-19.065	-0.092
Cu(1)	6.188e-010					
	Cu+	6.188e-010	5.862e-010	-9.208	-9.232	-0.024
Cu(2)	4.204e-008					
	Cu(OH)2	2.558e-008	2.560e-008	-7.592	-7.592	0.000
	Cu+2	1.508e-008	1.228e-008	-7.822	-7.911	-0.089
	CuOH+	1.293e-009	1.226e-009	-8.888	-8.911	-0.023
	CuSO4	8.351e-011	8.355e-011	-10.078	-10.078	0.000
	Cu(OH)3-	1.624e-014	1.540e-014	-13.789	-13.812	-0.023
	Cu(OH)4-2	3.793e-020	3.070e-020	-19.421	-19.513	-0.092
H(0)	1.565e-025					
	H2	7.827e-026	7.831e-026	-25.106	-25.106	0.000
K	2.619e-005					
	K+	2.618e-005	2.483e-005	-4.582	-4.605	-0.023
	KSO4-	5.508e-009	5.225e-009	-8.259	-8.282	-0.023
	KOH	8.593e-013	8.598e-013	-12.066	-12.066	0.000
Mg	2.998e-004					
	Mg+2	2.961e-004	2.410e-004	-3.529	-3.618	-0.089
	MgHCO3+	2.066e-006	1.959e-006	-5.685	-5.708	-0.023
	MgSO4	1.555e-006	1.556e-006	-5.808	-5.808	0.000
	MgCO3	5.324e-008	5.327e-008	-7.274	-7.274	0.000
	MgOH+	3.691e-009	3.501e-009	-8.433	-8.456	-0.023
Mn(2)	2.138e-007					
	Mn+2	1.980e-007	1.612e-007	-6.703	-6.793	-0.089
	MnHCO3+	1.088e-008	1.032e-008	-7.963	-7.986	-0.023
	MnCO3	3.446e-009	3.448e-009	-8.463	-8.462	0.000
	MnSO4	8.441e-010	8.445e-010	-9.074	-9.073	0.000
	MnCl+	6.373e-010	6.045e-010	-9.196	-9.219	-0.023
	MnOH+	1.910e-011	1.812e-011	-10.719	-10.742	-0.023
	MnCl2	2.427e-013	2.429e-013	-12.615	-12.615	0.000
	MnCl3-	6.491e-017	6.157e-017	-16.188	-16.211	-0.023
Mn(3)	1.825e-029					
	Mn+3	1.825e-029	1.134e-029	-28.739	-28.945	-0.207
Na	1.013e-003					
	Na+	1.013e-003	9.608e-004	-2.995	-3.017	-0.023
	NaHCO3	3.879e-007	3.881e-007	-6.411	-6.411	0.000
	NaSO4-	1.702e-007	1.614e-007	-6.769	-6.792	-0.023
	NaCO3-	3.046e-009	2.889e-009	-8.516	-8.539	-0.023
	NaOH	6.337e-011	6.340e-011	-10.198	-10.198	0.000
O(0)	0.000e+000					
	O2	0.000e+000	0.000e+000	-45.439	-45.439	0.000
S(6)	4.651e-005					
	SO4-2	4.404e-005	3.574e-005	-4.356	-4.447	-0.091
	MgSO4	1.555e-006	1.556e-006	-5.808	-5.808	0.000
	CaSO4	7.302e-007	7.306e-007	-6.137	-6.136	0.000
	NaSO4-	1.702e-007	1.614e-007	-6.769	-6.792	-0.023
	KSO4-	5.508e-009	5.225e-009	-8.259	-8.282	-0.023
	ZnSO4	1.098e-009	1.099e-009	-8.959	-8.959	0.000
	MnSO4	8.441e-010	8.445e-010	-9.074	-9.073	0.000
	HSO4-	2.985e-010	2.831e-010	-9.525	-9.548	-0.023

```
   BaSO4               2.190e-010  2.191e-010    -9.660    -9.659     0.000
   CuSO4               8.351e-011  8.355e-011   -10.078   -10.078     0.000
   Zn(SO4)2-2          4.264e-013  3.452e-013   -12.370   -12.462    -0.092
   CaHSO4+             4.041e-013  3.833e-013   -12.393   -12.416    -0.023
Si                6.349e-002
   H4SiO4              6.343e-002  6.346e-002    -1.198    -1.198     0.000
   H3SiO4-             6.857e-005  6.504e-005    -4.164    -4.187    -0.023
   H2SiO4-2            2.778e-011  2.249e-011   -10.556   -10.648    -0.092
Zn                1.981e-007
   Zn+2                1.747e-007  1.418e-007    -6.758    -6.848    -0.091
   ZnHCO3+             1.352e-008  1.282e-008    -7.869    -7.892    -0.023
   ZnCO3               7.612e-009  7.617e-009    -8.118    -8.118     0.000
   ZnSO4               1.098e-009  1.099e-009    -8.959    -8.959     0.000
   ZnOH+               7.590e-010  7.200e-010    -9.120    -9.143    -0.023
   ZnCl+               2.369e-010  2.247e-010    -9.625    -9.648    -0.023
   Zn(OH)2             1.780e-010  1.781e-010    -9.750    -9.749     0.000
   Zn(CO3)2-2          5.416e-011  4.384e-011   -10.266   -10.358    -0.092
   Zn(SO4)2-2          4.264e-013  3.452e-013   -12.370   -12.462    -0.092
   ZnCl2               2.078e-013  2.079e-013   -12.682   -12.682     0.000
   Zn(OH)3-            5.930e-015  5.625e-015   -14.227   -14.250    -0.023
   ZnCl3-              2.130e-016  2.021e-016   -15.672   -15.695    -0.023
   ZnCl4-2             1.063e-019  8.602e-020   -18.974   -19.065    -0.092
   Zn(OH)4-2           1.100e-020  8.905e-021   -19.959   -20.050    -0.092

------------------------------Saturation indices-------------------------------

      Phase               SI log IAP  log KT

      Anhydrite        -4.06   -8.40   -4.34  CaSO4
      Aragonite        -2.24  -10.52   -8.28  CaCO3
      Barite           -2.22  -12.36  -10.14  BaSO4
      Calcite          -2.09  -10.52   -8.43  CaCO3
      Chalcedony        2.47   -1.20   -3.67  SiO2
      Chrysotile       -4.70   28.75   33.46  Mg3Si2O5(OH)4
      CO2(g)           -2.38   -3.73   -1.34  CO2
      Dolomite         -3.85  -20.71  -16.85  CaMg(CO3)2
      Gypsum           -3.81   -8.40   -4.58  CaSO4:2H2O
      H2(g)           -22.00  -25.11   -3.11  H2
      H2O(g)           -1.77   -0.00    1.77  H2O
      Halite           -7.61   -6.05    1.56  NaCl
      Hausmannite     -19.92   43.62   63.54  Mn3O4
      Manganite        -7.13   18.21   25.34  MnOOH
      O2(g)           -42.52  -45.44   -2.91  O2
      Pyrochroite      -7.99    7.21   15.20  Mn(OH)2
      Pyrolusite      -13.80   29.21   43.00  MnO2
      Quartz            2.93   -1.20   -4.13  SiO2
      Rhodochrosite    -2.27  -13.36  -11.09  MnCO3
      Sepiolite         1.15   17.17   16.03  Mg2Si3O7.5OH:3H2O
      Sepiolite(d)     -1.49   17.17   18.66  Mg2Si3O7.5OH:3H2O
      SiO2(a)           1.60   -1.20   -2.80  SiO2
      Smithsonite      -3.53  -13.42   -9.89  ZnCO3
      Talc              3.80   26.36   22.55  Mg3Si4O10(OH)2
      Willemite        -3.06   13.11   16.16  Zn2SiO4
      Witherite        -5.89  -14.48   -8.60  BaCO3
      Zn(OH)2(e)       -4.35    7.15   11.50  Zn(OH)2

Initial solution 7.     4LMartina-1

-----------------------------Solution composition------------------------------

      Elements           Molality       Moles

      Alkalinity       2.011e-003  2.011e-003
      Ba               3.238e-008  3.238e-008
      Ca               9.685e-004  9.685e-004
      Cl               1.486e-002  1.486e-002
      Cu               6.681e-008  6.681e-008
```

K	1.081e-004	1.081e-004
Mg	3.763e-003	3.763e-003
Mn	3.330e-007	3.330e-007
Na	8.355e-003	8.355e-003
S(6)	4.719e-004	4.719e-004
Si	1.632e-001	1.632e-001
Zn	2.165e-007	2.165e-007

----------------------------Description of solution----------------------------

pH	=	7.000
pe	=	4.000
Activity of water	=	0.997
Ionic strength	=	2.253e-002
Mass of water (kg)	=	1.000e+000
Total carbon (mol/kg)	=	2.190e-003
Total CO2 (mol/kg)	=	2.190e-003
Temperature (deg C)	=	16.600
Electrical balance (eq)	=	1.093e-004
Percent error, 100*(Cat-|An|)/(Cat+|An|)	=	0.31
Iterations	=	9
Total H	=	1.116668e+002
Total O	=	5.616704e+001

----------------------------Distribution of species----------------------------

Species		Molality	Activity	Log Molality	Log Activity	Log Gamma
H+		1.128e-007	1.000e-007	-6.948	-7.000	-0.052
OH-		5.954e-008	5.134e-008	-7.225	-7.290	-0.064
H2O		5.551e+001	9.967e-001	1.744	-0.001	0.000
Ba	3.238e-008					
Ba+2		3.037e-008	1.739e-008	-7.518	-7.760	-0.242
BaSO4		1.789e-009	1.798e-009	-8.747	-8.745	0.002
BaHCO3+		2.242e-010	1.943e-010	-9.649	-9.712	-0.062
BaCO3		4.451e-012	4.474e-012	-11.352	-11.349	0.002
BaOH+		6.775e-015	5.871e-015	-14.169	-14.231	-0.062
C(4)	2.190e-003					
HCO3-		1.742e-003	1.519e-003	-2.759	-2.819	-0.060
CO2		3.862e-004	3.882e-004	-3.413	-3.411	0.002
MgHCO3+		4.233e-005	3.668e-005	-4.373	-4.436	-0.062
CaHCO3+		1.032e-005	8.992e-006	-4.987	-5.046	-0.060
NaHCO3		6.152e-006	6.184e-006	-5.211	-5.209	0.002
MgCO3		1.047e-006	1.052e-006	-5.980	-5.978	0.002
CO3-2		1.021e-006	5.897e-007	-5.991	-6.229	-0.238
CaCO3		4.631e-007	4.655e-007	-6.334	-6.332	0.002
NaCO3-		5.934e-008	5.142e-008	-7.227	-7.289	-0.062
MnHCO3+		2.595e-008	2.249e-008	-7.586	-7.648	-0.062
ZnHCO3+		2.203e-008	1.910e-008	-7.657	-7.719	-0.062
ZnCO3		1.169e-008	1.175e-008	-7.932	-7.930	0.002
MnCO3		7.742e-009	7.782e-009	-8.111	-8.109	0.002
Zn(CO3)2-2		2.627e-010	1.482e-010	-9.581	-9.829	-0.249
BaHCO3+		2.242e-010	1.943e-010	-9.649	-9.712	-0.062
BaCO3		4.451e-012	4.474e-012	-11.352	-11.349	0.002
Ca	9.685e-004					
Ca+2		9.372e-004	5.412e-004	-3.028	-3.267	-0.239
CaSO4		2.045e-005	2.056e-005	-4.689	-4.687	0.002
CaHCO3+		1.032e-005	8.992e-006	-4.987	-5.046	-0.060
CaCO3		4.631e-007	4.655e-007	-6.334	-6.332	0.002
CaOH+		1.033e-009	8.952e-010	-8.986	-9.048	-0.062
CaHSO4+		1.262e-011	1.094e-011	-10.899	-10.961	-0.062
Cl	1.486e-002					
Cl-		1.486e-002	1.283e-002	-1.828	-1.892	-0.064
MnCl+		1.002e-008	8.680e-009	-7.999	-8.061	-0.062
ZnCl+		2.718e-009	2.355e-009	-8.566	-8.628	-0.062
MnCl2		4.834e-011	4.859e-011	-10.316	-10.313	0.002

ZnCl2		3.039e-011	3.055e-011	-10.517	-10.515	0.002
ZnCl3-		4.816e-013	4.174e-013	-12.317	-12.379	-0.062
MnCl3-		1.981e-013	1.717e-013	-12.703	-12.765	-0.062
ZnCl4-2		4.442e-015	2.505e-015	-14.352	-14.601	-0.249
Cu(1)	9.471e-010					
Cu+		9.471e-010	8.114e-010	-9.024	-9.091	-0.067
Cu(2)	6.586e-008					
Cu(OH)2		3.461e-008	3.479e-008	-7.461	-7.459	0.002
Cu+2		2.866e-008	1.676e-008	-7.543	-7.776	-0.233
CuOH+		1.931e-009	1.670e-009	-8.714	-8.777	-0.063
CuSO4		6.618e-010	6.653e-010	-9.179	-9.177	0.002
Cu(OH)3-		2.411e-014	2.089e-014	-13.618	-13.680	-0.062
Cu(OH)4-2		7.366e-020	4.155e-020	-19.133	-19.381	-0.249
H(0)	1.535e-025					
H2		7.676e-026	7.716e-026	-25.115	-25.113	0.002
K	1.081e-004					
K+		1.079e-004	9.313e-005	-3.967	-4.031	-0.064
KSO4-		1.340e-007	1.161e-007	-6.873	-6.935	-0.062
KOH		3.202e-012	3.219e-012	-11.495	-11.492	0.002
Mg	3.763e-003					
Mg+2		3.637e-003	2.127e-003	-2.439	-2.672	-0.233
MgSO4		8.193e-005	8.236e-005	-4.087	-4.084	0.002
MgHCO3+		4.233e-005	3.668e-005	-4.373	-4.436	-0.062
MgCO3		1.047e-006	1.052e-006	-5.980	-5.978	0.002
MgOH+		4.069e-008	3.527e-008	-7.390	-7.453	-0.062
Mn(2)	3.330e-007					
Mn+2		2.841e-007	1.661e-007	-6.547	-6.780	-0.233
MnHCO3+		2.595e-008	2.249e-008	-7.586	-7.648	-0.062
MnCl+		1.002e-008	8.680e-009	-7.999	-8.061	-0.062
MnCO3		7.742e-009	7.782e-009	-8.111	-8.109	0.002
MnSO4		5.143e-009	5.170e-009	-8.289	-8.287	0.002
MnCl2		4.834e-011	4.859e-011	-10.316	-10.313	0.002
MnOH+		2.428e-011	2.104e-011	-10.615	-10.677	-0.062
MnCl3-		1.981e-013	1.717e-013	-12.703	-12.765	-0.062
Mn(3)	5.270e-029					
Mn+3		5.270e-029	1.453e-029	-28.278	-28.838	-0.560
Na	8.355e-003					
Na+		8.340e-003	7.242e-003	-2.079	-2.140	-0.061
NaSO4-		8.182e-006	7.091e-006	-5.087	-5.149	-0.062
NaHCO3		6.152e-006	6.184e-006	-5.211	-5.209	0.002
NaCO3-		5.934e-008	5.142e-008	-7.227	-7.289	-0.062
NaOH		4.744e-010	4.769e-010	-9.324	-9.322	0.002
O(0)	0.000e+000					
O2		0.000e+000	0.000e+000	-44.949	-44.947	0.002
S(6)	4.719e-004					
SO4-2		3.612e-004	2.064e-004	-3.442	-3.685	-0.243
MgSO4		8.193e-005	8.236e-005	-4.087	-4.084	0.002
CaSO4		2.045e-005	2.056e-005	-4.689	-4.687	0.002
NaSO4-		8.182e-006	7.091e-006	-5.087	-5.149	-0.062
KSO4-		1.340e-007	1.161e-007	-6.873	-6.935	-0.062
MnSO4		5.143e-009	5.170e-009	-8.289	-8.287	0.002
ZnSO4		4.497e-009	4.521e-009	-8.347	-8.345	0.002
HSO4-		1.940e-009	1.681e-009	-8.712	-8.774	-0.062
BaSO4		1.789e-009	1.798e-009	-8.747	-8.745	0.002
CuSO4		6.618e-010	6.653e-010	-9.179	-9.177	0.002
Zn(SO4)2-2		1.437e-011	8.105e-012	-10.843	-11.091	-0.249
CaHSO4+		1.262e-011	1.094e-011	-10.899	-10.961	-0.062
Si	1.632e-001					
H4SiO4		1.630e-001	1.638e-001	-0.788	-0.786	0.002
H3SiO4-		2.047e-004	1.774e-004	-3.689	-3.751	-0.062
H2SiO4-2		1.204e-010	6.789e-011	-9.919	-10.168	-0.249
Zn	2.165e-007					
Zn+2		1.745e-007	9.988e-008	-6.758	-7.001	-0.242
ZnHCO3+		2.203e-008	1.910e-008	-7.657	-7.719	-0.062
ZnCO3		1.169e-008	1.175e-008	-7.932	-7.930	0.002
ZnSO4		4.497e-009	4.521e-009	-8.347	-8.345	0.002
ZnCl+		2.718e-009	2.355e-009	-8.566	-8.628	-0.062

ZnOH+	6.538e-010	5.666e-010	-9.185	-9.247	-0.062
Zn(CO3)2-2	2.627e-010	1.482e-010	-9.581	-9.829	-0.249
Zn(OH)2	1.243e-010	1.249e-010	-9.906	-9.903	0.002
ZnCl2	3.039e-011	3.055e-011	-10.517	-10.515	0.002
Zn(SO4)2-2	1.437e-011	8.105e-012	-10.843	-11.091	-0.249
ZnCl3-	4.816e-013	4.174e-013	-12.317	-12.379	-0.062
Zn(OH)3-	4.543e-015	3.937e-015	-14.343	-14.405	-0.062
ZnCl4-2	4.442e-015	2.505e-015	-14.352	-14.601	-0.249
Zn(OH)4-2	1.103e-020	6.219e-021	-19.958	-20.206	-0.249

------------------------------Saturation indices-------------------------------

Phase	SI	log IAP	log KT	
Anhydrite	-2.61	-6.95	-4.34	CaSO4
Aragonite	-1.21	-9.50	-8.29	CaCO3
Barite	-1.33	-11.45	-10.11	BaSO4
Calcite	-1.06	-9.50	-8.44	CaCO3
Chalcedony	2.87	-0.78	-3.65	SiO2
Chrysotile	-0.86	32.41	33.27	Mg3Si2O5(OH)4
CO2(g)	-2.05	-3.41	-1.36	CO2
Dolomite	-1.51	-18.40	-16.89	CaMg(CO3)2
Gypsum	-2.37	-6.95	-4.58	CaSO4:2H2O
H2(g)	-22.00	-25.11	-3.11	H2
H2O(g)	-1.74	-0.00	1.73	H2O
Halite	-5.59	-4.03	1.56	NaCl
Hausmannite	-19.51	43.66	63.17	Mn3O4
Manganite	-7.12	18.22	25.34	MnOOH
O2(g)	-42.03	-44.95	-2.92	O2
Pyrochroite	-7.98	7.22	15.20	Mn(OH)2
Pyrolusite	-13.55	29.22	42.76	MnO2
Quartz	3.32	-0.78	-4.11	SiO2
Rhodochrosite	-1.91	-13.01	-11.10	MnCO3
Sepiolite	4.31	20.30	15.99	Mg2Si3O7.5OH:3H2O
Sepiolite(d)	1.64	20.30	18.66	Mg2Si3O7.5OH:3H2O
SiO2(a)	2.00	-0.78	-2.78	SiO2
Smithsonite	-3.32	-13.23	-9.91	ZnCO3
Talc	8.46	30.85	22.38	Mg3Si4O10(OH)2
Willemite	-2.83	13.21	16.04	Zn2SiO4
Witherite	-5.40	-13.99	-8.59	BaCO3
Zn(OH)2(e)	-4.50	7.00	11.50	Zn(OH)2

Initial solution 8.   4LNatasha-1

-----------------------------Solution composition------------------------------

Elements	Molality	Moles
Alkalinity	2.558e-003	2.558e-003
Ba	1.097e-008	1.097e-008
Ca	3.558e-004	3.558e-004
Cl	5.791e-003	5.791e-003
Cu	6.322e-008	6.322e-008
K	7.397e-005	7.397e-005
Mg	2.045e-003	2.045e-003
Mn	3.967e-007	3.967e-007
Na	3.456e-003	3.456e-003
S(6)	7.371e-005	7.371e-005
Si	6.352e-002	6.352e-002
Zn	2.258e-007	2.258e-007

----------------------------Description of solution----------------------------

pH = 7.000
pe = 4.000
Activity of water = 0.999
Ionic strength = 1.077e-002

```
                     Mass of water (kg)  =  1.000e+000
                   Total carbon (mol/kg)  =  3.329e-003
                      Total CO2 (mol/kg)  =  3.329e-003
                     Temperature (deg C)  =   1.400
                 Electrical balance (eq)  = -1.649e-004
 Percent error, 100*(Cat-|An|)/(Cat+|An|)  =  -0.99
                              Iterations  =  10
                                 Total H  = 1.112690e+002
                                 Total O  = 5.576976e+001
```

----------------------------Distribution of species----------------------------

Species		Molality	Activity	Log Molality	Log Activity	Log Gamma
H+		1.094e-007	1.000e-007	-6.961	-7.000	-0.039
OH-		1.455e-008	1.310e-008	-7.837	-7.883	-0.046
H2O		5.551e+001	9.987e-001	1.744	-0.001	0.000
Ba	1.097e-008					
Ba+2		1.073e-008	7.177e-009	-7.969	-8.144	-0.175
BaSO4		1.545e-010	1.549e-010	-9.811	-9.810	0.001
BaHCO3+		8.113e-011	7.320e-011	-10.091	-10.135	-0.045
BaCO3		1.273e-012	1.276e-012	-11.895	-11.894	0.001
BaOH+		2.692e-015	2.429e-015	-14.570	-14.615	-0.045
C(4)	3.329e-003					
HCO3-		2.470e-003	2.236e-003	-2.607	-2.650	-0.043
CO2		8.113e-004	8.133e-004	-3.091	-3.090	0.001
MgHCO3+		3.763e-005	3.395e-005	-4.424	-4.469	-0.045
CaHCO3+		4.055e-006	3.671e-006	-5.392	-5.435	-0.043
NaHCO3		3.910e-006	3.920e-006	-5.408	-5.407	0.001
CO3-2		8.227e-007	5.527e-007	-6.085	-6.257	-0.173
MgCO3		4.950e-007	4.962e-007	-6.305	-6.304	0.001
CaCO3		1.756e-007	1.760e-007	-6.756	-6.754	0.001
MnHCO3+		4.947e-008	4.463e-008	-7.306	-7.350	-0.045
ZnHCO3+		3.641e-008	3.285e-008	-7.439	-7.483	-0.045
ZnCO3		1.284e-008	1.287e-008	-7.892	-7.890	0.001
MnCO3		9.808e-009	9.832e-009	-8.008	-8.007	0.001
NaCO3-		9.763e-009	8.809e-009	-8.010	-8.055	-0.045
Zn(CO3)2-2		2.294e-010	1.521e-010	-9.639	-9.818	-0.179
BaHCO3+		8.113e-011	7.320e-011	-10.091	-10.135	-0.045
BaCO3		1.273e-012	1.276e-012	-11.895	-11.894	0.001
Ca	3.558e-004					
Ca+2		3.500e-004	2.350e-004	-3.456	-3.629	-0.173
CaHCO3+		4.055e-006	3.671e-006	-5.392	-5.435	-0.043
CaSO4		1.585e-006	1.589e-006	-5.800	-5.799	0.001
CaCO3		1.756e-007	1.760e-007	-6.756	-6.754	0.001
CaOH+		4.317e-010	3.895e-010	-9.365	-9.409	-0.045
CaHSO4+		8.279e-013	7.470e-013	-12.082	-12.127	-0.045
Cl	5.791e-003					
Cl-		5.791e-003	5.215e-003	-2.237	-2.283	-0.045
MnCl+		5.273e-009	4.758e-009	-8.278	-8.323	-0.045
ZnCl+		5.863e-010	5.290e-010	-9.232	-9.277	-0.045
MnCl2		1.080e-011	1.083e-011	-10.966	-10.965	0.001
ZnCl2		2.600e-012	2.606e-012	-11.585	-11.584	0.001
MnCl3-		1.724e-014	1.556e-014	-13.763	-13.808	-0.045
ZnCl3-		1.449e-014	1.308e-014	-13.839	-13.883	-0.045
ZnCl4-2		4.209e-017	2.790e-017	-16.376	-16.554	-0.179
Cu(1)	7.821e-010					
Cu+		7.821e-010	7.018e-010	-9.107	-9.154	-0.047
Cu(2)	6.244e-008					
Cu(OH)2		3.531e-008	3.540e-008	-7.452	-7.451	0.001
Cu+2		2.512e-008	1.699e-008	-7.600	-7.770	-0.170
CuOH+		1.882e-009	1.697e-009	-8.725	-8.770	-0.045
CuSO4		1.248e-010	1.251e-010	-9.904	-9.903	0.001
Cu(OH)3-		2.361e-014	2.130e-014	-13.627	-13.672	-0.045
Cu(OH)4-2		6.404e-020	4.245e-020	-19.194	-19.372	-0.179
H(0)	1.823e-025					

```
   H2                  9.115e-026  9.138e-026   -25.040   -25.039     0.001
K                 7.397e-005
   K+                  7.396e-005  6.660e-005    -4.131    -4.177    -0.045
   KSO4-               1.428e-008  1.288e-008    -7.845    -7.890    -0.045
   KOH                 2.301e-012  2.306e-012   -11.638   -11.637     0.001
Mg                2.045e-003
   Mg+2                2.000e-003  1.352e-003    -2.699    -2.869    -0.170
   MgHCO3+             3.763e-005  3.395e-005    -4.424    -4.469    -0.045
   MgSO4               7.033e-006  7.050e-006    -5.153    -5.152     0.001
   MgCO3               4.950e-007  4.962e-007    -6.305    -6.304     0.001
   MgOH+               5.369e-009  4.844e-009    -8.270    -8.315    -0.045
Mn(2)             3.967e-007
   Mn+2                3.311e-007  2.239e-007    -6.480    -6.650    -0.170
   MnHCO3+             4.947e-008  4.463e-008    -7.306    -7.350    -0.045
   MnCO3               9.808e-009  9.832e-009    -8.008    -8.007     0.001
   MnCl+               5.273e-009  4.758e-009    -8.278    -8.323    -0.045
   MnSO4               1.049e-009  1.051e-009    -8.979    -8.978     0.001
   MnCl2               1.080e-011  1.083e-011   -10.966   -10.965     0.001
   MnOH+               7.887e-012  7.116e-012   -11.103   -11.148    -0.045
   MnCl3-              1.724e-014  1.556e-014   -13.763   -13.808    -0.045
Mn(3)             4.135e-030
   Mn+3                4.135e-030  1.639e-030   -29.384   -29.785    -0.402
Na                3.456e-003
   Na+                 3.451e-003  3.117e-003    -2.462    -2.506    -0.044
   NaHCO3              3.910e-006  3.920e-006    -5.408    -5.407     0.001
   NaSO4-              6.337e-007  5.717e-007    -6.198    -6.243    -0.045
   NaCO3-              9.763e-009  8.809e-009    -8.010    -8.055    -0.045
   NaOH                2.052e-010  2.057e-010    -9.688    -9.687     0.001
O(0)              0.000e+000
   O2                  0.000e+000  0.000e+000   -50.575   -50.574     0.001
S(6)              7.371e-005
   SO4-2               6.444e-005  4.305e-005    -4.191    -4.366    -0.175
   MgSO4               7.033e-006  7.050e-006    -5.153    -5.152     0.001
   CaSO4               1.585e-006  1.589e-006    -5.800    -5.799     0.001
   NaSO4-              6.337e-007  5.717e-007    -6.198    -6.243    -0.045
   KSO4-               1.428e-008  1.288e-008    -7.845    -7.890    -0.045
   MnSO4               1.049e-009  1.051e-009    -8.979    -8.978     0.001
   ZnSO4               9.643e-010  9.667e-010    -9.016    -9.015     0.001
   HSO4-               2.930e-010  2.644e-010    -9.533    -9.578    -0.045
   BaSO4               1.545e-010  1.549e-010    -9.811    -9.810     0.001
   CuSO4               1.248e-010  1.251e-010    -9.904    -9.903     0.001
   CaHSO4+             8.279e-013  7.470e-013   -12.082   -12.127    -0.045
   Zn(SO4)2-2          6.217e-013  4.121e-013   -12.206   -12.385    -0.179
Si                6.352e-002
   H4SiO4              6.348e-002  6.364e-002    -1.197    -1.196     0.001
   H3SiO4-             3.965e-005  3.577e-005    -4.402    -4.446    -0.045
   H2SiO4-2            6.245e-012  4.139e-012   -11.204   -11.383    -0.179
Zn                2.258e-007
   Zn+2                1.745e-007  1.167e-007    -6.758    -6.933    -0.175
   ZnHCO3+             3.641e-008  3.285e-008    -7.439    -7.483    -0.045
   ZnCO3               1.284e-008  1.287e-008    -7.892    -7.890     0.001
   ZnSO4               9.643e-010  9.667e-010    -9.016    -9.015     0.001
   ZnCl+               5.863e-010  5.290e-010    -9.232    -9.277    -0.045
   Zn(CO3)2-2          2.294e-010  1.521e-010    -9.639    -9.818    -0.179
   ZnOH+               2.027e-010  1.829e-010    -9.693    -9.738    -0.045
   Zn(OH)2             1.461e-010  1.465e-010    -9.835    -9.834     0.001
   ZnCl2               2.600e-012  2.606e-012   -11.585   -11.584     0.001
   Zn(SO4)2-2          6.217e-013  4.121e-013   -12.206   -12.385    -0.179
   ZnCl3-              1.449e-014  1.308e-014   -13.839   -13.883    -0.045
   Zn(OH)3-            5.128e-015  4.627e-015   -14.290   -14.335    -0.045
   ZnCl4-2             4.209e-017  2.790e-017   -16.376   -16.554    -0.179
   Zn(OH)4-2           1.105e-020  7.323e-021   -19.957   -20.135    -0.179

------------------------------Saturation indices-------------------------------

      Phase               SI log IAP  log KT
```

Anhydrite	-3.64	-7.99	-4.36	CaSO4
Aragonite	-1.66	-9.89	-8.22	CaCO3
Barite	-2.07	-12.51	-10.44	BaSO4
Calcite	-1.50	-9.89	-8.38	CaCO3
Chalcedony	2.65	-1.20	-3.85	SiO2
Chrysotile	-4.37	31.00	35.37	Mg3Si2O5(OH)4
CO2(g)	-1.96	-3.09	-1.13	CO2
Dolomite	-2.52	-19.01	-16.50	CaMg(CO3)2
Gypsum	-3.38	-8.00	-4.61	CaSO4:2H2O
H2(g)	-22.00	-25.04	-3.04	H2
H2O(g)	-2.17	-0.00	2.17	H2O
Halite	-6.31	-4.79	1.52	NaCl
Hausmannite	-23.32	44.05	67.37	Mn3O4
Manganite	-6.99	18.35	25.34	MnOOH
O2(g)	-47.73	-50.57	-2.84	O2
Pyrochroite	-7.85	7.35	15.20	Mn(OH)2
Pyrolusite	-16.13	29.35	45.48	MnO2
Quartz	3.16	-1.20	-4.36	SiO2
Rhodochrosite	-1.87	-12.91	-11.04	MnCO3
Sepiolite	2.24	18.67	16.43	Mg2Si3O7.5OH:3H2O
Sepiolite(d)	0.01	18.67	18.66	Mg2Si3O7.5OH:3H2O
SiO2(a)	1.73	-1.20	-2.92	SiO2
Smithsonite	-3.47	-13.19	-9.73	ZnCO3
Talc	4.29	28.61	24.32	Mg3Si4O10(OH)2
Willemite	-4.49	12.94	17.43	Zn2SiO4
Witherite	-5.68	-14.40	-8.72	BaCO3
Zn(OH)2(e)	-4.43	7.07	11.50	Zn(OH)2

Initial solution 9.   4HNatasha-1

-----------------------------Solution composition------------------------------

Elements	Molality	Moles
Alkalinity	4.115e-003	4.115e-003
Ba	5.109e-009	5.109e-009
Ca	2.201e-004	2.201e-004
Cl	2.014e-003	2.014e-003
Cu	9.937e-008	9.937e-008
F	5.381e-006	5.381e-006
K	3.819e-005	3.819e-005
Mg	1.563e-003	1.563e-003
Mn	1.897e-007	1.897e-007
Na	2.612e-003	2.612e-003
S(6)	2.107e-005	2.107e-005
Si	3.170e-002	3.170e-002
Zn	5.673e-007	5.673e-007

----------------------------Description of solution----------------------------

pH = 7.000
pe = 4.000
Activity of water = 0.999
Ionic strength = 7.867e-003
Mass of water (kg) = 1.000e+000
Total carbon (mol/kg) = 4.949e-003
Total CO2 (mol/kg) = 4.949e-003
Temperature (deg C) = 20.000
Electrical balance (eq) = 4.022e-005
Percent error, 100*(Cat-|An|)/(Cat+|An|) = 0.33
Iterations = 10
Total H = 1.111432e+002
Total O = 5.564705e+001

----------------------------Distribution of species----------------------------

Log   Log   Log

Species		Molality	Activity	Molality	Activity	Gamma
H+		1.085e-007	1.000e-007	-6.964	-7.000	-0.036
OH-		7.450e-008	6.784e-008	-7.128	-7.169	-0.041
H2O		5.551e+001	9.993e-001	1.744	-0.000	0.000
Ba	5.109e-009					
Ba+2		4.971e-009	3.467e-009	-8.304	-8.460	-0.156
BaHCO3+		1.140e-010	1.040e-010	-9.943	-9.983	-0.040
BaSO4		2.157e-011	2.161e-011	-10.666	-10.665	0.001
BaCO3		2.493e-012	2.498e-012	-11.603	-11.602	0.001
BaOH+		1.287e-015	1.174e-015	-14.890	-14.930	-0.040
C(4)	4.949e-003					
HCO3-		4.003e-003	3.661e-003	-2.398	-2.436	-0.039
CO2		8.811e-004	8.827e-004	-3.055	-3.054	0.001
MgHCO3+		4.887e-005	4.458e-005	-4.311	-4.351	-0.040
CaHCO3+		6.970e-006	6.376e-006	-5.157	-5.195	-0.039
NaHCO3		4.890e-006	4.899e-006	-5.311	-5.310	0.001
CO3-2		2.203e-006	1.542e-006	-5.657	-5.812	-0.155
MgCO3		1.443e-006	1.446e-006	-5.841	-5.840	0.001
CaCO3		3.507e-007	3.514e-007	-6.455	-6.454	0.001
ZnHCO3+		1.259e-007	1.148e-007	-6.900	-6.940	-0.040
ZnCO3		7.650e-008	7.664e-008	-7.116	-7.116	0.001
NaCO3-		5.796e-008	5.287e-008	-7.237	-7.277	-0.040
MnHCO3+		3.551e-008	3.239e-008	-7.450	-7.490	-0.040
MnCO3		1.214e-008	1.216e-008	-7.916	-7.915	0.001
Zn(CO3)2-2		3.651e-009	2.526e-009	-8.438	-8.597	-0.160
BaHCO3+		1.140e-010	1.040e-010	-9.943	-9.983	-0.040
BaCO3		2.493e-012	2.498e-012	-11.603	-11.602	0.001
Ca	2.201e-004					
Ca+2		2.124e-004	1.486e-004	-3.673	-3.828	-0.155
CaHCO3+		6.970e-006	6.376e-006	-5.157	-5.195	-0.039
CaSO4		3.510e-007	3.516e-007	-6.455	-6.454	0.001
CaCO3		3.507e-007	3.514e-007	-6.455	-6.454	0.001
CaF+		5.791e-009	5.282e-009	-8.237	-8.277	-0.040
CaOH+		2.702e-010	2.464e-010	-9.568	-9.608	-0.040
CaHSO4+		2.128e-013	1.941e-013	-12.672	-12.712	-0.040
Cl	2.014e-003					
Cl-		2.014e-003	1.835e-003	-2.696	-2.736	-0.041
ZnCl+		1.078e-009	9.830e-010	-8.967	-9.007	-0.040
MnCl+		8.134e-010	7.419e-010	-9.090	-9.130	-0.040
ZnCl2		1.847e-012	1.850e-012	-11.734	-11.733	0.001
MnCl2		5.931e-013	5.941e-013	-12.227	-12.226	0.001
ZnCl3-		4.050e-015	3.694e-015	-14.393	-14.432	-0.040
MnCl3-		3.291e-016	3.002e-016	-15.483	-15.523	-0.040
ZnCl4-2		4.714e-018	3.262e-018	-17.327	-17.486	-0.160
Cu(1)	1.492e-009					
Cu+		1.492e-009	1.355e-009	-8.826	-8.868	-0.042
Cu(2)	9.788e-008					
Cu(OH)2		5.638e-008	5.648e-008	-7.249	-7.248	0.001
Cu+2		3.847e-008	2.707e-008	-7.415	-7.567	-0.153
CuOH+		2.967e-009	2.705e-009	-8.528	-8.568	-0.040
CuSO4		6.625e-011	6.637e-011	-10.179	-10.178	0.001
Cu(OH)3-		3.728e-014	3.401e-014	-13.428	-13.468	-0.040
Cu(OH)4-2		9.797e-020	6.780e-020	-19.009	-19.169	-0.160
F	5.381e-006					
F-		5.047e-006	4.595e-006	-5.297	-5.338	-0.041
MgF+		3.222e-007	2.938e-007	-6.492	-6.532	-0.040
NaF		6.281e-009	6.293e-009	-8.202	-8.201	0.001
CaF+		5.791e-009	5.282e-009	-8.237	-8.277	-0.040
HF		6.293e-010	6.305e-010	-9.201	-9.200	0.001
MnF+		3.460e-012	3.156e-012	-11.461	-11.501	-0.040
HF2-		1.169e-014	1.066e-014	-13.932	-13.972	-0.040
SiF6-2		1.046e-031	7.240e-032	-30.980	-31.140	-0.160
H(0)	1.487e-025					
H2		7.434e-026	7.447e-026	-25.129	-25.128	0.001
K	3.819e-005					
K+		3.819e-005	3.479e-005	-4.418	-4.459	-0.041

KSO4-		3.050e-009	2.782e-009	-8.516	-8.556	-0.040
KOH		1.203e-012	1.205e-012	-11.920	-11.919	0.001
Mg	1.563e-003					
Mg+2		1.509e-003	1.061e-003	-2.821	-2.974	-0.153
MgHCO3+		4.887e-005	4.458e-005	-4.311	-4.351	-0.040
MgSO4		2.709e-006	2.714e-006	-5.567	-5.566	0.001
MgCO3		1.443e-006	1.446e-006	-5.841	-5.840	0.001
MgF+		3.222e-007	2.938e-007	-6.492	-6.532	-0.040
MgOH+		2.667e-008	2.432e-008	-7.574	-7.614	-0.040
Mn(2)	1.897e-007					
Mn+2		1.411e-007	9.927e-008	-6.851	-7.003	-0.153
MnHCO3+		3.551e-008	3.239e-008	-7.450	-7.490	-0.040
MnCO3		1.214e-008	1.216e-008	-7.916	-7.915	0.001
MnCl+		8.134e-010	7.419e-010	-9.090	-9.130	-0.040
MnSO4		1.989e-010	1.992e-010	-9.701	-9.701	0.001
MnOH+		1.847e-011	1.684e-011	-10.734	-10.774	-0.040
MnF+		3.460e-012	3.156e-012	-11.461	-11.501	-0.040
MnCl2		5.931e-013	5.941e-013	-12.227	-12.226	0.001
MnCl3-		3.291e-016	3.002e-016	-15.483	-15.523	-0.040
Mn(3)	3.341e-029					
Mn+3		3.341e-029	1.460e-029	-28.476	-28.836	-0.360
Na	2.612e-003					
Na+		2.606e-003	2.380e-003	-2.584	-2.623	-0.040
NaHCO3		4.890e-006	4.899e-006	-5.311	-5.310	0.001
NaSO4-		1.575e-007	1.436e-007	-6.803	-6.843	-0.040
NaCO3-		5.796e-008	5.287e-008	-7.237	-7.277	-0.040
NaF		6.281e-009	6.293e-009	-8.202	-8.201	0.001
NaOH		1.568e-010	1.571e-010	-9.805	-9.804	0.001
O(0)	0.000e+000					
O2		0.000e+000	0.000e+000	-43.767	-43.766	0.001
S(6)	2.107e-005					
SO4-2		1.784e-005	1.244e-005	-4.748	-4.905	-0.157
MgSO4		2.709e-006	2.714e-006	-5.567	-5.566	0.001
CaSO4		3.510e-007	3.516e-007	-6.455	-6.454	0.001
NaSO4-		1.575e-007	1.436e-007	-6.803	-6.843	-0.040
KSO4-		3.050e-009	2.782e-009	-8.516	-8.556	-0.040
ZnSO4		6.971e-010	6.984e-010	-9.157	-9.156	0.001
MnSO4		1.989e-010	1.992e-010	-9.701	-9.701	0.001
HSO4-		1.191e-010	1.087e-010	-9.924	-9.964	-0.040
CuSO4		6.625e-011	6.637e-011	-10.179	-10.178	0.001
BaSO4		2.157e-011	2.161e-011	-10.666	-10.665	0.001
CaHSO4+		2.128e-013	1.941e-013	-12.672	-12.712	-0.040
Zn(SO4)2-2		1.061e-013	7.342e-014	-12.974	-13.134	-0.160
Si	3.170e-002					
H4SiO4		3.165e-002	3.171e-002	-1.500	-1.499	0.001
H3SiO4-		4.283e-005	3.907e-005	-4.368	-4.408	-0.040
H2SiO4-2		2.746e-011	1.900e-011	-10.561	-10.721	-0.160
SiF6-2		1.046e-031	7.240e-032	-30.980	-31.140	-0.160
Zn	5.673e-007					
Zn+2		3.572e-007	2.491e-007	-6.447	-6.604	-0.156
ZnHCO3+		1.259e-007	1.148e-007	-6.900	-6.940	-0.040
ZnCO3		7.650e-008	7.664e-008	-7.116	-7.116	0.001
Zn(CO3)2-2		3.651e-009	2.526e-009	-8.438	-8.597	-0.160
ZnOH+		2.035e-009	1.856e-009	-8.691	-8.731	-0.040
ZnCl+		1.078e-009	9.830e-010	-8.967	-9.007	-0.040
ZnSO4		6.971e-010	6.984e-010	-9.157	-9.156	0.001
Zn(OH)2		3.126e-010	3.132e-010	-9.505	-9.504	0.001
ZnCl2		1.847e-012	1.850e-012	-11.734	-11.733	0.001
Zn(SO4)2-2		1.061e-013	7.342e-014	-12.974	-13.134	-0.160
Zn(OH)3-		1.085e-014	9.896e-015	-13.965	-14.005	-0.040
ZnCl3-		4.050e-015	3.694e-015	-14.393	-14.432	-0.040
ZnCl4-2		4.714e-018	3.262e-018	-17.327	-17.486	-0.160
Zn(OH)4-2		2.264e-020	1.567e-020	-19.645	-19.805	-0.160

------------------------------Saturation indices-------------------------------

Phase	SI	log IAP	log KT

Anhydrite	-4.39	-8.73	-4.34	CaSO4
Aragonite	-1.33	-9.64	-8.31	CaCO3
Barite	-3.31	-13.37	-10.05	BaSO4
Calcite	-1.19	-9.64	-8.45	CaCO3
Chalcedony	2.11	-1.50	-3.61	SiO2
Chrysotile	-2.75	30.08	32.83	Mg3Si2O5(OH)4
CO2(g)	-1.65	-3.05	-1.41	CO2
Dolomite	-1.45	-18.43	-16.97	CaMg(CO3)2
Fluorite	-3.84	-14.50	-10.66	CaF2
Gypsum	-4.15	-8.73	-4.58	CaSO4:2H2O
H2(g)	-22.00	-25.13	-3.13	H2
H2O(g)	-1.64	-0.00	1.64	H2O
Halite	-6.93	-5.36	1.57	NaCl
Hausmannite	-19.30	42.99	62.29	Mn3O4
Manganite	-7.34	18.00	25.34	MnOOH
O2(g)	-40.83	-43.77	-2.94	O2
Pyrochroite	-8.20	7.00	15.20	Mn(OH)2
Pyrolusite	-13.20	29.00	42.19	MnO2
Quartz	2.56	-1.50	-4.06	SiO2
Rhodochrosite	-1.70	-12.82	-11.11	MnCO3
Sepiolite	1.66	17.56	15.89	Mg2Si3O7.5OH:3H2O
Sepiolite(d)	-1.10	17.56	18.66	Mg2Si3O7.5OH:3H2O
SiO2(a)	1.26	-1.50	-2.75	SiO2
Smithsonite	-2.47	-12.42	-9.95	ZnCO3
Talc	5.10	27.08	21.98	Mg3Si4O10(OH)2
Willemite	-2.45	13.29	15.75	Zn2SiO4
Witherite	-5.70	-14.27	-8.57	BaCO3
Zn(OH)2(e)	-4.10	7.40	11.50	Zn(OH)2

Initial solution 10.   4LSoledad-1

-----------------------------Solution composition------------------------------

Elements	Molality	Moles
Alkalinity	4.644e-004	4.644e-004
Ba	1.314e-008	1.314e-008
Ca	8.006e-005	8.006e-005
Cl	7.774e-004	7.774e-004
Cu	4.261e-008	4.261e-008
F	4.170e-006	4.170e-006
K	1.923e-005	1.923e-005
Mg	2.029e-004	2.029e-004
Mn	2.172e-007	2.172e-007
Na	7.633e-004	7.633e-004
S(6)	4.374e-005	4.374e-005
Si	4.506e-002	4.506e-002
Zn	1.672e-007	1.672e-007

----------------------------Description of solution----------------------------

pH	=	7.000
pe	=	4.000
Activity of water	=	0.999
Ionic strength	=	1.660e-003
Mass of water (kg)	=	1.000e+000
Total carbon (mol/kg)	=	5.175e-004
Total CO2 (mol/kg)	=	5.175e-004
Temperature (deg C)	=	15.700
Electrical balance (eq)	=	1.598e-005
Percent error, 100*(Cat-|An|)/(Cat+|An|)	=	0.60
Iterations	=	10
Total H	=	1.111930e+002
Total O	=	5.568809e+001

----------------------------Distribution of species----------------------------

Species		Molality	Activity	Log Molality	Log Activity	Log Gamma
H+		1.043e-007	1.000e-007	-6.982	-7.000	-0.018
OH-		4.996e-008	4.776e-008	-7.301	-7.321	-0.020
H2O		5.551e+001	9.992e-001	1.744	-0.000	0.000
Ba	1.314e-008					
Ba+2		1.292e-008	1.083e-008	-7.889	-7.965	-0.077
BaSO4		1.913e-010	1.914e-010	-9.718	-9.718	0.000
BaHCO3+		3.205e-011	3.065e-011	-10.494	-10.514	-0.019
BaCO3		6.970e-013	6.972e-013	-12.157	-12.157	0.000
BaOH+		3.834e-015	3.666e-015	-14.416	-14.436	-0.019
C(4)	5.175e-004					
HCO3-		4.135e-004	3.957e-004	-3.384	-3.403	-0.019
CO2		1.025e-004	1.026e-004	-3.989	-3.989	0.000
MgHCO3+		7.914e-007	7.569e-007	-6.102	-6.121	-0.019
CaHCO3+		2.951e-007	2.824e-007	-6.530	-6.549	-0.019
CO3-2		1.791e-007	1.502e-007	-6.747	-6.823	-0.076
NaHCO3		1.624e-007	1.624e-007	-6.789	-6.789	0.000
MgCO3		2.097e-008	2.098e-008	-7.678	-7.678	0.000
CaCO3		1.441e-008	1.441e-008	-7.841	-7.841	0.000
ZnHCO3+		6.747e-009	6.452e-009	-8.171	-8.190	-0.019
MnHCO3+		6.420e-009	6.140e-009	-8.192	-8.212	-0.019
ZnCO3		3.881e-009	3.882e-009	-8.411	-8.411	0.000
MnCO3		2.077e-009	2.077e-009	-8.683	-8.682	0.000
NaCO3-		1.316e-009	1.258e-009	-8.881	-8.900	-0.019
BaHCO3+		3.205e-011	3.065e-011	-10.494	-10.514	-0.019
Zn(CO3)2-2		1.491e-011	1.247e-011	-10.827	-10.904	-0.078
BaCO3		6.970e-013	6.972e-013	-12.157	-12.157	0.000
Ca	8.006e-005					
Ca+2		7.932e-005	6.653e-005	-4.101	-4.177	-0.076
CaSO4		4.279e-007	4.281e-007	-6.369	-6.368	0.000
CaHCO3+		2.951e-007	2.824e-007	-6.530	-6.549	-0.019
CaCO3		1.441e-008	1.441e-008	-7.841	-7.841	0.000
CaF+		1.911e-009	1.828e-009	-8.719	-8.738	-0.019
CaOH+		1.154e-010	1.103e-010	-9.938	-9.957	-0.019
CaHSO4+		2.360e-013	2.257e-013	-12.627	-12.646	-0.019
Cl	7.774e-004					
Cl-		7.774e-004	7.432e-004	-3.109	-3.129	-0.020
MnCl+		5.511e-010	5.271e-010	-9.259	-9.278	-0.019
ZnCl+		1.774e-010	1.697e-010	-9.751	-9.770	-0.019
MnCl2		1.709e-013	1.710e-013	-12.767	-12.767	0.000
ZnCl2		1.270e-013	1.271e-013	-12.896	-12.896	0.000
ZnCl3-		1.046e-016	1.000e-016	-15.981	-16.000	-0.019
MnCl3-		3.660e-017	3.501e-017	-16.436	-16.456	-0.019
ZnCl4-2		4.128e-020	3.453e-020	-19.384	-19.462	-0.078
Cu(1)	6.225e-010					
Cu+		6.225e-010	5.948e-010	-9.206	-9.226	-0.020
Cu(2)	4.199e-008					
Cu(OH)2		2.585e-008	2.586e-008	-7.588	-7.587	0.000
Cu+2		1.476e-008	1.240e-008	-7.831	-7.907	-0.076
CuOH+		1.295e-009	1.239e-009	-8.888	-8.907	-0.019
CuSO4		8.352e-011	8.355e-011	-10.078	-10.078	0.000
Cu(OH)3-		1.628e-014	1.557e-014	-13.788	-13.808	-0.019
Cu(OH)4-2		3.711e-020	3.104e-020	-19.431	-19.508	-0.078
F	4.170e-006					
F-		4.127e-006	3.946e-006	-5.384	-5.404	-0.020
MgF+		3.866e-008	3.698e-008	-7.413	-7.432	-0.019
CaF+		1.911e-009	1.828e-009	-8.719	-8.738	-0.019
NaF		1.657e-009	1.657e-009	-8.781	-8.781	0.000
HF		5.020e-010	5.022e-010	-9.299	-9.299	0.000
MnF+		4.969e-012	4.752e-012	-11.304	-11.323	-0.019
HF2-		7.316e-015	6.996e-015	-14.136	-14.155	-0.019
SiF6-2		7.463e-032	6.242e-032	-31.127	-31.205	-0.078
H(0)	1.557e-025					
H2		7.787e-026	7.790e-026	-25.109	-25.108	0.000

K	1.923e-005					
K+		1.923e-005	1.839e-005	-4.716	-4.736	-0.020
KSO4-		4.030e-009	3.854e-009	-8.395	-8.414	-0.019
KOH		6.367e-013	6.370e-013	-12.196	-12.196	0.000
Mg	2.029e-004					
Mg+2		2.010e-004	1.688e-004	-3.697	-3.773	-0.076
MgSO4		1.089e-006	1.090e-006	-5.963	-5.963	0.000
MgHCO3+		7.914e-007	7.569e-007	-6.102	-6.121	-0.019
MgF+		3.866e-008	3.698e-008	-7.413	-7.432	-0.019
MgCO3		2.097e-008	2.098e-008	-7.678	-7.678	0.000
MgOH+		2.691e-009	2.573e-009	-8.570	-8.589	-0.019
Mn(2)	2.172e-007					
Mn+2		2.072e-007	1.741e-007	-6.684	-6.759	-0.076
MnHCO3+		6.420e-009	6.140e-009	-8.192	-8.212	-0.019
MnCO3		2.077e-009	2.077e-009	-8.683	-8.682	0.000
MnSO4		9.088e-010	9.092e-010	-9.042	-9.041	0.000
MnCl+		5.511e-010	5.271e-010	-9.259	-9.278	-0.019
MnOH+		2.138e-011	2.044e-011	-10.670	-10.689	-0.019
MnF+		4.969e-012	4.752e-012	-11.304	-11.323	-0.019
MnCl2		1.709e-013	1.710e-013	-12.767	-12.767	0.000
MnCl3-		3.660e-017	3.501e-017	-16.436	-16.456	-0.019
Mn(3)	1.979e-029					
Mn+3		1.979e-029	1.324e-029	-28.704	-28.878	-0.175
Na	7.633e-004					
Na+		7.630e-004	7.299e-004	-3.117	-3.137	-0.019
NaHCO3		1.624e-007	1.624e-007	-6.789	-6.789	0.000
NaSO4-		1.270e-007	1.214e-007	-6.896	-6.916	-0.019
NaF		1.657e-009	1.657e-009	-8.781	-8.781	0.000
NaCO3-		1.316e-009	1.258e-009	-8.881	-8.900	-0.019
NaOH		4.817e-011	4.819e-011	-10.317	-10.317	0.000
O(0)	0.000e+000					
O2		0.000e+000	0.000e+000	-45.262	-45.262	0.000
S(6)	4.374e-005					
SO4-2		4.209e-005	3.527e-005	-4.376	-4.453	-0.077
MgSO4		1.089e-006	1.090e-006	-5.963	-5.963	0.000
CaSO4		4.279e-007	4.281e-007	-6.369	-6.368	0.000
NaSO4-		1.270e-007	1.214e-007	-6.896	-6.916	-0.019
KSO4-		4.030e-009	3.854e-009	-8.395	-8.414	-0.019
ZnSO4		9.942e-010	9.946e-010	-9.003	-9.002	0.000
MnSO4		9.088e-010	9.092e-010	-9.042	-9.041	0.000
HSO4-		2.951e-010	2.822e-010	-9.530	-9.549	-0.019
BaSO4		1.913e-010	1.914e-010	-9.718	-9.718	0.000
CuSO4		8.352e-011	8.355e-011	-10.078	-10.078	0.000
Zn(SO4)2-2		3.671e-013	3.070e-013	-12.435	-12.513	-0.078
CaHSO4+		2.360e-013	2.257e-013	-12.627	-12.646	-0.019
Si	4.506e-002					
H4SiO4		4.501e-002	4.503e-002	-1.347	-1.346	0.000
H3SiO4-		4.922e-005	4.707e-005	-4.308	-4.327	-0.019
H2SiO4-2		2.018e-011	1.688e-011	-10.695	-10.773	-0.078
SiF6-2		7.463e-032	6.242e-032	-31.127	-31.205	-0.078
Zn	1.672e-007					
Zn+2		1.545e-007	1.295e-007	-6.811	-6.888	-0.077
ZnHCO3+		6.747e-009	6.452e-009	-8.171	-8.190	-0.019
ZnCO3		3.881e-009	3.882e-009	-8.411	-8.411	0.000
ZnSO4		9.942e-010	9.946e-010	-9.003	-9.002	0.000
ZnOH+		7.163e-010	6.851e-010	-9.145	-9.164	-0.019
ZnCl+		1.774e-010	1.697e-010	-9.751	-9.770	-0.019
Zn(OH)2		1.627e-010	1.628e-010	-9.789	-9.788	0.000
Zn(CO3)2-2		1.491e-011	1.247e-011	-10.827	-10.904	-0.078
Zn(SO4)2-2		3.671e-013	3.070e-013	-12.435	-12.513	-0.078
ZnCl2		1.270e-013	1.271e-013	-12.896	-12.896	0.000
Zn(OH)3-		5.378e-015	5.144e-015	-14.269	-14.289	-0.019
ZnCl3-		1.046e-016	1.000e-016	-15.981	-16.000	-0.019
ZnCl4-2		4.128e-020	3.453e-020	-19.384	-19.462	-0.078
Zn(OH)4-2		9.738e-021	8.145e-021	-20.012	-20.089	-0.078

------------------------------Saturation indices------------------------------

Phase	SI	log IAP	log KT	
Anhydrite	-4.29	-8.63	-4.34	CaSO4
Aragonite	-2.72	-11.00	-8.28	CaCO3
Barite	-2.29	-12.42	-10.13	BaSO4
Calcite	-2.57	-11.00	-8.43	CaCO3
Chalcedony	2.32	-1.35	-3.66	SiO2
Chrysotile	-5.40	27.99	33.39	Mg3Si2O5(OH)4
CO2(g)	-2.64	-3.99	-1.35	CO2
Dolomite	-4.73	-21.60	-16.87	CaMg(CO3)2
Fluorite	-4.27	-14.98	-10.72	CaF2
Gypsum	-4.05	-8.63	-4.58	CaSO4:2H2O
H2(g)	-22.00	-25.11	-3.11	H2
H2O(g)	-1.76	-0.00	1.76	H2O
Halite	-7.83	-6.27	1.56	NaCl
Hausmannite	-19.68	43.72	63.41	Mn3O4
Manganite	-7.10	18.24	25.34	MnOOH
O2(g)	-42.35	-45.26	-2.92	O2
Pyrochroite	-7.96	7.24	15.20	Mn(OH)2
Pyrolusite	-13.68	29.24	42.92	MnO2
Quartz	2.78	-1.35	-4.12	SiO2
Rhodochrosite	-2.49	-13.58	-11.10	MnCO3
Sepiolite	0.40	16.42	16.01	Mg2Si3O7.5OH:3H2O
Sepiolite(d)	-2.24	16.42	18.66	Mg2Si3O7.5OH:3H2O
SiO2(a)	1.44	-1.35	-2.79	SiO2
Smithsonite	-3.81	-13.71	-9.90	ZnCO3
Talc	2.80	25.30	22.49	Mg3Si4O10(OH)2
Willemite	-3.24	12.88	16.12	Zn2SiO4
Witherite	-6.19	-14.79	-8.59	BaCO3
Zn(OH)2(e)	-4.39	7.11	11.50	Zn(OH)2

Initial solution 11.   4LTamara-1

-----------------------------Solution composition------------------------------

Elements	Molality	Moles
Alkalinity	6.750e-004	6.750e-004
Ba	5.840e-009	5.840e-009
Ca	1.551e-004	1.551e-004
Cl	1.486e-003	1.486e-003
Cu	2.682e-008	2.682e-008
K	1.949e-005	1.949e-005
Mg	3.720e-004	3.720e-004
Mn	1.350e-007	1.350e-007
Na	1.269e-003	1.269e-003
S(6)	8.130e-005	8.130e-005
Si	4.005e-002	4.005e-002
Zn	9.662e-008	9.662e-008

----------------------------Description of solution----------------------------

pH	=	7.000
pe	=	4.000
Activity of water	=	0.999
Ionic strength	=	2.919e-003
Mass of water (kg)	=	1.000e+000
Total carbon (mol/kg)	=	8.226e-004
Total CO2 (mol/kg)	=	8.226e-004
Temperature (deg C)	=	8.600
Electrical balance (eq)	=	2.004e-005
Percent error, 100*(Cat-|An|)/(Cat+|An|)	=	0.43
Iterations	=	9
Total H	=	1.111732e+002
Total O	=	5.566901e+001

---------------------------Distribution of species---------------------------

Species		Molality	Activity	Log Molality	Log Activity	Log Gamma
H+		1.055e-007	1.000e-007	-6.977	-7.000	-0.023
OH-		2.733e-008	2.579e-008	-7.563	-7.589	-0.025
H2O		5.551e+001	9.992e-001	1.744	-0.000	0.000
Ba	5.840e-009					
Ba+2		5.684e-009	4.529e-009	-8.245	-8.344	-0.099
BaSO4		1.391e-010	1.392e-010	-9.857	-9.856	0.000
BaHCO3+		1.655e-011	1.562e-011	-10.781	-10.806	-0.025
BaCO3		3.166e-013	3.168e-013	-12.500	-12.499	0.000
BaOH+		1.624e-015	1.533e-015	-14.789	-14.814	-0.025
C(4)	8.226e-004					
HCO3-		6.381e-004	6.031e-004	-3.195	-3.220	-0.024
CO2		1.810e-004	1.811e-004	-3.742	-3.742	0.000
MgHCO3+		2.101e-006	1.983e-006	-5.678	-5.703	-0.025
CaHCO3+		6.953e-007	6.572e-007	-6.158	-6.182	-0.024
NaHCO3		4.059e-007	4.062e-007	-6.392	-6.391	0.000
CO3-2		2.358e-007	1.882e-007	-6.627	-6.725	-0.098
MgCO3		4.094e-008	4.097e-008	-7.388	-7.388	0.000
CaCO3		3.114e-008	3.116e-008	-7.507	-7.506	0.000
MnHCO3+		5.754e-009	5.432e-009	-8.240	-8.265	-0.025
ZnHCO3+		5.583e-009	5.270e-009	-8.253	-8.278	-0.025
ZnCO3		2.605e-009	2.606e-009	-8.584	-8.584	0.000
NaCO3-		1.853e-009	1.749e-009	-8.732	-8.757	-0.025
MnCO3		1.510e-009	1.511e-009	-8.821	-8.821	0.000
BaHCO3+		1.655e-011	1.562e-011	-10.781	-10.806	-0.025
Zn(CO3)2-2		1.320e-011	1.049e-011	-10.879	-10.979	-0.100
BaCO3		3.166e-013	3.168e-013	-12.500	-12.499	0.000
Ca	1.551e-004					
Ca+2		1.531e-004	1.221e-004	-3.815	-3.913	-0.098
CaSO4		1.270e-006	1.271e-006	-5.896	-5.896	0.000
CaHCO3+		6.953e-007	6.572e-007	-6.158	-6.182	-0.024
CaCO3		3.114e-008	3.116e-008	-7.507	-7.506	0.000
CaOH+		2.145e-010	2.025e-010	-9.669	-9.694	-0.025
CaHSO4+		6.653e-013	6.280e-013	-12.177	-12.202	-0.025
Cl	1.486e-003					
Cl-		1.486e-003	1.402e-003	-2.828	-2.853	-0.025
MnCl+		6.113e-010	5.771e-010	-9.214	-9.239	-0.025
ZnCl+		1.291e-010	1.218e-010	-9.889	-9.914	-0.025
MnCl2		3.528e-013	3.531e-013	-12.452	-12.452	0.000
ZnCl2		1.667e-013	1.668e-013	-12.778	-12.778	0.000
ZnCl3-		2.503e-016	2.363e-016	-15.601	-15.626	-0.025
MnCl3-		1.444e-016	1.363e-016	-15.840	-15.865	-0.025
ZnCl4-2		1.822e-019	1.447e-019	-18.739	-18.840	-0.100
Cu(1)	3.629e-010					
Cu+		3.629e-010	3.420e-010	-9.440	-9.466	-0.026
Cu(2)	2.646e-008					
Cu(OH)2		1.598e-008	1.599e-008	-7.796	-7.796	0.000
Cu+2		9.584e-009	7.664e-009	-8.018	-8.116	-0.097
CuOH+		8.113e-010	7.658e-010	-9.091	-9.116	-0.025
CuSO4		8.506e-011	8.512e-011	-10.070	-10.070	0.000
Cu(OH)3-		1.020e-014	9.627e-015	-13.991	-14.017	-0.025
Cu(OH)4-2		2.417e-020	1.919e-020	-19.617	-19.717	-0.100
H(0)	1.682e-025					
H2		8.409e-026	8.415e-026	-25.075	-25.075	0.000
K	1.949e-005					
K+		1.948e-005	1.838e-005	-4.710	-4.736	-0.025
KSO4-		6.197e-009	5.850e-009	-8.208	-8.233	-0.025
KOH		6.364e-013	6.368e-013	-12.196	-12.196	0.000
Mg	3.720e-004					
Mg+2		3.671e-004	2.935e-004	-3.435	-3.532	-0.097
MgSO4		2.696e-006	2.698e-006	-5.569	-5.569	0.000
MgHCO3+		2.101e-006	1.983e-006	-5.678	-5.703	-0.025
MgCO3		4.094e-008	4.097e-008	-7.388	-7.388	0.000

	MgOH+		2.353e-009	2.221e-009	-8.628	-8.653	-0.025
Mn(2)		1.350e-007					
	Mn+2		1.264e-007	1.011e-007	-6.898	-6.995	-0.097
	MnHCO3+		5.754e-009	5.432e-009	-8.240	-8.265	-0.025
	MnCO3		1.510e-009	1.511e-009	-8.821	-8.821	0.000
	MnSO4		7.909e-010	7.914e-010	-9.102	-9.102	0.000
	MnCl+		6.113e-010	5.771e-010	-9.214	-9.239	-0.025
	MnOH+		6.682e-012	6.308e-012	-11.175	-11.200	-0.025
	MnCl2		3.528e-013	3.531e-013	-12.452	-12.452	0.000
	MnCl3-		1.444e-016	1.363e-016	-15.840	-15.865	-0.025
Mn(3)		4.159e-030					
	Mn+3		4.159e-030	2.476e-030	-29.381	-29.606	-0.225
Na		1.269e-003					
	Na+		1.268e-003	1.198e-003	-2.897	-2.922	-0.025
	NaHCO3		4.059e-007	4.062e-007	-6.392	-6.391	0.000
	NaSO4-		3.493e-007	3.297e-007	-6.457	-6.482	-0.025
	NaCO3-		1.853e-009	1.749e-009	-8.732	-8.757	-0.025
	NaOH		7.902e-011	7.907e-011	-10.102	-10.102	0.000
O(0)		0.000e+000					
	O2		0.000e+000	0.000e+000	-47.832	-47.832	0.000
S(6)		8.130e-005					
	SO4-2		7.698e-005	6.132e-005	-4.114	-4.212	-0.099
	MgSO4		2.696e-006	2.698e-006	-5.569	-5.569	0.000
	CaSO4		1.270e-006	1.271e-006	-5.896	-5.896	0.000
	NaSO4-		3.493e-007	3.297e-007	-6.457	-6.482	-0.025
	KSO4-		6.197e-009	5.850e-009	-8.208	-8.233	-0.025
	ZnSO4		8.724e-010	8.730e-010	-9.059	-9.059	0.000
	MnSO4		7.909e-010	7.914e-010	-9.102	-9.102	0.000
	HSO4-		4.531e-010	4.277e-010	-9.344	-9.369	-0.025
	BaSO4		1.391e-010	1.392e-010	-9.857	-9.856	0.000
	CuSO4		8.506e-011	8.512e-011	-10.070	-10.070	0.000
	CaHSO4+		6.653e-013	6.280e-013	-12.177	-12.202	-0.025
	Zn(SO4)2-2		6.262e-013	4.973e-013	-12.203	-12.303	-0.100
Si		4.005e-002					
	H4SiO4		4.001e-002	4.004e-002	-1.398	-1.398	0.000
	H3SiO4-		3.308e-005	3.123e-005	-4.480	-4.505	-0.025
	H2SiO4-2		8.233e-012	6.539e-012	-11.084	-11.184	-0.100
Zn		9.662e-008					
	Zn+2		8.711e-008	6.942e-008	-7.060	-7.159	-0.099
	ZnHCO3+		5.583e-009	5.270e-009	-8.253	-8.278	-0.025
	ZnCO3		2.605e-009	2.606e-009	-8.584	-8.584	0.000
	ZnSO4		8.724e-010	8.730e-010	-9.059	-9.059	0.000
	ZnOH+		2.160e-010	2.039e-010	-9.666	-9.691	-0.025
	ZnCl+		1.291e-010	1.218e-010	-9.889	-9.914	-0.025
	Zn(OH)2		8.720e-011	8.726e-011	-10.059	-10.059	0.000
	Zn(CO3)2-2		1.320e-011	1.049e-011	-10.879	-10.979	-0.100
	Zn(SO4)2-2		6.262e-013	4.973e-013	-12.203	-12.303	-0.100
	ZnCl2		1.667e-013	1.668e-013	-12.778	-12.778	0.000
	Zn(OH)3-		2.921e-015	2.757e-015	-14.535	-14.560	-0.025
	ZnCl3-		2.503e-016	2.363e-016	-15.601	-15.626	-0.025
	ZnCl4-2		1.822e-019	1.447e-019	-18.739	-18.840	-0.100
	Zn(OH)4-2		5.498e-021	4.367e-021	-20.260	-20.360	-0.100

------------------------------Saturation indices-------------------------------

Phase	SI	log IAP	log KT	
Anhydrite	-3.79	-8.13	-4.34	CaSO4
Aragonite	-2.39	-10.64	-8.25	CaCO3
Barite	-2.28	-12.56	-10.28	BaSO4
Calcite	-2.23	-10.64	-8.41	CaCO3
Chalcedony	2.36	-1.40	-3.75	SiO2
Chrysotile	-5.74	28.61	34.35	Mg3Si2O5(OH)4
CO2(g)	-2.49	-3.74	-1.25	CO2
Dolomite	-4.21	-20.90	-16.69	CaMg(CO3)2
Gypsum	-3.53	-8.13	-4.59	CaSO4:2H2O
H2(g)	-22.00	-25.07	-3.07	H2

H2O(g)	-1.96	-0.00	1.96	H2O
Halite	-7.32	-5.77	1.54	NaCl
Hausmannite	-22.31	43.01	65.32	Mn3O4
Manganite	-7.34	18.00	25.34	MnOOH
O2(g)	-44.95	-47.83	-2.88	O2
Pyrochroite	-8.20	7.00	15.20	Mn(OH)2
Pyrolusite	-15.15	29.00	44.16	MnO2
Quartz	2.84	-1.40	-4.24	SiO2
Rhodochrosite	-2.65	-13.72	-11.07	MnCO3
Sepiolite	0.53	16.74	16.22	Mg2Si3O7.5OH:3H2O
Sepiolite(d)	-1.92	16.74	18.66	Mg2Si3O7.5OH:3H2O
SiO2(a)	1.46	-1.40	-2.85	SiO2
Smithsonite	-4.07	-13.88	-9.81	ZnCO3
Talc	2.44	25.81	23.38	Mg3Si4O10(OH)2
Willemite	-4.47	12.29	16.75	Zn2SiO4
Witherite	-6.43	-15.07	-8.64	BaCO3
Zn(OH)2(e)	-4.66	6.84	11.50	Zn(OH)2

Initial solution 12.   4LAdela-1

----------------------------Solution composition-----------------------------

Elements	Molality	Moles
Alkalinity	3.400e-003	3.400e-003
Ba	2.049e-008	2.049e-008
Ca	1.956e-004	1.956e-004
Cl	5.917e-004	5.917e-004
Cu	8.699e-008	8.699e-008
F	5.079e-006	5.079e-006
K	3.342e-005	3.342e-005
Mg	5.250e-004	5.250e-004
Mn	3.183e-007	3.183e-007
Na	1.504e-003	1.504e-003
S(6)	7.900e-005	7.900e-005
Si	8.030e-002	8.030e-002
Zn	1.338e-007	1.338e-007

----------------------------Description of solution----------------------------

pH	= 7.000
pe	= 4.000
Activity of water	= 0.999
Ionic strength	= 4.303e-003
Mass of water (kg)	= 1.000e+000
Total carbon (mol/kg)	= 4.106e-003
Total CO2 (mol/kg)	= 4.106e-003
Temperature (deg C)	= 15.500
Electrical balance (eq)	= -1.175e-003
Percent error, 100*(Cat-|An|)/(Cat+|An|)	= -16.63
Iterations	= 10
Total H	= 1.113368e+002
Total O	= 5.583924e+001

----------------------------Distribution of species----------------------------

Species		Molality	Activity	Log Molality	Log Activity	Log Gamma
H+		1.066e-007	1.000e-007	-6.972	-7.000	-0.028
OH-		5.036e-008	4.694e-008	-7.298	-7.328	-0.031
H2O		5.551e+001	9.985e-001	1.744	-0.001	0.000
Ba	2.049e-008					
Ba+2		1.972e-008	1.500e-008	-7.705	-7.824	-0.119
BaSO4		4.179e-010	4.183e-010	-9.379	-9.378	0.000
BaHCO3+		3.507e-010	3.271e-010	-9.455	-9.485	-0.030
BaCO3		7.412e-012	7.420e-012	-11.130	-11.130	0.000

BaOH+		5.439e-015	5.073e-015	-14.264	-14.295	-0.030
C(4)	4.106e-003					
HCO3-		3.284e-003	3.069e-003	-2.484	-2.513	-0.029
CO2		7.979e-004	7.987e-004	-3.098	-3.098	0.000
MgHCO3+		1.441e-005	1.344e-005	-4.841	-4.871	-0.030
CaHCO3+		5.046e-006	4.715e-006	-5.297	-5.327	-0.029
NaHCO3		2.415e-006	2.418e-006	-5.617	-5.617	0.000
CO3-2		1.521e-006	1.159e-006	-5.818	-5.936	-0.118
MgCO3		3.694e-007	3.697e-007	-6.433	-6.432	0.000
CaCO3		2.397e-007	2.399e-007	-6.620	-6.620	0.000
MnHCO3+		5.478e-008	5.110e-008	-7.261	-7.292	-0.030
ZnHCO3+		2.788e-008	2.600e-008	-7.555	-7.585	-0.030
NaCO3-		1.976e-008	1.843e-008	-7.704	-7.734	-0.030
MnCO3		1.718e-008	1.720e-008	-7.765	-7.764	0.000
ZnCO3		1.555e-008	1.557e-008	-7.808	-7.808	0.000
Zn(CO3)2-2		5.095e-010	3.857e-010	-9.293	-9.414	-0.121
BaHCO3+		3.507e-010	3.271e-010	-9.455	-9.485	-0.030
BaCO3		7.412e-012	7.420e-012	-11.130	-11.130	0.000
Ca	1.956e-004					
Ca+2		1.889e-004	1.439e-004	-3.724	-3.842	-0.118
CaHCO3+		5.046e-006	4.715e-006	-5.297	-5.327	-0.029
CaSO4		1.457e-006	1.458e-006	-5.837	-5.836	0.000
CaCO3		2.397e-007	2.399e-007	-6.620	-6.620	0.000
CaF+		4.943e-009	4.611e-009	-8.306	-8.336	-0.030
CaOH+		2.556e-010	2.384e-010	-9.592	-9.623	-0.030
CaHSO4+		8.226e-013	7.673e-013	-12.085	-12.115	-0.030
Cl	5.917e-004					
Cl-		5.917e-004	5.515e-004	-3.228	-3.258	-0.031
MnCl+		4.500e-010	4.197e-010	-9.347	-9.377	-0.030
ZnCl+		6.948e-011	6.481e-011	-10.158	-10.188	-0.030
MnCl2		1.009e-013	1.010e-013	-12.996	-12.996	0.000
ZnCl2		3.594e-014	3.598e-014	-13.444	-13.444	0.000
ZnCl3-		2.250e-017	2.099e-017	-16.648	-16.678	-0.030
MnCl3-		1.645e-017	1.535e-017	-16.784	-16.814	-0.030
ZnCl4-2		7.090e-021	5.367e-021	-20.149	-20.270	-0.121
Cu(1)	1.258e-009					
Cu+		1.258e-009	1.171e-009	-8.900	-8.932	-0.031
Cu(2)	8.574e-008					
Cu(OH)2		5.088e-008	5.093e-008	-7.293	-7.293	0.000
Cu+2		3.198e-008	2.445e-008	-7.495	-7.612	-0.117
CuOH+		2.618e-009	2.441e-009	-8.582	-8.612	-0.030
CuSO4		2.594e-010	2.597e-010	-9.586	-9.586	0.000
Cu(OH)3-		3.285e-014	3.064e-014	-13.483	-13.514	-0.030
Cu(OH)4-2		8.065e-020	6.105e-020	-19.093	-19.214	-0.121
F	5.079e-006					
F-		4.964e-006	4.626e-006	-5.304	-5.335	-0.031
MgF+		1.061e-007	9.897e-008	-6.974	-7.005	-0.030
CaF+		4.943e-009	4.611e-009	-8.306	-8.336	-0.030
NaF		3.726e-009	3.729e-009	-8.429	-8.428	0.000
HF		5.861e-010	5.867e-010	-9.232	-9.232	0.000
MnF+		6.410e-012	5.979e-012	-11.193	-11.223	-0.030
HF2-		1.025e-014	9.563e-015	-13.989	-14.019	-0.030
SiF6-2		3.904e-031	2.955e-031	-30.408	-30.529	-0.121
H(0)	1.560e-025					
H2		7.798e-026	7.806e-026	-25.108	-25.108	0.000
K	3.342e-005					
K+		3.341e-005	3.114e-005	-4.476	-4.507	-0.031
KSO4-		1.100e-008	1.026e-008	-7.959	-7.989	-0.030
KOH		1.077e-012	1.078e-012	-11.968	-11.967	0.000
Mg	5.250e-004					
Mg+2		5.062e-004	3.868e-004	-3.296	-3.413	-0.117
MgHCO3+		1.441e-005	1.344e-005	-4.841	-4.871	-0.030
MgSO4		3.916e-006	3.920e-006	-5.407	-5.407	0.000
MgCO3		3.694e-007	3.697e-007	-6.433	-6.432	0.000
MgF+		1.061e-007	9.897e-008	-6.974	-7.005	-0.030
MgOH+		6.198e-009	5.781e-009	-8.208	-8.238	-0.030
Mn(2)	3.183e-007					

Mn+2		2.444e-007	1.868e-007	-6.612	-6.729	-0.117
MnHCO3+		5.478e-008	5.110e-008	-7.261	-7.292	-0.030
MnCO3		1.718e-008	1.720e-008	-7.765	-7.764	0.000
MnSO4		1.532e-009	1.534e-009	-8.815	-8.814	0.000
MnCl+		4.500e-010	4.197e-010	-9.347	-9.377	-0.030
MnOH+		2.310e-011	2.155e-011	-10.636	-10.667	-0.030
MnF+		6.410e-012	5.979e-012	-11.193	-11.223	-0.030
MnCl2		1.009e-013	1.010e-013	-12.996	-12.996	0.000
MnCl3-		1.645e-017	1.535e-017	-16.784	-16.814	-0.030
Mn(3)	2.577e-029					
Mn+3		2.577e-029	1.377e-029	-28.589	-28.861	-0.272
Na	1.504e-003					
Na+		1.501e-003	1.401e-003	-2.824	-2.854	-0.030
NaHCO3		2.415e-006	2.418e-006	-5.617	-5.617	0.000
NaSO4-		3.937e-007	3.673e-007	-6.405	-6.435	-0.030
NaCO3-		1.976e-008	1.843e-008	-7.704	-7.734	-0.030
NaF		3.726e-009	3.729e-009	-8.429	-8.428	0.000
NaOH		9.234e-011	9.243e-011	-10.035	-10.034	0.000
O(0)	0.000e+000					
O2		0.000e+000	0.000e+000	-45.333	-45.333	0.000
S(6)	7.900e-005					
SO4-2		7.321e-005	5.566e-005	-4.135	-4.254	-0.119
MgSO4		3.916e-006	3.920e-006	-5.407	-5.407	0.000
CaSO4		1.457e-006	1.458e-006	-5.837	-5.836	0.000
NaSO4-		3.937e-007	3.673e-007	-6.405	-6.435	-0.030
KSO4-		1.100e-008	1.026e-008	-7.959	-7.989	-0.030
MnSO4		1.532e-009	1.534e-009	-8.815	-8.814	0.000
ZnSO4		8.135e-010	8.143e-010	-9.090	-9.089	0.000
HSO4-		4.755e-010	4.436e-010	-9.323	-9.353	-0.030
BaSO4		4.179e-010	4.183e-010	-9.379	-9.378	0.000
CuSO4		2.594e-010	2.597e-010	-9.586	-9.586	0.000
CaHSO4+		8.226e-013	7.673e-013	-12.085	-12.115	-0.030
Zn(SO4)2-2		5.249e-013	3.973e-013	-12.280	-12.401	-0.121
Si	8.030e-002					
H4SiO4		8.021e-002	8.029e-002	-1.096	-1.095	0.000
H3SiO4-		8.927e-005	8.327e-005	-4.049	-4.080	-0.030
H2SiO4-2		3.888e-011	2.943e-011	-10.410	-10.531	-0.121
SiF6-2		3.904e-031	2.955e-031	-30.408	-30.529	-0.121
Zn	1.338e-007					
Zn+2		8.849e-008	6.730e-008	-7.053	-7.172	-0.119
ZnHCO3+		2.788e-008	2.600e-008	-7.555	-7.585	-0.030
ZnCO3		1.555e-008	1.557e-008	-7.808	-7.808	0.000
ZnSO4		8.135e-010	8.143e-010	-9.090	-9.089	0.000
Zn(CO3)2-2		5.095e-010	3.857e-010	-9.293	-9.414	-0.121
ZnOH+		3.753e-010	3.501e-010	-9.426	-9.456	-0.030
Zn(OH)2		8.440e-011	8.448e-011	-10.074	-10.073	0.000
ZnCl+		6.948e-011	6.481e-011	-10.158	-10.188	-0.030
Zn(SO4)2-2		5.249e-013	3.973e-013	-12.280	-12.401	-0.121
ZnCl2		3.594e-014	3.598e-014	-13.444	-13.444	0.000
Zn(OH)3-		2.860e-015	2.667e-015	-14.544	-14.574	-0.030
ZnCl3-		2.250e-017	2.099e-017	-16.648	-16.678	-0.030
ZnCl4-2		7.090e-021	5.367e-021	-20.149	-20.270	-0.121
Zn(OH)4-2		5.577e-021	4.221e-021	-20.254	-20.375	-0.121

------------------------------Saturation indices-------------------------------

Phase	SI	log IAP	log KT	
Anhydrite	-3.76	-8.10	-4.34	CaSO4
Aragonite	-1.50	-9.78	-8.28	CaCO3
Barite	-1.94	-12.08	-10.13	BaSO4
Calcite	-1.35	-9.78	-8.43	CaCO3
Chalcedony	2.57	-1.09	-3.67	SiO2
Chrysotile	-3.84	29.57	33.42	Mg3Si2O5(OH)4
CO2(g)	-1.75	-3.10	-1.35	CO2
Dolomite	-2.26	-19.13	-16.86	CaMg(CO3)2
Fluorite	-3.79	-14.51	-10.72	CaF2

Gypsum	-3.51	-8.10	-4.58	CaSO4:2H2O
H2(g)	-22.00	-25.11	-3.11	H2
H2O(g)	-1.76	-0.00	1.76	H2O
Halite	-7.67	-6.11	1.56	NaCl
Hausmannite	-19.65	43.81	63.46	Mn3O4
Manganite	-7.07	18.27	25.34	MnOOH
O2(g)	-42.42	-45.33	-2.92	O2
Pyrochroite	-7.93	7.27	15.20	Mn(OH)2
Pyrolusite	-13.68	29.27	42.95	MnO2
Quartz	3.03	-1.09	-4.12	SiO2
Rhodochrosite	-1.57	-12.66	-11.10	MnCO3
Sepiolite	1.87	17.89	16.02	Mg2Si3O7.5OH:3H2O
Sepiolite(d)	-0.77	17.89	18.66	Mg2Si3O7.5OH:3H2O
SiO2(a)	1.70	-1.09	-2.79	SiO2
Smithsonite	-3.21	-13.11	-9.89	ZnCO3
Talc	4.87	27.38	22.52	Mg3Si4O10(OH)2
Willemite	-3.57	12.56	16.14	Zn2SiO4
Witherite	-5.16	-13.76	-8.60	BaCO3
Zn(OH)2(e)	-4.67	6.83	11.50	Zn(OH)2

Initial solution 13.   4LAlejandra-1

----------------------------Solution composition----------------------------

Elements	Molality	Moles
Alkalinity	2.265e-003	2.265e-003
Ba	2.194e-009	2.194e-009
Ca	2.381e-004	2.381e-004
Cl	1.254e-003	1.254e-003
Cu	2.687e-008	2.687e-008
F	6.610e-006	6.610e-006
K	2.569e-005	2.569e-005
Mg	5.330e-004	5.330e-004
Mn	6.583e-008	6.583e-008
Na	1.315e-003	1.315e-003
S(6)	7.111e-005	7.111e-005
Si	7.189e-002	7.189e-002
Zn	9.528e-008	9.528e-008

----------------------------Description of solution----------------------------

pH	=	7.000
pe	=	4.000
Activity of water	=	0.999
Ionic strength	=	4.066e-003
Mass of water (kg)	=	1.000e+000
Total carbon (mol/kg)	=	2.711e-003
Total CO2 (mol/kg)	=	2.711e-003
Temperature (deg C)	=	15.500
Electrical balance (eq)	=	-7.839e-004
Percent error, 100*(Cat-|An|)/(Cat+|An|)	=	-12.07
Iterations	=	9
Total H	=	1.113021e+002
Total O	=	5.580168e+001

----------------------------Distribution of species----------------------------

Species		Molality	Activity	Log Molality	Log Activity	Log Gamma
H+		1.064e-007	1.000e-007	-6.973	-7.000	-0.027
OH-		5.028e-008	4.694e-008	-7.299	-7.328	-0.030
H2O		5.551e+001	9.987e-001	1.744	-0.001	0.000
Ba	2.194e-009					
Ba+2		2.128e-009	1.630e-009	-8.672	-8.788	-0.116
BaSO4		4.093e-011	4.097e-011	-10.388	-10.388	0.000

	BaHCO3+	2.514e-011	2.349e-011	-10.600	-10.629	-0.029
	BaCO3	5.323e-013	5.328e-013	-12.274	-12.273	0.000
	BaOH+	5.901e-016	5.514e-016	-15.229	-15.259	-0.029
C(4)	2.711e-003					
	HCO3-	2.167e-003	2.028e-003	-2.664	-2.693	-0.029
	CO2	5.272e-004	5.277e-004	-3.278	-3.278	0.000
	MgHCO3+	9.816e-006	9.172e-006	-5.008	-5.038	-0.029
	CaHCO3+	4.120e-006	3.856e-006	-5.385	-5.414	-0.029
	NaHCO3	1.399e-006	1.400e-006	-5.854	-5.854	0.000
	CO3-2	9.981e-007	7.660e-007	-6.001	-6.116	-0.115
	MgCO3	2.520e-007	2.523e-007	-6.599	-6.598	0.000
	CaCO3	1.960e-007	1.962e-007	-6.708	-6.707	0.000
	ZnHCO3+	1.483e-008	1.386e-008	-7.829	-7.858	-0.029
	NaCO3-	1.143e-008	1.068e-008	-7.942	-7.972	-0.029
	ZnCO3	8.290e-009	8.298e-009	-8.081	-8.081	0.000
	MnHCO3+	8.130e-009	7.597e-009	-8.090	-8.119	-0.029
	MnCO3	2.555e-009	2.557e-009	-8.593	-8.592	0.000
	Zn(CO3)2-2	1.782e-010	1.359e-010	-9.749	-9.867	-0.118
	BaHCO3+	2.514e-011	2.349e-011	-10.600	-10.629	-0.029
	BaCO3	5.323e-013	5.328e-013	-12.274	-12.273	0.000
Ca	2.381e-004					
	Ca+2	2.322e-004	1.781e-004	-3.634	-3.749	-0.115
	CaHCO3+	4.120e-006	3.856e-006	-5.385	-5.414	-0.029
	CaSO4	1.625e-006	1.626e-006	-5.789	-5.789	0.000
	CaCO3	1.960e-007	1.962e-007	-6.708	-6.707	0.000
	CaF+	7.956e-009	7.434e-009	-8.099	-8.129	-0.029
	CaOH+	3.158e-010	2.951e-010	-9.501	-9.530	-0.029
	CaHSO4+	9.158e-013	8.557e-013	-12.038	-12.068	-0.029
Cl	1.254e-003					
	Cl-	1.254e-003	1.171e-003	-2.902	-2.931	-0.030
	MnCl+	2.145e-010	2.005e-010	-9.668	-9.698	-0.029
	ZnCl+	1.188e-010	1.110e-010	-9.925	-9.955	-0.029
	ZnCl2	1.307e-013	1.308e-013	-12.884	-12.883	0.000
	MnCl2	1.024e-013	1.025e-013	-12.990	-12.989	0.000
	ZnCl3-	1.734e-016	1.620e-016	-15.761	-15.790	-0.029
	MnCl3-	3.536e-017	3.304e-017	-16.451	-16.481	-0.029
	ZnCl4-2	1.154e-019	8.798e-020	-18.938	-19.056	-0.118
Cu(1)	3.889e-010					
	Cu+	3.889e-010	3.626e-010	-9.410	-9.441	-0.030
Cu(2)	2.649e-008					
	Cu(OH)2	1.576e-008	1.578e-008	-7.802	-7.802	0.000
	Cu+2	9.839e-009	7.573e-009	-8.007	-8.121	-0.114
	CuOH+	8.094e-010	7.562e-010	-9.092	-9.121	-0.030
	CuSO4	7.240e-011	7.247e-011	-10.140	-10.140	0.000
	Cu(OH)3-	1.016e-014	9.495e-015	-13.993	-14.022	-0.029
	Cu(OH)4-2	2.482e-020	1.892e-020	-19.605	-19.723	-0.118
F	6.610e-006					
	F-	6.454e-006	6.026e-006	-5.190	-5.220	-0.030
	MgF+	1.424e-007	1.331e-007	-6.846	-6.876	-0.029
	CaF+	7.956e-009	7.434e-009	-8.099	-8.129	-0.029
	NaF	4.254e-009	4.258e-009	-8.371	-8.371	0.000
	HF	7.636e-010	7.643e-010	-9.117	-9.117	0.000
	MnF+	1.875e-012	1.752e-012	-11.727	-11.756	-0.029
	HF2-	1.737e-014	1.623e-014	-13.760	-13.790	-0.029
	SiF6-2	1.695e-030	1.292e-030	-29.771	-29.889	-0.118
H(0)	1.560e-025					
	H2	7.799e-026	7.806e-026	-25.108	-25.108	0.000
K	2.569e-005					
	K+	2.568e-005	2.398e-005	-4.590	-4.620	-0.030
	KSO4-	7.622e-009	7.123e-009	-8.118	-8.147	-0.029
	KOH	8.297e-013	8.305e-013	-12.081	-12.081	0.000
Mg	5.330e-004					
	Mg+2	5.192e-004	3.993e-004	-3.285	-3.399	-0.114
	MgHCO3+	9.816e-006	9.172e-006	-5.008	-5.038	-0.029
	MgSO4	3.644e-006	3.647e-006	-5.438	-5.438	0.000
	MgCO3	2.520e-007	2.523e-007	-6.599	-6.598	0.000
	MgF+	1.424e-007	1.331e-007	-6.846	-6.876	-0.029

```
   MgOH+                 6.388e-009  5.970e-009    -8.195    -8.224    -0.029
Mn(2)           6.583e-008
   Mn+2                  5.461e-008  4.203e-008    -7.263    -7.376    -0.114
   MnHCO3+               8.130e-009  7.597e-009    -8.090    -8.119    -0.029
   MnCO3                 2.555e-009  2.557e-009    -8.593    -8.592     0.000
   MnSO4                 3.106e-010  3.109e-010    -9.508    -9.507     0.000
   MnCl+                 2.145e-010  2.005e-010    -9.668    -9.698    -0.029
   MnOH+                 5.189e-012  4.848e-012   -11.285   -11.314    -0.029
   MnF+                  1.875e-012  1.752e-012   -11.727   -11.756    -0.029
   MnCl2                 1.024e-013  1.025e-013   -12.990   -12.989     0.000
   MnCl3-                3.536e-017  3.304e-017   -16.451   -16.481    -0.029
Mn(3)           5.705e-030
   Mn+3                  5.705e-030  3.099e-030   -29.244   -29.509    -0.265
Na              1.315e-003
   Na+                   1.314e-003  1.228e-003    -2.882    -2.911    -0.029
   NaHCO3                1.399e-006  1.400e-006    -5.854    -5.854     0.000
   NaSO4-                3.105e-007  2.901e-007    -6.508    -6.537    -0.029
   NaCO3-                1.143e-008  1.068e-008    -7.942    -7.972    -0.029
   NaF                   4.254e-009  4.258e-009    -8.371    -8.371     0.000
   NaOH                  8.095e-011  8.103e-011   -10.092   -10.091     0.000
O(0)            0.000e+000
   O2                    0.000e+000  0.000e+000   -45.333   -45.333     0.000
S(6)            7.111e-005
   SO4-2                 6.552e-005  5.016e-005    -4.184    -4.300    -0.116
   MgSO4                 3.644e-006  3.647e-006    -5.438    -5.438     0.000
   CaSO4                 1.625e-006  1.626e-006    -5.789    -5.789     0.000
   NaSO4-                3.105e-007  2.901e-007    -6.508    -6.537    -0.029
   KSO4-                 7.622e-009  7.123e-009    -8.118    -8.147    -0.029
   ZnSO4                 5.914e-010  5.920e-010    -9.228    -9.228     0.000
   HSO4-                 4.278e-010  3.997e-010    -9.369    -9.398    -0.029
   MnSO4                 3.106e-010  3.109e-010    -9.508    -9.507     0.000
   CuSO4                 7.240e-011  7.247e-011   -10.140   -10.140     0.000
   BaSO4                 4.093e-011  4.097e-011   -10.388   -10.388     0.000
   CaHSO4+               9.158e-013  8.557e-013   -12.038   -12.068    -0.029
   Zn(SO4)2-2            3.414e-013  2.603e-013   -12.467   -12.585    -0.118
Si              7.189e-002
   H4SiO4                7.181e-002  7.188e-002    -1.144    -1.143     0.000
   H3SiO4-               7.978e-005  7.455e-005    -4.098    -4.128    -0.029
   H2SiO4-2              3.456e-011  2.635e-011   -10.461   -10.579    -0.118
   SiF6-2                1.695e-030  1.292e-030   -29.771   -29.889    -0.118
Zn              9.528e-008
   Zn+2                  7.089e-008  5.429e-008    -7.149    -7.265    -0.116
   ZnHCO3+               1.483e-008  1.386e-008    -7.829    -7.858    -0.029
   ZnCO3                 8.290e-009  8.298e-009    -8.081    -8.081     0.000
   ZnSO4                 5.914e-010  5.920e-010    -9.228    -9.228     0.000
   ZnOH+                 3.023e-010  2.824e-010    -9.520    -9.549    -0.029
   Zn(CO3)2-2            1.782e-010  1.359e-010    -9.749    -9.867    -0.118
   ZnCl+                 1.188e-010  1.110e-010    -9.925    -9.955    -0.029
   Zn(OH)2               6.811e-011  6.817e-011   -10.167   -10.166     0.000
   Zn(SO4)2-2            3.414e-013  2.603e-013   -12.467   -12.585    -0.118
   ZnCl2                 1.307e-013  1.308e-013   -12.884   -12.883     0.000
   Zn(OH)3-              2.304e-015  2.153e-015   -14.638   -14.667    -0.029
   ZnCl3-                1.734e-016  1.620e-016   -15.761   -15.790    -0.029
   ZnCl4-2               1.154e-019  8.798e-020   -18.938   -19.056    -0.118
   Zn(OH)4-2             4.470e-021  3.408e-021   -20.350   -20.468    -0.118

------------------------------Saturation indices-------------------------------

      Phase               SI  log IAP  log KT

      Anhydrite        -3.71   -8.05   -4.34  CaSO4
      Aragonite        -1.58   -9.87   -8.28  CaCO3
      Barite           -2.95  -13.09  -10.13  BaSO4
      Calcite          -1.43   -9.87   -8.43  CaCO3
      Chalcedony        2.52   -1.14   -3.67  SiO2
      Chrysotile       -3.90   29.52   33.42  Mg3Si2O5(OH)4
      CO2(g)           -1.93   -3.28   -1.35  CO2
```

Dolomite	-2.52	-19.38	-16.86	CaMg(CO3)2
Fluorite	-3.47	-14.19	-10.72	CaF2
Gypsum	-3.47	-8.05	-4.58	CaSO4:2H2O
H2(g)	-22.00	-25.11	-3.11	H2
H2O(g)	-1.76	-0.00	1.76	H2O
Halite	-7.40	-5.84	1.56	NaCl
Hausmannite	-21.59	41.87	63.46	Mn3O4
Manganite	-7.72	17.62	25.34	MnOOH
O2(g)	-42.42	-45.33	-2.92	O2
Pyrochroite	-8.58	6.62	15.20	Mn(OH)2
Pyrolusite	-14.33	28.62	42.95	MnO2
Quartz	2.98	-1.14	-4.12	SiO2
Rhodochrosite	-2.40	-13.49	-11.10	MnCO3
Sepiolite	1.75	17.77	16.02	Mg2Si3O7.5OH:3H2O
Sepiolite(d)	-0.89	17.77	18.66	Mg2Si3O7.5OH:3H2O
SiO2(a)	1.65	-1.14	-2.79	SiO2
Smithsonite	-3.49	-13.38	-9.89	ZnCO3
Talc	4.72	27.23	22.52	Mg3Si4O10(OH)2
Willemite	-3.81	12.33	16.14	Zn2SiO4
Witherite	-6.31	-14.90	-8.60	BaCO3
Zn(OH)2(e)	-4.77	6.73	11.50	Zn(OH)2

Initial solution 14.   4LClaudina-1

-----------------------------Solution composition------------------------------

Elements	Molality	Moles
Alkalinity	1.851e-003	1.851e-003
Ba	7.301e-010	7.301e-010
Ca	1.601e-004	1.601e-004
Cl	3.543e-004	3.543e-004
Cu	2.525e-008	2.525e-008
F	6.069e-006	6.069e-006
K	2.077e-005	2.077e-005
Mg	3.369e-004	3.369e-004
Mn	5.110e-008	5.110e-008
Na	1.029e-003	1.029e-003
S(6)	2.923e-005	2.923e-005
Si	4.172e-002	4.172e-002
Zn	3.374e-008	3.374e-008

----------------------------Description of solution----------------------------

pH	=	7.000
pe	=	4.000
Activity of water	=	0.999
Ionic strength	=	2.660e-003
Mass of water (kg)	=	1.000e+000
Total carbon (mol/kg)	=	2.233e-003
Total CO2 (mol/kg)	=	2.233e-003
Temperature (deg C)	=	16.800
Electrical balance (eq)	=	-2.253e-004
Percent error, 100*(Cat-|An|)/(Cat+|An|)	=	-5.25
Iterations	=	8
Total H	=	1.111811e+002
Total O	=	5.567948e+001

----------------------------Distribution of species----------------------------

Species	Molality	Activity	Log Molality	Log Activity	Log Gamma
H+	1.053e-007	1.000e-007	-6.977	-7.000	-0.023
OH-	5.536e-008	5.233e-008	-7.257	-7.281	-0.024
H2O	5.551e+001	9.992e-001	1.744	-0.000	0.000
Ba	7.301e-010				

Ba+2		7.159e-010	5.745e-010	-9.145	-9.241	-0.096
BaHCO3+		7.631e-012	7.216e-012	-11.117	-11.142	-0.024
BaSO4		6.347e-012	6.351e-012	-11.197	-11.197	0.000
BaCO3		1.665e-013	1.666e-013	-12.778	-12.778	0.000
BaOH+		2.057e-016	1.945e-016	-15.687	-15.711	-0.024
C(4)	2.233e-003					
HCO3-		1.792e-003	1.696e-003	-2.747	-2.770	-0.024
CO2		4.308e-004	4.311e-004	-3.366	-3.365	0.000
MgHCO3+		5.416e-006	5.122e-006	-5.266	-5.291	-0.024
CaHCO3+		2.483e-006	2.351e-006	-5.605	-5.629	-0.024
NaHCO3		9.274e-007	9.280e-007	-6.033	-6.032	0.000
CO3-2		8.239e-007	6.620e-007	-6.084	-6.179	-0.095
MgCO3		1.479e-007	1.480e-007	-6.830	-6.830	0.000
CaCO3		1.220e-007	1.221e-007	-6.914	-6.913	0.000
NaCO3-		8.288e-009	7.838e-009	-8.082	-8.106	-0.024
MnHCO3+		5.598e-009	5.294e-009	-8.252	-8.276	-0.024
ZnHCO3+		4.706e-009	4.450e-009	-8.327	-8.352	-0.024
ZnCO3		2.751e-009	2.753e-009	-8.561	-8.560	0.000
MnCO3		1.840e-009	1.841e-009	-8.735	-8.735	0.000
Zn(CO3)2-2		4.871e-011	3.896e-011	-10.312	-10.409	-0.097
BaHCO3+		7.631e-012	7.216e-012	-11.117	-11.142	-0.024
BaCO3		1.665e-013	1.666e-013	-12.778	-12.778	0.000
Ca	1.601e-004					
Ca+2		1.570e-004	1.261e-004	-3.804	-3.899	-0.095
CaHCO3+		2.483e-006	2.351e-006	-5.605	-5.629	-0.024
CaSO4		5.126e-007	5.129e-007	-6.290	-6.290	0.000
CaCO3		1.220e-007	1.221e-007	-6.914	-6.913	0.000
CaF+		5.383e-009	5.091e-009	-8.269	-8.293	-0.024
CaOH+		2.211e-010	2.091e-010	-9.655	-9.680	-0.024
CaHSO4+		2.892e-013	2.735e-013	-12.539	-12.563	-0.024
Cl	3.543e-004					
Cl-		3.543e-004	3.349e-004	-3.451	-3.475	-0.024
MnCl+		5.051e-011	4.777e-011	-10.297	-10.321	-0.024
ZnCl+		1.369e-011	1.295e-011	-10.863	-10.888	-0.024
MnCl2		6.978e-015	6.983e-015	-14.156	-14.156	0.000
ZnCl2		4.387e-015	4.390e-015	-14.358	-14.358	0.000
ZnCl3-		1.658e-018	1.568e-018	-17.780	-17.805	-0.024
MnCl3-		6.810e-019	6.440e-019	-18.167	-18.191	-0.024
ZnCl4-2		3.078e-022	2.462e-022	-21.512	-21.609	-0.097
Cu(1)	3.718e-010					
Cu+		3.718e-010	3.511e-010	-9.430	-9.455	-0.025
Cu(2)	2.487e-008					
Cu(OH)2		1.509e-008	1.510e-008	-7.821	-7.821	0.000
Cu+2		8.990e-009	7.238e-009	-8.046	-8.140	-0.094
CuOH+		7.648e-010	7.232e-010	-9.116	-9.141	-0.024
CuSO4		3.073e-011	3.075e-011	-10.512	-10.512	0.000
Cu(OH)3-		9.612e-015	9.090e-015	-14.017	-14.041	-0.024
Cu(OH)4-2		2.266e-020	1.812e-020	-19.645	-19.742	-0.097
F	6.069e-006					
F-		5.970e-006	5.643e-006	-5.224	-5.248	-0.024
MgF+		8.992e-008	8.503e-008	-7.046	-7.070	-0.024
CaF+		5.383e-009	5.091e-009	-8.269	-8.293	-0.024
NaF		3.157e-009	3.159e-009	-8.501	-8.500	0.000
HF		7.316e-010	7.320e-010	-9.136	-9.135	0.000
MnF+		1.445e-012	1.367e-012	-11.840	-11.864	-0.024
HF2-		1.559e-014	1.475e-014	-13.807	-13.831	-0.024
SiF6-2		5.554e-031	4.442e-031	-30.255	-30.352	-0.097
H(0)	1.539e-025					
H2		7.695e-026	7.700e-026	-25.114	-25.114	0.000
K	2.077e-005					
K+		2.077e-005	1.963e-005	-4.683	-4.707	-0.024
KSO4-		2.777e-009	2.626e-009	-8.556	-8.581	-0.024
KOH		6.797e-013	6.802e-013	-12.168	-12.167	0.000
Mg	3.369e-004					
Mg+2		3.302e-004	2.657e-004	-3.481	-3.576	-0.094
MgHCO3+		5.416e-006	5.122e-006	-5.266	-5.291	-0.024
MgSO4		1.105e-006	1.106e-006	-5.957	-5.956	0.000

```
   MgCO3              1.479e-007  1.480e-007    -6.830    -6.830     0.000
   MgF+               8.992e-008  8.503e-008    -7.046    -7.070    -0.024
   MgOH+              4.760e-009  4.502e-009    -8.322    -8.347    -0.024
Mn(2)            5.110e-008
   Mn+2               4.349e-008  3.501e-008    -7.362    -7.456    -0.094
   MnHCO3+            5.598e-009  5.294e-009    -8.252    -8.276    -0.024
   MnCO3              1.840e-009  1.841e-009    -8.735    -8.735     0.000
   MnSO4              1.169e-010  1.169e-010    -9.932    -9.932     0.000
   MnCl+              5.051e-011  4.777e-011   -10.297   -10.321    -0.024
   MnOH+              4.783e-012  4.523e-012   -11.320   -11.345    -0.024
   MnF+               1.445e-012  1.367e-012   -11.840   -11.864    -0.024
   MnCl2              6.978e-015  6.983e-015   -14.156   -14.156     0.000
   MnCl3-             6.810e-019  6.440e-019   -18.167   -18.191    -0.024
Mn(3)            5.220e-030
   Mn+3               5.220e-030  3.158e-030   -29.282   -29.501    -0.218
Na               1.029e-003
   Na+                1.028e-003  9.728e-004    -2.988    -3.012    -0.024
   NaHCO3             9.274e-007  9.280e-007    -6.033    -6.032     0.000
   NaSO4-             1.078e-007  1.019e-007    -6.967    -6.992    -0.024
   NaCO3-             8.288e-009  7.838e-009    -8.082    -8.106    -0.024
   NaF                3.157e-009  3.159e-009    -8.501    -8.500     0.000
   NaOH               6.418e-011  6.422e-011   -10.193   -10.192     0.000
O(0)             0.000e+000
   O2                 0.000e+000  0.000e+000   -44.875   -44.875     0.000
S(6)             2.923e-005
   SO4-2              2.750e-005  2.206e-005    -4.561    -4.656    -0.096
   MgSO4              1.105e-006  1.106e-006    -5.957    -5.956     0.000
   CaSO4              5.126e-007  5.129e-007    -6.290    -6.290     0.000
   NaSO4-             1.078e-007  1.019e-007    -6.967    -6.992    -0.024
   KSO4-              2.777e-009  2.626e-009    -8.556    -8.581    -0.024
   HSO4-              1.908e-010  1.804e-010    -9.719    -9.744    -0.024
   MnSO4              1.169e-010  1.169e-010    -9.932    -9.932     0.000
   ZnSO4              1.009e-010  1.010e-010    -9.996    -9.996     0.000
   CuSO4              3.073e-011  3.075e-011   -10.512   -10.512     0.000
   BaSO4              6.347e-012  6.351e-012   -11.197   -11.197     0.000
   CaHSO4+            2.892e-013  2.735e-013   -12.539   -12.563    -0.024
   Zn(SO4)2-2         2.415e-014  1.932e-014   -13.617   -13.714    -0.097
Si               4.172e-002
   H4SiO4             4.167e-002  4.170e-002    -1.380    -1.380     0.000
   H3SiO4-            4.811e-005  4.550e-005    -4.318    -4.342    -0.024
   H2SiO4-2           2.209e-011  1.767e-011   -10.656   -10.753    -0.097
   SiF6-2             5.554e-031  4.442e-031   -30.255   -30.352    -0.097
Zn               3.374e-008
   Zn+2               2.597e-008  2.084e-008    -7.586    -7.681    -0.096
   ZnHCO3+            4.706e-009  4.450e-009    -8.327    -8.352    -0.024
   ZnCO3              2.751e-009  2.753e-009    -8.561    -8.560     0.000
   ZnOH+              1.274e-010  1.204e-010    -9.895    -9.919    -0.024
   ZnSO4              1.009e-010  1.010e-010    -9.996    -9.996     0.000
   Zn(CO3)2-2         4.871e-011  3.896e-011   -10.312   -10.409    -0.097
   Zn(OH)2            2.618e-011  2.619e-011   -10.582   -10.582     0.000
   ZnCl+              1.369e-011  1.295e-011   -10.863   -10.888    -0.024
   Zn(SO4)2-2         2.415e-014  1.932e-014   -13.617   -13.714    -0.097
   ZnCl2              4.387e-015  4.390e-015   -14.358   -14.358     0.000
   Zn(OH)3-           8.752e-016  8.277e-016   -15.058   -15.082    -0.024
   ZnCl3-             1.658e-018  1.568e-018   -17.780   -17.805    -0.024
   Zn(OH)4-2          1.639e-021  1.311e-021   -20.785   -20.882    -0.097
   ZnCl4-2            3.078e-022  2.462e-022   -21.512   -21.609    -0.097

------------------------------Saturation indices-------------------------------

      Phase               SI log IAP  log KT

      Anhydrite        -4.22   -8.56   -4.34  CaSO4
      Aragonite        -1.79  -10.08   -8.29  CaCO3
      Barite           -3.79  -13.90  -10.11  BaSO4
      Calcite          -1.64  -10.08   -8.44  CaCO3
      Chalcedony        2.27   -1.38   -3.65  SiO2
```

Chrysotile	-4.73	28.51	33.25	Mg3Si2O5(OH)4
CO2(g)	-2.00	-3.37	-1.37	CO2
Dolomite	-2.94	-19.83	-16.89	CaMg(CO3)2
Fluorite	-3.69	-14.40	-10.70	CaF2
Gypsum	-3.97	-8.56	-4.58	CaSO4:2H2O
H2(g)	-22.00	-25.11	-3.11	H2
H2O(g)	-1.73	-0.00	1.73	H2O
Halite	-8.05	-6.49	1.56	NaCl
Hausmannite	-21.48	41.63	63.12	Mn3O4
Manganite	-7.80	17.54	25.34	MnOOH
O2(g)	-41.95	-44.87	-2.92	O2
Pyrochroite	-8.66	6.54	15.20	Mn(OH)2
Pyrolusite	-14.19	28.54	42.73	MnO2
Quartz	2.73	-1.38	-4.10	SiO2
Rhodochrosite	-2.53	-13.63	-11.10	MnCO3
Sepiolite	0.73	16.71	15.98	Mg2Si3O7.5OH:3H2O
Sepiolite(d)	-1.95	16.71	18.66	Mg2Si3O7.5OH:3H2O
SiO2(a)	1.40	-1.38	-2.78	SiO2
Smithsonite	-3.95	-13.86	-9.91	ZnCO3
Talc	3.40	25.76	22.36	Mg3Si4O10(OH)2
Willemite	-4.76	11.26	16.02	Zn2SiO4
Witherite	-6.83	-15.42	-8.59	BaCO3
Zn(OH)2(e)	-5.18	6.32	11.50	Zn(OH)2

Initial solution 15.   4LGraciela-1

-----------------------------Solution composition------------------------------

Elements	Molality	Moles
Alkalinity	1.625e-003	1.625e-003
Ba	1.900e-008	1.900e-008
Ca	3.756e-004	3.756e-004
Cl	3.640e-003	3.640e-003
Cu	5.370e-008	5.370e-008
F	6.603e-006	6.603e-006
K	4.312e-005	4.312e-005
Mg	9.949e-004	9.949e-004
Mn	2.137e-007	2.137e-007
Na	2.008e-003	2.008e-003
S(6)	1.838e-004	1.838e-004
Si	5.512e-002	5.512e-002
Zn	1.858e-007	1.858e-007

----------------------------Description of solution----------------------------

pH	=	7.000
pe	=	4.000
Activity of water	=	0.999
Ionic strength	=	6.646e-003
Mass of water (kg)	=	1.000e+000
Total carbon (mol/kg)	=	1.901e-003
Total CO2 (mol/kg)	=	1.901e-003
Temperature (deg C)	=	18.500
Electrical balance (eq)	=	-8.455e-004
Percent error, 100*(Cat-|An|)/(Cat+|An|)	=	-8.21
Iterations	=	9
Total H	=	1.112344e+002
Total O	=	5.573280e+001

----------------------------Distribution of species----------------------------

Species	Molality	Activity	Log Molality	Log Activity	Log Gamma
H+	1.079e-007	1.000e-007	-6.967	-7.000	-0.033
OH-	6.555e-008	6.011e-008	-7.183	-7.221	-0.038

H2O		5.551e+001	9.989e-001	1.744	-0.000	0.000
Ba	1.900e-008					
Ba+2		1.809e-008	1.296e-008	-7.742	-7.887	-0.145
BaSO4		7.478e-010	7.489e-010	-9.126	-9.126	0.001
BaHCO3+		1.556e-010	1.429e-010	-9.808	-9.845	-0.037
BaCO3		3.368e-012	3.373e-012	-11.473	-11.472	0.001
BaOH+		4.776e-015	4.386e-015	-14.321	-14.358	-0.037
C(4)	1.901e-003					
HCO3-		1.533e-003	1.412e-003	-2.814	-2.850	-0.036
CO2		3.482e-004	3.487e-004	-3.458	-3.458	0.001
MgHCO3+		1.223e-005	1.123e-005	-4.913	-4.950	-0.037
CaHCO3+		4.576e-006	4.213e-006	-5.339	-5.375	-0.036
NaHCO3		1.461e-006	1.463e-006	-5.835	-5.835	0.001
CO3-2		7.991e-007	5.741e-007	-6.097	-6.241	-0.144
MgCO3		3.449e-007	3.455e-007	-6.462	-6.462	0.001
CaCO3		2.252e-007	2.255e-007	-6.647	-6.647	0.001
ZnHCO3+		2.057e-008	1.889e-008	-7.687	-7.724	-0.037
MnHCO3+		1.829e-008	1.680e-008	-7.738	-7.775	-0.037
NaCO3-		1.535e-008	1.410e-008	-7.814	-7.851	-0.037
ZnCO3		1.216e-008	1.218e-008	-7.915	-7.914	0.001
MnCO3		6.078e-009	6.088e-009	-8.216	-8.216	0.001
Zn(CO3)2-2		2.101e-010	1.495e-010	-9.678	-9.825	-0.148
BaHCO3+		1.556e-010	1.429e-010	-9.808	-9.845	-0.037
BaCO3		3.368e-012	3.373e-012	-11.473	-11.472	0.001
Ca	3.756e-004					
Ca+2		3.651e-004	2.622e-004	-3.438	-3.581	-0.144
CaSO4		5.661e-006	5.669e-006	-5.247	-5.246	0.001
CaHCO3+		4.576e-006	4.213e-006	-5.339	-5.375	-0.036
CaCO3		2.252e-007	2.255e-007	-6.647	-6.647	0.001
CaF+		1.235e-008	1.135e-008	-7.908	-7.945	-0.037
CaOH+		4.732e-010	4.346e-010	-9.325	-9.362	-0.037
CaHSO4+		3.352e-012	3.078e-012	-11.475	-11.512	-0.037
Cl	3.640e-003					
Cl-		3.640e-003	3.339e-003	-2.439	-2.476	-0.037
MnCl+		1.977e-009	1.816e-009	-8.704	-8.741	-0.037
ZnCl+		7.761e-010	7.127e-010	-9.110	-9.147	-0.037
MnCl2		2.643e-012	2.647e-012	-11.578	-11.577	0.001
ZnCl2		2.423e-012	2.426e-012	-11.616	-11.615	0.001
ZnCl3-		9.512e-015	8.736e-015	-14.022	-14.059	-0.037
MnCl3-		2.651e-015	2.434e-015	-14.577	-14.614	-0.037
ZnCl4-2		1.950e-017	1.387e-017	-16.710	-16.858	-0.148
Cu(1)	7.924e-010					
Cu+		7.924e-010	7.252e-010	-9.101	-9.140	-0.039
Cu(2)	5.291e-008					
Cu(OH)2		3.060e-008	3.065e-008	-7.514	-7.514	0.001
Cu+2		2.037e-008	1.470e-008	-7.691	-7.833	-0.142
CuOH+		1.600e-009	1.469e-009	-8.796	-8.833	-0.037
CuSO4		3.301e-010	3.307e-010	-9.481	-9.481	0.001
Cu(OH)3-		2.009e-014	1.845e-014	-13.697	-13.734	-0.037
Cu(OH)4-2		5.169e-020	3.677e-020	-19.287	-19.434	-0.148
F	6.603e-006					
F-		6.326e-006	5.802e-006	-5.199	-5.236	-0.038
MgF+		2.579e-007	2.368e-007	-6.589	-6.626	-0.037
CaF+		1.235e-008	1.135e-008	-7.908	-7.945	-0.037
NaF		6.145e-009	6.155e-009	-8.211	-8.211	0.001
HF		7.741e-010	7.753e-010	-9.111	-9.111	0.001
MnF+		5.834e-012	5.358e-012	-11.234	-11.271	-0.037
HF2-		1.778e-014	1.632e-014	-13.750	-13.787	-0.037
SiF6-2		8.287e-031	5.895e-031	-30.082	-30.230	-0.148
H(0)	1.510e-025					
H2		7.552e-026	7.564e-026	-25.122	-25.121	0.001
K	4.312e-005					
K+		4.309e-005	3.953e-005	-4.366	-4.403	-0.037
KSO4-		3.106e-008	2.852e-008	-7.508	-7.545	-0.037
KOH		1.367e-012	1.369e-012	-11.864	-11.864	0.001
Mg	9.949e-004					
Mg+2		9.662e-004	6.968e-004	-3.015	-3.157	-0.142

	MgSO4	1.585e-005	1.587e-005	-4.800	-4.799	0.001
	MgHCO3+	1.223e-005	1.123e-005	-4.913	-4.950	-0.037
	MgCO3	3.449e-007	3.455e-007	-6.462	-6.462	0.001
	MgF+	2.579e-007	2.368e-007	-6.589	-6.626	-0.037
	MgOH+	1.510e-008	1.387e-008	-7.821	-7.858	-0.037
Mn(2)	2.137e-007					
	Mn+2	1.850e-007	1.335e-007	-6.733	-6.875	-0.142
	MnHCO3+	1.829e-008	1.680e-008	-7.738	-7.775	-0.037
	MnCO3	6.078e-009	6.088e-009	-8.216	-8.216	0.001
	MnSO4	2.408e-009	2.411e-009	-8.618	-8.618	0.001
	MnCl+	1.977e-009	1.816e-009	-8.704	-8.741	-0.037
	MnOH+	2.171e-011	1.994e-011	-10.663	-10.700	-0.037
	MnF+	5.834e-012	5.358e-012	-11.234	-11.271	-0.037
	MnCl2	2.643e-012	2.647e-012	-11.578	-11.577	0.001
	MnCl3-	2.651e-015	2.434e-015	-14.577	-14.614	-0.037
Mn(3)	3.363e-029					
	Mn+3	3.363e-029	1.563e-029	-28.473	-28.806	-0.333
Na	2.008e-003					
	Na+	2.006e-003	1.843e-003	-2.698	-2.734	-0.037
	NaHCO3	1.461e-006	1.463e-006	-5.835	-5.835	0.001
	NaSO4-	1.112e-006	1.021e-006	-5.954	-5.991	-0.037
	NaCO3-	1.535e-008	1.410e-008	-7.814	-7.851	-0.037
	NaF	6.145e-009	6.155e-009	-8.211	-8.211	0.001
	NaOH	1.215e-010	1.217e-010	-9.916	-9.915	0.001
O(0)	0.000e+000					
	O2	0.000e+000	0.000e+000	-44.284	-44.283	0.001
S(6)	1.838e-004					
	SO4-2	1.611e-004	1.153e-004	-3.793	-3.938	-0.145
	MgSO4	1.585e-005	1.587e-005	-4.800	-4.799	0.001
	CaSO4	5.661e-006	5.669e-006	-5.247	-5.246	0.001
	NaSO4-	1.112e-006	1.021e-006	-5.954	-5.991	-0.037
	KSO4-	3.106e-008	2.852e-008	-7.508	-7.545	-0.037
	ZnSO4	2.726e-009	2.730e-009	-8.564	-8.564	0.001
	MnSO4	2.408e-009	2.411e-009	-8.618	-8.618	0.001
	HSO4-	1.063e-009	9.767e-010	-8.973	-9.010	-0.037
	BaSO4	7.478e-010	7.489e-010	-9.126	-9.126	0.001
	CuSO4	3.301e-010	3.307e-010	-9.481	-9.481	0.001
	Zn(SO4)2-2	3.786e-012	2.694e-012	-11.422	-11.570	-0.148
	CaHSO4+	3.352e-012	3.078e-012	-11.475	-11.512	-0.037
Si	5.512e-002					
	H4SiO4	5.505e-002	5.514e-002	-1.259	-1.259	0.001
	H3SiO4-	6.992e-005	6.421e-005	-4.155	-4.192	-0.037
	H2SiO4-2	3.955e-011	2.813e-011	-10.403	-10.551	-0.148
	SiF6-2	8.287e-031	5.895e-031	-30.082	-30.230	-0.148
Zn	1.858e-007					
	Zn+2	1.484e-007	1.063e-007	-6.828	-6.973	-0.145
	ZnHCO3+	2.057e-008	1.889e-008	-7.687	-7.724	-0.037
	ZnCO3	1.216e-008	1.218e-008	-7.915	-7.914	0.001
	ZnSO4	2.726e-009	2.730e-009	-8.564	-8.564	0.001
	ZnCl+	7.761e-010	7.127e-010	-9.110	-9.147	-0.037
	ZnOH+	7.658e-010	7.033e-010	-9.116	-9.153	-0.037
	Zn(CO3)2-2	2.101e-010	1.495e-010	-9.678	-9.825	-0.148
	Zn(OH)2	1.333e-010	1.335e-010	-9.875	-9.874	0.001
	Zn(SO4)2-2	3.786e-012	2.694e-012	-11.422	-11.570	-0.148
	ZnCl2	2.423e-012	2.426e-012	-11.616	-11.615	0.001
	ZnCl3-	9.512e-015	8.736e-015	-14.022	-14.059	-0.037
	Zn(OH)3-	4.593e-015	4.218e-015	-14.338	-14.375	-0.037
	ZnCl4-2	1.950e-017	1.387e-017	-16.710	-16.858	-0.148
	Zn(OH)4-2	9.387e-021	6.678e-021	-20.027	-20.175	-0.148

------------------------------Saturation indices-------------------------------

Phase	SI	log IAP	log KT	
Anhydrite	-3.18	-7.52	-4.34	CaSO4
Aragonite	-1.52	-9.82	-8.30	CaCO3
Barite	-1.75	-11.83	-10.08	BaSO4

Calcite	-1.38	-9.82	-8.45	CaCO3
Chalcedony	2.37	-1.26	-3.63	SiO2
Chrysotile	-3.01	30.01	33.02	Mg3Si2O5(OH)4
CO2(g)	-2.07	-3.46	-1.39	CO2
Dolomite	-2.28	-19.22	-16.94	CaMg(CO3)2
Fluorite	-3.38	-14.05	-10.68	CaF2
Gypsum	-2.94	-7.52	-4.58	CaSO4:2H2O
H2(g)	-22.00	-25.12	-3.12	H2
H2O(g)	-1.68	-0.00	1.68	H2O
Halite	-6.78	-5.21	1.57	NaCl
Hausmannite	-19.30	43.37	62.67	Mn3O4
Manganite	-7.22	18.12	25.34	MnOOH
O2(g)	-41.35	-44.28	-2.93	O2
Pyrochroite	-8.08	7.12	15.20	Mn(OH)2
Pyrolusite	-13.32	29.12	42.44	MnO2
Quartz	2.82	-1.26	-4.08	SiO2
Rhodochrosite	-2.01	-13.12	-11.11	MnCO3
Sepiolite	1.98	17.91	15.93	Mg2Si3O7.5OH:3H2O
Sepiolite(d)	-0.75	17.91	18.66	Mg2Si3O7.5OH:3H2O
SiO2(a)	1.51	-1.26	-2.77	SiO2
Smithsonite	-3.29	-13.21	-9.93	ZnCO3
Talc	5.34	27.50	22.16	Mg3Si4O10(OH)2
Willemite	-3.08	12.79	15.88	Zn2SiO4
Witherite	-5.55	-14.13	-8.58	BaCO3
Zn(OH)2(e)	-4.47	7.03	11.50	Zn(OH)2

Initial solution 16.   4LIleana-1

-----------------------------Solution composition------------------------------

Elements	Molality	Moles
Alkalinity	5.684e-004	5.684e-004
Ba	1.095e-008	1.095e-008
Ca	1.326e-004	1.326e-004
Cl	1.019e-003	1.019e-003
Cu	3.788e-008	3.788e-008
F	2.798e-006	2.798e-006
K	2.488e-005	2.488e-005
Mg	3.049e-004	3.049e-004
Mn	1.406e-007	1.406e-007
Na	7.896e-004	7.896e-004
S(6)	3.832e-005	3.832e-005
Si	4.674e-002	4.674e-002
Zn	9.359e-008	9.359e-008

----------------------------Description of solution----------------------------

```
                                       pH  =   7.000
                                       pe  =   4.000
                        Activity of water  =   0.999
                           Ionic strength  =  2.143e-003
                       Mass of water (kg)  =  1.000e+000
                 Total carbon (mol/kg)  =  6.504e-004
                    Total CO2 (mol/kg)  =  6.504e-004
                   Temperature (deg C)  =  14.300
              Electrical balance (eq)  =  2.279e-005
 Percent error, 100*(Cat-|An|)/(Cat+|An|)  =   0.68
                               Iterations  =  10
                                  Total H  = 1.111999e+002
                                  Total O  = 5.569514e+001
```

----------------------------Distribution of species----------------------------

Species	Molality	Activity	Log Molality	Log Activity	Log Gamma

H+		1.048e-007	1.000e-007	-6.980	-7.000	-0.020
OH-		4.466e-008	4.245e-008	-7.350	-7.372	-0.022
H2O		5.551e+001	9.992e-001	1.744	-0.000	0.000
Ba	1.095e-008					
Ba+2		1.079e-008	8.848e-009	-7.967	-8.053	-0.086
BaSO4		1.320e-010	1.320e-010	-9.880	-9.879	0.000
BaHCO3+		3.134e-011	2.980e-011	-10.504	-10.526	-0.022
BaCO3		6.640e-013	6.643e-013	-12.178	-12.178	0.000
BaOH+		3.150e-015	2.995e-015	-14.502	-14.524	-0.022
C(4)	6.504e-004					
HCO3-		5.170e-004	4.921e-004	-3.287	-3.308	-0.021
CO2		1.308e-004	1.309e-004	-3.883	-3.883	0.000
MgHCO3+		1.451e-006	1.380e-006	-5.838	-5.860	-0.022
CaHCO3+		5.791e-007	5.512e-007	-6.237	-6.259	-0.021
CO3-2		2.195e-007	1.802e-007	-6.659	-6.744	-0.086
NaHCO3		2.077e-007	2.078e-007	-6.683	-6.682	0.000
MgCO3		3.620e-008	3.621e-008	-7.441	-7.441	0.000
CaCO3		2.756e-008	2.757e-008	-7.560	-7.560	0.000
MnHCO3+		5.044e-009	4.797e-009	-8.297	-8.319	-0.022
ZnHCO3+		4.567e-009	4.343e-009	-8.340	-8.362	-0.022
ZnCO3		2.519e-009	2.520e-009	-8.599	-8.599	0.000
MnCO3		1.565e-009	1.565e-009	-8.806	-8.805	0.000
NaCO3-		1.513e-009	1.439e-009	-8.820	-8.842	-0.022
BaHCO3+		3.134e-011	2.980e-011	-10.504	-10.526	-0.022
Zn(CO3)2-2		1.187e-011	9.708e-012	-10.926	-11.013	-0.087
BaCO3		6.640e-013	6.643e-013	-12.178	-12.178	0.000
Ca	1.326e-004					
Ca+2		1.314e-004	1.079e-004	-3.881	-3.967	-0.086
CaHCO3+		5.791e-007	5.512e-007	-6.237	-6.259	-0.021
CaSO4		5.775e-007	5.778e-007	-6.238	-6.238	0.000
CaCO3		2.756e-008	2.757e-008	-7.560	-7.560	0.000
CaF+		1.999e-009	1.901e-009	-8.699	-8.721	-0.022
CaOH+		1.881e-010	1.789e-010	-9.726	-9.747	-0.022
CaHSO4+		3.159e-013	3.005e-013	-12.500	-12.522	-0.022
Cl	1.019e-003					
Cl-		1.019e-003	9.691e-004	-2.992	-3.014	-0.022
MnCl+		4.541e-010	4.318e-010	-9.343	-9.365	-0.022
ZnCl+		1.179e-010	1.121e-010	-9.929	-9.950	-0.022
MnCl2		1.826e-013	1.827e-013	-12.739	-12.738	0.000
ZnCl2		1.087e-013	1.088e-013	-12.964	-12.963	0.000
ZnCl3-		1.164e-016	1.107e-016	-15.934	-15.956	-0.022
MnCl3-		5.127e-017	4.876e-017	-16.290	-16.312	-0.022
ZnCl4-2		6.018e-020	4.922e-020	-19.221	-19.308	-0.087
Cu(1)	5.451e-010					
Cu+		5.451e-010	5.177e-010	-9.264	-9.286	-0.022
Cu(2)	3.733e-008					
Cu(OH)2		2.281e-008	2.282e-008	-7.642	-7.642	0.000
Cu+2		1.331e-008	1.094e-008	-7.876	-7.961	-0.085
CuOH+		1.150e-009	1.093e-009	-8.939	-8.961	-0.022
CuSO4		6.159e-011	6.162e-011	-10.210	-10.210	0.000
Cu(OH)3-		1.445e-014	1.374e-014	-13.840	-13.862	-0.022
Cu(OH)4-2		3.349e-020	2.739e-020	-19.475	-19.562	-0.087
F	2.798e-006					
F-		2.757e-006	2.621e-006	-5.559	-5.582	-0.022
MgF+		3.697e-008	3.516e-008	-7.432	-7.454	-0.022
CaF+		1.999e-009	1.901e-009	-8.699	-8.721	-0.022
NaF		1.132e-009	1.132e-009	-8.946	-8.946	0.000
HF		3.255e-010	3.257e-010	-9.487	-9.487	0.000
MnF+		2.086e-012	1.983e-012	-11.681	-11.703	-0.022
HF2-		3.124e-015	2.971e-015	-14.505	-14.527	-0.022
SiF6-2		7.813e-033	6.390e-033	-32.107	-32.195	-0.087
H(0)	1.581e-025					
H2		7.903e-026	7.907e-026	-25.102	-25.102	0.000
K	2.488e-005					
K+		2.487e-005	2.365e-005	-4.604	-4.626	-0.022
KSO4-		4.287e-009	4.077e-009	-8.368	-8.390	-0.022
KOH		8.188e-013	8.193e-013	-12.087	-12.087	0.000

Mg	3.049e-004					
Mg+2		3.020e-004	2.482e-004	-3.520	-3.605	-0.085
MgHCO3+		1.451e-006	1.380e-006	-5.838	-5.860	-0.022
MgSO4		1.301e-006	1.302e-006	-5.886	-5.885	0.000
MgF+		3.697e-008	3.516e-008	-7.432	-7.454	-0.022
MgCO3		3.620e-008	3.621e-008	-7.441	-7.441	0.000
MgOH+		3.476e-009	3.306e-009	-8.459	-8.481	-0.022
Mn(2)	1.406e-007					
Mn+2		1.330e-007	1.094e-007	-6.876	-6.961	-0.085
MnHCO3+		5.044e-009	4.797e-009	-8.297	-8.319	-0.022
MnCO3		1.565e-009	1.565e-009	-8.806	-8.805	0.000
MnSO4		4.684e-010	4.687e-010	-9.329	-9.329	0.000
MnCl+		4.541e-010	4.318e-010	-9.343	-9.365	-0.022
MnOH+		1.195e-011	1.137e-011	-10.923	-10.944	-0.022
MnF+		2.086e-012	1.983e-012	-11.681	-11.703	-0.022
MnCl2		1.826e-013	1.827e-013	-12.739	-12.738	0.000
MnCl3-		5.127e-017	4.876e-017	-16.290	-16.312	-0.022
Mn(3)	1.051e-029					
Mn+3		1.051e-029	6.683e-030	-28.979	-29.175	-0.196
Na	7.896e-004					
Na+		7.893e-004	7.508e-004	-3.103	-3.124	-0.022
NaHCO3		2.077e-007	2.078e-007	-6.683	-6.682	0.000
NaSO4-		1.098e-007	1.044e-007	-6.959	-6.981	-0.022
NaCO3-		1.513e-009	1.439e-009	-8.820	-8.842	-0.022
NaF		1.132e-009	1.132e-009	-8.946	-8.946	0.000
NaOH		4.954e-011	4.956e-011	-10.305	-10.305	0.000
O(0)	0.000e+000					
O2		0.000e+000	0.000e+000	-45.759	-45.758	0.000
S(6)	3.832e-005					
SO4-2		3.632e-005	2.978e-005	-4.440	-4.526	-0.086
MgSO4		1.301e-006	1.302e-006	-5.886	-5.885	0.000
CaSO4		5.775e-007	5.778e-007	-6.238	-6.238	0.000
NaSO4-		1.098e-007	1.044e-007	-6.959	-6.981	-0.022
KSO4-		4.287e-009	4.077e-009	-8.368	-8.390	-0.022
MnSO4		4.684e-010	4.687e-010	-9.329	-9.329	0.000
ZnSO4		4.490e-010	4.493e-010	-9.348	-9.348	0.000
HSO4-		2.436e-010	2.317e-010	-9.613	-9.635	-0.022
BaSO4		1.320e-010	1.320e-010	-9.880	-9.879	0.000
CuSO4		6.159e-011	6.162e-011	-10.210	-10.210	0.000
CaHSO4+		3.159e-013	3.005e-013	-12.500	-12.522	-0.022
Zn(SO4)2-2		1.448e-013	1.184e-013	-12.839	-12.927	-0.087
Si	4.674e-002					
H4SiO4		4.669e-002	4.671e-002	-1.331	-1.331	0.000
H3SiO4-		4.857e-005	4.619e-005	-4.314	-4.335	-0.022
H2SiO4-2		1.828e-011	1.495e-011	-10.738	-10.825	-0.087
SiF6-2		7.813e-033	6.390e-033	-32.107	-32.195	-0.087
Zn	9.359e-008					
Zn+2		8.549e-008	7.010e-008	-7.068	-7.154	-0.086
ZnHCO3+		4.567e-009	4.343e-009	-8.340	-8.362	-0.022
ZnCO3		2.519e-009	2.520e-009	-8.599	-8.599	0.000
ZnSO4		4.490e-010	4.493e-010	-9.348	-9.348	0.000
ZnOH+		3.480e-010	3.309e-010	-9.458	-9.480	-0.022
ZnCl+		1.179e-010	1.121e-010	-9.929	-9.950	-0.022
Zn(OH)2		8.806e-011	8.810e-011	-10.055	-10.055	0.000
Zn(CO3)2-2		1.187e-011	9.708e-012	-10.926	-11.013	-0.087
Zn(SO4)2-2		1.448e-013	1.184e-013	-12.839	-12.927	-0.087
ZnCl2		1.087e-013	1.088e-013	-12.964	-12.963	0.000
Zn(OH)3-		2.927e-015	2.784e-015	-14.534	-14.555	-0.022
ZnCl3-		1.164e-016	1.107e-016	-15.934	-15.956	-0.022
ZnCl4-2		6.018e-020	4.922e-020	-19.221	-19.308	-0.087
Zn(OH)4-2		5.390e-021	4.408e-021	-20.268	-20.356	-0.087

------------------------------Saturation indices-------------------------------

Phase	SI	log IAP	log KT	
Anhydrite	-4.16	-8.49	-4.33	CaSO4

Aragonite	-2.44	-10.71	-8.28	CaCO3
Barite	-2.42	-12.58	-10.16	BaSO4
Calcite	-2.28	-10.71	-8.43	CaCO3
Chalcedony	2.35	-1.33	-3.68	SiO2
Chrysotile	-5.05	28.52	33.57	Mg3Si2O5(OH)4
CO2(g)	-2.55	-3.88	-1.33	CO2
Dolomite	-4.23	-21.06	-16.83	CaMg(CO3)2
Fluorite	-4.39	-15.13	-10.74	CaF2
Gypsum	-3.91	-8.49	-4.59	CaSO4:2H2O
H2(g)	-22.00	-25.10	-3.10	H2
H2O(g)	-1.80	-0.00	1.80	H2O
Halite	-7.70	-6.14	1.56	NaCl
Hausmannite	-20.66	43.12	63.78	Mn3O4
Manganite	-7.30	18.04	25.34	MnOOH
O2(g)	-42.85	-45.76	-2.91	O2
Pyrochroite	-8.16	7.04	15.20	Mn(OH)2
Pyrolusite	-14.12	29.04	43.16	MnO2
Quartz	2.81	-1.33	-4.14	SiO2
Rhodochrosite	-2.61	-13.71	-11.09	MnCO3
Sepiolite	0.75	16.80	16.05	Mg2Si3O7.5OH:3H2O
Sepiolite(d)	-1.86	16.80	18.66	Mg2Si3O7.5OH:3H2O
SiO2(a)	1.47	-1.33	-2.80	SiO2
Smithsonite	-4.02	-13.90	-9.88	ZnCO3
Talc	3.20	25.86	22.66	Mg3Si4O10(OH)2
Willemite	-3.88	12.36	16.24	Zn2SiO4
Witherite	-6.20	-14.80	-8.60	BaCO3
Zn(OH)2(e)	-4.66	6.84	11.50	Zn(OH)2

Initial solution 17.   4LKarina-1

-----------------------------Solution composition------------------------------

Elements	Molality	Moles
Alkalinity	2.342e-003	2.342e-003
Ba	3.155e-007	3.155e-007
Ca	1.827e-004	1.827e-004
Cl	3.346e-004	3.346e-004
Cu	4.577e-008	4.577e-008
F	5.807e-006	5.807e-006
K	2.463e-005	2.463e-005
Mg	6.518e-004	6.518e-004
Mn	1.442e-007	1.442e-007
Na	1.038e-003	1.038e-003
S(6)	1.451e-005	1.451e-005
Si	4.674e-002	4.674e-002
Zn	8.899e-008	8.899e-008

----------------------------Description of solution----------------------------

pH	=	7.000
pe	=	4.000
Activity of water	=	0.999
Ionic strength	=	3.534e-003
Mass of water (kg)	=	1.000e+000
Total carbon (mol/kg)	=	2.903e-003
Total CO2 (mol/kg)	=	2.903e-003
Temperature (deg C)	=	11.300
Electrical balance (eq)	=	2.139e-005
Percent error, 100*(Cat-|An|)/(Cat+|An|)	=	0.40
Iterations	=	9
Total H	=	1.112017e+002
Total O	=	5.570134e+001

----------------------------Distribution of species----------------------------

Log   Log   Log

Species		Molality	Activity	Molality	Activity	Gamma
H+		1.060e-007	1.000e-007	-6.975	-7.000	-0.025
OH-		3.494e-008	3.278e-008	-7.457	-7.484	-0.028
H2O		5.551e+001	9.991e-001	1.744	-0.000	0.000
Ba	3.155e-007					
Ba+2		3.107e-007	2.423e-007	-6.508	-6.616	-0.108
BaHCO3+		3.444e-009	3.233e-009	-8.463	-8.490	-0.027
BaSO4		1.264e-009	1.265e-009	-8.898	-8.898	0.000
BaCO3		6.868e-011	6.873e-011	-10.163	-10.163	0.000
BaOH+		8.737e-014	8.202e-014	-13.059	-13.086	-0.027
C(4)	2.903e-003					
HCO3-		2.279e-003	2.143e-003	-2.642	-2.669	-0.027
CO2		6.053e-004	6.057e-004	-3.218	-3.218	0.000
MgHCO3+		1.280e-005	1.202e-005	-4.893	-4.920	-0.027
CaHCO3+		3.070e-006	2.887e-006	-5.513	-5.540	-0.027
NaHCO3		1.173e-006	1.174e-006	-5.931	-5.930	0.000
CO3-2		9.257e-007	7.230e-007	-6.034	-6.141	-0.107
MgCO3		2.788e-007	2.791e-007	-6.555	-6.554	0.000
CaCO3		1.394e-007	1.395e-007	-6.856	-6.855	0.000
MnHCO3+		1.904e-008	1.788e-008	-7.720	-7.748	-0.027
ZnHCO3+		1.483e-008	1.392e-008	-7.829	-7.856	-0.027
ZnCO3		7.438e-009	7.444e-009	-8.129	-8.128	0.000
NaCO3-		6.771e-009	6.356e-009	-8.169	-8.197	-0.027
MnCO3		5.372e-009	5.377e-009	-8.270	-8.269	0.000
BaHCO3+		3.444e-009	3.233e-009	-8.463	-8.490	-0.027
Zn(CO3)2-2		1.482e-010	1.151e-010	-9.829	-9.939	-0.110
BaCO3		6.868e-011	6.873e-011	-10.163	-10.163	0.000
Ca	1.827e-004					
Ca+2		1.792e-004	1.399e-004	-3.747	-3.854	-0.107
CaHCO3+		3.070e-006	2.887e-006	-5.513	-5.540	-0.027
CaSO4		2.541e-007	2.544e-007	-6.595	-6.595	0.000
CaCO3		1.394e-007	1.395e-007	-6.856	-6.855	0.000
CaF+		4.927e-009	4.625e-009	-8.307	-8.335	-0.027
CaOH+		2.472e-010	2.320e-010	-9.607	-9.634	-0.027
CaHSO4+		1.370e-013	1.286e-013	-12.863	-12.891	-0.027
Cl	3.346e-004					
Cl-		3.346e-004	3.140e-004	-3.475	-3.503	-0.028
MnCl+		1.275e-010	1.197e-010	-9.894	-9.922	-0.027
ZnCl+		2.466e-011	2.315e-011	-10.608	-10.635	-0.027
MnCl2		1.640e-014	1.641e-014	-13.785	-13.785	0.000
ZnCl2		7.177e-015	7.183e-015	-14.144	-14.144	0.000
ZnCl3-		2.473e-018	2.321e-018	-17.607	-17.634	-0.027
MnCl3-		1.512e-018	1.419e-018	-17.821	-17.848	-0.027
ZnCl4-2		4.197e-022	3.260e-022	-21.377	-21.487	-0.110
Cu(1)	6.372e-010					
Cu+		6.372e-010	5.971e-010	-9.196	-9.224	-0.028
Cu(2)	4.514e-008					
Cu(OH)2		2.711e-008	2.713e-008	-7.567	-7.566	0.000
Cu+2		1.661e-008	1.301e-008	-7.780	-7.886	-0.106
CuOH+		1.385e-009	1.300e-009	-8.859	-8.886	-0.027
CuSO4		2.504e-011	2.506e-011	-10.601	-10.601	0.000
Cu(OH)3-		1.740e-014	1.634e-014	-13.759	-13.787	-0.027
Cu(OH)4-2		4.193e-020	3.257e-020	-19.377	-19.487	-0.110
F	5.807e-006					
F-		5.655e-006	5.305e-006	-5.248	-5.275	-0.028
MgF+		1.437e-007	1.349e-007	-6.843	-6.870	-0.027
CaF+		4.927e-009	4.625e-009	-8.307	-8.335	-0.027
NaF		2.971e-009	2.974e-009	-8.527	-8.527	0.000
HF		6.258e-010	6.263e-010	-9.204	-9.203	0.000
MnF+		3.660e-012	3.436e-012	-11.437	-11.464	-0.027
HF2-		1.192e-014	1.119e-014	-13.924	-13.951	-0.027
SiF6-2		7.641e-031	5.935e-031	-30.117	-30.227	-0.110
H(0)	1.632e-025					
H2		8.161e-026	8.168e-026	-25.088	-25.088	0.000
K	2.463e-005					
K+		2.462e-005	2.310e-005	-4.609	-4.636	-0.028

KSO4-	1.402e-009	1.316e-009	-8.853	-8.881	-0.027
KOH	7.997e-013	8.003e-013	-12.097	-12.097	0.000
Mg	6.518e-004				
Mg+2	6.378e-004	4.992e-004	-3.195	-3.302	-0.106
MgHCO3+	1.280e-005	1.202e-005	-4.893	-4.920	-0.027
MgSO4	8.415e-007	8.422e-007	-6.075	-6.075	0.000
MgCO3	2.788e-007	2.791e-007	-6.555	-6.554	0.000
MgF+	1.437e-007	1.349e-007	-6.843	-6.870	-0.027
MgOH+	5.274e-009	4.951e-009	-8.278	-8.305	-0.027
Mn(2)	1.442e-007				
Mn+2	1.195e-007	9.362e-008	-6.922	-7.029	-0.106
MnHCO3+	1.904e-008	1.788e-008	-7.720	-7.748	-0.027
MnCO3	5.372e-009	5.377e-009	-8.270	-8.269	0.000
MnSO4	1.318e-010	1.319e-010	-9.880	-9.880	0.000
MnCl+	1.275e-010	1.197e-010	-9.894	-9.922	-0.027
MnOH+	7.944e-012	7.458e-012	-11.100	-11.127	-0.027
MnF+	3.660e-012	3.436e-012	-11.437	-11.464	-0.027
MnCl2	1.640e-014	1.641e-014	-13.785	-13.785	0.000
MnCl3-	1.512e-018	1.419e-018	-17.821	-17.848	-0.027
Mn(3)	6.274e-030				
Mn+3	6.274e-030	3.553e-030	-29.202	-29.449	-0.247
Na	1.038e-003				
Na+	1.037e-003	9.741e-004	-2.984	-3.011	-0.027
NaHCO3	1.173e-006	1.174e-006	-5.931	-5.930	0.000
NaSO4-	4.946e-008	4.643e-008	-7.306	-7.333	-0.027
NaCO3-	6.771e-009	6.356e-009	-8.169	-8.197	-0.027
NaF	2.971e-009	2.974e-009	-8.527	-8.527	0.000
NaOH	6.425e-011	6.430e-011	-10.192	-10.192	0.000
O(0)	0.000e+000				
O2	0.000e+000	0.000e+000	-46.840	-46.839	0.000
S(6)	1.451e-005				
SO4-2	1.336e-005	1.042e-005	-4.874	-4.982	-0.108
MgSO4	8.415e-007	8.422e-007	-6.075	-6.075	0.000
CaSO4	2.541e-007	2.544e-007	-6.595	-6.595	0.000
NaSO4-	4.946e-008	4.643e-008	-7.306	-7.333	-0.027
KSO4-	1.402e-009	1.316e-009	-8.853	-8.881	-0.027
BaSO4	1.264e-009	1.265e-009	-8.898	-8.898	0.000
MnSO4	1.318e-010	1.319e-010	-9.880	-9.880	0.000
ZnSO4	1.127e-010	1.128e-010	-9.948	-9.948	0.000
HSO4-	8.145e-011	7.646e-011	-10.089	-10.117	-0.027
CuSO4	2.504e-011	2.506e-011	-10.601	-10.601	0.000
CaHSO4+	1.370e-013	1.286e-013	-12.863	-12.891	-0.027
Zn(SO4)2-2	1.374e-014	1.067e-014	-13.862	-13.972	-0.110
Si	4.674e-002				
H4SiO4	4.670e-002	4.674e-002	-1.331	-1.330	0.000
H3SiO4-	4.355e-005	4.088e-005	-4.361	-4.388	-0.027
H2SiO4-2	1.360e-011	1.056e-011	-10.867	-10.976	-0.110
SiF6-2	7.641e-031	5.935e-031	-30.117	-30.227	-0.110
Zn	8.899e-008				
Zn+2	6.618e-008	5.160e-008	-7.179	-7.287	-0.108
ZnHCO3+	1.483e-008	1.392e-008	-7.829	-7.856	-0.027
ZnCO3	7.438e-009	7.444e-009	-8.129	-8.128	0.000
ZnOH+	2.026e-010	1.902e-010	-9.693	-9.721	-0.027
Zn(CO3)2-2	1.482e-010	1.151e-010	-9.829	-9.939	-0.110
ZnSO4	1.127e-010	1.128e-010	-9.948	-9.948	0.000
Zn(OH)2	6.479e-011	6.485e-011	-10.188	-10.188	0.000
ZnCl+	2.466e-011	2.315e-011	-10.608	-10.635	-0.027
Zn(SO4)2-2	1.374e-014	1.067e-014	-13.862	-13.972	-0.110
ZnCl2	7.177e-015	7.183e-015	-14.144	-14.144	0.000
Zn(OH)3-	2.182e-015	2.049e-015	-14.661	-14.688	-0.027
ZnCl3-	2.473e-018	2.321e-018	-17.607	-17.634	-0.027
Zn(OH)4-2	4.177e-021	3.244e-021	-20.379	-20.489	-0.110
ZnCl4-2	4.197e-022	3.260e-022	-21.377	-21.487	-0.110

------------------------------Saturation indices------------------------------

Phase	SI	log IAP	log KT

Anhydrite	-4.50	-8.84	-4.33	CaSO4
Aragonite	-1.73	-9.99	-8.26	CaCO3
Barite	-1.38	-11.60	-10.22	BaSO4
Calcite	-1.58	-9.99	-8.42	CaCO3
Chalcedony	2.39	-1.33	-3.72	SiO2
Chrysotile	-4.54	29.43	33.98	Mg3Si2O5(OH)4
CO2(g)	-1.93	-3.22	-1.29	CO2
Dolomite	-2.68	-19.44	-16.76	CaMg(CO3)2
Fluorite	-3.63	-14.40	-10.78	CaF2
Gypsum	-4.25	-8.84	-4.59	CaSO4:2H2O
H2(g)	-22.00	-25.09	-3.09	H2
H2O(g)	-1.88	-0.00	1.88	H2O
Halite	-8.06	-6.51	1.55	NaCl
Hausmannite	-21.67	42.91	64.58	Mn3O4
Manganite	-7.37	17.97	25.34	MnOOH
O2(g)	-43.94	-46.84	-2.89	O2
Pyrochroite	-8.23	6.97	15.20	Mn(OH)2
Pyrolusite	-14.71	28.97	43.68	MnO2
Quartz	2.86	-1.33	-4.19	SiO2
Rhodochrosite	-2.09	-13.17	-11.08	MnCO3
Sepiolite	1.27	17.41	16.14	Mg2Si3O7.5OH:3H2O
Sepiolite(d)	-1.25	17.41	18.66	Mg2Si3O7.5OH:3H2O
SiO2(a)	1.50	-1.33	-2.83	SiO2
Smithsonite	-3.58	-13.43	-9.85	ZnCO3
Talc	3.74	26.77	23.04	Mg3Si4O10(OH)2
Willemite	-4.41	12.09	16.51	Zn2SiO4
Witherite	-4.13	-12.76	-8.62	BaCO3
Zn(OH)2(e)	-4.79	6.71	11.50	Zn(OH)2

Initial solution 18.   4LKaterina-1

----------------------------Solution composition-----------------------------

Elements	Molality	Moles
Alkalinity	3.142e-003	3.142e-003
Ba	1.899e-008	1.899e-008
Ca	2.979e-004	2.979e-004
Cl	8.832e-004	8.832e-004
Cu	3.947e-008	3.947e-008
F	4.911e-006	4.911e-006
K	3.182e-005	3.182e-005
Mg	1.052e-003	1.052e-003
Mn	2.557e-007	2.557e-007
Na	1.431e-003	1.431e-003
S(6)	5.306e-005	5.306e-005
Si	5.010e-002	5.010e-002
Zn	1.059e-007	1.059e-007

----------------------------Description of solution----------------------------

pH	=	7.000
pe	=	4.000
Activity of water	=	0.999
Ionic strength	=	5.460e-003
Mass of water (kg)	=	1.000e+000
Total carbon (mol/kg)	=	3.841e-003
Total CO2 (mol/kg)	=	3.841e-003
Temperature (deg C)	=	14.100
Electrical balance (eq)	=	2.817e-005
Percent error, 100*(Cat-|An|)/(Cat+|An|)	=	0.34
Iterations	=	10
Total H	=	1.112158e+002
Total O	=	5.571758e+001

---------------------------Distribution of species---------------------------

Species		Molality	Activity	Log Molality	Log Activity	Log Gamma
H+		1.072e-007	1.000e-007	-6.970	-7.000	-0.030
OH-		4.514e-008	4.173e-008	-7.345	-7.380	-0.034
H2O		5.551e+001	9.990e-001	1.744	-0.000	0.000
Ba	1.899e-008					
Ba+2		1.847e-008	1.363e-008	-7.734	-7.866	-0.132
BaHCO3+		2.832e-010	2.621e-010	-9.548	-9.581	-0.034
BaSO4		2.360e-010	2.363e-010	-9.627	-9.627	0.001
BaCO3		5.819e-012	5.826e-012	-11.235	-11.235	0.001
BaOH+		4.985e-015	4.613e-015	-14.302	-14.336	-0.034
C(4)	3.841e-003					
HCO3-		3.049e-003	2.828e-003	-2.516	-2.549	-0.033
CO2		7.544e-004	7.553e-004	-3.122	-3.122	0.001
MgHCO3+		2.614e-005	2.419e-005	-4.583	-4.616	-0.034
CaHCO3+		6.746e-006	6.257e-006	-5.171	-5.204	-0.033
NaHCO3		2.102e-006	2.105e-006	-5.677	-5.677	0.001
CO3-2		1.392e-006	1.030e-006	-5.856	-5.987	-0.131
MgCO3		6.291e-007	6.298e-007	-6.201	-6.201	0.001
CaCO3		3.117e-007	3.121e-007	-6.506	-6.506	0.001
MnHCO3+		4.074e-008	3.770e-008	-7.390	-7.424	-0.034
ZnHCO3+		2.072e-008	1.917e-008	-7.684	-7.717	-0.034
NaCO3-		1.550e-008	1.435e-008	-7.810	-7.843	-0.034
MnCO3		1.222e-008	1.224e-008	-7.913	-7.912	0.001
ZnCO3		1.105e-008	1.107e-008	-7.956	-7.956	0.001
Zn(CO3)2-2		3.322e-010	2.437e-010	-9.479	-9.613	-0.134
BaHCO3+		2.832e-010	2.621e-010	-9.548	-9.581	-0.034
BaCO3		5.819e-012	5.826e-012	-11.235	-11.235	0.001
Ca	2.979e-004					
Ca+2		2.895e-004	2.141e-004	-3.538	-3.669	-0.131
CaHCO3+		6.746e-006	6.257e-006	-5.171	-5.204	-0.033
CaSO4		1.328e-006	1.329e-006	-5.877	-5.876	0.001
CaCO3		3.117e-007	3.121e-007	-6.506	-6.506	0.001
CaF+		6.736e-009	6.234e-009	-8.172	-8.205	-0.034
CaOH+		3.835e-010	3.549e-010	-9.416	-9.450	-0.034
CaHSO4+		7.455e-013	6.900e-013	-12.128	-12.161	-0.034
Cl	8.832e-004					
Cl-		8.832e-004	8.166e-004	-3.054	-3.088	-0.034
MnCl+		5.377e-010	4.976e-010	-9.269	-9.303	-0.034
ZnCl+		7.766e-011	7.188e-011	-10.110	-10.143	-0.034
MnCl2		1.772e-013	1.774e-013	-12.752	-12.751	0.001
ZnCl2		5.866e-014	5.873e-014	-13.232	-13.231	0.001
ZnCl3-		5.433e-017	5.028e-017	-16.265	-16.299	-0.034
MnCl3-		4.311e-017	3.990e-017	-16.365	-16.399	-0.034
ZnCl4-2		2.564e-020	1.881e-020	-19.591	-19.726	-0.134
Cu(1)	5.618e-010					
Cu+		5.618e-010	5.185e-010	-9.250	-9.285	-0.035
Cu(2)	3.891e-008					
Cu(OH)2		2.287e-008	2.290e-008	-7.641	-7.640	0.001
Cu+2		1.479e-008	1.098e-008	-7.830	-7.959	-0.129
CuOH+		1.186e-009	1.097e-009	-8.926	-8.960	-0.034
CuSO4		7.164e-011	7.173e-011	-10.145	-10.144	0.001
Cu(OH)3-		1.489e-014	1.378e-014	-13.827	-13.861	-0.034
Cu(OH)4-2		3.744e-020	2.747e-020	-19.427	-19.561	-0.134
F	4.911e-006					
F-		4.709e-006	4.353e-006	-5.327	-5.361	-0.034
MgF+		1.918e-007	1.775e-007	-6.717	-6.751	-0.034
CaF+		6.736e-009	6.234e-009	-8.172	-8.205	-0.034
NaF		3.311e-009	3.316e-009	-8.480	-8.479	0.001
HF		5.383e-010	5.390e-010	-9.269	-9.268	0.001
MnF+		4.868e-012	4.505e-012	-11.313	-11.346	-0.034
HF2-		8.805e-015	8.149e-015	-14.055	-14.089	-0.034
SiF6-2		2.001e-031	1.468e-031	-30.699	-30.833	-0.134
H(0)	1.583e-025					
H2		7.914e-026	7.924e-026	-25.102	-25.101	0.001

K	3.182e-005				
K+	3.181e-005	2.941e-005	-4.497	-4.531	-0.034
KSO4-	6.341e-009	5.869e-009	-8.198	-8.231	-0.034
KOH	1.018e-012	1.019e-012	-11.992	-11.992	0.001
Mg	1.052e-003				
Mg+2	1.021e-003	7.576e-004	-2.991	-3.121	-0.130
MgHCO3+	2.614e-005	2.419e-005	-4.583	-4.616	-0.034
MgSO4	4.585e-006	4.591e-006	-5.339	-5.338	0.001
MgCO3	6.291e-007	6.298e-007	-6.201	-6.201	0.001
MgF+	1.918e-007	1.775e-007	-6.717	-6.751	-0.034
MgOH+	1.069e-008	9.893e-009	-7.971	-8.005	-0.034
Mn(2)	2.557e-007				
Mn+2	2.014e-007	1.496e-007	-6.696	-6.825	-0.129
MnHCO3+	4.074e-008	3.770e-008	-7.390	-7.424	-0.034
MnCO3	1.222e-008	1.224e-008	-7.913	-7.912	0.001
MnSO4	7.406e-010	7.416e-010	-9.130	-9.130	0.001
MnCl+	5.377e-010	4.976e-010	-9.269	-9.303	-0.034
MnOH+	1.650e-011	1.527e-011	-10.782	-10.816	-0.034
MnF+	4.868e-012	4.505e-012	-11.313	-11.346	-0.034
MnCl2	1.772e-013	1.774e-013	-12.752	-12.751	0.001
MnCl3-	4.311e-017	3.990e-017	-16.365	-16.399	-0.034
Mn(3)	1.778e-029				
Mn+3	1.778e-029	8.857e-030	-28.750	-29.053	-0.303
Na	1.431e-003				
Na+	1.429e-003	1.324e-003	-2.845	-2.878	-0.033
NaHCO3	2.102e-006	2.105e-006	-5.677	-5.677	0.001
NaSO4-	2.308e-007	2.136e-007	-6.637	-6.670	-0.034
NaCO3-	1.550e-008	1.435e-008	-7.810	-7.843	-0.034
NaF	3.311e-009	3.316e-009	-8.480	-8.479	0.001
NaOH	8.725e-011	8.736e-011	-10.059	-10.059	0.001
O(0)	0.000e+000				
O2	0.000e+000	0.000e+000	-45.830	-45.830	0.001
S(6)	5.306e-005				
SO4-2	4.691e-005	3.459e-005	-4.329	-4.461	-0.132
MgSO4	4.585e-006	4.591e-006	-5.339	-5.338	0.001
CaSO4	1.328e-006	1.329e-006	-5.877	-5.876	0.001
NaSO4-	2.308e-007	2.136e-007	-6.637	-6.670	-0.034
KSO4-	6.341e-009	5.869e-009	-8.198	-8.231	-0.034
MnSO4	7.406e-010	7.416e-010	-9.130	-9.130	0.001
ZnSO4	3.998e-010	4.003e-010	-9.398	-9.398	0.001
HSO4-	2.897e-010	2.681e-010	-9.538	-9.572	-0.034
BaSO4	2.360e-010	2.363e-010	-9.627	-9.627	0.001
CuSO4	7.164e-011	7.173e-011	-10.145	-10.144	0.001
CaHSO4+	7.455e-013	6.900e-013	-12.128	-12.161	-0.034
Zn(SO4)2-2	1.674e-013	1.228e-013	-12.776	-12.911	-0.134
Si	5.010e-002				
H4SiO4	5.004e-002	5.010e-002	-1.301	-1.300	0.001
H3SiO4-	5.311e-005	4.915e-005	-4.275	-4.308	-0.034
H2SiO4-2	2.136e-011	1.567e-011	-10.670	-10.805	-0.134
SiF6-2	2.001e-031	1.468e-031	-30.699	-30.833	-0.134
Zn	1.059e-007				
Zn+2	7.298e-008	5.385e-008	-7.137	-7.269	-0.132
ZnHCO3+	2.072e-008	1.917e-008	-7.684	-7.717	-0.034
ZnCO3	1.105e-008	1.107e-008	-7.956	-7.956	0.001
ZnSO4	3.998e-010	4.003e-010	-9.398	-9.398	0.001
Zn(CO3)2-2	3.322e-010	2.437e-010	-9.479	-9.613	-0.134
ZnOH+	2.702e-010	2.501e-010	-9.568	-9.602	-0.034
ZnCl+	7.766e-011	7.188e-011	-10.110	-10.143	-0.034
Zn(OH)2	6.758e-011	6.767e-011	-10.170	-10.170	0.001
Zn(SO4)2-2	1.674e-013	1.228e-013	-12.776	-12.911	-0.134
ZnCl2	5.866e-014	5.873e-014	-13.232	-13.231	0.001
Zn(OH)3-	2.310e-015	2.138e-015	-14.636	-14.670	-0.034
ZnCl3-	5.433e-017	5.028e-017	-16.265	-16.299	-0.034
ZnCl4-2	2.564e-020	1.881e-020	-19.591	-19.726	-0.134
Zn(OH)4-2	4.613e-021	3.385e-021	-20.336	-20.470	-0.134

------------------------------Saturation indices------------------------------

Phase	SI	log IAP	log KT	
Anhydrite	-3.80	-8.13	-4.33	CaSO4
Aragonite	-1.38	-9.66	-8.27	CaCO3
Barite	-2.16	-12.33	-10.16	BaSO4
Calcite	-1.23	-9.66	-8.43	CaCO3
Chalcedony	2.38	-1.30	-3.68	SiO2
Chrysotile	-3.56	30.04	33.60	Mg3Si2O5(OH)4
CO2(g)	-1.79	-3.12	-1.33	CO2
Dolomite	-1.94	-18.76	-16.83	CaMg(CO3)2
Fluorite	-3.65	-14.39	-10.74	CaF2
Gypsum	-3.55	-8.13	-4.59	CaSO4:2H2O
H2(g)	-22.00	-25.10	-3.10	H2
H2O(g)	-1.80	-0.00	1.80	H2O
Halite	-7.52	-5.97	1.56	NaCl
Hausmannite	-20.31	43.52	63.83	Mn3O4
Manganite	-7.17	18.17	25.34	MnOOH
O2(g)	-42.92	-45.83	-2.91	O2
Pyrochroite	-8.03	7.17	15.20	Mn(OH)2
Pyrolusite	-14.02	29.17	43.19	MnO2
Quartz	2.85	-1.30	-4.15	SiO2
Rhodochrosite	-1.72	-12.81	-11.09	MnCO3
Sepiolite	1.80	17.86	16.06	Mg2Si3O7.5OH:3H2O
Sepiolite(d)	-0.80	17.86	18.66	Mg2Si3O7.5OH:3H2O
SiO2(a)	1.51	-1.30	-2.80	SiO2
Smithsonite	-3.38	-13.26	-9.88	ZnCO3
Talc	4.75	27.44	22.69	Mg3Si4O10(OH)2
Willemite	-4.10	12.16	16.26	Zn2SiO4
Witherite	-5.25	-13.85	-8.60	BaCO3
Zn(OH)2(e)	-4.77	6.73	11.50	Zn(OH)2

Initial solution 19.   4LLinda-1

-----------------------------Solution composition------------------------------

Elements	Molality	Moles
Alkalinity	3.386e-003	3.386e-003
Ba	1.537e-008	1.537e-008
Ca	4.589e-004	4.589e-004
Cl	2.713e-003	2.713e-003
Cu	6.960e-008	6.960e-008
F	5.978e-006	5.978e-006
K	5.038e-005	5.038e-005
Mg	1.166e-003	1.166e-003
Mn	2.854e-007	2.854e-007
Na	2.142e-003	2.142e-003
S(6)	1.690e-004	1.690e-004
Si	7.863e-002	7.863e-002
Zn	1.707e-007	1.707e-007

----------------------------Description of solution----------------------------

```
                                       pH  =   7.000
                                       pe  =   4.000
                        Activity of water  =   0.998
                           Ionic strength  =  7.559e-003
                       Mass of water (kg)  =  1.000e+000
                 Total carbon (mol/kg)  =  4.013e-003
                    Total CO2 (mol/kg)  =  4.013e-003
                   Temperature (deg C)  =  18.500
               Electrical balance (eq)  = -9.994e-004
 Percent error, 100*(Cat-|An|)/(Cat+|An|)  =  -8.54
                               Iterations  =   9
                                  Total H  = 1.113301e+002
                                  Total O  = 5.583270e+001
```

----------------------------Distribution of species----------------------------

Species		Molality	Activity	Log Molality	Log Activity	Log Gamma
H+		1.084e-007	1.000e-007	-6.965	-7.000	-0.035
OH-		6.586e-008	6.009e-008	-7.181	-7.221	-0.040
H2O		5.551e+001	9.985e-001	1.744	-0.001	0.000
Ba	1.537e-008					
Ba+2		1.458e-008	1.024e-008	-7.836	-7.990	-0.153
BaSO4		5.248e-010	5.257e-010	-9.280	-9.279	0.001
BaHCO3+		2.595e-010	2.371e-010	-9.586	-9.625	-0.039
BaCO3		5.587e-012	5.596e-012	-11.253	-11.252	0.001
BaOH+		3.792e-015	3.465e-015	-14.421	-14.460	-0.039
C(4)	4.013e-003					
HCO3-		3.235e-003	2.964e-003	-2.490	-2.528	-0.038
CO2		7.311e-004	7.324e-004	-3.136	-3.135	0.001
MgHCO3+		2.935e-005	2.682e-005	-4.532	-4.572	-0.039
CaHCO3+		1.144e-005	1.048e-005	-4.942	-4.980	-0.038
NaHCO3		3.254e-006	3.259e-006	-5.488	-5.487	0.001
CO3-2		1.710e-006	1.205e-006	-5.767	-5.919	-0.152
MgCO3		8.235e-007	8.249e-007	-6.084	-6.084	0.001
CaCO3		5.600e-007	5.610e-007	-6.252	-6.251	0.001
MnHCO3+		4.525e-008	4.135e-008	-7.344	-7.384	-0.039
NaCO3-		3.435e-008	3.139e-008	-7.464	-7.503	-0.039
ZnHCO3+		3.291e-008	3.007e-008	-7.483	-7.522	-0.039
ZnCO3		1.935e-008	1.938e-008	-7.713	-7.713	0.001
MnCO3		1.496e-008	1.499e-008	-7.825	-7.824	0.001
Zn(CO3)2-2		7.164e-010	4.995e-010	-9.145	-9.301	-0.157
BaHCO3+		2.595e-010	2.371e-010	-9.586	-9.625	-0.039
BaCO3		5.587e-012	5.596e-012	-11.253	-11.252	0.001
Ca	4.589e-004					
Ca+2		4.410e-004	3.106e-004	-3.356	-3.508	-0.152
CaHCO3+		1.144e-005	1.048e-005	-4.942	-4.980	-0.038
CaSO4		5.956e-006	5.966e-006	-5.225	-5.224	0.001
CaCO3		5.600e-007	5.610e-007	-6.252	-6.251	0.001
CaF+		1.317e-008	1.204e-008	-7.880	-7.919	-0.039
CaOH+		5.633e-010	5.147e-010	-9.249	-9.288	-0.039
CaHSO4+		3.545e-012	3.240e-012	-11.450	-11.489	-0.039
Cl	2.713e-003					
Cl-		2.713e-003	2.476e-003	-2.567	-2.606	-0.040
MnCl+		1.728e-009	1.579e-009	-8.762	-8.802	-0.039
ZnCl+		4.385e-010	4.007e-010	-9.358	-9.397	-0.039
MnCl2		1.704e-012	1.707e-012	-11.769	-11.768	0.001
ZnCl2		1.010e-012	1.012e-012	-11.996	-11.995	0.001
ZnCl3-		2.955e-015	2.700e-015	-14.529	-14.569	-0.039
MnCl3-		1.274e-015	1.164e-015	-14.895	-14.934	-0.039
ZnCl4-2		4.560e-018	3.179e-018	-17.341	-17.498	-0.157
Cu(1)	1.027e-009					
Cu+		1.027e-009	9.342e-010	-8.989	-9.030	-0.041
Cu(2)	6.857e-008					
Cu(OH)2		3.939e-008	3.945e-008	-7.405	-7.404	0.001
Cu+2		2.674e-008	1.894e-008	-7.573	-7.723	-0.150
CuOH+		2.071e-009	1.891e-009	-8.684	-8.723	-0.039
CuSO4		3.777e-010	3.784e-010	-9.423	-9.422	0.001
Cu(OH)3-		2.598e-014	2.374e-014	-13.585	-13.625	-0.039
Cu(OH)4-2		6.783e-020	4.729e-020	-19.169	-19.325	-0.157
F	5.978e-006					
F-		5.695e-006	5.196e-006	-5.245	-5.284	-0.040
MgF+		2.639e-007	2.412e-007	-6.578	-6.618	-0.039
CaF+		1.317e-008	1.204e-008	-7.880	-7.919	-0.039
NaF		5.836e-009	5.846e-009	-8.234	-8.233	0.001
HF		6.931e-010	6.943e-010	-9.159	-9.158	0.001
MnF+		6.157e-012	5.626e-012	-11.211	-11.250	-0.039
HF2-		1.433e-014	1.309e-014	-13.844	-13.883	-0.039
SiF6-2		6.230e-031	4.344e-031	-30.206	-30.362	-0.157

H(0)	1.510e-025				
H2	7.551e-026	7.564e-026	-25.122	-25.121	0.001
K	5.038e-005				
K+	5.035e-005	4.595e-005	-4.298	-4.338	-0.040
KSO4-	3.223e-008	2.945e-008	-7.492	-7.531	-0.039
KOH	1.588e-012	1.591e-012	-11.799	-11.798	0.001
Mg	1.166e-003				
Mg+2	1.119e-003	7.925e-004	-2.951	-3.101	-0.150
MgHCO3+	2.935e-005	2.682e-005	-4.532	-4.572	-0.039
MgSO4	1.601e-005	1.603e-005	-4.796	-4.795	0.001
MgCO3	8.235e-007	8.249e-007	-6.084	-6.084	0.001
MgF+	2.639e-007	2.412e-007	-6.578	-6.618	-0.039
MgOH+	1.725e-008	1.577e-008	-7.763	-7.802	-0.039
Mn(2)	2.854e-007				
Mn+2	2.209e-007	1.565e-007	-6.656	-6.805	-0.150
MnHCO3+	4.525e-008	4.135e-008	-7.344	-7.384	-0.039
MnCO3	1.496e-008	1.499e-008	-7.825	-7.824	0.001
MnSO4	2.507e-009	2.512e-009	-8.601	-8.600	0.001
MnCl+	1.728e-009	1.579e-009	-8.762	-8.802	-0.039
MnOH+	2.558e-011	2.337e-011	-10.592	-10.631	-0.039
MnF+	6.157e-012	5.626e-012	-11.211	-11.250	-0.039
MnCl2	1.704e-012	1.707e-012	-11.769	-11.768	0.001
MnCl3-	1.274e-015	1.164e-015	-14.895	-14.934	-0.039
Mn(3)	4.127e-029				
Mn+3	4.127e-029	1.833e-029	-28.384	-28.737	-0.352
Na	2.142e-003				
Na+	2.138e-003	1.955e-003	-2.670	-2.709	-0.039
NaHCO3	3.254e-006	3.259e-006	-5.488	-5.487	0.001
NaSO4-	1.053e-006	9.624e-007	-5.977	-6.017	-0.039
NaCO3-	3.435e-008	3.139e-008	-7.464	-7.503	-0.039
NaF	5.836e-009	5.846e-009	-8.234	-8.233	0.001
NaOH	1.288e-010	1.290e-010	-9.890	-9.889	0.001
O(0)	0.000e+000				
O2	0.000e+000	0.000e+000	-44.284	-44.283	0.001
S(6)	1.690e-004				
SO4-2	1.459e-004	1.024e-004	-3.836	-3.990	-0.154
MgSO4	1.601e-005	1.603e-005	-4.796	-4.795	0.001
CaSO4	5.956e-006	5.966e-006	-5.225	-5.224	0.001
NaSO4-	1.053e-006	9.624e-007	-5.977	-6.017	-0.039
KSO4-	3.223e-008	2.945e-008	-7.492	-7.531	-0.039
MnSO4	2.507e-009	2.512e-009	-8.601	-8.600	0.001
ZnSO4	1.835e-009	1.839e-009	-8.736	-8.736	0.001
HSO4-	9.494e-010	8.675e-010	-9.023	-9.062	-0.039
BaSO4	5.248e-010	5.257e-010	-9.280	-9.279	0.001
CuSO4	3.777e-010	3.784e-010	-9.423	-9.422	0.001
CaHSO4+	3.545e-012	3.240e-012	-11.450	-11.489	-0.039
Zn(SO4)2-2	2.311e-012	1.611e-012	-11.636	-11.793	-0.157
Si	7.863e-002				
H4SiO4	7.853e-002	7.866e-002	-1.105	-1.104	0.001
H3SiO4-	1.003e-004	9.161e-005	-3.999	-4.038	-0.039
H2SiO4-2	5.757e-011	4.013e-011	-10.240	-10.396	-0.157
SiF6-2	6.230e-031	4.344e-031	-30.206	-30.362	-0.157
Zn	1.707e-007				
Zn+2	1.147e-007	8.059e-008	-6.940	-7.094	-0.153
ZnHCO3+	3.291e-008	3.007e-008	-7.483	-7.522	-0.039
ZnCO3	1.935e-008	1.938e-008	-7.713	-7.713	0.001
ZnSO4	1.835e-009	1.839e-009	-8.736	-8.736	0.001
Zn(CO3)2-2	7.164e-010	4.995e-010	-9.145	-9.301	-0.157
ZnOH+	5.833e-010	5.330e-010	-9.234	-9.273	-0.039
ZnCl+	4.385e-010	4.007e-010	-9.358	-9.397	-0.039
Zn(OH)2	1.010e-010	1.012e-010	-9.996	-9.995	0.001
Zn(SO4)2-2	2.311e-012	1.611e-012	-11.636	-11.793	-0.157
ZnCl2	1.010e-012	1.012e-012	-11.996	-11.995	0.001
Zn(OH)3-	3.495e-015	3.194e-015	-14.457	-14.496	-0.039
ZnCl3-	2.955e-015	2.700e-015	-14.529	-14.569	-0.039
ZnCl4-2	4.560e-018	3.179e-018	-17.341	-17.498	-0.157
Zn(OH)4-2	7.250e-021	5.054e-021	-20.140	-20.296	-0.157

----------------------------Saturation indices-----------------------------

Phase	SI	log IAP	log KT	
Anhydrite	-3.16	-7.50	-4.34	CaSO4
Aragonite	-1.13	-9.43	-8.30	CaCO3
Barite	-1.90	-11.98	-10.08	BaSO4
Calcite	-0.98	-9.43	-8.45	CaCO3
Chalcedony	2.53	-1.10	-3.63	SiO2
Chrysotile	-2.54	30.49	33.02	Mg3Si2O5(OH)4
CO2(g)	-1.75	-3.14	-1.39	CO2
Dolomite	-1.51	-18.45	-16.94	CaMg(CO3)2
Fluorite	-3.40	-14.08	-10.68	CaF2
Gypsum	-2.92	-7.50	-4.58	CaSO4:2H2O
H2(g)	-22.00	-25.12	-3.12	H2
H2O(g)	-1.68	-0.00	1.68	H2O
Halite	-6.88	-5.31	1.57	NaCl
Hausmannite	-19.09	43.58	62.67	Mn3O4
Manganite	-7.15	18.19	25.34	MnOOH
O2(g)	-41.35	-44.28	-2.93	O2
Pyrochroite	-8.01	7.19	15.20	Mn(OH)2
Pyrolusite	-13.25	29.19	42.44	MnO2
Quartz	2.98	-1.10	-4.08	SiO2
Rhodochrosite	-1.62	-12.72	-11.11	MnCO3
Sepiolite	2.55	18.49	15.93	Mg2Si3O7.5OH:3H2O
Sepiolite(d)	-0.17	18.49	18.66	Mg2Si3O7.5OH:3H2O
SiO2(a)	1.66	-1.10	-2.77	SiO2
Smithsonite	-3.08	-13.01	-9.93	ZnCO3
Talc	6.13	28.28	22.16	Mg3Si4O10(OH)2
Willemite	-3.17	12.71	15.88	Zn2SiO4
Witherite	-5.33	-13.91	-8.58	BaCO3
Zn(OH)2(e)	-4.60	6.90	11.50	Zn(OH)2

Initial solution 20.   4LLudmila-1

----------------------------Solution composition----------------------------

Elements	Molality	Moles
Alkalinity	1.075e-003	1.075e-003
Ba	8.039e-009	8.039e-009
Ca	1.227e-004	1.227e-004
Cl	6.239e-004	6.239e-004
Cu	4.897e-008	4.897e-008
F	2.166e-006	2.166e-006
K	1.746e-005	1.746e-005
Mg	2.374e-004	2.374e-004
Mn	1.407e-007	1.407e-007
Na	1.017e-003	1.017e-003
S(6)	1.923e-005	1.923e-005
Si	6.014e-002	6.014e-002
Zn	1.520e-007	1.520e-007

----------------------------Description of solution----------------------------

pH	=	7.000
pe	=	4.000
Activity of water	=	0.999
Ionic strength	=	2.117e-003
Mass of water (kg)	=	1.000e+000
Total carbon (mol/kg)	=	1.259e-003
Total CO2 (mol/kg)	=	1.259e-003
Temperature (deg C)	=	15.300
Electrical balance (eq)	=	1.595e-005
Percent error, 100*(Cat-|An|)/(Cat+|An|)	=	0.46
Iterations	=	9

Total H = 1.112539e+002
Total O = 5.575038e+001

----------------------------Distribution of species----------------------------

Species		Molality	Activity	Log Molality	Log Activity	Log Gamma
H+		1.048e-007	1.000e-007	-6.980	-7.000	-0.020
OH-		4.857e-008	4.618e-008	-7.314	-7.336	-0.022
H2O		5.551e+001	9.989e-001	1.744	-0.000	0.000
Ba	8.039e-009					
Ba+2		7.943e-009	6.518e-009	-8.100	-8.186	-0.086
BaSO4		4.917e-011	4.920e-011	-10.308	-10.308	0.000
BaHCO3+		4.632e-011	4.406e-011	-10.334	-10.356	-0.022
BaCO3		9.962e-013	9.967e-013	-12.002	-12.001	0.000
BaOH+		2.320e-015	2.206e-015	-14.635	-14.656	-0.022
C(4)	1.259e-003					
HCO3-		1.005e-003	9.570e-004	-2.998	-3.019	-0.021
CO2		2.498e-004	2.499e-004	-3.602	-3.602	0.000
MgHCO3+		2.198e-006	2.091e-006	-5.658	-5.680	-0.022
CaHCO3+		1.065e-006	1.014e-006	-5.973	-5.994	-0.021
NaHCO3		5.203e-007	5.206e-007	-6.284	-6.284	0.000
CO3-2		4.377e-007	3.596e-007	-6.359	-6.444	-0.085
MgCO3		5.703e-008	5.705e-008	-7.244	-7.244	0.000
CaCO3		5.140e-008	5.142e-008	-7.289	-7.289	0.000
ZnHCO3+		1.348e-008	1.282e-008	-7.870	-7.892	-0.022
MnHCO3+		9.426e-009	8.966e-009	-8.026	-8.047	-0.022
ZnCO3		7.631e-009	7.635e-009	-8.117	-8.117	0.000
NaCO3-		4.107e-009	3.907e-009	-8.386	-8.408	-0.022
MnCO3		3.001e-009	3.003e-009	-8.523	-8.522	0.000
Zn(CO3)2-2		7.171e-011	5.870e-011	-10.144	-10.231	-0.087
BaHCO3+		4.632e-011	4.406e-011	-10.334	-10.356	-0.022
BaCO3		9.962e-013	9.967e-013	-12.002	-12.001	0.000
Ca	1.227e-004					
Ca+2		1.213e-004	9.965e-005	-3.916	-4.002	-0.085
CaHCO3+		1.065e-006	1.014e-006	-5.973	-5.994	-0.021
CaSO4		2.725e-007	2.726e-007	-6.565	-6.564	0.000
CaCO3		5.140e-008	5.142e-008	-7.289	-7.289	0.000
CaF+		1.470e-009	1.398e-009	-8.833	-8.854	-0.022
CaOH+		1.737e-010	1.652e-010	-9.760	-9.782	-0.022
CaHSO4+		1.505e-013	1.432e-013	-12.822	-12.844	-0.022
Cl	6.239e-004					
Cl-		6.239e-004	5.933e-004	-3.205	-3.227	-0.022
MnCl+		2.671e-010	2.541e-010	-9.573	-9.595	-0.022
ZnCl+		1.148e-010	1.092e-010	-9.940	-9.962	-0.022
MnCl2		6.576e-014	6.580e-014	-13.182	-13.182	0.000
ZnCl2		6.513e-014	6.516e-014	-13.186	-13.186	0.000
ZnCl3-		4.294e-017	4.084e-017	-16.367	-16.389	-0.022
MnCl3-		1.130e-017	1.075e-017	-16.947	-16.969	-0.022
ZnCl4-2		1.370e-020	1.122e-020	-19.863	-19.950	-0.087
Cu(1)	7.124e-010					
Cu+		7.124e-010	6.769e-010	-9.147	-9.169	-0.022
Cu(2)	4.825e-008					
Cu(OH)2		2.951e-008	2.953e-008	-7.530	-7.530	0.000
Cu+2		1.721e-008	1.416e-008	-7.764	-7.849	-0.085
CuOH+		1.488e-009	1.415e-009	-8.828	-8.849	-0.022
CuSO4		4.061e-011	4.063e-011	-10.391	-10.391	0.000
Cu(OH)3-		1.869e-014	1.777e-014	-13.728	-13.750	-0.022
Cu(OH)4-2		4.328e-020	3.542e-020	-19.364	-19.451	-0.087
F	2.166e-006					
F-		2.141e-006	2.035e-006	-5.669	-5.691	-0.022
MgF+		2.275e-008	2.164e-008	-7.643	-7.665	-0.022
CaF+		1.470e-009	1.398e-009	-8.833	-8.854	-0.022
NaF		1.132e-009	1.133e-009	-8.946	-8.946	0.000
HF		2.571e-010	2.572e-010	-9.590	-9.590	0.000
MnF+		1.556e-012	1.480e-012	-11.808	-11.830	-0.022

HF2-		1.936e-015	1.841e-015	-14.713	-14.735	-0.022
SiF6-2		1.996e-033	1.634e-033	-32.700	-32.787	-0.087
H(0)	1.564e-025					
H2		7.819e-026	7.823e-026	-25.107	-25.107	0.000
K	1.746e-005					
K+		1.745e-005	1.660e-005	-4.758	-4.780	-0.022
KSO4-		1.550e-009	1.474e-009	-8.810	-8.831	-0.022
KOH		5.745e-013	5.748e-013	-12.241	-12.240	0.000
Mg	2.374e-004					
Mg+2		2.346e-004	1.930e-004	-3.630	-3.715	-0.085
MgHCO3+		2.198e-006	2.091e-006	-5.658	-5.680	-0.022
MgSO4		5.259e-007	5.262e-007	-6.279	-6.279	0.000
MgCO3		5.703e-008	5.705e-008	-7.244	-7.244	0.000
MgF+		2.275e-008	2.164e-008	-7.643	-7.665	-0.022
MgOH+		2.976e-009	2.830e-009	-8.526	-8.548	-0.022
Mn(2)	1.407e-007					
Mn+2		1.277e-007	1.051e-007	-6.894	-6.978	-0.085
MnHCO3+		9.426e-009	8.966e-009	-8.026	-8.047	-0.022
MnCO3		3.001e-009	3.003e-009	-8.523	-8.522	0.000
MnCl+		2.671e-010	2.541e-010	-9.573	-9.595	-0.022
MnSO4		2.324e-010	2.325e-010	-9.634	-9.634	0.000
MnOH+		1.253e-011	1.192e-011	-10.902	-10.924	-0.022
MnF+		1.556e-012	1.480e-012	-11.808	-11.830	-0.022
MnCl2		6.576e-014	6.580e-014	-13.182	-13.182	0.000
MnCl3-		1.130e-017	1.075e-017	-16.947	-16.969	-0.022
Mn(3)	1.179e-029					
Mn+3		1.179e-029	7.512e-030	-28.929	-29.124	-0.196
Na	1.017e-003					
Na+		1.017e-003	9.673e-004	-2.993	-3.014	-0.022
NaHCO3		5.203e-007	5.206e-007	-6.284	-6.284	0.000
NaSO4-		7.203e-008	6.851e-008	-7.142	-7.164	-0.022
NaCO3-		4.107e-009	3.907e-009	-8.386	-8.408	-0.022
NaF		1.132e-009	1.133e-009	-8.946	-8.946	0.000
NaOH		6.381e-011	6.384e-011	-10.195	-10.195	0.000
O(0)	0.000e+000					
O2		0.000e+000	0.000e+000	-45.404	-45.403	0.000
S(6)	1.923e-005					
SO4-2		1.835e-005	1.506e-005	-4.736	-4.822	-0.086
MgSO4		5.259e-007	5.262e-007	-6.279	-6.279	0.000
CaSO4		2.725e-007	2.726e-007	-6.565	-6.564	0.000
NaSO4-		7.203e-008	6.851e-008	-7.142	-7.164	-0.022
KSO4-		1.550e-009	1.474e-009	-8.810	-8.831	-0.022
ZnSO4		3.476e-010	3.477e-010	-9.459	-9.459	0.000
MnSO4		2.324e-010	2.325e-010	-9.634	-9.634	0.000
HSO4-		1.257e-010	1.195e-010	-9.901	-9.923	-0.022
BaSO4		4.917e-011	4.920e-011	-10.308	-10.308	0.000
CuSO4		4.061e-011	4.063e-011	-10.391	-10.391	0.000
CaHSO4+		1.505e-013	1.432e-013	-12.822	-12.844	-0.022
Zn(SO4)2-2		5.617e-014	4.598e-014	-13.250	-13.337	-0.087
Si	6.014e-002					
H4SiO4		6.007e-002	6.010e-002	-1.221	-1.221	0.000
H3SiO4-		6.502e-005	6.185e-005	-4.187	-4.209	-0.022
H2SiO4-2		2.632e-011	2.154e-011	-10.580	-10.667	-0.087
SiF6-2		1.996e-033	1.634e-033	-32.700	-32.787	-0.087
Zn	1.520e-007					
Zn+2		1.297e-007	1.064e-007	-6.887	-6.973	-0.086
ZnHCO3+		1.348e-008	1.282e-008	-7.870	-7.892	-0.022
ZnCO3		7.631e-009	7.635e-009	-8.117	-8.117	0.000
ZnOH+		5.727e-010	5.448e-010	-9.242	-9.264	-0.022
ZnSO4		3.476e-010	3.477e-010	-9.459	-9.459	0.000
Zn(OH)2		1.336e-010	1.337e-010	-9.874	-9.874	0.000
ZnCl+		1.148e-010	1.092e-010	-9.940	-9.962	-0.022
Zn(CO3)2-2		7.171e-011	5.870e-011	-10.144	-10.231	-0.087
ZnCl2		6.513e-014	6.516e-014	-13.186	-13.186	0.000
Zn(SO4)2-2		5.617e-014	4.598e-014	-13.250	-13.337	-0.087
Zn(OH)3-		4.439e-015	4.223e-015	-14.353	-14.374	-0.022
ZnCl3-		4.294e-017	4.084e-017	-16.367	-16.389	-0.022

ZnCl4-2	1.370e-020	1.122e-020	-19.863	-19.950	-0.087
Zn(OH)4-2	8.167e-021	6.685e-021	-20.088	-20.175	-0.087

------------------------------Saturation indices-------------------------------

Phase	SI	log IAP	log KT	
Anhydrite	-4.49	-8.82	-4.34	CaSO4
Aragonite	-2.17	-10.45	-8.28	CaCO3
Barite	-2.87	-13.01	-10.14	BaSO4
Calcite	-2.01	-10.45	-8.43	CaCO3
Chalcedony	2.45	-1.22	-3.67	SiO2
Chrysotile	-5.03	28.41	33.44	Mg3Si2O5(OH)4
CO2(g)	-2.26	-3.60	-1.34	CO2
Dolomite	-3.75	-20.60	-16.86	CaMg(CO3)2
Fluorite	-4.66	-15.38	-10.72	CaF2
Gypsum	-4.24	-8.82	-4.58	CaSO4:2H2O
H2(g)	-22.00	-25.11	-3.11	H2
H2O(g)	-1.77	-0.00	1.77	H2O
Halite	-7.80	-6.24	1.56	NaCl
Hausmannite	-20.45	43.06	63.51	Mn3O4
Manganite	-7.32	18.02	25.34	MnOOH
O2(g)	-42.49	-45.40	-2.91	O2
Pyrochroite	-8.18	7.02	15.20	Mn(OH)2
Pyrolusite	-13.96	29.02	42.98	MnO2
Quartz	2.91	-1.22	-4.13	SiO2
Rhodochrosite	-2.33	-13.42	-11.09	MnCO3
Sepiolite	0.88	16.91	16.02	Mg2Si3O7.5OH:3H2O
Sepiolite(d)	-1.75	16.91	18.66	Mg2Si3O7.5OH:3H2O
SiO2(a)	1.57	-1.22	-2.79	SiO2
Smithsonite	-3.52	-13.42	-9.89	ZnCO3
Talc	3.43	25.97	22.54	Mg3Si4O10(OH)2
Willemite	-3.32	12.83	16.15	Zn2SiO4
Witherite	-6.03	-14.63	-8.60	BaCO3
Zn(OH)2(e)	-4.47	7.03	11.50	Zn(OH)2

Initial solution 21.   4LMarta-1

-----------------------------Solution composition------------------------------

Elements	Molality	Moles
Alkalinity	2.134e-003	2.134e-003
Ba	9.510e-009	9.510e-009
Ca	2.331e-004	2.331e-004
Cl	1.529e-003	1.529e-003
Cu	3.004e-008	3.004e-008
F	4.707e-006	4.707e-006
K	3.623e-005	3.623e-005
Mg	6.075e-004	6.075e-004
Mn	1.061e-007	1.061e-007
Na	1.578e-003	1.578e-003
S(6)	7.938e-005	7.938e-005
Si	7.357e-002	7.357e-002
Zn	6.148e-008	6.148e-008

----------------------------Description of solution----------------------------

pH	=	7.000
pe	=	4.000
Activity of water	=	0.999
Ionic strength	=	4.422e-003
Mass of water (kg)	=	1.000e+000
Total carbon (mol/kg)	=	2.506e-003
Total CO2 (mol/kg)	=	2.506e-003
Temperature (deg C)	=	18.500
Electrical balance (eq)	=	-5.304e-004

Percent error, 100*(Cat-|An|)/(Cat+|An|) = -7.51
Iterations = 8
Total H = 1.113087e+002
Total O = 5.580788e+001

----------------------------Distribution of species----------------------------

Species		Molality	Activity	Log Molality	Log Activity	Log Gamma
H+		1.067e-007	1.000e-007	-6.972	-7.000	-0.028
OH-		6.457e-008	6.010e-008	-7.190	-7.221	-0.031
H2O		5.551e+001	9.986e-001	1.744	-0.001	0.000
Ba	9.510e-009					
Ba+2		9.206e-009	6.969e-009	-8.036	-8.157	-0.121
BaSO4		1.910e-010	1.912e-010	-9.719	-9.719	0.000
BaHCO3+		1.103e-010	1.028e-010	-9.957	-9.988	-0.031
BaCO3		2.423e-012	2.426e-012	-11.616	-11.615	0.000
BaOH+		2.531e-015	2.358e-015	-14.597	-14.627	-0.031
C(4)	2.506e-003					
HCO3-		2.023e-003	1.888e-003	-2.694	-2.724	-0.030
CO2		4.659e-004	4.664e-004	-3.332	-3.331	0.000
MgHCO3+		1.041e-005	9.702e-006	-4.982	-5.013	-0.031
CaHCO3+		3.966e-006	3.702e-006	-5.402	-5.432	-0.030
NaHCO3		1.558e-006	1.559e-006	-5.808	-5.807	0.000
CO3-2		1.012e-006	7.677e-007	-5.995	-6.115	-0.120
MgCO3		2.981e-007	2.984e-007	-6.526	-6.525	0.000
CaCO3		1.979e-007	1.981e-007	-6.703	-6.703	0.000
NaCO3-		1.612e-008	1.502e-008	-7.793	-7.823	-0.031
MnHCO3+		1.220e-008	1.136e-008	-7.914	-7.944	-0.031
ZnHCO3+		8.930e-009	8.320e-009	-8.049	-8.080	-0.031
ZnCO3		5.357e-009	5.362e-009	-8.271	-8.271	0.000
MnCO3		4.114e-009	4.119e-009	-8.386	-8.385	0.000
Zn(CO3)2-2		1.168e-010	8.801e-011	-9.932	-10.055	-0.123
BaHCO3+		1.103e-010	1.028e-010	-9.957	-9.988	-0.031
BaCO3		2.423e-012	2.426e-012	-11.616	-11.615	0.000
Ca	2.331e-004					
Ca+2		2.272e-004	1.723e-004	-3.644	-3.764	-0.120
CaHCO3+		3.966e-006	3.702e-006	-5.402	-5.432	-0.030
CaSO4		1.766e-006	1.768e-006	-5.753	-5.753	0.000
CaCO3		1.979e-007	1.981e-007	-6.703	-6.703	0.000
CaF+		5.875e-009	5.473e-009	-8.231	-8.262	-0.031
CaOH+		3.065e-010	2.855e-010	-9.514	-9.544	-0.031
CaHSO4+		1.030e-012	9.601e-013	-11.987	-12.018	-0.031
Cl	1.529e-003					
Cl-		1.529e-003	1.423e-003	-2.816	-2.847	-0.031
MnCl+		4.203e-010	3.916e-010	-9.376	-9.407	-0.031
ZnCl+		1.074e-010	1.000e-010	-9.969	-10.000	-0.031
MnCl2		2.430e-013	2.433e-013	-12.614	-12.614	0.000
ZnCl2		1.450e-013	1.452e-013	-12.839	-12.838	0.000
ZnCl3-		2.391e-016	2.228e-016	-15.621	-15.652	-0.031
MnCl3-		1.024e-016	9.537e-017	-15.990	-16.021	-0.031
ZnCl4-2		2.001e-019	1.507e-019	-18.699	-18.822	-0.123
Cu(1)	4.470e-010					
Cu+		4.470e-010	4.154e-010	-9.350	-9.381	-0.032
Cu(2)	2.959e-008					
Cu(OH)2		1.753e-008	1.755e-008	-7.756	-7.756	0.000
Cu+2		1.107e-008	8.423e-009	-7.956	-8.075	-0.119
CuOH+		9.030e-010	8.412e-010	-9.044	-9.075	-0.031
CuSO4		8.981e-011	8.990e-011	-10.047	-10.046	0.000
Cu(OH)3-		1.134e-014	1.056e-014	-13.946	-13.976	-0.031
Cu(OH)4-2		2.793e-020	2.104e-020	-19.554	-19.677	-0.123
F	4.707e-006					
F-		4.576e-006	4.259e-006	-5.340	-5.371	-0.031
MgF+		1.205e-007	1.123e-007	-6.919	-6.950	-0.031
CaF+		5.875e-009	5.473e-009	-8.231	-8.262	-0.031
NaF		3.596e-009	3.600e-009	-8.444	-8.444	0.000

	HF		5.686e-010	5.692e-010	-9.245	-9.245	0.000
	MnF+		2.136e-012	1.990e-012	-11.670	-11.701	-0.031
	HF2-		9.443e-015	8.797e-015	-14.025	-14.056	-0.031
	SiF6-2		1.636e-031	1.232e-031	-30.786	-30.909	-0.123
H(0)		1.511e-025					
	H2		7.556e-026	7.564e-026	-25.122	-25.121	0.000
K		3.623e-005					
	K+		3.622e-005	3.372e-005	-4.441	-4.472	-0.031
	KSO4-		1.239e-008	1.155e-008	-7.907	-7.938	-0.031
	KOH		1.166e-012	1.167e-012	-11.933	-11.933	0.000
Mg		6.075e-004					
	Mg+2		5.918e-004	4.501e-004	-3.228	-3.347	-0.119
	MgHCO3+		1.041e-005	9.702e-006	-4.982	-5.013	-0.031
	MgSO4		4.861e-006	4.866e-006	-5.313	-5.313	0.000
	MgCO3		2.981e-007	2.984e-007	-6.526	-6.525	0.000
	MgF+		1.205e-007	1.123e-007	-6.919	-6.950	-0.031
	MgOH+		9.614e-009	8.957e-009	-8.017	-8.048	-0.031
Mn(2)		1.061e-007					
	Mn+2		8.874e-008	6.754e-008	-7.052	-7.170	-0.119
	MnHCO3+		1.220e-008	1.136e-008	-7.914	-7.944	-0.031
	MnCO3		4.114e-009	4.119e-009	-8.386	-8.385	0.000
	MnSO4		5.785e-010	5.791e-010	-9.238	-9.237	0.000
	MnCl+		4.203e-010	3.916e-010	-9.376	-9.407	-0.031
	MnOH+		1.083e-011	1.009e-011	-10.965	-10.996	-0.031
	MnF+		2.136e-012	1.990e-012	-11.670	-11.701	-0.031
	MnCl2		2.430e-013	2.433e-013	-12.614	-12.614	0.000
	MnCl3-		1.024e-016	9.537e-017	-15.990	-16.021	-0.031
Mn(3)		1.496e-029					
	Mn+3		1.496e-029	7.909e-030	-28.825	-29.102	-0.277
Na		1.578e-003					
	Na+		1.576e-003	1.469e-003	-2.803	-2.833	-0.030
	NaHCO3		1.558e-006	1.559e-006	-5.808	-5.807	0.000
	NaSO4-		4.146e-007	3.863e-007	-6.382	-6.413	-0.031
	NaCO3-		1.612e-008	1.502e-008	-7.793	-7.823	-0.031
	NaF		3.596e-009	3.600e-009	-8.444	-8.444	0.000
	NaOH		9.681e-011	9.691e-011	-10.014	-10.014	0.000
O(0)		0.000e+000					
	O2		0.000e+000	0.000e+000	-44.284	-44.283	0.000
S(6)		7.938e-005					
	SO4-2		7.232e-005	5.473e-005	-4.141	-4.262	-0.121
	MgSO4		4.861e-006	4.866e-006	-5.313	-5.313	0.000
	CaSO4		1.766e-006	1.768e-006	-5.753	-5.753	0.000
	NaSO4-		4.146e-007	3.863e-007	-6.382	-6.413	-0.031
	KSO4-		1.239e-008	1.155e-008	-7.907	-7.938	-0.031
	MnSO4		5.785e-010	5.791e-010	-9.238	-9.237	0.000
	HSO4-		4.975e-010	4.635e-010	-9.303	-9.334	-0.031
	ZnSO4		4.263e-010	4.268e-010	-9.370	-9.370	0.000
	BaSO4		1.910e-010	1.912e-010	-9.719	-9.719	0.000
	CuSO4		8.981e-011	8.990e-011	-10.047	-10.046	0.000
	CaHSO4+		1.030e-012	9.601e-013	-11.987	-12.018	-0.031
	Zn(SO4)2-2		2.652e-013	1.998e-013	-12.576	-12.699	-0.123
Si		7.357e-002					
	H4SiO4		7.348e-002	7.356e-002	-1.134	-1.133	0.000
	H3SiO4-		9.195e-005	8.566e-005	-4.036	-4.067	-0.031
	H2SiO4-2		4.982e-011	3.753e-011	-10.303	-10.426	-0.123
	SiF6-2		1.636e-031	1.232e-031	-30.786	-30.909	-0.123
Zn		6.148e-008					
	Zn+2		4.625e-008	3.501e-008	-7.335	-7.456	-0.121
	ZnHCO3+		8.930e-009	8.320e-009	-8.049	-8.080	-0.031
	ZnCO3		5.357e-009	5.362e-009	-8.271	-8.271	0.000
	ZnSO4		4.263e-010	4.268e-010	-9.370	-9.370	0.000
	ZnOH+		2.486e-010	2.316e-010	-9.605	-9.635	-0.031
	Zn(CO3)2-2		1.168e-010	8.801e-011	-9.932	-10.055	-0.123
	ZnCl+		1.074e-010	1.000e-010	-9.969	-10.000	-0.031
	Zn(OH)2		4.391e-011	4.395e-011	-10.357	-10.357	0.000
	Zn(SO4)2-2		2.652e-013	1.998e-013	-12.576	-12.699	-0.123
	ZnCl2		1.450e-013	1.452e-013	-12.839	-12.838	0.000

Zn(OH)3-	1.490e-015	1.388e-015	-14.827	-14.858	-0.031
ZnCl3-	2.391e-016	2.228e-016	-15.621	-15.652	-0.031
ZnCl4-2	2.001e-019	1.507e-019	-18.699	-18.822	-0.123
Zn(OH)4-2	2.916e-021	2.197e-021	-20.535	-20.658	-0.123

------------------------------Saturation indices-------------------------------

Phase	SI	log IAP	log KT	
Anhydrite	-3.69	-8.03	-4.34	CaSO4
Aragonite	-1.58	-9.88	-8.30	CaCO3
Barite	-2.34	-12.42	-10.08	BaSO4
Calcite	-1.43	-9.88	-8.45	CaCO3
Chalcedony	2.50	-1.13	-3.63	SiO2
Chrysotile	-3.33	29.69	33.02	Mg3Si2O5(OH)4
CO2(g)	-1.94	-3.33	-1.39	CO2
Dolomite	-2.40	-19.34	-16.94	CaMg(CO3)2
Fluorite	-3.83	-14.51	-10.68	CaF2
Gypsum	-3.44	-8.03	-4.58	CaSO4:2H2O
H2(g)	-22.00	-25.12	-3.12	H2
H2O(g)	-1.68	-0.00	1.68	H2O
Halite	-7.25	-5.68	1.57	NaCl
Hausmannite	-20.19	42.49	62.67	Mn3O4
Manganite	-7.51	17.83	25.34	MnOOH
O2(g)	-41.35	-44.28	-2.93	O2
Pyrochroite	-8.37	6.83	15.20	Mn(OH)2
Pyrolusite	-13.62	28.83	42.44	MnO2
Quartz	2.95	-1.13	-4.08	SiO2
Rhodochrosite	-2.18	-13.29	-11.11	MnCO3
Sepiolite	1.97	17.91	15.93	Mg2Si3O7.5OH:3H2O
Sepiolite(d)	-0.75	17.91	18.66	Mg2Si3O7.5OH:3H2O
SiO2(a)	1.63	-1.13	-2.77	SiO2
Smithsonite	-3.64	-13.57	-9.93	ZnCO3
Talc	5.27	27.43	22.16	Mg3Si4O10(OH)2
Willemite	-3.92	11.95	15.88	Zn2SiO4
Witherite	-5.69	-14.27	-8.58	BaCO3
Zn(OH)2(e)	-4.96	6.54	11.50	Zn(OH)2

Initial solution 22.   4LNora-1

-----------------------------Solution composition------------------------------

Elements	Molality	Moles
Alkalinity	2.703e-003	2.703e-003
Ba	1.096e-008	1.096e-008
Ca	2.403e-004	2.403e-004
Cl	1.698e-003	1.698e-003
Cu	3.158e-008	3.158e-008
F	5.175e-006	5.175e-006
K	3.361e-005	3.361e-005
Mg	9.162e-004	9.162e-004
Mn	1.552e-007	1.552e-007
Na	1.527e-003	1.527e-003
S(6)	8.115e-005	8.115e-005
Si	5.010e-002	5.010e-002
Zn	4.451e-008	4.451e-008

----------------------------Description of solution----------------------------

pH	=	7.000
pe	=	4.000
Activity of water	=	0.999
Ionic strength	=	5.373e-003
Mass of water (kg)	=	1.000e+000
Total carbon (mol/kg)	=	3.269e-003
Total CO2 (mol/kg)	=	3.269e-003

```
                     Temperature (deg C)  =  15.900
                 Electrical balance (eq)  = -6.931e-004
 Percent error, 100*(Cat-|An|)/(Cat+|An|)  =  -8.30
                              Iterations  =   9
                                 Total H  = 1.112154e+002
                                 Total O  = 5.571610e+001
```

----------------------------Distribution of species----------------------------

Species		Molality	Activity	Log Molality	Log Activity	Log Gamma
H+		1.072e-007	1.000e-007	-6.970	-7.000	-0.030
OH-		5.250e-008	4.856e-008	-7.280	-7.314	-0.034
H2O		5.551e+001	9.990e-001	1.744	-0.000	0.000
Ba	1.096e-008					
Ba+2		1.060e-008	7.830e-009	-7.975	-8.106	-0.131
BaSO4		2.100e-010	2.103e-010	-9.678	-9.677	0.001
BaHCO3+		1.477e-010	1.367e-010	-9.831	-9.864	-0.033
BaCO3		3.116e-012	3.119e-012	-11.506	-11.506	0.001
BaOH+		2.863e-015	2.651e-015	-14.543	-14.577	-0.033
C(4)	3.269e-003					
HCO3-		2.615e-003	2.426e-003	-2.583	-2.615	-0.033
CO2		6.258e-004	6.265e-004	-3.204	-3.203	0.001
MgHCO3+		1.963e-005	1.818e-005	-4.707	-4.740	-0.033
CaHCO3+		4.872e-006	4.520e-006	-5.312	-5.345	-0.033
NaHCO3		1.925e-006	1.928e-006	-5.715	-5.715	0.001
CO3-2		1.250e-006	9.257e-007	-5.903	-6.034	-0.130
MgCO3		5.070e-007	5.076e-007	-6.295	-6.294	0.001
CaCO3		2.311e-007	2.314e-007	-6.636	-6.636	0.001
MnHCO3+		2.175e-008	2.014e-008	-7.663	-7.696	-0.033
NaCO3-		1.639e-008	1.517e-008	-7.786	-7.819	-0.033
ZnHCO3+		7.751e-009	7.176e-009	-8.111	-8.144	-0.033
MnCO3		6.840e-009	6.848e-009	-8.165	-8.164	0.001
ZnCO3		4.334e-009	4.340e-009	-8.363	-8.363	0.001
BaHCO3+		1.477e-010	1.367e-010	-9.831	-9.864	-0.033
Zn(CO3)2-2		1.169e-010	8.589e-011	-9.932	-10.066	-0.134
BaCO3		3.116e-012	3.119e-012	-11.506	-11.506	0.001
Ca	2.403e-004					
Ca+2		2.335e-004	1.729e-004	-3.632	-3.762	-0.131
CaHCO3+		4.872e-006	4.520e-006	-5.312	-5.345	-0.033
CaSO4		1.691e-006	1.693e-006	-5.772	-5.771	0.001
CaCO3		2.311e-007	2.314e-007	-6.636	-6.636	0.001
CaF+		6.020e-009	5.574e-009	-8.220	-8.254	-0.033
CaOH+		3.096e-010	2.867e-010	-9.509	-9.543	-0.033
CaHSO4+		9.664e-013	8.947e-013	-12.015	-12.048	-0.033
Cl	1.698e-003					
Cl-		1.698e-003	1.570e-003	-2.770	-2.804	-0.034
MnCl+		6.435e-010	5.957e-010	-9.191	-9.225	-0.033
ZnCl+		7.090e-011	6.564e-011	-10.149	-10.183	-0.033
MnCl2		4.078e-013	4.083e-013	-12.390	-12.389	0.001
ZnCl2		1.038e-013	1.039e-013	-12.984	-12.983	0.001
MnCl3-		1.907e-016	1.766e-016	-15.720	-15.753	-0.033
ZnCl3-		1.870e-016	1.731e-016	-15.728	-15.762	-0.033
ZnCl4-2		1.721e-019	1.265e-019	-18.764	-18.898	-0.134
Cu(1)	4.571e-010					
Cu+		4.571e-010	4.220e-010	-9.340	-9.375	-0.035
Cu(2)	3.112e-008					
Cu(OH)2		1.828e-008	1.830e-008	-7.738	-7.737	0.001
Cu+2		1.180e-008	8.777e-009	-7.928	-8.057	-0.129
CuOH+		9.473e-010	8.769e-010	-9.023	-9.057	-0.034
CuSO4		8.989e-011	9.000e-011	-10.046	-10.046	0.001
Cu(OH)3-		1.190e-014	1.102e-014	-13.924	-13.958	-0.033
Cu(OH)4-2		2.989e-020	2.196e-020	-19.524	-19.658	-0.134
F	5.175e-006					
F-		4.982e-006	4.607e-006	-5.303	-5.337	-0.034
MgF+		1.833e-007	1.697e-007	-6.737	-6.770	-0.033

CaF+		6.020e-009	5.574e-009	-8.220	-8.254	-0.033
NaF		3.741e-009	3.746e-009	-8.427	-8.426	0.001
HF		5.877e-010	5.884e-010	-9.231	-9.230	0.001
MnF+		3.206e-012	2.969e-012	-11.494	-11.527	-0.033
HF2-		1.036e-014	9.592e-015	-13.985	-14.018	-0.033
SiF6-2		2.351e-031	1.727e-031	-30.629	-30.763	-0.134
H(0)	1.553e-025					
H2		7.763e-026	7.773e-026	-25.110	-25.109	0.001
K	3.361e-005					
K+		3.360e-005	3.108e-005	-4.474	-4.507	-0.034
KSO4-		1.073e-008	9.934e-009	-7.969	-8.003	-0.033
KOH		1.075e-012	1.077e-012	-11.968	-11.968	0.001
Mg	9.162e-004					
Mg+2		8.893e-004	6.609e-004	-3.051	-3.180	-0.129
MgHCO3+		1.963e-005	1.818e-005	-4.707	-4.740	-0.033
MgSO4		6.510e-006	6.518e-006	-5.186	-5.186	0.001
MgCO3		5.070e-007	5.076e-007	-6.295	-6.294	0.001
MgF+		1.833e-007	1.697e-007	-6.737	-6.770	-0.033
MgOH+		1.109e-008	1.027e-008	-7.955	-7.988	-0.033
Mn(2)	1.552e-007					
Mn+2		1.252e-007	9.313e-008	-6.902	-7.031	-0.129
MnHCO3+		2.175e-008	2.014e-008	-7.663	-7.696	-0.033
MnCO3		6.840e-009	6.848e-009	-8.165	-8.164	0.001
MnSO4		7.410e-010	7.419e-010	-9.130	-9.130	0.001
MnCl+		6.435e-010	5.957e-010	-9.191	-9.225	-0.033
MnOH+		1.202e-011	1.113e-011	-10.920	-10.954	-0.033
MnF+		3.206e-012	2.969e-012	-11.494	-11.527	-0.033
MnCl2		4.078e-013	4.083e-013	-12.390	-12.389	0.001
MnCl3-		1.907e-016	1.766e-016	-15.720	-15.753	-0.033
Mn(3)	1.462e-029					
Mn+3		1.462e-029	7.307e-030	-28.835	-29.136	-0.301
Na	1.527e-003					
Na+		1.525e-003	1.413e-003	-2.817	-2.850	-0.033
NaHCO3		1.925e-006	1.928e-006	-5.715	-5.715	0.001
NaSO4-		3.862e-007	3.575e-007	-6.413	-6.447	-0.033
NaCO3-		1.639e-008	1.517e-008	-7.786	-7.819	-0.033
NaF		3.741e-009	3.746e-009	-8.427	-8.426	0.001
NaOH		9.315e-011	9.326e-011	-10.031	-10.030	0.001
O(0)	0.000e+000					
O2		0.000e+000	0.000e+000	-45.192	-45.191	0.001
S(6)	8.115e-005					
SO4-2		7.255e-005	5.358e-005	-4.139	-4.271	-0.132
MgSO4		6.510e-006	6.518e-006	-5.186	-5.186	0.001
CaSO4		1.691e-006	1.693e-006	-5.772	-5.771	0.001
NaSO4-		3.862e-007	3.575e-007	-6.413	-6.447	-0.033
KSO4-		1.073e-008	9.934e-009	-7.969	-8.003	-0.033
MnSO4		7.410e-010	7.419e-010	-9.130	-9.130	0.001
HSO4-		4.649e-010	4.304e-010	-9.333	-9.366	-0.033
ZnSO4		2.742e-010	2.745e-010	-9.562	-9.561	0.001
BaSO4		2.100e-010	2.103e-010	-9.678	-9.677	0.001
CuSO4		8.989e-011	9.000e-011	-10.046	-10.046	0.001
CaHSO4+		9.664e-013	8.947e-013	-12.015	-12.048	-0.033
Zn(SO4)2-2		1.749e-013	1.285e-013	-12.757	-12.891	-0.134
Si	5.010e-002					
H4SiO4		5.004e-002	5.010e-002	-1.301	-1.300	0.001
H3SiO4-		5.701e-005	5.278e-005	-4.244	-4.277	-0.033
H2SiO4-2		2.614e-011	1.921e-011	-10.583	-10.717	-0.134
SiF6-2		2.351e-031	1.727e-031	-30.629	-30.763	-0.134
Zn	4.451e-008					
Zn+2		3.180e-008	2.349e-008	-7.498	-7.629	-0.131
ZnHCO3+		7.751e-009	7.176e-009	-8.111	-8.144	-0.033
ZnCO3		4.334e-009	4.340e-009	-8.363	-8.363	0.001
ZnSO4		2.742e-010	2.745e-010	-9.562	-9.561	0.001
ZnOH+		1.364e-010	1.263e-010	-9.865	-9.899	-0.033
Zn(CO3)2-2		1.169e-010	8.589e-011	-9.932	-10.066	-0.134
ZnCl+		7.090e-011	6.564e-011	-10.149	-10.183	-0.033
Zn(OH)2		2.948e-011	2.952e-011	-10.530	-10.530	0.001

Zn(SO4)2-2	1.749e-013	1.285e-013	-12.757	-12.891	-0.134
ZnCl2	1.038e-013	1.039e-013	-12.984	-12.983	0.001
Zn(OH)3-	1.007e-015	9.326e-016	-14.997	-15.030	-0.033
ZnCl3-	1.870e-016	1.731e-016	-15.728	-15.762	-0.033
ZnCl4-2	1.721e-019	1.265e-019	-18.764	-18.898	-0.134
Zn(OH)4-2	2.010e-021	1.477e-021	-20.697	-20.831	-0.134

------------------------------Saturation indices-------------------------------

Phase	SI	log IAP	log KT	
Anhydrite	-3.70	-8.03	-4.34	CaSO4
Aragonite	-1.51	-9.80	-8.28	CaCO3
Barite	-2.25	-12.38	-10.13	BaSO4
Calcite	-1.36	-9.80	-8.43	CaCO3
Chalcedony	2.36	-1.30	-3.66	SiO2
Chrysotile	-3.50	29.86	33.36	Mg3Si2O5(OH)4
CO2(g)	-1.85	-3.20	-1.35	CO2
Dolomite	-2.14	-19.01	-16.87	CaMg(CO3)2
Fluorite	-3.72	-14.44	-10.71	CaF2
Gypsum	-3.45	-8.03	-4.58	CaSO4:2H2O
H2(g)	-22.00	-25.11	-3.11	H2
H2O(g)	-1.75	-0.00	1.75	H2O
Halite	-7.21	-5.65	1.56	NaCl
Hausmannite	-20.45	42.91	63.35	Mn3O4
Manganite	-7.37	17.97	25.34	MnOOH
O2(g)	-42.27	-45.19	-2.92	O2
Pyrochroite	-8.23	6.97	15.20	Mn(OH)2
Pyrolusite	-13.91	28.97	42.88	MnO2
Quartz	2.82	-1.30	-4.12	SiO2
Rhodochrosite	-1.97	-13.06	-11.10	MnCO3
Sepiolite	1.73	17.74	16.01	Mg2Si3O7.5OH:3H2O
Sepiolite(d)	-0.92	17.74	18.66	Mg2Si3O7.5OH:3H2O
SiO2(a)	1.49	-1.30	-2.79	SiO2
Smithsonite	-3.76	-13.66	-9.90	ZnCO3
Talc	4.79	27.26	22.47	Mg3Si4O10(OH)2
Willemite	-4.66	11.44	16.10	Zn2SiO4
Witherite	-5.55	-14.14	-8.59	BaCO3
Zn(OH)2(e)	-5.13	6.37	11.50	Zn(OH)2

Initial solution 23.   4LPaula-1

-----------------------------Solution composition------------------------------

Elements	Molality	Moles
Alkalinity	3.384e-003	3.384e-003
Ba	1.683e-008	1.683e-008
Ca	3.184e-004	3.184e-004
Cl	1.283e-003	1.283e-003
Cu	6.483e-008	6.483e-008
K	4.625e-005	4.625e-005
Mg	6.778e-004	6.778e-004
Mn	4.316e-007	4.316e-007
Na	1.621e-003	1.621e-003
S(6)	7.604e-005	7.604e-005
Si	7.358e-002	7.358e-002
Zn	1.406e-006	1.406e-006

----------------------------Description of solution----------------------------

pH = 7.000
pe = 4.000
Activity of water = 0.999
Ionic strength = 5.229e-003
Mass of water (kg) = 1.000e+000
Total carbon (mol/kg) = 4.057e-003

```
                       Total CO2 (mol/kg)  =  4.057e-003
                      Temperature (deg C)  =  17.200
                  Electrical balance (eq)  = -1.155e-003
 Percent error, 100*(Cat-|An|)/(Cat+|An|)  = -13.76
                               Iterations  =  10
                                  Total H  = 1.113099e+002
                                  Total O  = 5.581224e+001
```

---------------------------Distribution of species---------------------------

Species		Molality	Activity	Log Molality	Log Activity	Log Gamma
H+		1.071e-007	1.000e-007	-6.970	-7.000	-0.030
OH-		5.839e-008	5.405e-008	-7.234	-7.267	-0.034
H2O		5.551e+001	9.986e-001	1.744	-0.001	0.000
Ba	1.683e-008					
Ba+2		1.622e-008	1.202e-008	-7.790	-7.920	-0.130
BaSO4		3.064e-010	3.068e-010	-9.514	-9.513	0.001
BaHCO3+		2.946e-010	2.729e-010	-9.531	-9.564	-0.033
BaCO3		6.329e-012	6.336e-012	-11.199	-11.198	0.001
BaOH+		4.390e-015	4.068e-015	-14.358	-14.391	-0.033
C(4)	4.057e-003					
HCO3-		3.261e-003	3.028e-003	-2.487	-2.519	-0.032
CO2		7.636e-004	7.645e-004	-3.117	-3.117	0.001
MgHCO3+		1.813e-005	1.680e-005	-4.742	-4.775	-0.033
CaHCO3+		8.255e-006	7.665e-006	-5.083	-5.116	-0.032
NaHCO3		2.552e-006	2.555e-006	-5.593	-5.593	0.001
CO3-2		1.606e-006	1.193e-006	-5.794	-5.923	-0.129
MgCO3		4.921e-007	4.927e-007	-6.308	-6.307	0.001
CaCO3		4.004e-007	4.008e-007	-6.398	-6.397	0.001
ZnHCO3+		2.853e-007	2.643e-007	-6.545	-6.578	-0.033
ZnCO3		1.649e-007	1.651e-007	-6.783	-6.782	0.001
MnHCO3+		7.225e-008	6.694e-008	-7.141	-7.174	-0.033
NaCO3-		2.403e-008	2.226e-008	-7.619	-7.652	-0.033
MnCO3		2.349e-008	2.351e-008	-7.629	-7.629	0.001
Zn(CO3)2-2		5.715e-009	4.212e-009	-8.243	-8.375	-0.132
BaHCO3+		2.946e-010	2.729e-010	-9.531	-9.564	-0.033
BaCO3		6.329e-012	6.336e-012	-11.199	-11.198	0.001
Ca	3.184e-004					
Ca+2		3.076e-004	2.284e-004	-3.512	-3.641	-0.129
CaHCO3+		8.255e-006	7.665e-006	-5.083	-5.116	-0.032
CaSO4		2.151e-006	2.154e-006	-5.667	-5.667	0.001
CaCO3		4.004e-007	4.008e-007	-6.398	-6.397	0.001
CaOH+		4.085e-010	3.785e-010	-9.389	-9.422	-0.033
CaHSO4+		1.245e-012	1.153e-012	-11.905	-11.938	-0.033
Cl	1.283e-003					
Cl-		1.283e-003	1.187e-003	-2.892	-2.925	-0.034
ZnCl+		1.680e-009	1.557e-009	-8.775	-8.808	-0.033
MnCl+		1.295e-009	1.200e-009	-8.888	-8.921	-0.033
ZnCl2		1.872e-012	1.875e-012	-11.728	-11.727	0.001
MnCl2		6.213e-013	6.221e-013	-12.207	-12.206	0.001
ZnCl3-		2.569e-015	2.381e-015	-14.590	-14.623	-0.033
MnCl3-		2.196e-016	2.035e-016	-15.658	-15.692	-0.033
ZnCl4-2		1.804e-018	1.330e-018	-17.744	-17.876	-0.132
Cu(1)	9.511e-010					
Cu+		9.511e-010	8.788e-010	-9.022	-9.056	-0.034
Cu(2)	6.388e-008					
Cu(OH)2		3.755e-008	3.760e-008	-7.425	-7.425	0.001
Cu+2		2.420e-008	1.805e-008	-7.616	-7.744	-0.127
CuOH+		1.945e-009	1.802e-009	-8.711	-8.744	-0.033
CuSO4		1.773e-010	1.775e-010	-9.751	-9.751	0.001
Cu(OH)3-		2.442e-014	2.262e-014	-13.612	-13.645	-0.033
Cu(OH)4-2		6.116e-020	4.508e-020	-19.214	-19.346	-0.132
H(0)	1.532e-025					
H2		7.658e-026	7.667e-026	-25.116	-25.115	0.001
K	4.625e-005					

```
   K+                     4.624e-005  4.280e-005    -4.335    -4.369    -0.034
   KSO4-                  1.437e-008  1.332e-008    -7.842    -7.876    -0.033
   KOH                    1.480e-012  1.482e-012   -11.830   -11.829     0.001
Mg               6.778e-004
   Mg+2                   6.544e-004  4.877e-004    -3.184    -3.312    -0.128
   MgHCO3+                1.813e-005  1.680e-005    -4.742    -4.775    -0.033
   MgSO4                  4.731e-006  4.737e-006    -5.325    -5.325     0.001
   MgCO3                  4.921e-007  4.927e-007    -6.308    -6.307     0.001
   MgOH+                  9.260e-009  8.580e-009    -8.033    -8.067    -0.033
Mn(2)            4.316e-007
   Mn+2                   3.326e-007  2.481e-007    -6.478    -6.605    -0.127
   MnHCO3+                7.225e-008  6.694e-008    -7.141    -7.174    -0.033
   MnCO3                  2.349e-008  2.351e-008    -7.629    -7.629     0.001
   MnSO4                  1.926e-009  1.928e-009    -8.715    -8.715     0.001
   MnCl+                  1.295e-009  1.200e-009    -8.888    -8.921    -0.033
   MnOH+                  3.577e-011  3.315e-011   -10.446   -10.480    -0.033
   MnCl2                  6.213e-013  6.221e-013   -12.207   -12.206     0.001
   MnCl3-                 2.196e-016  2.035e-016   -15.658   -15.692    -0.033
Mn(3)            4.728e-029
   Mn+3                   4.728e-029  2.380e-029   -28.325   -28.623    -0.298
Na               1.621e-003
   Na+                    1.618e-003  1.501e-003    -2.791    -2.824    -0.033
   NaHCO3                 2.552e-006  2.555e-006    -5.593    -5.593     0.001
   NaSO4-                 3.929e-007  3.640e-007    -6.406    -6.439    -0.033
   NaCO3-                 2.403e-008  2.226e-008    -7.619    -7.652    -0.033
   NaOH                   9.889e-011  9.901e-011   -10.005   -10.004     0.001
O(0)             0.000e+000
   O2                     0.000e+000  0.000e+000   -44.736   -44.735     0.001
S(6)             7.604e-005
   SO4-2                  6.874e-005  5.092e-005    -4.163    -4.293    -0.130
   MgSO4                  4.731e-006  4.737e-006    -5.325    -5.325     0.001
   CaSO4                  2.151e-006  2.154e-006    -5.667    -5.667     0.001
   NaSO4-                 3.929e-007  3.640e-007    -6.406    -6.439    -0.033
   KSO4-                  1.437e-008  1.332e-008    -7.842    -7.876    -0.033
   ZnSO4                  7.774e-009  7.784e-009    -8.109    -8.109     0.001
   MnSO4                  1.926e-009  1.928e-009    -8.715    -8.715     0.001
   HSO4-                  4.532e-010  4.200e-010    -9.344    -9.377    -0.033
   BaSO4                  3.064e-010  3.068e-010    -9.514    -9.513     0.001
   CuSO4                  1.773e-010  1.775e-010    -9.751    -9.751     0.001
   Zn(SO4)2-2             4.649e-012  3.427e-012   -11.333   -11.465    -0.132
   CaHSO4+                1.245e-012  1.153e-012   -11.905   -11.938    -0.033
Si               7.358e-002
   H4SiO4                 7.349e-002  7.358e-002    -1.134    -1.133     0.001
   H3SiO4-                8.800e-005  8.154e-005    -4.056    -4.089    -0.033
   H2SiO4-2               4.420e-011  3.258e-011   -10.355   -10.487    -0.132
Zn               1.406e-006
   Zn+2                   9.357e-007  6.935e-007    -6.029    -6.159    -0.130
   ZnHCO3+                2.853e-007  2.643e-007    -6.545    -6.578    -0.033
   ZnCO3                  1.649e-007  1.651e-007    -6.783    -6.782     0.001
   ZnSO4                  7.774e-009  7.784e-009    -8.109    -8.109     0.001
   Zn(CO3)2-2             5.715e-009  4.212e-009    -8.243    -8.375    -0.132
   ZnOH+                  4.464e-009  4.136e-009    -8.350    -8.383    -0.033
   ZnCl+                  1.680e-009  1.557e-009    -8.775    -8.808    -0.033
   Zn(OH)2                8.696e-010  8.706e-010    -9.061    -9.060     0.001
   Zn(SO4)2-2             4.649e-012  3.427e-012   -11.333   -11.465    -0.132
   ZnCl2                  1.872e-012  1.875e-012   -11.728   -11.727     0.001
   Zn(OH)3-               2.967e-014  2.749e-014   -13.528   -13.561    -0.033
   ZnCl3-                 2.569e-015  2.381e-015   -14.590   -14.623    -0.033
   ZnCl4-2                1.804e-018  1.330e-018   -17.744   -17.876    -0.132
   Zn(OH)4-2              5.904e-020  4.351e-020   -19.229   -19.361    -0.132

------------------------------Saturation indices-------------------------------

      Phase               SI log IAP  log KT

      Anhydrite        -3.60   -7.93   -4.34  CaSO4
      Aragonite        -1.27   -9.56   -8.29  CaCO3
```

Barite	-2.11	-12.21	-10.10	BaSO4
Calcite	-1.12	-9.56	-8.44	CaCO3
Chalcedony	2.51	-1.13	-3.64	SiO2
Chrysotile	-3.40	29.80	33.19	Mg3Si2O5(OH)4
CO2(g)	-1.75	-3.12	-1.37	CO2
Dolomite	-1.90	-18.80	-16.90	CaMg(CO3)2
Gypsum	-3.35	-7.94	-4.58	CaSO4:2H2O
H2(g)	-22.00	-25.12	-3.12	H2
H2O(g)	-1.72	-0.00	1.72	H2O
Halite	-7.31	-5.75	1.56	NaCl
Hausmannite	-18.83	44.18	63.01	Mn3O4
Manganite	-6.95	18.39	25.34	MnOOH
O2(g)	-41.81	-44.74	-2.92	O2
Pyrochroite	-7.81	7.39	15.20	Mn(OH)2
Pyrolusite	-13.27	29.39	42.66	MnO2
Quartz	2.97	-1.13	-4.10	SiO2
Rhodochrosite	-1.43	-12.53	-11.10	MnCO3
Sepiolite	2.01	17.98	15.97	Mg2Si3O7.5OH:3H2O
Sepiolite(d)	-0.68	17.98	18.66	Mg2Si3O7.5OH:3H2O
SiO2(a)	1.65	-1.13	-2.78	SiO2
Smithsonite	-2.17	-12.08	-9.91	ZnCO3
Talc	5.22	27.53	22.31	Mg3Si4O10(OH)2
Willemite	-1.44	14.55	15.99	Zn2SiO4
Witherite	-5.26	-13.84	-8.59	BaCO3
Zn(OH)2(e)	-3.66	7.84	11.50	Zn(OH)2

Initial solution 24.   4LSandra-1

-----------------------------Solution composition------------------------------

Elements	Molality	Moles
Alkalinity	9.783e-004	9.783e-004
Ba	1.462e-008	1.462e-008
Ca	1.027e-004	1.027e-004
Cl	6.783e-004	6.783e-004
Cu	3.159e-008	3.159e-008
F	5.230e-006	5.230e-006
K	2.002e-005	2.002e-005
Mg	2.275e-004	2.275e-004
Mn	1.535e-007	1.535e-007
Na	1.065e-003	1.065e-003
S(6)	3.437e-005	3.437e-005
Si	5.846e-002	5.846e-002
Zn	1.305e-007	1.305e-007

----------------------------Description of solution----------------------------

pH	= 7.000
pe	= 4.000
Activity of water	= 0.999
Ionic strength	= 2.092e-003
Mass of water (kg)	= 1.000e+000
Total carbon (mol/kg)	= 1.166e-003
Total CO2 (mol/kg)	= 1.166e-003
Temperature (deg C)	= 12.000
Electrical balance (eq)	= 1.550e-005
Percent error, 100*(Cat-|An|)/(Cat+|An|)	= 0.45
Iterations	= 9
Total H	= 1.112472e+002
Total O	= 5.574346e+001

----------------------------Distribution of species----------------------------

Species	Molality	Activity	Log Molality	Log Activity	Log Gamma

H+		1.047e-007	1.000e-007	-6.980	-7.000	-0.020
OH-		3.662e-008	3.484e-008	-7.436	-7.458	-0.022
H2O		5.551e+001	9.990e-001	1.744	-0.000	0.000
Ba	1.462e-008					
Ba+2		1.438e-008	1.183e-008	-7.842	-7.927	-0.085
BaSO4		1.608e-010	1.609e-010	-9.794	-9.793	0.000
BaHCO3+		6.927e-011	6.592e-011	-10.159	-10.181	-0.022
BaCO3		1.417e-012	1.418e-012	-11.849	-11.848	0.000
BaOH+		4.207e-015	4.004e-015	-14.376	-14.398	-0.022
C(4)	1.166e-003					
HCO3-		9.190e-004	8.753e-004	-3.037	-3.058	-0.021
CO2		2.438e-004	2.439e-004	-3.613	-3.613	0.000
MgHCO3+		1.916e-006	1.823e-006	-5.718	-5.739	-0.022
CaHCO3+		7.527e-007	7.170e-007	-6.123	-6.144	-0.021
NaHCO3		4.986e-007	4.988e-007	-6.302	-6.302	0.000
CO3-2		3.659e-007	3.012e-007	-6.437	-6.521	-0.084
MgCO3		4.357e-008	4.359e-008	-7.361	-7.361	0.000
CaCO3		3.488e-008	3.490e-008	-7.457	-7.457	0.000
ZnHCO3+		1.076e-008	1.024e-008	-7.968	-7.990	-0.022
MnHCO3+		9.492e-009	9.033e-009	-8.023	-8.044	-0.022
ZnCO3		5.582e-009	5.585e-009	-8.253	-8.253	0.000
NaCO3-		3.009e-009	2.864e-009	-8.522	-8.543	-0.022
MnCO3		2.769e-009	2.771e-009	-8.558	-8.557	0.000
BaHCO3+		6.927e-011	6.592e-011	-10.159	-10.181	-0.022
Zn(CO3)2-2		4.385e-011	3.597e-011	-10.358	-10.444	-0.086
BaCO3		1.417e-012	1.418e-012	-11.849	-11.848	0.000
Ca	1.027e-004					
Ca+2		1.015e-004	8.352e-005	-3.994	-4.078	-0.085
CaHCO3+		7.527e-007	7.170e-007	-6.123	-6.144	-0.021
CaSO4		3.981e-007	3.983e-007	-6.400	-6.400	0.000
CaCO3		3.488e-008	3.490e-008	-7.457	-7.457	0.000
CaF+		2.740e-009	2.608e-009	-8.562	-8.584	-0.022
CaOH+		1.455e-010	1.385e-010	-9.837	-9.859	-0.022
CaHSO4+		2.130e-013	2.027e-013	-12.672	-12.693	-0.022
Cl	6.783e-004					
Cl-		6.783e-004	6.453e-004	-3.169	-3.190	-0.022
MnCl+		3.199e-010	3.044e-010	-9.495	-9.517	-0.022
ZnCl+		9.313e-011	8.863e-011	-10.031	-10.052	-0.022
MnCl2		8.571e-014	8.575e-014	-13.067	-13.067	0.000
ZnCl2		5.668e-014	5.671e-014	-13.247	-13.246	0.000
ZnCl3-		3.977e-017	3.784e-017	-16.400	-16.422	-0.022
MnCl3-		1.602e-017	1.524e-017	-16.795	-16.817	-0.022
ZnCl4-2		1.340e-020	1.099e-020	-19.873	-19.959	-0.086
Cu(1)	4.445e-010					
Cu+		4.445e-010	4.226e-010	-9.352	-9.374	-0.022
Cu(2)	3.114e-008					
Cu(OH)2		1.905e-008	1.906e-008	-7.720	-7.720	0.000
Cu+2		1.109e-008	9.142e-009	-7.955	-8.039	-0.084
CuOH+		9.596e-010	9.132e-010	-9.018	-9.039	-0.022
CuSO4		4.609e-011	4.611e-011	-10.336	-10.336	0.000
Cu(OH)3-		1.206e-014	1.147e-014	-13.919	-13.940	-0.022
Cu(OH)4-2		2.788e-020	2.287e-020	-19.555	-19.641	-0.086
F	5.230e-006					
F-		5.174e-006	4.922e-006	-5.286	-5.308	-0.022
MgF+		4.946e-008	4.707e-008	-7.306	-7.327	-0.022
NaF		2.869e-009	2.870e-009	-8.542	-8.542	0.000
CaF+		2.740e-009	2.608e-009	-8.562	-8.584	-0.022
HF		5.877e-010	5.880e-010	-9.231	-9.231	0.000
MnF+		4.143e-012	3.943e-012	-11.383	-11.404	-0.022
HF2-		1.032e-014	9.824e-015	-13.986	-14.008	-0.022
SiF6-2		5.381e-031	4.414e-031	-30.269	-30.355	-0.086
H(0)	1.620e-025					
H2		8.102e-026	8.106e-026	-25.091	-25.091	0.000
K	2.002e-005					
K+		2.002e-005	1.904e-005	-4.699	-4.720	-0.022
KSO4-		3.010e-009	2.865e-009	-8.521	-8.543	-0.022
KOH		6.593e-013	6.596e-013	-12.181	-12.181	0.000

```
Mg               2.275e-004
   Mg+2                   2.246e-004  1.851e-004    -3.649    -3.732    -0.084
   MgHCO3+                1.916e-006  1.823e-006    -5.718    -5.739    -0.022
   MgSO4                  8.295e-007  8.299e-007    -6.081    -6.081     0.000
   MgF+                   4.946e-008  4.707e-008    -7.306    -7.327    -0.022
   MgCO3                  4.357e-008  4.359e-008    -7.361    -7.361     0.000
   MgOH+                  2.068e-009  1.968e-009    -8.685    -8.706    -0.022
Mn(2)            1.535e-007
   Mn+2                   1.404e-007  1.158e-007    -6.853    -6.936    -0.084
   MnHCO3+                9.492e-009  9.033e-009    -8.023    -8.044    -0.022
   MnCO3                  2.769e-009  2.771e-009    -8.558    -8.557     0.000
   MnSO4                  4.309e-010  4.312e-010    -9.366    -9.365     0.000
   MnCl+                  3.199e-010  3.044e-010    -9.495    -9.517    -0.022
   MnOH+                  1.032e-011  9.818e-012   -10.986   -11.008    -0.022
   MnF+                   4.143e-012  3.943e-012   -11.383   -11.404    -0.022
   MnCl2                  8.571e-014  8.575e-014   -13.067   -13.067     0.000
   MnCl3-                 1.602e-017  1.524e-017   -16.795   -16.817    -0.022
Mn(3)            7.676e-030
   Mn+3                   7.676e-030  4.915e-030   -29.115   -29.308    -0.194
Na               1.065e-003
   Na+                    1.065e-003  1.013e-003    -2.973    -2.994    -0.021
   NaHCO3                 4.986e-007  4.988e-007    -6.302    -6.302     0.000
   NaSO4-                 1.329e-007  1.265e-007    -6.877    -6.898    -0.022
   NaCO3-                 3.009e-009  2.864e-009    -8.522    -8.543    -0.022
   NaF                    2.869e-009  2.870e-009    -8.542    -8.542     0.000
   NaOH                   6.685e-011  6.689e-011   -10.175   -10.175     0.000
O(0)             0.000e+000
   O2                     0.000e+000  0.000e+000   -46.585   -46.585     0.000
S(6)             3.437e-005
   SO4-2                  3.301e-005  2.714e-005    -4.481    -4.566    -0.085
   MgSO4                  8.295e-007  8.299e-007    -6.081    -6.081     0.000
   CaSO4                  3.981e-007  3.983e-007    -6.400    -6.400     0.000
   NaSO4-                 1.329e-007  1.265e-007    -6.877    -6.898    -0.022
   KSO4-                  3.010e-009  2.865e-009    -8.521    -8.543    -0.022
   ZnSO4                  5.321e-010  5.324e-010    -9.274    -9.274     0.000
   MnSO4                  4.309e-010  4.312e-010    -9.366    -9.365     0.000
   HSO4-                  2.121e-010  2.019e-010    -9.673    -9.695    -0.022
   BaSO4                  1.608e-010  1.609e-010    -9.794    -9.793     0.000
   CuSO4                  4.609e-011  4.611e-011   -10.336   -10.336     0.000
   CaHSO4+                2.130e-013  2.027e-013   -12.672   -12.693    -0.022
   Zn(SO4)2-2             1.590e-013  1.304e-013   -12.799   -12.885    -0.086
Si               5.846e-002
   H4SiO4                 5.841e-002  5.844e-002    -1.234    -1.233     0.000
   H3SiO4-                5.530e-005  5.262e-005    -4.257    -4.279    -0.022
   H2SiO4-2               1.748e-011  1.434e-011   -10.758   -10.844    -0.086
   SiF6-2                 5.381e-031  4.414e-031   -30.269   -30.355    -0.086
Zn               1.305e-007
   Zn+2                   1.130e-007  9.292e-008    -6.947    -7.032    -0.085
   ZnHCO3+                1.076e-008  1.024e-008    -7.968    -7.990    -0.022
   ZnCO3                  5.582e-009  5.585e-009    -8.253    -8.253     0.000
   ZnSO4                  5.321e-010  5.324e-010    -9.274    -9.274     0.000
   ZnOH+                  3.814e-010  3.630e-010    -9.419    -9.440    -0.022
   Zn(OH)2                1.167e-010  1.167e-010    -9.933    -9.933     0.000
   ZnCl+                  9.313e-011  8.863e-011   -10.031   -10.052    -0.022
   Zn(CO3)2-2             4.385e-011  3.597e-011   -10.358   -10.444    -0.086
   Zn(SO4)2-2             1.590e-013  1.304e-013   -12.799   -12.885    -0.086
   ZnCl2                  5.668e-014  5.671e-014   -13.247   -13.246     0.000
   Zn(OH)3-               3.875e-015  3.688e-015   -14.412   -14.433    -0.022
   ZnCl3-                 3.977e-017  3.784e-017   -16.400   -16.422    -0.022
   ZnCl4-2                1.340e-020  1.099e-020   -19.873   -19.959    -0.086
   Zn(OH)4-2              7.117e-021  5.838e-021   -20.148   -20.234    -0.086

------------------------------Saturation indices-------------------------------

      Phase               SI log IAP  log KT

      Anhydrite        -4.31   -8.64   -4.33  CaSO4
```

Aragonite	-2.33	-10.60	-8.26	CaCO3
Barite	-2.29	-12.49	-10.20	BaSO4
Calcite	-2.18	-10.60	-8.42	CaCO3
Chalcedony	2.48	-1.23	-3.71	SiO2
Chrysotile	-5.55	28.34	33.88	Mg3Si2O5(OH)4
CO2(g)	-2.31	-3.61	-1.30	CO2
Dolomite	-4.08	-20.85	-16.77	CaMg(CO3)2
Fluorite	-3.93	-14.69	-10.77	CaF2
Gypsum	-4.06	-8.65	-4.59	CaSO4:2H2O
H2(g)	-22.00	-25.09	-3.09	H2
H2O(g)	-1.86	-0.00	1.86	H2O
Halite	-7.74	-6.18	1.55	NaCl
Hausmannite	-21.20	43.19	64.39	Mn3O4
Manganite	-7.28	18.06	25.34	MnOOH
O2(g)	-43.69	-46.59	-2.90	O2
Pyrochroite	-8.14	7.06	15.20	Mn(OH)2
Pyrolusite	-14.49	29.06	43.56	MnO2
Quartz	2.95	-1.23	-4.18	SiO2
Rhodochrosite	-2.38	-13.46	-11.08	MnCO3
Sepiolite	0.72	16.84	16.12	Mg2Si3O7.5OH:3H2O
Sepiolite(d)	-1.82	16.84	18.66	Mg2Si3O7.5OH:3H2O
SiO2(a)	1.59	-1.23	-2.82	SiO2
Smithsonite	-3.70	-13.55	-9.85	ZnCO3
Talc	2.92	25.87	22.95	Mg3Si4O10(OH)2
Willemite	-3.74	12.70	16.45	Zn2SiO4
Witherite	-5.83	-14.45	-8.62	BaCO3
Zn(OH)2(e)	-4.53	6.97	11.50	Zn(OH)2

Initial solution 25.   4LSara-1

----------------------------Solution composition-----------------------------

Elements	Molality	Moles
Alkalinity	2.577e-003	2.577e-003
Ba	3.364e-008	3.364e-008
Ca	3.133e-004	3.133e-004
Cl	2.514e-003	2.514e-003
Cu	6.323e-008	6.323e-008
F	7.138e-006	7.138e-006
K	4.393e-005	4.393e-005
Mg	1.070e-003	1.070e-003
Mn	3.236e-007	3.236e-007
Na	1.905e-003	1.905e-003
S(6)	1.114e-004	1.114e-004
Si	6.854e-002	6.854e-002
Zn	2.120e-007	2.120e-007

---------------------------Description of solution----------------------------

pH	= 7.000
pe	= 4.000
Activity of water	= 0.999
Ionic strength	= 6.402e-003
Mass of water (kg)	= 1.000e+000
Total carbon (mol/kg)	= 3.059e-003
Total CO2 (mol/kg)	= 3.059e-003
Temperature (deg C)	= 17.600
Electrical balance (eq)	= -6.043e-004
Percent error, 100*(Cat-|An|)/(Cat+|An|)	= -6.09
Iterations	= 9
Total H	= 1.112890e+002
Total O	= 5.578944e+001

---------------------------Distribution of species----------------------------

Log   Log   Log

Species		Molality	Activity	Molality	Activity	Gamma
H+		1.078e-007	1.000e-007	-6.967	-7.000	-0.033
OH-		6.080e-008	5.585e-008	-7.216	-7.253	-0.037
H2O		5.551e+001	9.987e-001	1.744	-0.001	0.000
Ba	3.364e-008					
Ba+2		3.238e-008	2.332e-008	-7.490	-7.632	-0.142
BaSO4		8.206e-010	8.218e-010	-9.086	-9.085	0.001
BaHCO3+		4.367e-010	4.017e-010	-9.360	-9.396	-0.036
BaCO3		9.360e-012	9.374e-012	-11.029	-11.028	0.001
BaOH+		8.581e-015	7.893e-015	-14.066	-14.103	-0.036
C(4)	3.059e-003					
HCO3-		2.460e-003	2.268e-003	-2.609	-2.644	-0.035
CO2		5.678e-004	5.687e-004	-3.246	-3.245	0.001
MgHCO3+		2.114e-005	1.944e-005	-4.675	-4.711	-0.036
CaHCO3+		6.036e-006	5.566e-006	-5.219	-5.254	-0.035
NaHCO3		2.230e-006	2.233e-006	-5.652	-5.651	0.001
CO3-2		1.249e-006	9.025e-007	-5.903	-6.045	-0.141
MgCO3		5.778e-007	5.786e-007	-6.238	-6.238	0.001
CaCO3		2.927e-007	2.931e-007	-6.534	-6.533	0.001
MnHCO3+		4.207e-008	3.870e-008	-7.376	-7.412	-0.036
ZnHCO3+		3.443e-008	3.167e-008	-7.463	-7.499	-0.036
NaCO3-		2.182e-008	2.007e-008	-7.661	-7.698	-0.036
ZnCO3		1.995e-008	1.998e-008	-7.700	-7.699	0.001
MnCO3		1.370e-008	1.373e-008	-7.863	-7.862	0.001
Zn(CO3)2-2		5.386e-010	3.855e-010	-9.269	-9.414	-0.145
BaHCO3+		4.367e-010	4.017e-010	-9.360	-9.396	-0.036
BaCO3		9.360e-012	9.374e-012	-11.029	-11.028	0.001
Ca	3.133e-004					
Ca+2		3.041e-004	2.196e-004	-3.517	-3.658	-0.141
CaHCO3+		6.036e-006	5.566e-006	-5.219	-5.254	-0.035
CaSO4		2.865e-006	2.869e-006	-5.543	-5.542	0.001
CaCO3		2.927e-007	2.931e-007	-6.534	-6.533	0.001
CaF+		1.092e-008	1.004e-008	-7.962	-7.998	-0.036
CaOH+		3.957e-010	3.639e-010	-9.403	-9.439	-0.036
CaHSO4+		1.678e-012	1.543e-012	-11.775	-11.812	-0.036
Cl	2.514e-003					
Cl-		2.514e-003	2.310e-003	-2.600	-2.636	-0.037
MnCl+		1.958e-009	1.801e-009	-8.708	-8.744	-0.036
ZnCl+		5.366e-010	4.935e-010	-9.270	-9.307	-0.036
MnCl2		1.814e-012	1.816e-012	-11.741	-11.741	0.001
ZnCl2		1.156e-012	1.158e-012	-11.937	-11.936	0.001
ZnCl3-		3.117e-015	2.867e-015	-14.506	-14.543	-0.036
MnCl3-		1.256e-015	1.155e-015	-14.901	-14.937	-0.036
ZnCl4-2		4.366e-018	3.125e-018	-17.360	-17.505	-0.145
Cu(1)	9.279e-010					
Cu+		9.279e-010	8.506e-010	-9.032	-9.070	-0.038
Cu(2)	6.230e-008					
Cu(OH)2		3.620e-008	3.625e-008	-7.441	-7.441	0.001
Cu+2		2.397e-008	1.740e-008	-7.620	-7.759	-0.139
CuOH+		1.890e-009	1.738e-009	-8.724	-8.760	-0.036
CuSO4		2.366e-010	2.370e-010	-9.626	-9.625	0.001
Cu(OH)3-		2.372e-014	2.182e-014	-13.625	-13.661	-0.036
Cu(OH)4-2		6.074e-020	4.347e-020	-19.217	-19.362	-0.145
F	7.138e-006					
F-		6.824e-006	6.268e-006	-5.166	-5.203	-0.037
MgF+		2.954e-007	2.717e-007	-6.530	-6.566	-0.036
CaF+		1.092e-008	1.004e-008	-7.962	-7.998	-0.036
NaF		6.306e-009	6.316e-009	-8.200	-8.200	0.001
HF		8.234e-010	8.246e-010	-9.084	-9.084	0.001
MnF+		9.027e-012	8.303e-012	-11.044	-11.081	-0.036
HF2-		2.022e-014	1.860e-014	-13.694	-13.731	-0.036
SiF6-2		1.778e-030	1.273e-030	-29.750	-29.895	-0.145
H(0)	1.525e-025					
H2		7.624e-026	7.635e-026	-25.118	-25.117	0.001
K	4.393e-005					
K+		4.391e-005	4.034e-005	-4.357	-4.394	-0.037

KSO4-		1.898e-008	1.746e-008	-7.722	-7.758	-0.036
KOH		1.395e-012	1.397e-012	-11.855	-11.855	0.001
Mg	1.070e-003					
Mg+2		1.038e-003	7.527e-004	-2.984	-3.123	-0.139
MgHCO3+		2.114e-005	1.944e-005	-4.675	-4.711	-0.036
MgSO4		1.019e-005	1.020e-005	-4.992	-4.991	0.001
MgCO3		5.778e-007	5.786e-007	-6.238	-6.238	0.001
MgF+		2.954e-007	2.717e-007	-6.530	-6.566	-0.036
MgOH+		1.496e-008	1.376e-008	-7.825	-7.862	-0.036
Mn(2)	3.236e-007					
Mn+2		2.638e-007	1.914e-007	-6.579	-6.718	-0.139
MnHCO3+		4.207e-008	3.870e-008	-7.376	-7.412	-0.036
MnCO3		1.370e-008	1.373e-008	-7.863	-7.862	0.001
MnSO4		2.068e-009	2.071e-009	-8.684	-8.684	0.001
MnCl+		1.958e-009	1.801e-009	-8.708	-8.744	-0.036
MnOH+		2.878e-011	2.648e-011	-10.541	-10.577	-0.036
MnF+		9.027e-012	8.303e-012	-11.044	-11.081	-0.036
MnCl2		1.814e-012	1.816e-012	-11.741	-11.741	0.001
MnCl3-		1.256e-015	1.155e-015	-14.901	-14.937	-0.036
Mn(3)	4.145e-029					
Mn+3		4.145e-029	1.953e-029	-28.382	-28.709	-0.327
Na	1.905e-003					
Na+		1.902e-003	1.751e-003	-2.721	-2.757	-0.036
NaHCO3		2.230e-006	2.233e-006	-5.652	-5.651	0.001
NaSO4-		6.392e-007	5.879e-007	-6.194	-6.231	-0.036
NaCO3-		2.182e-008	2.007e-008	-7.661	-7.698	-0.036
NaF		6.306e-009	6.316e-009	-8.200	-8.200	0.001
NaOH		1.154e-010	1.155e-010	-9.938	-9.937	0.001
O(0)	0.000e+000					
O2		0.000e+000	0.000e+000	-44.596	-44.596	0.001
S(6)	1.114e-004					
SO4-2		9.764e-005	7.030e-005	-4.010	-4.153	-0.143
MgSO4		1.019e-005	1.020e-005	-4.992	-4.991	0.001
CaSO4		2.865e-006	2.869e-006	-5.543	-5.542	0.001
NaSO4-		6.392e-007	5.879e-007	-6.194	-6.231	-0.036
KSO4-		1.898e-008	1.746e-008	-7.722	-7.758	-0.036
MnSO4		2.068e-009	2.071e-009	-8.684	-8.684	0.001
ZnSO4		1.722e-009	1.725e-009	-8.764	-8.763	0.001
BaSO4		8.206e-010	8.218e-010	-9.086	-9.085	0.001
HSO4-		6.355e-010	5.845e-010	-9.197	-9.233	-0.036
CuSO4		2.366e-010	2.370e-010	-9.626	-9.625	0.001
CaHSO4+		1.678e-012	1.543e-012	-11.775	-11.812	-0.036
Zn(SO4)2-2		1.460e-012	1.045e-012	-11.836	-11.981	-0.145
Si	6.854e-002					
H4SiO4		6.846e-002	6.856e-002	-1.165	-1.164	0.001
H3SiO4-		8.388e-005	7.715e-005	-4.076	-4.113	-0.036
H2SiO4-2		4.431e-011	3.172e-011	-10.353	-10.499	-0.145
SiF6-2		1.778e-030	1.273e-030	-29.750	-29.895	-0.145
Zn	2.120e-007					
Zn+2		1.540e-007	1.109e-007	-6.813	-6.955	-0.142
ZnHCO3+		3.443e-008	3.167e-008	-7.463	-7.499	-0.036
ZnCO3		1.995e-008	1.998e-008	-7.700	-7.699	0.001
ZnSO4		1.722e-009	1.725e-009	-8.764	-8.763	0.001
ZnOH+		7.427e-010	6.831e-010	-9.129	-9.165	-0.036
Zn(CO3)2-2		5.386e-010	3.855e-010	-9.269	-9.414	-0.145
ZnCl+		5.366e-010	4.935e-010	-9.270	-9.307	-0.036
Zn(OH)2		1.391e-010	1.393e-010	-9.857	-9.856	0.001
Zn(SO4)2-2		1.460e-012	1.045e-012	-11.836	-11.981	-0.145
ZnCl2		1.156e-012	1.158e-012	-11.937	-11.936	0.001
Zn(OH)3-		4.783e-015	4.399e-015	-14.320	-14.357	-0.036
ZnCl3-		3.117e-015	2.867e-015	-14.506	-14.543	-0.036
ZnCl4-2		4.366e-018	3.125e-018	-17.360	-17.505	-0.145
Zn(OH)4-2		9.728e-021	6.963e-021	-20.012	-20.157	-0.145

------------------------------Saturation indices-------------------------------

Phase	SI	log IAP	log KT

Anhydrite	-3.47	-7.81	-4.34	CaSO4
Aragonite	-1.41	-9.70	-8.29	CaCO3
Barite	-1.69	-11.79	-10.10	BaSO4
Calcite	-1.26	-9.70	-8.44	CaCO3
Chalcedony	2.48	-1.16	-3.64	SiO2
Chrysotile	-2.84	30.30	33.14	Mg3Si2O5(OH)4
CO2(g)	-1.87	-3.25	-1.38	CO2
Dolomite	-1.96	-18.87	-16.91	CaMg(CO3)2
Fluorite	-3.37	-14.06	-10.69	CaF2
Gypsum	-3.23	-7.81	-4.58	CaSO4:2H2O
H2(g)	-22.00	-25.12	-3.12	H2
H2O(g)	-1.71	-0.00	1.71	H2O
Halite	-6.96	-5.39	1.56	NaCl
Hausmannite	-19.06	43.84	62.91	Mn3O4
Manganite	-7.06	18.28	25.34	MnOOH
O2(g)	-41.67	-44.60	-2.93	O2
Pyrochroite	-7.92	7.28	15.20	Mn(OH)2
Pyrolusite	-13.31	29.28	42.59	MnO2
Quartz	2.93	-1.16	-4.09	SiO2
Rhodochrosite	-1.66	-12.76	-11.10	MnCO3
Sepiolite	2.30	18.26	15.96	Mg2Si3O7.5OH:3H2O
Sepiolite(d)	-0.40	18.26	18.66	Mg2Si3O7.5OH:3H2O
SiO2(a)	1.61	-1.16	-2.77	SiO2
Smithsonite	-3.08	-13.00	-9.92	ZnCO3
Talc	5.71	27.98	22.26	Mg3Si4O10(OH)2
Willemite	-3.03	12.93	15.95	Zn2SiO4
Witherite	-5.09	-13.68	-8.58	BaCO3
Zn(OH)2(e)	-4.46	7.04	11.50	Zn(OH)2

Initial solution 26.   4LSilvia-1

-----------------------------Solution composition------------------------------

Elements	Molality	Moles
Alkalinity	3.250e-003	3.250e-003
Ba	2.192e-009	2.192e-009
Ca	4.456e-004	4.456e-004
Cl	1.812e-003	1.812e-003
Cu	3.947e-008	3.947e-008
F	2.271e-006	2.271e-006
K	4.541e-005	4.541e-005
Mg	1.213e-003	1.213e-003
Mn	9.679e-008	9.679e-008
Na	2.025e-003	2.025e-003
S(6)	1.401e-004	1.401e-004
Si	4.842e-002	4.842e-002
Zn	7.520e-008	7.520e-008

----------------------------Description of solution----------------------------

pH	=	7.000
pe	=	4.000
Activity of water	=	0.999
Ionic strength	=	7.011e-003
Mass of water (kg)	=	1.000e+000
Total carbon (mol/kg)	=	3.988e-003
Total CO2 (mol/kg)	=	3.988e-003
Temperature (deg C)	=	13.000
Electrical balance (eq)	=	4.404e-005
Percent error, 100*(Cat-|An|)/(Cat+|An|)	=	0.42
Iterations	=	10
Total H	=	1.112093e+002
Total O	=	5.571165e+001

----------------------------Distribution of species----------------------------

	Species	Molality	Activity	Log Molality	Log Activity	Log Gamma
	H+	1.080e-007	1.000e-007	-6.966	-7.000	-0.034
	OH-	4.148e-008	3.799e-008	-7.382	-7.420	-0.038
	H2O	5.551e+001	9.990e-001	1.744	-0.000	0.000
Ba	2.192e-009					
	Ba+2	2.095e-009	1.493e-009	-8.679	-8.826	-0.147
	BaSO4	6.493e-011	6.503e-011	-10.188	-10.187	0.001
	BaHCO3+	3.100e-011	2.843e-011	-10.509	-10.546	-0.038
	BaCO3	6.203e-013	6.213e-013	-12.207	-12.207	0.001
	BaOH+	5.509e-016	5.053e-016	-15.259	-15.296	-0.038
C(4)	3.988e-003					
	HCO3-	3.153e-003	2.899e-003	-2.501	-2.538	-0.036
	CO2	7.900e-004	7.913e-004	-3.102	-3.102	0.001
	MgHCO3+	2.991e-005	2.743e-005	-4.524	-4.562	-0.038
	CaHCO3+	9.758e-006	8.972e-006	-5.011	-5.047	-0.036
	NaHCO3	3.019e-006	3.024e-006	-5.520	-5.519	0.001
	CO3-2	1.434e-006	1.025e-006	-5.843	-5.989	-0.146
	MgCO3	6.822e-007	6.833e-007	-6.166	-6.165	0.001
	CaCO3	4.408e-007	4.415e-007	-6.356	-6.355	0.001
	NaCO3-	2.055e-008	1.885e-008	-7.687	-7.725	-0.038
	MnHCO3+	1.536e-008	1.409e-008	-7.814	-7.851	-0.038
	ZnHCO3+	1.467e-008	1.346e-008	-7.833	-7.871	-0.038
	ZnCO3	7.532e-009	7.544e-009	-8.123	-8.122	0.001
	MnCO3	4.434e-009	4.441e-009	-8.353	-8.353	0.001
	Zn(CO3)2-2	2.337e-010	1.654e-010	-9.631	-9.782	-0.150
	BaHCO3+	3.100e-011	2.843e-011	-10.509	-10.546	-0.038
	BaCO3	6.203e-013	6.213e-013	-12.207	-12.207	0.001
Ca	4.456e-004					
	Ca+2	4.306e-004	3.076e-004	-3.366	-3.512	-0.146
	CaHCO3+	9.758e-006	8.972e-006	-5.011	-5.047	-0.036
	CaSO4	4.740e-006	4.748e-006	-5.324	-5.324	0.001
	CaCO3	4.408e-007	4.415e-007	-6.356	-6.355	0.001
	CaF+	4.334e-009	3.975e-009	-8.363	-8.401	-0.038
	CaOH+	5.561e-010	5.100e-010	-9.255	-9.292	-0.038
	CaHSO4+	2.659e-012	2.439e-012	-11.575	-11.613	-0.038
Cl	1.812e-003					
	Cl-	1.812e-003	1.660e-003	-2.742	-2.780	-0.038
	MnCl+	4.019e-010	3.686e-010	-9.396	-9.433	-0.038
	ZnCl+	1.035e-010	9.491e-011	-9.985	-10.023	-0.038
	MnCl2	2.666e-013	2.671e-013	-12.574	-12.573	0.001
	ZnCl2	1.566e-013	1.569e-013	-12.805	-12.804	0.001
	ZnCl3-	2.954e-016	2.710e-016	-15.530	-15.567	-0.038
	MnCl3-	1.331e-016	1.221e-016	-15.876	-15.913	-0.038
	ZnCl4-2	2.885e-019	2.041e-019	-18.540	-18.690	-0.150
Cu(1)	5.525e-010					
	Cu+	5.525e-010	5.049e-010	-9.258	-9.297	-0.039
Cu(2)	3.892e-008					
	Cu(OH)2	2.251e-008	2.255e-008	-7.648	-7.647	0.001
	Cu+2	1.506e-008	1.081e-008	-7.822	-7.966	-0.144
	CuOH+	1.178e-009	1.080e-009	-8.929	-8.967	-0.038
	CuSO4	1.757e-010	1.760e-010	-9.755	-9.754	0.001
	Cu(OH)3-	1.480e-014	1.357e-014	-13.830	-13.867	-0.038
	Cu(OH)4-2	3.823e-020	2.705e-020	-19.418	-19.568	-0.150
F	2.271e-006					
	F-	2.168e-006	1.986e-006	-5.664	-5.702	-0.038
	MgF+	9.578e-008	8.785e-008	-7.019	-7.056	-0.038
	CaF+	4.334e-009	3.975e-009	-8.363	-8.401	-0.038
	NaF	2.117e-009	2.120e-009	-8.674	-8.674	0.001
	HF	2.409e-010	2.413e-010	-9.618	-9.617	0.001
	MnF+	8.168e-013	7.491e-013	-12.088	-12.125	-0.038
	HF2-	1.793e-015	1.644e-015	-14.746	-14.784	-0.038
	SiF6-2	2.017e-033	1.427e-033	-32.695	-32.846	-0.150
H(0)	1.601e-025					
	H2	8.005e-026	8.018e-026	-25.097	-25.096	0.001

K		4.541e-005				
	K+	4.539e-005	4.158e-005	-4.343	-4.381	-0.038
	KSO4-	2.227e-008	2.042e-008	-7.652	-7.690	-0.038
	KOH	1.438e-012	1.440e-012	-11.842	-11.842	0.001
Mg		1.213e-003				
	Mg+2	1.170e-003	8.398e-004	-2.932	-3.076	-0.144
	MgHCO3+	2.991e-005	2.743e-005	-4.524	-4.562	-0.038
	MgSO4	1.238e-005	1.240e-005	-4.907	-4.907	0.001
	MgCO3	6.822e-007	6.833e-007	-6.166	-6.165	0.001
	MgF+	9.578e-008	8.785e-008	-7.019	-7.056	-0.038
	MgOH+	1.074e-008	9.849e-009	-7.969	-8.007	-0.038
Mn(2)		9.679e-008				
	Mn+2	7.592e-008	5.453e-008	-7.120	-7.263	-0.144
	MnHCO3+	1.536e-008	1.409e-008	-7.814	-7.851	-0.038
	MnCO3	4.434e-009	4.441e-009	-8.353	-8.353	0.001
	MnSO4	6.630e-010	6.640e-010	-9.179	-9.178	0.001
	MnCl+	4.019e-010	3.686e-010	-9.396	-9.433	-0.038
	MnOH+	5.509e-012	5.053e-012	-11.259	-11.296	-0.038
	MnF+	8.168e-013	7.491e-013	-12.088	-12.125	-0.038
	MnCl2	2.666e-013	2.671e-013	-12.574	-12.573	0.001
	MnCl3-	1.331e-016	1.221e-016	-15.876	-15.913	-0.038
Mn(3)		5.909e-030				
	Mn+3	5.909e-030	2.714e-030	-29.228	-29.566	-0.338
Na		2.025e-003				
	Na+	2.021e-003	1.855e-003	-2.694	-2.732	-0.037
	NaHCO3	3.019e-006	3.024e-006	-5.520	-5.519	0.001
	NaSO4-	8.141e-007	7.467e-007	-6.089	-6.127	-0.038
	NaCO3-	2.055e-008	1.885e-008	-7.687	-7.725	-0.038
	NaF	2.117e-009	2.120e-009	-8.674	-8.674	0.001
	NaOH	1.223e-010	1.225e-010	-9.913	-9.912	0.001
O(0)		0.000e+000				
	O2	0.000e+000	0.000e+000	-46.225	-46.224	0.001
S(6)		1.401e-004				
	SO4-2	1.221e-004	8.693e-005	-3.913	-4.061	-0.148
	MgSO4	1.238e-005	1.240e-005	-4.907	-4.907	0.001
	CaSO4	4.740e-006	4.748e-006	-5.324	-5.324	0.001
	NaSO4-	8.141e-007	7.467e-007	-6.089	-6.127	-0.038
	KSO4-	2.227e-008	2.042e-008	-7.652	-7.690	-0.038
	HSO4-	7.189e-010	6.593e-010	-9.143	-9.181	-0.038
	ZnSO4	6.815e-010	6.826e-010	-9.167	-9.166	0.001
	MnSO4	6.630e-010	6.640e-010	-9.179	-9.178	0.001
	CuSO4	1.757e-010	1.760e-010	-9.755	-9.754	0.001
	BaSO4	6.493e-011	6.503e-011	-10.188	-10.187	0.001
	CaHSO4+	2.659e-012	2.439e-012	-11.575	-11.613	-0.038
	Zn(SO4)2-2	7.505e-013	5.310e-013	-12.125	-12.275	-0.150
Si		4.842e-002				
	H4SiO4	4.837e-002	4.845e-002	-1.315	-1.315	0.001
	H3SiO4-	4.957e-005	4.546e-005	-4.305	-4.342	-0.038
	H2SiO4-2	1.888e-011	1.336e-011	-10.724	-10.874	-0.150
	SiF6-2	2.017e-033	1.427e-033	-32.695	-32.846	-0.150
Zn		7.520e-008				
	Zn+2	5.176e-008	3.688e-008	-7.286	-7.433	-0.147
	ZnHCO3+	1.467e-008	1.346e-008	-7.833	-7.871	-0.038
	ZnCO3	7.532e-009	7.544e-009	-8.123	-8.122	0.001
	ZnSO4	6.815e-010	6.826e-010	-9.167	-9.166	0.001
	Zn(CO3)2-2	2.337e-010	1.654e-010	-9.631	-9.782	-0.150
	ZnOH+	1.706e-010	1.565e-010	-9.768	-9.806	-0.038
	ZnCl+	1.035e-010	9.491e-011	-9.985	-10.023	-0.038
	Zn(OH)2	4.626e-011	4.634e-011	-10.335	-10.334	0.001
	Zn(SO4)2-2	7.505e-013	5.310e-013	-12.125	-12.275	-0.150
	ZnCl2	1.566e-013	1.569e-013	-12.805	-12.804	0.001
	Zn(OH)3-	1.596e-015	1.464e-015	-14.797	-14.835	-0.038
	ZnCl3-	2.954e-016	2.710e-016	-15.530	-15.567	-0.038
	ZnCl4-2	2.885e-019	2.041e-019	-18.540	-18.690	-0.150
	Zn(OH)4-2	3.275e-021	2.318e-021	-20.485	-20.635	-0.150

------------------------------Saturation indices-------------------------------

Phase	SI	log IAP	log KT	
Anhydrite	-3.24	-7.57	-4.33	CaSO4
Aragonite	-1.23	-9.50	-8.27	CaCO3
Barite	-2.70	-12.89	-10.18	BaSO4
Calcite	-1.08	-9.50	-8.42	CaCO3
Chalcedony	2.38	-1.31	-3.70	SiO2
Chrysotile	-3.61	30.14	33.75	Mg3Si2O5(OH)4
CO2(g)	-1.79	-3.10	-1.31	CO2
Dolomite	-1.77	-18.57	-16.80	CaMg(CO3)2
Fluorite	-4.16	-14.92	-10.75	CaF2
Gypsum	-2.99	-7.57	-4.59	CaSO4:2H2O
H2(g)	-22.00	-25.10	-3.10	H2
H2O(g)	-1.83	-0.00	1.83	H2O
Halite	-7.07	-5.51	1.55	NaCl
Hausmannite	-21.92	42.21	64.12	Mn3O4
Manganite	-7.60	17.74	25.34	MnOOH
O2(g)	-43.32	-46.22	-2.90	O2
Pyrochroite	-8.46	6.74	15.20	Mn(OH)2
Pyrolusite	-14.65	28.74	43.38	MnO2
Quartz	2.85	-1.31	-4.16	SiO2
Rhodochrosite	-2.17	-13.25	-11.09	MnCO3
Sepiolite	1.82	17.90	16.09	Mg2Si3O7.5OH:3H2O
Sepiolite(d)	-0.76	17.90	18.66	Mg2Si3O7.5OH:3H2O
SiO2(a)	1.50	-1.31	-2.81	SiO2
Smithsonite	-3.56	-13.42	-9.87	ZnCO3
Talc	4.69	27.52	22.82	Mg3Si4O10(OH)2
Willemite	-4.54	11.82	16.36	Zn2SiO4
Witherite	-6.21	-14.82	-8.61	BaCO3
Zn(OH)2(e)	-4.93	6.57	11.50	Zn(OH)2

Initial solution 27.   4LSusan-1

----------------------------Solution composition----------------------------

Elements	Molality	Moles
Alkalinity	1.136e-003	1.136e-003
Ba	2.334e-008	2.334e-008
Ca	1.025e-004	1.025e-004
Cl	2.566e-004	2.566e-004
Cu	4.257e-008	4.257e-008
F	4.588e-006	4.588e-006
K	2.101e-005	2.101e-005
Mg	3.153e-004	3.153e-004
Mn	2.043e-007	2.043e-007
Na	5.927e-004	5.927e-004
S(6)	1.773e-005	1.773e-005
Si	3.002e-002	3.002e-002
Zn	1.763e-007	1.763e-007

----------------------------Description of solution----------------------------

pH	=	7.000
pe	=	4.000
Activity of water	=	0.999
Ionic strength	=	1.865e-003
Mass of water (kg)	=	1.000e+000
Total carbon (mol/kg)	=	1.405e-003
Total CO2 (mol/kg)	=	1.405e-003
Temperature (deg C)	=	11.300
Electrical balance (eq)	=	1.754e-005
Percent error, 100*(Cat-|An|)/(Cat+|An|)	=	0.61
Iterations	=	9
Total H	=	1.111336e+002
Total O	=	5.563027e+001

----------------------------Distribution of species----------------------------

Species		Molality	Activity	Log Molality	Log Activity	Log Gamma
H+		1.045e-007	1.000e-007	-6.981	-7.000	-0.019
OH-		3.438e-008	3.279e-008	-7.464	-7.484	-0.021
H2O		5.551e+001	9.994e-001	1.744	-0.000	0.000
Ba	2.334e-008					
Ba+2		2.308e-008	1.917e-008	-7.637	-7.717	-0.080
BaSO4		1.348e-010	1.349e-010	-9.870	-9.870	0.000
BaHCO3+		1.318e-010	1.258e-010	-9.880	-9.900	-0.020
BaCO3		2.673e-012	2.674e-012	-11.573	-11.573	0.000
BaOH+		6.805e-015	6.493e-015	-14.167	-14.188	-0.020
C(4)	1.405e-003					
HCO3-		1.103e-003	1.053e-003	-2.957	-2.977	-0.020
CO2		2.975e-004	2.977e-004	-3.526	-3.526	0.000
MgHCO3+		3.215e-006	3.068e-006	-5.493	-5.513	-0.020
CaHCO3+		8.948e-007	8.545e-007	-6.048	-6.068	-0.020
CO3-2		4.273e-007	3.554e-007	-6.369	-6.449	-0.080
NaHCO3		3.347e-007	3.348e-007	-6.475	-6.475	0.000
MgCO3		7.120e-008	7.123e-008	-7.147	-7.147	0.000
CaCO3		4.129e-008	4.131e-008	-7.384	-7.384	0.000
ZnHCO3+		1.722e-008	1.643e-008	-7.764	-7.784	-0.020
MnHCO3+		1.510e-008	1.441e-008	-7.821	-7.841	-0.020
ZnCO3		8.784e-009	8.788e-009	-8.056	-8.056	0.000
MnCO3		4.333e-009	4.335e-009	-8.363	-8.363	0.000
NaCO3-		1.901e-009	1.813e-009	-8.721	-8.741	-0.020
BaHCO3+		1.318e-010	1.258e-010	-9.880	-9.900	-0.020
Zn(CO3)2-2		8.055e-011	6.677e-011	-10.094	-10.175	-0.081
BaCO3		2.673e-012	2.674e-012	-11.573	-11.573	0.000
Ca	1.025e-004					
Ca+2		1.013e-004	8.427e-005	-3.994	-4.074	-0.080
CaHCO3+		8.948e-007	8.545e-007	-6.048	-6.068	-0.020
CaSO4		2.063e-007	2.064e-007	-6.686	-6.685	0.000
CaCO3		4.129e-008	4.131e-008	-7.384	-7.384	0.000
CaF+		2.375e-009	2.266e-009	-8.624	-8.645	-0.020
CaOH+		1.465e-010	1.398e-010	-9.834	-9.855	-0.020
CaHSO4+		1.094e-013	1.044e-013	-12.961	-12.981	-0.020
Cl	2.566e-004					
Cl-		2.566e-004	2.448e-004	-3.591	-3.611	-0.020
MnCl+		1.605e-010	1.531e-010	-9.795	-9.815	-0.020
ZnCl+		4.542e-011	4.334e-011	-10.343	-10.363	-0.020
MnCl2		1.635e-014	1.636e-014	-13.786	-13.786	0.000
ZnCl2		1.048e-014	1.049e-014	-13.980	-13.979	0.000
ZnCl3-		2.769e-018	2.642e-018	-17.558	-17.578	-0.020
MnCl3-		1.156e-018	1.103e-018	-17.937	-17.957	-0.020
ZnCl4-2		3.489e-022	2.893e-022	-21.457	-21.539	-0.081
Cu(1)	5.954e-010					
Cu+		5.954e-010	5.676e-010	-9.225	-9.246	-0.021
Cu(2)	4.197e-008					
Cu(OH)2		2.580e-008	2.581e-008	-7.588	-7.588	0.000
Cu+2		1.485e-008	1.237e-008	-7.828	-7.908	-0.079
CuOH+		1.295e-009	1.236e-009	-8.888	-8.908	-0.020
CuSO4		3.208e-011	3.209e-011	-10.494	-10.494	0.000
Cu(OH)3-		1.629e-014	1.554e-014	-13.788	-13.808	-0.020
Cu(OH)4-2		3.739e-020	3.100e-020	-19.427	-19.509	-0.081
F	4.588e-006					
F-		4.524e-006	4.315e-006	-5.344	-5.365	-0.021
MgF+		5.971e-008	5.698e-008	-7.224	-7.244	-0.020
CaF+		2.375e-009	2.266e-009	-8.624	-8.645	-0.020
NaF		1.403e-009	1.404e-009	-8.853	-8.853	0.000
HF		5.092e-010	5.094e-010	-9.293	-9.293	0.000
MnF+		4.804e-012	4.584e-012	-11.318	-11.339	-0.020
HF2-		7.758e-015	7.403e-015	-14.110	-14.131	-0.020
SiF6-2		1.329e-031	1.102e-031	-30.876	-30.958	-0.081

H(0)	1.633e-025					
H2		8.164e-026	8.168e-026	-25.088	-25.088	0.000
K	2.101e-005					
K+		2.101e-005	2.004e-005	-4.678	-4.698	-0.020
KSO4-		1.612e-009	1.538e-009	-8.793	-8.813	-0.020
KOH		6.942e-013	6.945e-013	-12.159	-12.158	0.000
Mg	3.153e-004					
Mg+2		3.113e-004	2.592e-004	-3.507	-3.586	-0.080
MgHCO3+		3.215e-006	3.068e-006	-5.493	-5.513	-0.020
MgSO4		5.889e-007	5.892e-007	-6.230	-6.230	0.000
MgCO3		7.120e-008	7.123e-008	-7.147	-7.147	0.000
MgF+		5.971e-008	5.698e-008	-7.224	-7.244	-0.020
MgOH+		2.695e-009	2.572e-009	-8.569	-8.590	-0.020
Mn(2)	2.043e-007					
Mn+2		1.843e-007	1.535e-007	-6.734	-6.814	-0.079
MnHCO3+		1.510e-008	1.441e-008	-7.821	-7.841	-0.020
MnCO3		4.333e-009	4.335e-009	-8.363	-8.363	0.000
MnSO4		2.913e-010	2.914e-010	-9.536	-9.536	0.000
MnCl+		1.605e-010	1.531e-010	-9.795	-9.815	-0.020
MnOH+		1.282e-011	1.224e-011	-10.892	-10.912	-0.020
MnF+		4.804e-012	4.584e-012	-11.318	-11.339	-0.020
MnCl2		1.635e-014	1.636e-014	-13.786	-13.786	0.000
MnCl3-		1.156e-018	1.103e-018	-17.937	-17.957	-0.020
Mn(3)	8.885e-030					
Mn+3		8.885e-030	5.827e-030	-29.051	-29.235	-0.183
Na	5.927e-004					
Na+		5.923e-004	5.653e-004	-3.227	-3.248	-0.020
NaHCO3		3.347e-007	3.348e-007	-6.475	-6.475	0.000
NaSO4-		3.805e-008	3.631e-008	-7.420	-7.440	-0.020
NaCO3-		1.901e-009	1.813e-009	-8.721	-8.741	-0.020
NaF		1.403e-009	1.404e-009	-8.853	-8.853	0.000
NaOH		3.732e-011	3.733e-011	-10.428	-10.428	0.000
O(0)	0.000e+000					
O2		0.000e+000	0.000e+000	-46.839	-46.839	0.000
S(6)	1.773e-005					
SO4-2		1.689e-005	1.404e-005	-4.772	-4.853	-0.081
MgSO4		5.889e-007	5.892e-007	-6.230	-6.230	0.000
CaSO4		2.063e-007	2.064e-007	-6.686	-6.685	0.000
NaSO4-		3.805e-008	3.631e-008	-7.420	-7.440	-0.020
KSO4-		1.612e-009	1.538e-009	-8.793	-8.813	-0.020
ZnSO4		3.649e-010	3.650e-010	-9.438	-9.438	0.000
MnSO4		2.913e-010	2.914e-010	-9.536	-9.536	0.000
BaSO4		1.348e-010	1.349e-010	-9.870	-9.870	0.000
HSO4-		1.080e-010	1.030e-010	-9.967	-9.987	-0.020
CuSO4		3.208e-011	3.209e-011	-10.494	-10.494	0.000
CaHSO4+		1.094e-013	1.044e-013	-12.961	-12.981	-0.020
Zn(SO4)2-2		5.611e-014	4.651e-014	-13.251	-13.332	-0.081
Si	3.002e-002					
H4SiO4		2.999e-002	3.000e-002	-1.523	-1.523	0.000
H3SiO4-		2.750e-005	2.624e-005	-4.561	-4.581	-0.020
H2SiO4-2		8.176e-012	6.778e-012	-11.087	-11.169	-0.081
SiF6-2		1.329e-031	1.102e-031	-30.876	-30.958	-0.081
Zn	1.763e-007					
Zn+2		1.491e-007	1.239e-007	-6.826	-6.907	-0.080
ZnHCO3+		1.722e-008	1.643e-008	-7.764	-7.784	-0.020
ZnCO3		8.784e-009	8.788e-009	-8.056	-8.056	0.000
ZnOH+		4.788e-010	4.569e-010	-9.320	-9.340	-0.020
ZnSO4		3.649e-010	3.650e-010	-9.438	-9.438	0.000
Zn(OH)2		1.558e-010	1.558e-010	-9.808	-9.807	0.000
Zn(CO3)2-2		8.055e-011	6.677e-011	-10.094	-10.175	-0.081
ZnCl+		4.542e-011	4.334e-011	-10.343	-10.363	-0.020
Zn(SO4)2-2		5.611e-014	4.651e-014	-13.251	-13.332	-0.081
ZnCl2		1.048e-014	1.049e-014	-13.980	-13.979	0.000
Zn(OH)3-		5.161e-015	4.925e-015	-14.287	-14.308	-0.020
ZnCl3-		2.769e-018	2.642e-018	-17.558	-17.578	-0.020
Zn(OH)4-2		9.410e-021	7.801e-021	-20.026	-20.108	-0.081
ZnCl4-2		3.489e-022	2.893e-022	-21.457	-21.539	-0.081

------------------------------Saturation indices-------------------------------

Phase	SI	log IAP	log KT	
Anhydrite	-4.59	-8.93	-4.33	CaSO4
Aragonite	-2.26	-10.52	-8.26	CaCO3
Barite	-2.35	-12.57	-10.22	BaSO4
Calcite	-2.11	-10.52	-8.42	CaCO3
Chalcedony	2.20	-1.52	-3.72	SiO2
Chrysotile	-5.78	28.20	33.98	Mg3Si2O5(OH)4
CO2(g)	-2.24	-3.53	-1.29	CO2
Dolomite	-3.80	-20.56	-16.76	CaMg(CO3)2
Fluorite	-4.03	-14.80	-10.78	CaF2
Gypsum	-4.34	-8.93	-4.59	CaSO4:2H2O
H2(g)	-22.00	-25.09	-3.09	H2
H2O(g)	-1.88	-0.00	1.88	H2O
Halite	-8.41	-6.86	1.55	NaCl
Hausmannite	-21.03	43.56	64.58	Mn3O4
Manganite	-7.15	18.19	25.34	MnOOH
O2(g)	-43.94	-46.84	-2.89	O2
Pyrochroite	-8.01	7.19	15.20	Mn(OH)2
Pyrolusite	-14.49	29.19	43.68	MnO2
Quartz	2.67	-1.52	-4.19	SiO2
Rhodochrosite	-2.18	-13.26	-11.08	MnCO3
Sepiolite	0.12	16.26	16.14	Mg2Si3O7.5OH:3H2O
Sepiolite(d)	-2.40	16.26	18.66	Mg2Si3O7.5OH:3H2O
SiO2(a)	1.31	-1.52	-2.83	SiO2
Smithsonite	-3.51	-13.36	-9.85	ZnCO3
Talc	2.12	25.15	23.04	Mg3Si4O10(OH)2
Willemite	-3.84	12.66	16.51	Zn2SiO4
Witherite	-5.54	-14.17	-8.62	BaCO3
Zn(OH)2(e)	-4.41	7.09	11.50	Zn(OH)2

Initial solution 28.   4LValentina-1

-----------------------------Solution composition------------------------------

Elements	Molality	Moles
Alkalinity	3.525e-003	3.525e-003
Ba	1.610e-008	1.610e-008
Ca	3.160e-004	3.160e-004
Cl	1.121e-003	1.121e-003
Cu	3.638e-008	3.638e-008
F	6.613e-006	6.613e-006
K	4.858e-005	4.858e-005
Mg	7.607e-004	7.607e-004
Mn	2.342e-007	2.342e-007
Na	1.902e-003	1.902e-003
S(6)	6.612e-005	6.612e-005
Si	7.862e-002	7.862e-002
Zn	6.765e-008	6.765e-008

----------------------------Description of solution----------------------------

pH	=	7.000
pe	=	4.000
Activity of water	=	0.999
Ionic strength	=	5.496e-003
Mass of water (kg)	=	1.000e+000
Total carbon (mol/kg)	=	4.188e-003
Total CO2 (mol/kg)	=	4.188e-003
Temperature (deg C)	=	19.100
Electrical balance (eq)	=	-6.804e-004
Percent error, 100*(Cat-|An|)/(Cat+|An|)	=	-7.74
Iterations	=	10

Total H = 1.113302e+002
Total O = 5.583276e+001

----------------------------Distribution of species----------------------------

Species		Molality	Activity	Log Molality	Log Activity	Log Gamma
H+		1.073e-007	1.000e-007	-6.969	-7.000	-0.031
OH-		6.828e-008	6.307e-008	-7.166	-7.200	-0.034
H2O		5.551e+001	9.985e-001	1.744	-0.001	0.000
Ba	1.610e-008					
Ba+2		1.554e-008	1.143e-008	-7.809	-7.942	-0.133
BaHCO3+		3.086e-010	2.854e-010	-9.511	-9.545	-0.034
BaSO4		2.487e-010	2.490e-010	-9.604	-9.604	0.001
BaCO3		6.777e-012	6.786e-012	-11.169	-11.168	0.001
BaOH+		4.181e-015	3.866e-015	-14.379	-14.413	-0.034
C(4)	4.188e-003					
HCO3-		3.385e-003	3.137e-003	-2.470	-2.503	-0.033
CO2		7.667e-004	7.677e-004	-3.115	-3.115	0.001
MgHCO3+		2.108e-005	1.949e-005	-4.676	-4.710	-0.034
CaHCO3+		8.764e-006	8.122e-006	-5.057	-5.090	-0.033
NaHCO3		3.095e-006	3.099e-006	-5.509	-5.509	0.001
CO3-2		1.754e-006	1.294e-006	-5.756	-5.888	-0.132
MgCO3		6.117e-007	6.125e-007	-6.213	-6.213	0.001
CaCO3		4.392e-007	4.397e-007	-6.357	-6.357	0.001
MnHCO3+		4.006e-008	3.705e-008	-7.397	-7.431	-0.034
NaCO3-		3.378e-008	3.124e-008	-7.471	-7.505	-0.034
ZnHCO3+		1.392e-008	1.288e-008	-7.856	-7.890	-0.034
MnCO3		1.360e-008	1.362e-008	-7.866	-7.866	0.001
ZnCO3		8.407e-009	8.417e-009	-8.075	-8.075	0.001
Zn(CO3)2-2		3.184e-010	2.329e-010	-9.497	-9.633	-0.136
BaHCO3+		3.086e-010	2.854e-010	-9.511	-9.545	-0.034
BaCO3		6.777e-012	6.786e-012	-11.169	-11.168	0.001
Ca	3.160e-004					
Ca+2		3.049e-004	2.247e-004	-3.516	-3.648	-0.132
CaHCO3+		8.764e-006	8.122e-006	-5.057	-5.090	-0.033
CaSO4		1.841e-006	1.843e-006	-5.735	-5.734	0.001
CaCO3		4.392e-007	4.397e-007	-6.357	-6.357	0.001
CaF+		1.086e-008	1.004e-008	-7.964	-7.998	-0.034
CaOH+		4.027e-010	3.724e-010	-9.395	-9.429	-0.034
CaHSO4+		1.089e-012	1.007e-012	-11.963	-11.997	-0.034
Cl	1.121e-003					
Cl-		1.121e-003	1.035e-003	-2.951	-2.985	-0.034
MnCl+		6.043e-010	5.588e-010	-9.219	-9.253	-0.034
ZnCl+		7.534e-011	6.967e-011	-10.123	-10.157	-0.034
MnCl2		2.522e-013	2.525e-013	-12.598	-12.598	0.001
ZnCl2		7.362e-014	7.371e-014	-13.133	-13.132	0.001
ZnCl3-		8.931e-017	8.259e-017	-16.049	-16.083	-0.034
MnCl3-		7.786e-017	7.200e-017	-16.109	-16.143	-0.034
ZnCl4-2		5.587e-020	4.085e-020	-19.253	-19.389	-0.136
Cu(1)	5.435e-010					
Cu+		5.435e-010	5.011e-010	-9.265	-9.300	-0.035
Cu(2)	3.583e-008					
Cu(OH)2		2.101e-008	2.104e-008	-7.677	-7.677	0.001
Cu+2		1.364e-008	1.010e-008	-7.865	-7.996	-0.131
CuOH+		1.091e-009	1.009e-009	-8.962	-8.996	-0.034
CuSO4		8.590e-011	8.601e-011	-10.066	-10.065	0.001
Cu(OH)3-		1.369e-014	1.266e-014	-13.864	-13.898	-0.034
Cu(OH)4-2		3.449e-020	2.522e-020	-19.462	-19.598	-0.136
F	6.613e-006					
F-		6.390e-006	5.902e-006	-5.195	-5.229	-0.034
MgF+		2.054e-007	1.899e-007	-6.687	-6.721	-0.034
CaF+		1.086e-008	1.004e-008	-7.964	-7.998	-0.034
NaF		5.959e-009	5.966e-009	-8.225	-8.224	0.001
HF		7.960e-010	7.970e-010	-9.099	-9.099	0.001
MnF+		5.851e-012	5.410e-012	-11.233	-11.267	-0.034

HF2-		1.857e-014	1.717e-014	-13.731	-13.765	-0.034
SiF6-2		1.204e-030	8.808e-031	-29.919	-30.055	-0.136
H(0)	1.501e-025					
H2		7.507e-026	7.517e-026	-25.125	-25.124	0.001
K	4.858e-005					
K+		4.857e-005	4.487e-005	-4.314	-4.348	-0.034
KSO4-		1.334e-008	1.234e-008	-7.875	-7.909	-0.034
KOH		1.552e-012	1.554e-012	-11.809	-11.809	0.001
Mg	7.607e-004					
Mg+2		7.340e-004	5.431e-004	-3.134	-3.265	-0.131
MgHCO3+		2.108e-005	1.949e-005	-4.676	-4.710	-0.034
MgSO4		4.734e-006	4.740e-006	-5.325	-5.324	0.001
MgCO3		6.117e-007	6.125e-007	-6.213	-6.213	0.001
MgF+		2.054e-007	1.899e-007	-6.687	-6.721	-0.034
MgOH+		1.236e-008	1.143e-008	-7.908	-7.942	-0.034
Mn(2)	2.342e-007					
Mn+2		1.790e-007	1.325e-007	-6.747	-6.878	-0.131
MnHCO3+		4.006e-008	3.705e-008	-7.397	-7.431	-0.034
MnCO3		1.360e-008	1.362e-008	-7.866	-7.866	0.001
MnSO4		9.121e-010	9.132e-010	-9.040	-9.039	0.001
MnCl+		6.043e-010	5.588e-010	-9.219	-9.253	-0.034
MnOH+		2.251e-011	2.082e-011	-10.648	-10.682	-0.034
MnF+		5.851e-012	5.410e-012	-11.233	-11.267	-0.034
MnCl2		2.522e-013	2.525e-013	-12.598	-12.598	0.001
MnCl3-		7.786e-017	7.200e-017	-16.109	-16.143	-0.034
Mn(3)	3.437e-029					
Mn+3		3.437e-029	1.700e-029	-28.464	-28.770	-0.306
Na	1.902e-003					
Na+		1.898e-003	1.757e-003	-2.722	-2.755	-0.034
NaHCO3		3.095e-006	3.099e-006	-5.509	-5.509	0.001
NaSO4-		3.984e-007	3.684e-007	-6.400	-6.434	-0.034
NaCO3-		3.378e-008	3.124e-008	-7.471	-7.505	-0.034
NaF		5.959e-009	5.966e-009	-8.225	-8.224	0.001
NaOH		1.157e-010	1.159e-010	-9.937	-9.936	0.001
O(0)	0.000e+000					
O2		0.000e+000	0.000e+000	-44.076	-44.076	0.001
S(6)	6.612e-005					
SO4-2		5.913e-005	4.348e-005	-4.228	-4.362	-0.134
MgSO4		4.734e-006	4.740e-006	-5.325	-5.324	0.001
CaSO4		1.841e-006	1.843e-006	-5.735	-5.734	0.001
NaSO4-		3.984e-007	3.684e-007	-6.400	-6.434	-0.034
KSO4-		1.334e-008	1.234e-008	-7.875	-7.909	-0.034
MnSO4		9.121e-010	9.132e-010	-9.040	-9.039	0.001
HSO4-		4.031e-010	3.728e-010	-9.395	-9.429	-0.034
ZnSO4		3.168e-010	3.172e-010	-9.499	-9.499	0.001
BaSO4		2.487e-010	2.490e-010	-9.604	-9.604	0.001
CuSO4		8.590e-011	8.601e-011	-10.066	-10.065	0.001
CaHSO4+		1.089e-012	1.007e-012	-11.963	-11.997	-0.034
Zn(SO4)2-2		1.606e-013	1.174e-013	-12.794	-12.930	-0.136
Si	7.862e-002					
H4SiO4		7.852e-002	7.862e-002	-1.105	-1.104	0.001
H3SiO4-		1.013e-004	9.365e-005	-3.994	-4.028	-0.034
H2SiO4-2		5.852e-011	4.279e-011	-10.233	-10.369	-0.136
SiF6-2		1.204e-030	8.808e-031	-29.919	-30.055	-0.136
Zn	6.765e-008					
Zn+2		4.432e-008	3.260e-008	-7.353	-7.487	-0.133
ZnHCO3+		1.392e-008	1.288e-008	-7.856	-7.890	-0.034
ZnCO3		8.407e-009	8.417e-009	-8.075	-8.075	0.001
Zn(CO3)2-2		3.184e-010	2.329e-010	-9.497	-9.633	-0.136
ZnSO4		3.168e-010	3.172e-010	-9.499	-9.499	0.001
ZnOH+		2.445e-010	2.261e-010	-9.612	-9.646	-0.034
ZnCl+		7.534e-011	6.967e-011	-10.123	-10.157	-0.034
Zn(OH)2		4.087e-011	4.092e-011	-10.389	-10.388	0.001
Zn(SO4)2-2		1.606e-013	1.174e-013	-12.794	-12.930	-0.136
ZnCl2		7.362e-014	7.371e-014	-13.133	-13.132	0.001
Zn(OH)3-		1.397e-015	1.292e-015	-14.855	-14.889	-0.034
ZnCl3-		8.931e-017	8.259e-017	-16.049	-16.083	-0.034

ZnCl4-2	5.587e-020	4.085e-020	-19.253	-19.389	-0.136
Zn(OH)4-2	2.796e-021	2.045e-021	-20.553	-20.689	-0.136

------------------------------Saturation indices-------------------------------

Phase	SI	log IAP	log KT	
Anhydrite	-3.67	-8.01	-4.34	CaSO4
Aragonite	-1.24	-9.54	-8.30	CaCO3
Barite	-2.24	-12.30	-10.07	BaSO4
Calcite	-1.09	-9.54	-8.45	CaCO3
Chalcedony	2.52	-1.10	-3.62	SiO2
Chrysotile	-2.95	29.99	32.95	Mg3Si2O5(OH)4
CO2(g)	-1.72	-3.11	-1.40	CO2
Dolomite	-1.74	-18.69	-16.95	CaMg(CO3)2
Fluorite	-3.43	-14.11	-10.67	CaF2
Gypsum	-3.43	-8.01	-4.58	CaSO4:2H2O
H2(g)	-22.00	-25.12	-3.12	H2
H2O(g)	-1.67	-0.00	1.67	H2O
Halite	-7.31	-5.74	1.57	NaCl
Hausmannite	-19.16	43.36	62.52	Mn3O4
Manganite	-7.22	18.12	25.34	MnOOH
O2(g)	-41.14	-44.08	-2.93	O2
Pyrochroite	-8.08	7.12	15.20	Mn(OH)2
Pyrolusite	-13.22	29.12	42.34	MnO2
Quartz	2.97	-1.10	-4.07	SiO2
Rhodochrosite	-1.66	-12.77	-11.11	MnCO3
Sepiolite	2.24	18.16	15.92	Mg2Si3O7.5OH:3H2O
Sepiolite(d)	-0.50	18.16	18.66	Mg2Si3O7.5OH:3H2O
SiO2(a)	1.66	-1.10	-2.76	SiO2
Smithsonite	-3.44	-13.37	-9.94	ZnCO3
Talc	5.70	27.79	22.08	Mg3Si4O10(OH)2
Willemite	-3.90	11.92	15.82	Zn2SiO4
Witherite	-5.25	-13.83	-8.58	BaCO3
Zn(OH)2(e)	-4.99	6.51	11.50	Zn(OH)2

Initial solution 29. 4LEsther-1

-----------------------------Solution composition------------------------------

Elements	Molality	Moles
Alkalinity	1.495e-003	1.495e-003
Ba	1.169e-008	1.169e-008
Ca	1.928e-004	1.928e-004
Cl	1.174e-003	1.174e-003
Cu	5.528e-008	5.528e-008
F	1.804e-005	1.804e-005
K	2.053e-005	2.053e-005
Mg	2.770e-004	2.770e-004
Mn	1.845e-007	1.845e-007
Na	1.183e-003	1.183e-003
S(6)	4.774e-005	4.774e-005
Si	5.679e-002	5.679e-002
Zn	1.320e-007	1.320e-007

----------------------------Description of solution----------------------------

pH	=	7.000
pe	=	4.000
Activity of water	=	0.999
Ionic strength	=	2.958e-003
Mass of water (kg)	=	1.000e+000
Total carbon (mol/kg)	=	1.774e-003
Total CO2 (mol/kg)	=	1.774e-003
Temperature (deg C)	=	16.300
Electrical balance (eq)	=	-6.387e-004

Percent error, 100*(Cat-|An|)/(Cat+|An|) = -13.03
Iterations = 9
Total H = 1.112410e+002
Total O = 5.573855e+001

----------------------------Distribution of species----------------------------

Species		Molality	Activity	Log Molality	Log Activity	Log Gamma
H+		1.056e-007	1.000e-007	-6.976	-7.000	-0.024
OH-		5.326e-008	5.019e-008	-7.274	-7.299	-0.026
H2O		5.551e+001	9.990e-001	1.744	-0.000	0.000
Ba	1.169e-008					
Ba+2		1.143e-008	9.075e-009	-7.942	-8.042	-0.100
BaSO4		1.627e-010	1.628e-010	-9.789	-9.788	0.000
BaHCO3+		9.420e-011	8.883e-011	-10.026	-10.051	-0.025
BaCO3		2.036e-012	2.038e-012	-11.691	-11.691	0.000
BaOH+		3.257e-015	3.072e-015	-14.487	-14.513	-0.025
C(4)	1.774e-003					
HCO3-		1.422e-003	1.343e-003	-2.847	-2.872	-0.025
CO2		3.441e-004	3.444e-004	-3.463	-3.463	0.000
MgHCO3+		3.497e-006	3.298e-006	-5.456	-5.482	-0.025
CaHCO3+		2.326e-006	2.197e-006	-5.633	-5.658	-0.025
NaHCO3		8.416e-007	8.421e-007	-6.075	-6.075	0.000
CO3-2		6.511e-007	5.176e-007	-6.186	-6.286	-0.100
CaCO3		1.131e-007	1.132e-007	-6.947	-6.946	0.000
MgCO3		9.344e-008	9.351e-008	-7.029	-7.029	0.000
MnHCO3+		1.633e-008	1.540e-008	-7.787	-7.812	-0.025
ZnHCO3+		1.516e-008	1.430e-008	-7.819	-7.845	-0.025
ZnCO3		8.730e-009	8.736e-009	-8.059	-8.059	0.000
NaCO3-		7.252e-009	6.839e-009	-8.140	-8.165	-0.025
MnCO3		5.288e-009	5.291e-009	-8.277	-8.276	0.000
Zn(CO3)2-2		1.222e-010	9.667e-011	-9.913	-10.015	-0.102
BaHCO3+		9.420e-011	8.883e-011	-10.026	-10.051	-0.025
BaCO3		2.036e-012	2.038e-012	-11.691	-11.691	0.000
Ca	1.928e-004					
Ca+2		1.894e-004	1.505e-004	-3.723	-3.823	-0.100
CaHCO3+		2.326e-006	2.197e-006	-5.633	-5.658	-0.025
CaSO4		9.877e-007	9.883e-007	-6.005	-6.005	0.000
CaCO3		1.131e-007	1.132e-007	-6.947	-6.946	0.000
CaF+		1.891e-008	1.783e-008	-7.723	-7.749	-0.025
CaOH+		2.645e-010	2.495e-010	-9.578	-9.603	-0.025
CaHSO4+		5.560e-013	5.244e-013	-12.255	-12.280	-0.025
Cl	1.174e-003					
Cl-		1.174e-003	1.107e-003	-2.930	-2.956	-0.026
MnCl+		6.152e-010	5.802e-010	-9.211	-9.236	-0.025
ZnCl+		1.799e-010	1.697e-010	-9.745	-9.770	-0.025
MnCl2		2.800e-013	2.802e-013	-12.553	-12.552	0.000
ZnCl2		1.895e-013	1.897e-013	-12.722	-12.722	0.000
ZnCl3-		2.366e-016	2.232e-016	-15.626	-15.651	-0.025
MnCl3-		9.056e-017	8.541e-017	-16.043	-16.069	-0.025
ZnCl4-2		1.457e-019	1.153e-019	-18.836	-18.938	-0.102
Cu(1)	8.090e-010					
Cu+		8.090e-010	7.617e-010	-9.092	-9.118	-0.026
Cu(2)	5.447e-008					
Cu(OH)2		3.288e-008	3.290e-008	-7.483	-7.483	0.000
Cu+2		1.981e-008	1.578e-008	-7.703	-7.802	-0.099
CuOH+		1.672e-009	1.576e-009	-8.777	-8.802	-0.025
CuSO4		1.083e-010	1.084e-010	-9.965	-9.965	0.000
Cu(OH)3-		2.100e-014	1.980e-014	-13.678	-13.703	-0.025
Cu(OH)4-2		4.990e-020	3.947e-020	-19.302	-19.404	-0.102
F	1.804e-005					
F-		1.779e-005	1.677e-005	-4.750	-4.775	-0.026
MgF+		2.161e-007	2.038e-007	-6.665	-6.691	-0.025
CaF+		1.891e-008	1.783e-008	-7.723	-7.749	-0.025
NaF		1.075e-008	1.076e-008	-7.968	-7.968	0.000

	HF		2.155e-009	2.157e-009	-8.667	-8.666	0.000
	MnF+		1.583e-011	1.493e-011	-10.800	-10.826	-0.025
	HF2-		1.362e-013	1.285e-013	-12.866	-12.891	-0.025
	SiF6-2		5.534e-028	4.377e-028	-27.257	-27.359	-0.102
H(0)		1.547e-025					
	H2		7.735e-026	7.740e-026	-25.112	-25.111	0.000
K		2.053e-005					
	K+		2.053e-005	1.935e-005	-4.688	-4.713	-0.026
	KSO4-		4.414e-009	4.162e-009	-8.355	-8.381	-0.025
	KOH		6.698e-013	6.702e-013	-12.174	-12.174	0.000
Mg		2.770e-004					
	Mg+2		2.717e-004	2.164e-004	-3.566	-3.665	-0.099
	MgHCO3+		3.497e-006	3.298e-006	-5.456	-5.482	-0.025
	MgSO4		1.440e-006	1.441e-006	-5.841	-5.841	0.000
	MgF+		2.161e-007	2.038e-007	-6.665	-6.691	-0.025
	MgCO3		9.344e-008	9.351e-008	-7.029	-7.029	0.000
	MgOH+		3.705e-009	3.494e-009	-8.431	-8.457	-0.025
Mn(2)		1.845e-007					
	Mn+2		1.615e-007	1.287e-007	-6.792	-6.890	-0.099
	MnHCO3+		1.633e-008	1.540e-008	-7.787	-7.812	-0.025
	MnCO3		5.288e-009	5.291e-009	-8.277	-8.276	0.000
	MnSO4		6.900e-010	6.905e-010	-9.161	-9.161	0.000
	MnCl+		6.152e-010	5.802e-010	-9.211	-9.236	-0.025
	MnOH+		1.688e-011	1.592e-011	-10.773	-10.798	-0.025
	MnF+		1.583e-011	1.493e-011	-10.800	-10.826	-0.025
	MnCl2		2.800e-013	2.802e-013	-12.553	-12.552	0.000
	MnCl3-		9.056e-017	8.541e-017	-16.043	-16.069	-0.025
Mn(3)		1.821e-029					
	Mn+3		1.821e-029	1.074e-029	-28.740	-28.969	-0.229
Na		1.183e-003					
	Na+		1.182e-003	1.115e-003	-2.927	-2.953	-0.025
	NaHCO3		8.416e-007	8.421e-007	-6.075	-6.075	0.000
	NaSO4-		2.004e-007	1.890e-007	-6.698	-6.724	-0.025
	NaF		1.075e-008	1.076e-008	-7.968	-7.968	0.000
	NaCO3-		7.252e-009	6.839e-009	-8.140	-8.165	-0.025
	NaOH		7.355e-011	7.360e-011	-10.133	-10.133	0.000
O(0)		0.000e+000					
	O2		0.000e+000	0.000e+000	-45.051	-45.051	0.000
S(6)		4.774e-005					
	SO4-2		4.511e-005	3.579e-005	-4.346	-4.446	-0.100
	MgSO4		1.440e-006	1.441e-006	-5.841	-5.841	0.000
	CaSO4		9.877e-007	9.883e-007	-6.005	-6.005	0.000
	NaSO4-		2.004e-007	1.890e-007	-6.698	-6.724	-0.025
	KSO4-		4.414e-009	4.162e-009	-8.355	-8.381	-0.025
	MnSO4		6.900e-010	6.905e-010	-9.161	-9.161	0.000
	ZnSO4		6.620e-010	6.625e-010	-9.179	-9.179	0.000
	HSO4-		3.074e-010	2.899e-010	-9.512	-9.538	-0.025
	BaSO4		1.627e-010	1.628e-010	-9.789	-9.788	0.000
	CuSO4		1.083e-010	1.084e-010	-9.965	-9.965	0.000
	CaHSO4+		5.560e-013	5.244e-013	-12.255	-12.280	-0.025
	Zn(SO4)2-2		2.611e-013	2.065e-013	-12.583	-12.685	-0.102
Si		5.679e-002					
	H4SiO4		5.673e-002	5.676e-002	-1.246	-1.246	0.000
	H3SiO4-		6.442e-005	6.075e-005	-4.191	-4.216	-0.025
	H2SiO4-2		2.876e-011	2.275e-011	-10.541	-10.643	-0.102
	SiF6-2		5.534e-028	4.377e-028	-27.257	-27.359	-0.102
Zn		1.320e-007					
	Zn+2		1.066e-007	8.459e-008	-6.972	-7.073	-0.100
	ZnHCO3+		1.516e-008	1.430e-008	-7.819	-7.845	-0.025
	ZnCO3		8.730e-009	8.736e-009	-8.059	-8.059	0.000
	ZnSO4		6.620e-010	6.625e-010	-9.179	-9.179	0.000
	ZnOH+		4.978e-010	4.695e-010	-9.303	-9.328	-0.025
	ZnCl+		1.799e-010	1.697e-010	-9.745	-9.770	-0.025
	Zn(CO3)2-2		1.222e-010	9.667e-011	-9.913	-10.015	-0.102
	Zn(OH)2		1.062e-010	1.063e-010	-9.974	-9.974	0.000
	Zn(SO4)2-2		2.611e-013	2.065e-013	-12.583	-12.685	-0.102
	ZnCl2		1.895e-013	1.897e-013	-12.722	-12.722	0.000

Zn(OH)3-	3.560e-015	3.357e-015	-14.449	-14.474	-0.025
ZnCl3-	2.366e-016	2.232e-016	-15.626	-15.651	-0.025
ZnCl4-2	1.457e-019	1.153e-019	-18.836	-18.938	-0.102
Zn(OH)4-2	6.719e-021	5.315e-021	-20.173	-20.275	-0.102

------------------------------Saturation indices-------------------------------

Phase	SI	log IAP	log KT	
Anhydrite	-3.93	-8.27	-4.34	CaSO4
Aragonite	-1.82	-10.11	-8.29	CaCO3
Barite	-2.37	-12.49	-10.12	BaSO4
Calcite	-1.67	-10.11	-8.44	CaCO3
Chalcedony	2.41	-1.25	-3.66	SiO2
Chrysotile	-4.80	28.51	33.31	Mg3Si2O5(OH)4
CO2(g)	-2.10	-3.46	-1.36	CO2
Dolomite	-3.18	-20.06	-16.88	CaMg(CO3)2
Fluorite	-2.67	-13.37	-10.71	CaF2
Gypsum	-3.69	-8.27	-4.58	CaSO4:2H2O
H2(g)	-22.00	-25.11	-3.11	H2
H2O(g)	-1.74	-0.00	1.74	H2O
Halite	-7.47	-5.91	1.56	NaCl
Hausmannite	-19.92	43.33	63.25	Mn3O4
Manganite	-7.23	18.11	25.34	MnOOH
O2(g)	-42.13	-45.05	-2.92	O2
Pyrochroite	-8.09	7.11	15.20	Mn(OH)2
Pyrolusite	-13.71	29.11	42.81	MnO2
Quartz	2.87	-1.25	-4.11	SiO2
Rhodochrosite	-2.08	-13.18	-11.10	MnCO3
Sepiolite	0.94	16.93	16.00	Mg2Si3O7.5OH:3H2O
Sepiolite(d)	-1.73	16.93	18.66	Mg2Si3O7.5OH:3H2O
SiO2(a)	1.54	-1.25	-2.79	SiO2
Smithsonite	-3.45	-13.36	-9.90	ZnCO3
Talc	3.60	26.02	22.42	Mg3Si4O10(OH)2
Willemite	-3.46	12.61	16.07	Zn2SiO4
Witherite	-5.74	-14.33	-8.59	BaCO3
Zn(OH)2(e)	-4.57	6.93	11.50	Zn(OH)2

Initial solution 30.   4LTatana-1

-----------------------------Solution composition------------------------------

Elements	Molality	Moles
Alkalinity	5.550e-003	5.550e-003
Ba	1.181e-008	1.181e-008
Ca	8.194e-004	8.194e-004
Cl	6.569e-003	6.569e-003
Cu	8.932e-008	8.932e-008
K	6.817e-005	6.817e-005
Mg	1.868e-003	1.868e-003
Mn	1.624e-007	1.624e-007
Na	6.393e-003	6.393e-003
S(6)	1.482e-004	1.482e-004
Si	2.126e-001	2.126e-001
Zn	1.395e-007	1.395e-007

----------------------------Description of solution----------------------------

pH = 7.000
pe = 4.000
Activity of water = 0.996
Ionic strength = 1.465e-002
Mass of water (kg) = 1.000e+000
Total carbon (mol/kg) = 6.478e-003
Total CO2 (mol/kg) = 6.478e-003
Temperature (deg C) = 16.000

```
                Electrical balance (eq)  = -5.788e-004
 Percent error, 100*(Cat-|An|)/(Cat+|An|)  =  -2.42
                             Iterations  =  11
                                Total H  = 1.118677e+002
                                Total O  = 5.637527e+001
```

----------------------------Distribution of species----------------------------

Species		Molality	Activity	Log Molality	Log Activity	Log Gamma
H+		1.109e-007	1.000e-007	-6.955	-7.000	-0.045
OH-		5.520e-008	4.881e-008	-7.258	-7.311	-0.053
H2O		5.551e+001	9.960e-001	1.744	-0.002	0.000
Ba	1.181e-008					
Ba+2		1.127e-008	7.061e-009	-7.948	-8.151	-0.203
BaSO4		2.714e-010	2.723e-010	-9.566	-9.565	0.001
BaHCO3+		2.650e-010	2.351e-010	-9.577	-9.629	-0.052
BaCO3		5.353e-012	5.371e-012	-11.271	-11.270	0.001
BaOH+		2.686e-015	2.383e-015	-14.571	-14.623	-0.052
C(4)	6.478e-003					
HCO3-		5.175e-003	4.611e-003	-2.286	-2.336	-0.050
CO2		1.188e-003	1.192e-003	-2.925	-2.924	0.001
MgHCO3+		6.683e-005	5.929e-005	-4.175	-4.227	-0.052
CaHCO3+		2.760e-005	2.459e-005	-4.559	-4.609	-0.050
NaHCO3		1.464e-005	1.469e-005	-4.834	-4.833	0.001
CO3-2		2.797e-006	1.764e-006	-5.553	-5.754	-0.200
MgCO3		1.656e-006	1.662e-006	-5.781	-5.779	0.001
CaCO3		1.257e-006	1.261e-006	-5.901	-5.899	0.001
NaCO3-		1.313e-007	1.165e-007	-6.882	-6.934	-0.052
ZnHCO3+		3.436e-008	3.048e-008	-7.464	-7.516	-0.052
MnHCO3+		3.399e-008	3.015e-008	-7.469	-7.521	-0.052
ZnCO3		1.842e-008	1.848e-008	-7.735	-7.733	0.001
MnCO3		1.024e-008	1.028e-008	-7.990	-7.988	0.001
Zn(CO3)2-2		1.125e-009	6.969e-010	-8.949	-9.157	-0.208
BaHCO3+		2.650e-010	2.351e-010	-9.577	-9.629	-0.052
BaCO3		5.353e-012	5.371e-012	-11.271	-11.270	0.001
Ca	8.194e-004					
Ca+2		7.836e-004	4.939e-004	-3.106	-3.306	-0.200
CaHCO3+		2.760e-005	2.459e-005	-4.559	-4.609	-0.050
CaSO4		6.928e-006	6.951e-006	-5.159	-5.158	0.001
CaCO3		1.257e-006	1.261e-006	-5.901	-5.899	0.001
CaOH+		9.201e-010	8.163e-010	-9.036	-9.088	-0.052
CaHSO4+		4.144e-012	3.677e-012	-11.383	-11.435	-0.052
Cl	6.569e-003					
Cl-		6.569e-003	5.812e-003	-2.183	-2.236	-0.053
MnCl+		1.958e-009	1.737e-009	-8.708	-8.760	-0.052
ZnCl+		6.149e-010	5.455e-010	-9.211	-9.263	-0.052
MnCl2		4.392e-012	4.407e-012	-11.357	-11.356	0.001
ZnCl2		3.188e-012	3.198e-012	-11.497	-11.495	0.001
ZnCl3-		2.224e-014	1.973e-014	-13.653	-13.705	-0.052
MnCl3-		7.952e-015	7.055e-015	-14.100	-14.152	-0.052
ZnCl4-2		8.619e-017	5.339e-017	-16.065	-16.273	-0.208
Cu(1)	1.278e-009					
Cu+		1.278e-009	1.125e-009	-8.893	-8.949	-0.055
Cu(2)	8.804e-008					
Cu(OH)2		4.831e-008	4.847e-008	-7.316	-7.314	0.001
Cu+2		3.676e-008	2.339e-008	-7.435	-7.631	-0.196
CuOH+		2.628e-009	2.329e-009	-8.580	-8.633	-0.052
CuSO4		3.434e-010	3.446e-010	-9.464	-9.463	0.001
Cu(OH)3-		3.279e-014	2.909e-014	-13.484	-13.536	-0.052
Cu(OH)4-2		9.333e-020	5.781e-020	-19.030	-19.238	-0.208
H(0)	1.548e-025					
H2		7.739e-026	7.765e-026	-25.111	-25.110	0.001
K	6.817e-005					
K+		6.814e-005	6.029e-005	-4.167	-4.220	-0.053
KSO4-		3.125e-008	2.772e-008	-7.505	-7.557	-0.052

```
   KOH                  2.075e-012  2.082e-012   -11.683   -11.681     0.001
Mg              1.868e-003
   Mg+2                 1.783e-003  1.134e-003    -2.749    -2.945    -0.197
   MgHCO3+              6.683e-005  5.929e-005    -4.175    -4.227    -0.052
   MgSO4                1.605e-005  1.610e-005    -4.795    -4.793     0.001
   MgCO3                1.656e-006  1.662e-006    -5.781    -5.779     0.001
   MgOH+                1.999e-008  1.774e-008    -7.699    -7.751    -0.052
Mn(2)           1.624e-007
   Mn+2                 1.153e-007  7.336e-008    -6.938    -7.135    -0.196
   MnHCO3+              3.399e-008  3.015e-008    -7.469    -7.521    -0.052
   MnCO3                1.024e-008  1.028e-008    -7.990    -7.988     0.001
   MnCl+                1.958e-009  1.737e-009    -8.708    -8.760    -0.052
   MnSO4                8.380e-010  8.408e-010    -9.077    -9.075     0.001
   MnOH+                9.936e-012  8.815e-012   -11.003   -11.055    -0.052
   MnCl2                4.392e-012  4.407e-012   -11.357   -11.356     0.001
   MnCl3-               7.952e-015  7.055e-015   -14.100   -14.152    -0.052
Mn(3)           1.717e-029
   Mn+3                 1.717e-029  5.846e-030   -28.765   -29.233    -0.468
Na              6.393e-003
   Na+                  6.376e-003  5.665e-003    -2.195    -2.247    -0.051
   NaHCO3               1.464e-005  1.469e-005    -4.834    -4.833     0.001
   NaSO4-               2.321e-006  2.059e-006    -5.634    -5.686    -0.052
   NaCO3-               1.313e-007  1.165e-007    -6.882    -6.934    -0.052
   NaOH                 3.715e-010  3.728e-010    -9.430    -9.429     0.001
O(0)            0.000e+000
   O2                   0.000e+000  0.000e+000   -45.160   -45.159     0.001
S(6)            1.482e-004
   SO4-2                1.229e-004  7.693e-005    -3.910    -4.114    -0.203
   MgSO4                1.605e-005  1.610e-005    -4.795    -4.793     0.001
   CaSO4                6.928e-006  6.951e-006    -5.159    -5.158     0.001
   NaSO4-               2.321e-006  2.059e-006    -5.634    -5.686    -0.052
   KSO4-                3.125e-008  2.772e-008    -7.505    -7.557    -0.052
   ZnSO4                8.786e-010  8.816e-010    -9.056    -9.055     0.001
   MnSO4                8.380e-010  8.408e-010    -9.077    -9.075     0.001
   HSO4-                6.980e-010  6.192e-010    -9.156    -9.208    -0.052
   CuSO4                3.434e-010  3.446e-010    -9.464    -9.463     0.001
   BaSO4                2.714e-010  2.723e-010    -9.566    -9.565     0.001
   CaHSO4+              4.144e-012  3.677e-012   -11.383   -11.435    -0.052
   Zn(SO4)2-2           9.558e-013  5.921e-013   -12.020   -12.228    -0.208
Si              2.126e-001
   H4SiO4               2.123e-001  2.130e-001    -0.673    -0.672     0.001
   H3SiO4-              2.540e-004  2.253e-004    -3.595    -3.647    -0.052
   H2SiO4-2             1.333e-010  8.258e-011    -9.875   -10.083    -0.208
Zn              1.395e-007
   Zn+2                 8.377e-008  5.250e-008    -7.077    -7.280    -0.203
   ZnHCO3+              3.436e-008  3.048e-008    -7.464    -7.516    -0.052
   ZnCO3                1.842e-008  1.848e-008    -7.735    -7.733     0.001
   Zn(CO3)2-2           1.125e-009  6.969e-010    -8.949    -9.157    -0.208
   ZnSO4                8.786e-010  8.816e-010    -9.056    -9.055     0.001
   ZnCl+                6.149e-010  5.455e-010    -9.211    -9.263    -0.052
   ZnOH+                3.197e-010  2.836e-010    -9.495    -9.547    -0.052
   Zn(OH)2              6.535e-011  6.557e-011   -10.185   -10.183     0.001
   ZnCl2                3.188e-012  3.198e-012   -11.497   -11.495     0.001
   Zn(SO4)2-2           9.558e-013  5.921e-013   -12.020   -12.228    -0.208
   ZnCl3-               2.224e-014  1.973e-014   -13.653   -13.705    -0.052
   Zn(OH)3-             2.328e-015  2.065e-015   -14.633   -14.685    -0.052
   ZnCl4-2              8.619e-017  5.339e-017   -16.065   -16.273    -0.208
   Zn(OH)4-2            5.263e-021  3.260e-021   -20.279   -20.487    -0.208

------------------------------Saturation indices-------------------------------

      Phase               SI  log IAP   log KT

      Anhydrite        -3.08    -7.42    -4.34  CaSO4
      Aragonite        -0.78    -9.06    -8.28  CaCO3
      Barite           -2.14   -12.27   -10.13  BaSO4
      Calcite          -0.63    -9.06    -8.43  CaCO3
```

Chalcedony	2.99	-0.67	-3.66	SiO2
Chrysotile	-1.53	31.82	33.35	Mg3Si2O5(OH)4
CO2(g)	-1.57	-2.92	-1.35	CO2
Dolomite	-0.88	-17.76	-16.87	CaMg(CO3)2
Gypsum	-2.84	-7.42	-4.58	CaSO4:2H2O
H2(g)	-22.00	-25.11	-3.11	H2
H2O(g)	-1.75	-0.00	1.75	H2O
Halite	-6.04	-4.48	1.56	NaCl
Hausmannite	-20.74	42.59	63.33	Mn3O4
Manganite	-7.48	17.86	25.34	MnOOH
O2(g)	-42.24	-45.16	-2.92	O2
Pyrochroite	-8.34	6.86	15.20	Mn(OH)2
Pyrolusite	-14.00	28.86	42.87	MnO2
Quartz	3.45	-0.67	-4.12	SiO2
Rhodochrosite	-1.79	-12.89	-11.10	MnCO3
Sepiolite	4.09	20.10	16.00	Mg2Si3O7.5OH:3H2O
Sepiolite(d)	1.44	20.10	18.66	Mg2Si3O7.5OH:3H2O
SiO2(a)	2.12	-0.67	-2.79	SiO2
Smithsonite	-3.13	-13.03	-9.90	ZnCO3
Talc	8.03	30.48	22.46	Mg3Si4O10(OH)2
Willemite	-3.32	12.77	16.09	Zn2SiO4
Witherite	-5.31	-13.90	-8.59	BaCO3
Zn(OH)2(e)	-4.78	6.72	11.50	Zn(OH)2

Initial solution 31.   4LArgentina-1

-----------------------------Solution composition------------------------------

Elements	Molality	Moles
Alkalinity	1.239e-003	1.239e-003
Ba	1.024e-008	1.024e-008
Ca	2.681e-004	2.681e-004
Cl	3.342e-003	3.342e-003
Cu	5.688e-008	5.688e-008
F	5.972e-006	5.972e-006
K	2.902e-005	2.902e-005
Mg	6.484e-004	6.484e-004
Mn	1.736e-007	1.736e-007
Na	2.188e-003	2.188e-003
S(6)	8.142e-005	8.142e-005
Si	6.350e-002	6.350e-002
Zn	1.690e-007	1.690e-007

----------------------------Description of solution----------------------------

pH = 7.000
pe = 4.000
Activity of water = 0.999
Ionic strength = 5.353e-003
Mass of water (kg) = 1.000e+000
Total carbon (mol/kg) = 1.466e-003
Total CO2 (mol/kg) = 1.466e-003
Temperature (deg C) = 13.300
Electrical balance (eq) = -6.989e-004
Percent error, 100*(Cat-|An|)/(Cat+|An|) = -7.98
Iterations = 9
Total H = 1.112676e+002
Total O = 5.576466e+001

----------------------------Distribution of species----------------------------

Species	Molality	Activity	Log Molality	Log Activity	Log Gamma
H+	1.072e-007	1.000e-007	-6.970	-7.000	-0.030
OH-	4.212e-008	3.897e-008	-7.376	-7.409	-0.034

H2O		5.551e+001	9.988e-001	1.744	-0.001	0.000
Ba	1.024e-008					
Ba+2		9.974e-009	7.382e-009	-8.001	-8.132	-0.131
BaSO4		2.036e-010	2.038e-010	-9.691	-9.691	0.001
BaHCO3+		5.703e-011	5.283e-011	-10.244	-10.277	-0.033
BaCO3		1.158e-012	1.160e-012	-11.936	-11.936	0.001
BaOH+		2.697e-015	2.498e-015	-14.569	-14.602	-0.033
C(4)	1.466e-003					
HCO3-		1.163e-003	1.079e-003	-2.935	-2.967	-0.032
CO2		2.924e-004	2.928e-004	-3.534	-3.533	0.001
MgHCO3+		6.231e-006	5.771e-006	-5.205	-5.239	-0.033
CaHCO3+		2.305e-006	2.139e-006	-5.637	-5.670	-0.032
NaHCO3		1.228e-006	1.230e-006	-5.911	-5.910	0.001
CO3-2		5.185e-007	3.847e-007	-6.285	-6.415	-0.130
MgCO3		1.453e-007	1.455e-007	-6.838	-6.837	0.001
CaCO3		1.055e-007	1.056e-007	-6.977	-6.976	0.001
ZnHCO3+		1.550e-008	1.436e-008	-7.810	-7.843	-0.033
MnHCO3+		1.203e-008	1.114e-008	-7.920	-7.953	-0.033
NaCO3-		8.480e-009	7.854e-009	-8.072	-8.105	-0.033
ZnCO3		8.105e-009	8.115e-009	-8.091	-8.091	0.001
MnCO3		3.536e-009	3.541e-009	-8.451	-8.451	0.001
Zn(CO3)2-2		9.068e-011	6.674e-011	-10.042	-10.176	-0.133
BaHCO3+		5.703e-011	5.283e-011	-10.244	-10.277	-0.033
BaCO3		1.158e-012	1.160e-012	-11.936	-11.936	0.001
Ca	2.681e-004					
Ca+2		2.637e-004	1.956e-004	-3.579	-3.709	-0.130
CaHCO3+		2.305e-006	2.139e-006	-5.637	-5.670	-0.032
CaSO4		1.916e-006	1.919e-006	-5.718	-5.717	0.001
CaCO3		1.055e-007	1.056e-007	-6.977	-6.976	0.001
CaF+		7.445e-009	6.895e-009	-8.128	-8.161	-0.033
CaOH+		3.500e-010	3.242e-010	-9.456	-9.489	-0.033
CaHSO4+		1.067e-012	9.882e-013	-11.972	-12.005	-0.033
Cl	3.342e-003					
Cl-		3.342e-003	3.093e-003	-2.476	-2.510	-0.034
MnCl+		1.576e-009	1.460e-009	-8.802	-8.836	-0.033
ZnCl+		5.553e-010	5.143e-010	-9.255	-9.289	-0.033
MnCl2		1.968e-012	1.971e-012	-11.706	-11.705	0.001
ZnCl2		1.584e-012	1.586e-012	-11.800	-11.800	0.001
ZnCl3-		5.523e-015	5.116e-015	-14.258	-14.291	-0.033
MnCl3-		1.812e-015	1.679e-015	-14.742	-14.775	-0.033
ZnCl4-2		9.783e-018	7.200e-018	-17.010	-17.143	-0.133
Cu(1)	8.028e-010					
Cu+		8.028e-010	7.415e-010	-9.095	-9.130	-0.035
Cu(2)	5.608e-008					
Cu(OH)2		3.295e-008	3.300e-008	-7.482	-7.482	0.001
Cu+2		2.126e-008	1.583e-008	-7.673	-7.800	-0.128
CuOH+		1.708e-009	1.581e-009	-8.768	-8.801	-0.033
CuSO4		1.635e-010	1.637e-010	-9.787	-9.786	0.001
Cu(OH)3-		2.144e-014	1.986e-014	-13.669	-13.702	-0.033
Cu(OH)4-2		5.377e-020	3.957e-020	-19.269	-19.403	-0.133
F	5.972e-006					
F-		5.812e-006	5.377e-006	-5.236	-5.269	-0.034
MgF+		1.460e-007	1.352e-007	-6.836	-6.869	-0.033
CaF+		7.445e-009	6.895e-009	-8.128	-8.161	-0.033
NaF		6.263e-009	6.270e-009	-8.203	-8.203	0.001
HF		6.559e-010	6.568e-010	-9.183	-9.183	0.001
MnF+		4.654e-012	4.311e-012	-11.332	-11.365	-0.033
HF2-		1.313e-014	1.216e-014	-13.882	-13.915	-0.033
SiF6-2		9.734e-031	7.165e-031	-30.012	-30.145	-0.133
H(0)	1.596e-025					
H2		7.982e-026	7.992e-026	-25.098	-25.097	0.001
K	2.902e-005					
K+		2.901e-005	2.684e-005	-4.537	-4.571	-0.034
KSO4-		9.070e-009	8.401e-009	-8.042	-8.076	-0.033
KOH		9.285e-013	9.296e-013	-12.032	-12.032	0.001
Mg	6.484e-004					
Mg+2		6.374e-004	4.744e-004	-3.196	-3.324	-0.128

	MgHCO3+		6.231e-006	5.771e-006	-5.205	-5.239	-0.033
	MgSO4		4.472e-006	4.477e-006	-5.350	-5.349	0.001
	MgF+		1.460e-007	1.352e-007	-6.836	-6.869	-0.033
	MgCO3		1.453e-007	1.455e-007	-6.838	-6.837	0.001
	MgOH+		6.185e-009	5.729e-009	-8.209	-8.242	-0.033
Mn(2)		1.736e-007					
	Mn+2		1.556e-007	1.159e-007	-6.808	-6.936	-0.128
	MnHCO3+		1.203e-008	1.114e-008	-7.920	-7.953	-0.033
	MnCO3		3.536e-009	3.541e-009	-8.451	-8.451	0.001
	MnCl+		1.576e-009	1.460e-009	-8.802	-8.836	-0.033
	MnSO4		8.986e-010	8.997e-010	-9.046	-9.046	0.001
	MnOH+		1.190e-011	1.102e-011	-10.924	-10.958	-0.033
	MnF+		4.654e-012	4.311e-012	-11.332	-11.365	-0.033
	MnCl2		1.968e-012	1.971e-012	-11.706	-11.705	0.001
	MnCl3-		1.812e-015	1.679e-015	-14.742	-14.775	-0.033
Mn(3)		1.205e-029					
	Mn+3		1.205e-029	6.047e-030	-28.919	-29.218	-0.300
Na		2.188e-003					
	Na+		2.186e-003	2.026e-003	-2.660	-2.693	-0.033
	NaHCO3		1.228e-006	1.230e-006	-5.911	-5.910	0.001
	NaSO4-		5.592e-007	5.179e-007	-6.252	-6.286	-0.033
	NaCO3-		8.480e-009	7.854e-009	-8.072	-8.105	-0.033
	NaF		6.263e-009	6.270e-009	-8.203	-8.203	0.001
	NaOH		1.336e-010	1.337e-010	-9.874	-9.874	0.001
O(0)		0.000e+000					
	O2		0.000e+000	0.000e+000	-46.117	-46.116	0.001
S(6)		8.142e-005					
	SO4-2		7.446e-005	5.509e-005	-4.128	-4.259	-0.131
	MgSO4		4.472e-006	4.477e-006	-5.350	-5.349	0.001
	CaSO4		1.916e-006	1.919e-006	-5.718	-5.717	0.001
	NaSO4-		5.592e-007	5.179e-007	-6.252	-6.286	-0.033
	KSO4-		9.070e-009	8.401e-009	-8.042	-8.076	-0.033
	ZnSO4		1.241e-009	1.243e-009	-8.906	-8.906	0.001
	MnSO4		8.986e-010	8.997e-010	-9.046	-9.046	0.001
	HSO4-		4.537e-010	4.203e-010	-9.343	-9.376	-0.033
	BaSO4		2.036e-010	2.038e-010	-9.691	-9.691	0.001
	CuSO4		1.635e-010	1.637e-010	-9.787	-9.786	0.001
	CaHSO4+		1.067e-012	9.882e-013	-11.972	-12.005	-0.033
	Zn(SO4)2-2		8.305e-013	6.113e-013	-12.081	-12.214	-0.133
Si		6.350e-002					
	H4SiO4		6.344e-002	6.352e-002	-1.198	-1.197	0.001
	H3SiO4-		6.513e-005	6.033e-005	-4.186	-4.219	-0.033
	H2SiO4-2		2.463e-011	1.813e-011	-10.609	-10.742	-0.133
	SiF6-2		9.734e-031	7.165e-031	-30.012	-30.145	-0.133
Zn		1.690e-007					
	Zn+2		1.428e-007	1.057e-007	-6.845	-6.976	-0.131
	ZnHCO3+		1.550e-008	1.436e-008	-7.810	-7.843	-0.033
	ZnCO3		8.105e-009	8.115e-009	-8.091	-8.091	0.001
	ZnSO4		1.241e-009	1.243e-009	-8.906	-8.906	0.001
	ZnCl+		5.553e-010	5.143e-010	-9.255	-9.289	-0.033
	ZnOH+		4.963e-010	4.597e-010	-9.304	-9.338	-0.033
	Zn(OH)2		1.326e-010	1.328e-010	-9.877	-9.877	0.001
	Zn(CO3)2-2		9.068e-011	6.674e-011	-10.042	-10.176	-0.133
	ZnCl2		1.584e-012	1.586e-012	-11.800	-11.800	0.001
	Zn(SO4)2-2		8.305e-013	6.113e-013	-12.081	-12.214	-0.133
	ZnCl3-		5.523e-015	5.116e-015	-14.258	-14.291	-0.033
	Zn(OH)3-		4.527e-015	4.193e-015	-14.344	-14.377	-0.033
	ZnCl4-2		9.783e-018	7.200e-018	-17.010	-17.143	-0.133
	Zn(OH)4-2		9.019e-021	6.638e-021	-20.045	-20.178	-0.133

------------------------------Saturation indices-------------------------------

Phase	SI	log IAP	log KT	
Anhydrite	-3.63	-7.97	-4.33	CaSO4
Aragonite	-1.85	-10.12	-8.27	CaCO3
Barite	-2.21	-12.39	-10.18	BaSO4

Calcite	-1.70	-10.12	-8.42	CaCO3
Chalcedony	2.50	-1.20	-3.69	SiO2
Chrysotile	-4.07	29.63	33.71	Mg3Si2O5(OH)4
CO2(g)	-2.22	-3.53	-1.32	CO2
Dolomite	-3.05	-19.86	-16.81	CaMg(CO3)2
Fluorite	-3.50	-14.25	-10.75	CaF2
Gypsum	-3.38	-7.97	-4.59	CaSO4:2H2O
H2(g)	-22.00	-25.10	-3.10	H2
H2O(g)	-1.83	-0.00	1.83	H2O
Halite	-6.76	-5.20	1.55	NaCl
Hausmannite	-20.85	43.19	64.04	Mn3O4
Manganite	-7.28	18.06	25.34	MnOOH
O2(g)	-43.21	-46.12	-2.90	O2
Pyrochroite	-8.14	7.06	15.20	Mn(OH)2
Pyrolusite	-14.27	29.06	43.33	MnO2
Quartz	2.96	-1.20	-4.16	SiO2
Rhodochrosite	-2.26	-13.35	-11.09	MnCO3
Sepiolite	1.68	17.76	16.08	Mg2Si3O7.5OH:3H2O
Sepiolite(d)	-0.90	17.76	18.66	Mg2Si3O7.5OH:3H2O
SiO2(a)	1.62	-1.20	-2.81	SiO2
Smithsonite	-3.52	-13.39	-9.87	ZnCO3
Talc	4.46	27.24	22.79	Mg3Si4O10(OH)2
Willemite	-3.48	12.85	16.33	Zn2SiO4
Witherite	-5.94	-14.55	-8.61	BaCO3
Zn(OH)2(e)	-4.48	7.02	11.50	Zn(OH)2

Initial solution 32.   4LTrinidad-1

----------------------------Solution composition-----------------------------

Elements	Molality	Moles
Alkalinity	1.337e-002	1.337e-002
Ba	5.848e-009	5.848e-009
Ca	5.009e-004	5.009e-004
Cl	2.352e-003	2.352e-003
Cu	5.529e-008	5.529e-008
F	5.812e-006	5.812e-006
K	7.779e-005	7.779e-005
Mg	3.617e-003	3.617e-003
Mn	1.517e-007	1.517e-007
Na	6.725e-003	6.725e-003
S(6)	1.923e-005	1.923e-005
Si	4.511e-002	4.511e-002
Zn	2.334e-007	2.334e-007

---------------------------Description of solution---------------------------

pH	=	7.000
pe	=	4.000
Activity of water	=	0.999
Ionic strength	=	1.881e-002
Mass of water (kg)	=	1.000e+000
Total carbon (mol/kg)	=	1.640e-002
Total CO2 (mol/kg)	=	1.640e-002
Temperature (deg C)	=	13.000
Electrical balance (eq)	=	-7.283e-004
Percent error, 100*(Cat-|An|)/(Cat+|An|)	=	-2.43
Iterations	=	11
Total H	=	1.112061e+002
Total O	=	5.573285e+001

---------------------------Distribution of species---------------------------

Species	Molality	Activity	Log Molality	Log Activity	Log Gamma

H+	1.119e-007	1.000e-007	-6.951	-7.000	-0.049
OH-	4.353e-008	3.798e-008	-7.361	-7.420	-0.059
H2O	5.551e+001	9.987e-001	1.744	-0.001	0.000
Ba 5.848e-009					
Ba+2	5.544e-009	3.310e-009	-8.256	-8.480	-0.224
BaHCO3+	2.826e-010	2.476e-010	-9.549	-9.606	-0.057
BaSO4	1.513e-011	1.520e-011	-10.820	-10.818	0.002
BaCO3	5.387e-012	5.410e-012	-11.269	-11.267	0.002
BaOH+	1.279e-015	1.120e-015	-14.893	-14.951	-0.057
C(4) 1.640e-002					
HCO3-	1.292e-002	1.138e-002	-1.889	-1.944	-0.055
CO2	3.094e-003	3.108e-003	-2.509	-2.508	0.002
MgHCO3+	2.947e-004	2.582e-004	-3.531	-3.588	-0.057
NaHCO3	3.740e-005	3.756e-005	-4.427	-4.425	0.002
CaHCO3+	3.618e-005	3.187e-005	-4.441	-4.497	-0.055
CO3-2	6.692e-006	4.026e-006	-5.174	-5.395	-0.221
MgCO3	6.405e-006	6.433e-006	-5.193	-5.192	0.002
CaCO3	1.561e-006	1.568e-006	-5.807	-5.805	0.002
NaCO3-	2.672e-007	2.341e-007	-6.573	-6.631	-0.057
ZnHCO3+	9.006e-008	7.890e-008	-7.045	-7.103	-0.057
MnHCO3+	5.607e-008	4.912e-008	-7.251	-7.309	-0.057
ZnCO3	4.404e-008	4.423e-008	-7.356	-7.354	0.002
MnCO3	1.542e-008	1.548e-008	-7.812	-7.810	0.002
Zn(CO3)2-2	6.461e-009	3.806e-009	-8.190	-8.419	-0.230
BaHCO3+	2.826e-010	2.476e-010	-9.549	-9.606	-0.057
BaCO3	5.387e-012	5.410e-012	-11.269	-11.267	0.002
Ca 5.009e-004					
Ca+2	4.627e-004	2.783e-004	-3.335	-3.556	-0.221
CaHCO3+	3.618e-005	3.187e-005	-4.441	-4.497	-0.055
CaCO3	1.561e-006	1.568e-006	-5.807	-5.805	0.002
CaSO4	4.506e-007	4.526e-007	-6.346	-6.344	0.002
CaF+	9.438e-009	8.268e-009	-8.025	-8.083	-0.057
CaOH+	5.264e-010	4.612e-010	-9.279	-9.336	-0.057
CaHSO4+	2.653e-013	2.324e-013	-12.576	-12.634	-0.057
Cl 2.352e-003					
Cl-	2.352e-003	2.054e-003	-2.628	-2.687	-0.059
MnCl+	4.625e-010	4.052e-010	-9.335	-9.392	-0.057
ZnCl+	2.002e-010	1.754e-010	-9.699	-9.756	-0.057
MnCl2	3.617e-013	3.632e-013	-12.442	-12.440	0.002
ZnCl2	3.571e-013	3.587e-013	-12.447	-12.445	0.002
ZnCl3-	8.752e-016	7.667e-016	-15.058	-15.115	-0.057
MnCl3-	2.345e-016	2.055e-016	-15.630	-15.687	-0.057
ZnCl4-2	1.213e-018	7.148e-019	-17.916	-18.146	-0.230
Cu(1) 7.651e-010					
Cu+	7.651e-010	6.638e-010	-9.116	-9.178	-0.062
Cu(2) 5.453e-008					
Cu(OH)2	2.950e-008	2.963e-008	-7.530	-7.528	0.002
Cu+2	2.338e-008	1.422e-008	-7.631	-7.847	-0.216
CuOH+	1.623e-009	1.420e-009	-8.790	-8.848	-0.058
CuSO4	2.428e-011	2.439e-011	-10.615	-10.613	0.002
Cu(OH)3-	2.035e-014	1.783e-014	-13.691	-13.749	-0.057
Cu(OH)4-2	6.031e-020	3.553e-020	-19.220	-19.449	-0.230
F 5.812e-006					
F-	5.234e-006	4.567e-006	-5.281	-5.340	-0.059
MgF+	5.529e-007	4.844e-007	-6.257	-6.315	-0.057
NaF	1.535e-008	1.542e-008	-7.814	-7.812	0.002
CaF+	9.438e-009	8.268e-009	-8.025	-8.083	-0.057
HF	5.525e-010	5.549e-010	-9.258	-9.256	0.002
MnF+	1.746e-012	1.530e-012	-11.758	-11.815	-0.057
HF2-	9.926e-015	8.697e-015	-14.003	-14.061	-0.057
SiF6-2	3.351e-031	1.974e-031	-30.475	-30.705	-0.230
H(0) 1.597e-025					
H2	7.983e-026	8.018e-026	-25.098	-25.096	0.002
K 7.779e-005					
K+	7.779e-005	6.791e-005	-4.109	-4.168	-0.059
KSO4-	4.011e-009	3.514e-009	-8.397	-8.454	-0.057
KOH	2.342e-012	2.352e-012	-11.630	-11.629	0.002

Mg	3.617e-003					
Mg+2		3.312e-003	2.013e-003	-2.480	-2.696	-0.216
MgHCO3+		2.947e-004	2.582e-004	-3.531	-3.588	-0.057
MgCO3		6.405e-006	6.433e-006	-5.193	-5.192	0.002
MgSO4		3.120e-006	3.133e-006	-5.506	-5.504	0.002
MgF+		5.529e-007	4.844e-007	-6.257	-6.315	-0.057
MgOH+		2.695e-008	2.361e-008	-7.570	-7.627	-0.057
Mn(2)	1.517e-007					
Mn+2		7.964e-008	4.843e-008	-7.099	-7.315	-0.216
MnHCO3+		5.607e-008	4.912e-008	-7.251	-7.309	-0.057
MnCO3		1.542e-008	1.548e-008	-7.812	-7.810	0.002
MnCl+		4.625e-010	4.052e-010	-9.335	-9.392	-0.057
MnSO4		6.188e-011	6.215e-011	-10.208	-10.207	0.002
MnOH+		5.121e-012	4.486e-012	-11.291	-11.348	-0.057
MnF+		1.746e-012	1.530e-012	-11.758	-11.815	-0.057
MnCl2		3.617e-013	3.632e-013	-12.442	-12.440	0.002
MnCl3-		2.345e-016	2.055e-016	-15.630	-15.687	-0.057
Mn(3)	7.926e-030					
Mn+3		7.926e-030	2.410e-030	-29.101	-29.618	-0.517
Na	6.725e-003					
Na+		6.687e-003	5.868e-003	-2.175	-2.232	-0.057
NaHCO3		3.740e-005	3.756e-005	-4.427	-4.425	0.002
NaSO4-		2.841e-007	2.489e-007	-6.547	-6.604	-0.057
NaCO3-		2.672e-007	2.341e-007	-6.573	-6.631	-0.057
NaF		1.535e-008	1.542e-008	-7.814	-7.812	0.002
NaOH		3.855e-010	3.872e-010	-9.414	-9.412	0.002
O(0)	0.000e+000					
O2		0.000e+000	0.000e+000	-46.226	-46.224	0.002
S(6)	1.923e-005					
SO4-2		1.537e-005	9.161e-006	-4.813	-5.038	-0.225
MgSO4		3.120e-006	3.133e-006	-5.506	-5.504	0.002
CaSO4		4.506e-007	4.526e-007	-6.346	-6.344	0.002
NaSO4-		2.841e-007	2.489e-007	-6.547	-6.604	-0.057
KSO4-		4.011e-009	3.514e-009	-8.397	-8.454	-0.057
ZnSO4		1.069e-010	1.074e-010	-9.971	-9.969	0.002
HSO4-		7.931e-011	6.948e-011	-10.101	-10.158	-0.057
MnSO4		6.188e-011	6.215e-011	-10.208	-10.207	0.002
CuSO4		2.428e-011	2.439e-011	-10.615	-10.613	0.002
BaSO4		1.513e-011	1.520e-011	-10.820	-10.818	0.002
CaHSO4+		2.653e-013	2.324e-013	-12.576	-12.634	-0.057
Zn(SO4)2-2		1.495e-014	8.805e-015	-13.825	-14.055	-0.230
Si	4.511e-002					
H4SiO4		4.506e-002	4.526e-002	-1.346	-1.344	0.002
H3SiO4-		4.847e-005	4.246e-005	-4.315	-4.372	-0.057
H2SiO4-2		2.118e-011	1.248e-011	-10.674	-10.904	-0.230
SiF6-2		3.351e-031	1.974e-031	-30.475	-30.705	-0.230
Zn	2.334e-007					
Zn+2		9.222e-008	5.506e-008	-7.035	-7.259	-0.224
ZnHCO3+		9.006e-008	7.890e-008	-7.045	-7.103	-0.057
ZnCO3		4.404e-008	4.423e-008	-7.356	-7.354	0.002
Zn(CO3)2-2		6.461e-009	3.806e-009	-8.190	-8.419	-0.230
ZnOH+		2.666e-010	2.336e-010	-9.574	-9.632	-0.057
ZnCl+		2.002e-010	1.754e-010	-9.699	-9.756	-0.057
ZnSO4		1.069e-010	1.074e-010	-9.971	-9.969	0.002
Zn(OH)2		6.885e-011	6.915e-011	-10.162	-10.160	0.002
ZnCl2		3.571e-013	3.587e-013	-12.447	-12.445	0.002
Zn(SO4)2-2		1.495e-014	8.805e-015	-13.825	-14.055	-0.230
Zn(OH)3-		2.493e-015	2.184e-015	-14.603	-14.661	-0.057
ZnCl3-		8.752e-016	7.667e-016	-15.058	-15.115	-0.057
ZnCl4-2		1.213e-018	7.148e-019	-17.916	-18.146	-0.230
Zn(OH)4-2		5.867e-021	3.457e-021	-20.232	-20.461	-0.230

------------------------------Saturation indices-------------------------------

Phase	SI	log IAP	log KT	
Anhydrite	-4.26	-8.59	-4.33	CaSO4

Aragonite	-0.68	-8.95	-8.27	CaCO3
Barite	-3.33	-13.52	-10.18	BaSO4
Calcite	-0.53	-8.95	-8.42	CaCO3
Chalcedony	2.35	-1.34	-3.70	SiO2
Chrysotile	-2.53	31.22	33.75	Mg3Si2O5(OH)4
CO2(g)	-1.19	-2.51	-1.31	CO2
Dolomite	-0.24	-17.04	-16.80	CaMg(CO3)2
Fluorite	-3.48	-14.24	-10.75	CaF2
Gypsum	-4.01	-8.59	-4.59	CaSO4:2H2O
H2(g)	-22.00	-25.10	-3.10	H2
H2O(g)	-1.83	-0.00	1.83	H2O
Halite	-6.47	-4.92	1.55	NaCl
Hausmannite	-22.07	42.05	64.12	Mn3O4
Manganite	-7.66	17.68	25.34	MnOOH
O2(g)	-43.32	-46.22	-2.90	O2
Pyrochroite	-8.52	6.68	15.20	Mn(OH)2
Pyrolusite	-14.70	28.68	43.38	MnO2
Quartz	2.82	-1.34	-4.16	SiO2
Rhodochrosite	-1.62	-12.71	-11.09	MnCO3
Sepiolite	2.49	18.58	16.09	Mg2Si3O7.5OH:3H2O
Sepiolite(d)	-0.08	18.58	18.66	Mg2Si3O7.5OH:3H2O
SiO2(a)	1.47	-1.34	-2.81	SiO2
Smithsonite	-2.79	-12.65	-9.87	ZnCO3
Talc	5.71	28.54	22.82	Mg3Si4O10(OH)2
Willemite	-4.22	12.14	16.36	Zn2SiO4
Witherite	-5.27	-13.88	-8.61	BaCO3
Zn(OH)2(e)	-4.76	6.74	11.50	Zn(OH)2

Initial solution 33.   4LJoaquina-1

----------------------------Solution composition-----------------------------

Elements	Molality	Moles
Alkalinity	1.812e-003	1.812e-003
Ba	4.396e-009	4.396e-009
Ca	2.410e-004	2.410e-004
Cl	1.330e-003	1.330e-003
Cu	3.009e-008	3.009e-008
F	6.833e-006	6.833e-006
K	3.551e-005	3.551e-005
Mg	5.505e-004	5.505e-004
Mn	1.026e-007	1.026e-007
Na	1.746e-003	1.746e-003
S(6)	9.323e-005	9.323e-005
Si	1.005e-001	1.005e-001
Zn	1.678e-007	1.678e-007

---------------------------Description of solution---------------------------

pH = 7.000
pe = 4.000
Activity of water = 0.998
Ionic strength = 4.187e-003
Mass of water (kg) = 1.000e+000
Total carbon (mol/kg) = 2.217e-003
Total CO2 (mol/kg) = 2.217e-003
Temperature (deg C) = 8.100
Electrical balance (eq) = 2.965e-005
Percent error, 100*(Cat-|An|)/(Cat+|An|) = 0.45
Iterations = 9
Total H = 1.114160e+002
Total O = 5.591470e+001

---------------------------Distribution of species---------------------------

Log   Log   Log

Species		Molality	Activity	Molality	Activity	Gamma
H+		1.064e-007	1.000e-007	-6.973	-7.000	-0.027
OH-		2.637e-008	2.462e-008	-7.579	-7.609	-0.030
H2O		5.551e+001	9.982e-001	1.744	-0.001	0.000
Ba	4.396e-009					
Ba+2		4.256e-009	3.257e-009	-8.371	-8.487	-0.116
BaSO4		1.081e-010	1.082e-010	-9.966	-9.966	0.000
BaHCO3+		3.152e-011	2.945e-011	-10.501	-10.531	-0.030
BaCO3		5.910e-013	5.916e-013	-12.228	-12.228	0.000
BaOH+		1.179e-015	1.102e-015	-14.928	-14.958	-0.030
C(4)	2.217e-003					
HCO3-		1.716e-003	1.606e-003	-2.765	-2.794	-0.029
CO2		4.881e-004	4.886e-004	-3.311	-3.311	0.000
MgHCO3+		7.967e-006	7.443e-006	-5.099	-5.128	-0.030
CaHCO3+		2.730e-006	2.555e-006	-5.564	-5.593	-0.029
NaHCO3		1.471e-006	1.473e-006	-5.832	-5.832	0.000
CO3-2		6.438e-007	4.936e-007	-6.191	-6.307	-0.115
MgCO3		1.502e-007	1.503e-007	-6.823	-6.823	0.000
CaCO3		1.208e-007	1.209e-007	-6.918	-6.918	0.000
ZnHCO3+		2.211e-008	2.065e-008	-7.655	-7.685	-0.030
MnHCO3+		1.043e-008	9.745e-009	-7.982	-8.011	-0.030
ZnCO3		1.005e-008	1.006e-008	-7.998	-7.997	0.000
NaCO3-		6.499e-009	6.072e-009	-8.187	-8.217	-0.030
MnCO3		2.667e-009	2.670e-009	-8.574	-8.574	0.000
Zn(CO3)2-2		1.394e-010	1.062e-010	-9.856	-9.974	-0.118
BaHCO3+		3.152e-011	2.945e-011	-10.501	-10.531	-0.030
BaCO3		5.910e-013	5.916e-013	-12.228	-12.228	0.000
Ca	2.410e-004					
Ca+2		2.361e-004	1.810e-004	-3.627	-3.742	-0.116
CaHCO3+		2.730e-006	2.555e-006	-5.564	-5.593	-0.029
CaSO4		2.022e-006	2.024e-006	-5.694	-5.694	0.000
CaCO3		1.208e-007	1.209e-007	-6.918	-6.918	0.000
CaF+		6.936e-009	6.480e-009	-8.159	-8.188	-0.030
CaOH+		3.209e-010	2.998e-010	-9.494	-9.523	-0.030
CaHSO4+		1.067e-012	9.966e-013	-11.972	-12.001	-0.030
Cl	1.330e-003					
Cl-		1.330e-003	1.242e-003	-2.876	-2.906	-0.030
MnCl+		3.688e-010	3.445e-010	-9.433	-9.463	-0.030
ZnCl+		1.659e-010	1.550e-010	-9.780	-9.810	-0.030
ZnCl2		1.874e-013	1.876e-013	-12.727	-12.727	0.000
MnCl2		1.866e-013	1.868e-013	-12.729	-12.729	0.000
ZnCl3-		2.513e-016	2.348e-016	-15.600	-15.629	-0.030
MnCl3-		6.840e-017	6.390e-017	-16.165	-16.194	-0.030
ZnCl4-2		1.665e-019	1.268e-019	-18.779	-18.897	-0.118
Cu(1)	4.040e-010					
Cu+		4.040e-010	3.766e-010	-9.394	-9.424	-0.031
Cu(2)	2.968e-008					
Cu(OH)2		1.764e-008	1.766e-008	-7.753	-7.753	0.000
Cu+2		1.103e-008	8.484e-009	-7.957	-8.071	-0.114
CuOH+		9.066e-010	8.469e-010	-9.043	-9.072	-0.030
CuSO4		1.013e-010	1.014e-010	-9.994	-9.994	0.000
Cu(OH)3-		1.137e-014	1.062e-014	-13.944	-13.974	-0.030
Cu(OH)4-2		2.777e-020	2.116e-020	-19.556	-19.675	-0.118
F	6.833e-006					
F-		6.687e-006	6.242e-006	-5.175	-5.205	-0.030
MgF+		1.320e-007	1.233e-007	-6.879	-6.909	-0.030
CaF+		6.936e-009	6.480e-009	-8.159	-8.188	-0.030
NaF		5.852e-009	5.857e-009	-8.233	-8.232	0.000
HF		6.977e-010	6.984e-010	-9.156	-9.156	0.000
MnF+		3.147e-012	2.940e-012	-11.502	-11.532	-0.030
HF2-		1.513e-014	1.413e-014	-13.820	-13.850	-0.030
SiF6-2		6.189e-030	4.715e-030	-29.208	-29.327	-0.118
H(0)	1.691e-025					
H2		8.454e-026	8.462e-026	-25.073	-25.073	0.000
K	3.551e-005					
K+		3.550e-005	3.314e-005	-4.450	-4.480	-0.030

```
   KSO4-                1.208e-008  1.129e-008    -7.918    -7.947    -0.030
   KOH                  1.146e-012  1.147e-012   -11.941   -11.940     0.000
Mg               5.505e-004
   Mg+2                 5.382e-004  4.137e-004    -3.269    -3.383    -0.114
   MgHCO3+              7.967e-006  7.443e-006    -5.099    -5.128    -0.030
   MgSO4                4.047e-006  4.051e-006    -5.393    -5.392     0.000
   MgCO3                1.502e-007  1.503e-007    -6.823    -6.823     0.000
   MgF+                 1.320e-007  1.233e-007    -6.879    -6.909    -0.030
   MgOH+                3.183e-009  2.974e-009    -8.497    -8.527    -0.030
Mn(2)            1.026e-007
   Mn+2                 8.853e-008  6.809e-008    -7.053    -7.167    -0.114
   MnHCO3+              1.043e-008  9.745e-009    -7.982    -8.011    -0.030
   MnCO3                2.667e-009  2.670e-009    -8.574    -8.574     0.000
   MnSO4                5.695e-010  5.701e-010    -9.244    -9.244     0.000
   MnCl+                3.688e-010  3.445e-010    -9.433    -9.463    -0.030
   MnOH+                4.341e-012  4.055e-012   -11.362   -11.392    -0.030
   MnF+                 3.147e-012  2.940e-012   -11.502   -11.532    -0.030
   MnCl2                1.866e-013  1.868e-013   -12.729   -12.729     0.000
   MnCl3-               6.840e-017  6.390e-017   -16.165   -16.194    -0.030
Mn(3)            2.835e-030
   Mn+3                 2.835e-030  1.537e-030   -29.547   -29.813    -0.266
Na               1.746e-003
   Na+                  1.744e-003  1.631e-003    -2.758    -2.788    -0.029
   NaHCO3               1.471e-006  1.473e-006    -5.832    -5.832     0.000
   NaSO4-               5.174e-007  4.834e-007    -6.286    -6.316    -0.030
   NaCO3-               6.499e-009  6.072e-009    -8.187    -8.217    -0.030
   NaF                  5.852e-009  5.857e-009    -8.233    -8.232     0.000
   NaOH                 1.074e-010  1.075e-010    -9.969    -9.968     0.000
O(0)             0.000e+000
   O2                   0.000e+000  0.000e+000   -48.019   -48.018     0.000
S(6)             9.323e-005
   SO4-2                8.663e-005  6.627e-005    -4.062    -4.179    -0.116
   MgSO4                4.047e-006  4.051e-006    -5.393    -5.392     0.000
   CaSO4                2.022e-006  2.024e-006    -5.694    -5.694     0.000
   NaSO4-               5.174e-007  4.834e-007    -6.286    -6.316    -0.030
   KSO4-                1.208e-008  1.129e-008    -7.918    -7.947    -0.030
   ZnSO4                1.381e-009  1.383e-009    -8.860    -8.859     0.000
   MnSO4                5.695e-010  5.701e-010    -9.244    -9.244     0.000
   HSO4-                4.902e-010  4.580e-010    -9.310    -9.339    -0.030
   BaSO4                1.081e-010  1.082e-010    -9.966    -9.966     0.000
   CuSO4                1.013e-010  1.014e-010    -9.994    -9.994     0.000
   Zn(SO4)2-2           1.122e-012  8.548e-013   -11.950   -12.068    -0.118
   CaHSO4+              1.067e-012  9.966e-013   -11.972   -12.001    -0.030
Si               1.005e-001
   H4SiO4               1.004e-001  1.005e-001    -0.998    -0.998     0.000
   H3SiO4-              8.210e-005  7.671e-005    -4.086    -4.115    -0.030
   H2SiO4-2             2.026e-011  1.544e-011   -10.693   -10.811    -0.118
   SiF6-2               6.189e-030  4.715e-030   -29.208   -29.327    -0.118
Zn               1.678e-007
   Zn+2                 1.335e-007  1.022e-007    -6.875    -6.991    -0.116
   ZnHCO3+              2.211e-008  2.065e-008    -7.655    -7.685    -0.030
   ZnCO3                1.005e-008  1.006e-008    -7.998    -7.997     0.000
   ZnSO4                1.381e-009  1.383e-009    -8.860    -8.859     0.000
   ZnOH+                3.075e-010  2.873e-010    -9.512    -9.542    -0.030
   ZnCl+                1.659e-010  1.550e-010    -9.780    -9.810    -0.030
   Zn(CO3)2-2           1.394e-010  1.062e-010    -9.856    -9.974    -0.118
   Zn(OH)2              1.280e-010  1.282e-010    -9.893    -9.892     0.000
   Zn(SO4)2-2           1.122e-012  8.548e-013   -11.950   -12.068    -0.118
   ZnCl2                1.874e-013  1.876e-013   -12.727   -12.727     0.000
   Zn(OH)3-             4.330e-015  4.045e-015   -14.364   -14.393    -0.030
   ZnCl3-               2.513e-016  2.348e-016   -15.600   -15.629    -0.030
   ZnCl4-2              1.665e-019  1.268e-019   -18.779   -18.897    -0.118
   Zn(OH)4-2            8.400e-021  6.400e-021   -20.076   -20.194    -0.118

------------------------------Saturation indices-------------------------------

      Phase                SI log IAP  log KT
```

Anhydrite	-3.58	-7.92	-4.34	CaSO4
Aragonite	-1.80	-10.05	-8.25	CaCO3
Barite	-2.38	-12.67	-10.29	BaSO4
Calcite	-1.65	-10.05	-8.40	CaCO3
Chalcedony	2.76	-1.00	-3.76	SiO2
Chrysotile	-4.56	29.85	34.42	Mg3Si2O5(OH)4
CO2(g)	-2.07	-3.31	-1.24	CO2
Dolomite	-3.06	-19.74	-16.67	CaMg(CO3)2
Fluorite	-3.33	-14.15	-10.83	CaF2
Gypsum	-3.33	-7.92	-4.60	CaSO4:2H2O
H2(g)	-22.00	-25.07	-3.07	H2
H2O(g)	-1.97	-0.00	1.97	H2O
Halite	-7.24	-5.69	1.54	NaCl
Hausmannite	-22.97	42.50	65.46	Mn3O4
Manganite	-7.51	17.83	25.34	MnOOH
O2(g)	-45.14	-48.02	-2.88	O2
Pyrochroite	-8.37	6.83	15.20	Mn(OH)2
Pyrolusite	-15.42	28.83	44.25	MnO2
Quartz	3.25	-1.00	-4.24	SiO2
Rhodochrosite	-2.41	-13.47	-11.07	MnCO3
Sepiolite	2.01	18.24	16.23	Mg2Si3O7.5OH:3H2O
Sepiolite(d)	-0.42	18.24	18.66	Mg2Si3O7.5OH:3H2O
SiO2(a)	1.86	-1.00	-2.86	SiO2
Smithsonite	-3.49	-13.30	-9.81	ZnCO3
Talc	4.42	27.86	23.44	Mg3Si4O10(OH)2
Willemite	-3.78	13.02	16.80	Zn2SiO4
Witherite	-6.15	-14.79	-8.65	BaCO3
Zn(OH)2(e)	-4.49	7.01	11.50	Zn(OH)2

Initial solution 34.   4LNorma-1

-----------------------------Solution composition------------------------------

Elements	Molality	Moles
Alkalinity	9.405e-004	9.405e-004
Ba	6.571e-009	6.571e-009
Ca	1.476e-004	1.476e-004
Cl	1.045e-003	1.045e-003
Cu	3.472e-008	3.472e-008
F	5.120e-006	5.120e-006
K	1.923e-005	1.923e-005
Mg	3.642e-004	3.642e-004
Mn	2.154e-007	2.154e-007
Na	1.056e-003	1.056e-003
S(6)	4.676e-005	4.676e-005
Si	4.339e-002	4.339e-002
Zn	1.703e-007	1.703e-007

----------------------------Description of solution----------------------------

pH	=	7.000
pe	=	4.000
Activity of water	=	0.999
Ionic strength	=	2.633e-003
Mass of water (kg)	=	1.000e+000
Total carbon (mol/kg)	=	1.175e-003
Total CO2 (mol/kg)	=	1.175e-003
Temperature (deg C)	=	6.800
Electrical balance (eq)	=	1.512e-005
Percent error, 100*(Cat-|An|)/(Cat+|An|)	=	0.36
Iterations	=	9
Total H	=	1.111869e+002
Total O	=	5.568323e+001

----------------------------Distribution of species----------------------------

Species		Molality	Activity	Log Molality	Log Activity	Log Gamma
H+		1.052e-007	1.000e-007	-6.978	-7.000	-0.022
OH-		2.313e-008	2.188e-008	-7.636	-7.660	-0.024
H2O		5.551e+001	9.992e-001	1.744	-0.000	0.000
Ba	6.571e-009					
Ba+2		6.452e-009	5.198e-009	-8.190	-8.284	-0.094
BaSO4		9.314e-011	9.320e-011	-10.031	-10.031	0.000
BaHCO3+		2.538e-011	2.403e-011	-10.595	-10.619	-0.024
BaCO3		4.704e-013	4.707e-013	-12.328	-12.327	0.000
BaOH+		1.859e-015	1.760e-015	-14.731	-14.754	-0.024
C(4)	1.175e-003					
HCO3-		9.023e-004	8.551e-004	-3.045	-3.068	-0.023
CO2		2.681e-004	2.683e-004	-3.572	-3.571	0.000
MgHCO3+		2.940e-006	2.783e-006	-5.532	-5.556	-0.024
CaHCO3+		8.969e-007	8.500e-007	-6.047	-6.071	-0.023
NaHCO3		4.801e-007	4.804e-007	-6.319	-6.318	0.000
CO3-2		3.131e-007	2.526e-007	-6.504	-6.598	-0.093
MgCO3		5.293e-008	5.296e-008	-7.276	-7.276	0.000
CaCO3		4.003e-008	4.006e-008	-7.398	-7.397	0.000
ZnHCO3+		1.366e-008	1.293e-008	-7.865	-7.888	-0.024
MnHCO3+		1.288e-008	1.219e-008	-7.890	-7.914	-0.024
ZnCO3		6.048e-009	6.052e-009	-8.218	-8.218	0.000
MnCO3		3.208e-009	3.210e-009	-8.494	-8.494	0.000
NaCO3-		1.867e-009	1.768e-009	-8.729	-8.753	-0.024
Zn(CO3)2-2		4.069e-011	3.268e-011	-10.391	-10.486	-0.095
BaHCO3+		2.538e-011	2.403e-011	-10.595	-10.619	-0.024
BaCO3		4.704e-013	4.707e-013	-12.328	-12.327	0.000
Ca	1.476e-004					
Ca+2		1.460e-004	1.177e-004	-3.836	-3.929	-0.093
CaHCO3+		8.969e-007	8.500e-007	-6.047	-6.071	-0.023
CaSO4		7.007e-007	7.011e-007	-6.154	-6.154	0.000
CaCO3		4.003e-008	4.006e-008	-7.398	-7.397	0.000
CaF+		3.290e-009	3.114e-009	-8.483	-8.507	-0.024
CaOH+		2.062e-010	1.952e-010	-9.686	-9.709	-0.024
CaHSO4+		3.609e-013	3.417e-013	-12.443	-12.466	-0.024
Cl	1.045e-003					
Cl-		1.045e-003	9.888e-004	-2.981	-3.005	-0.024
MnCl+		6.807e-010	6.444e-010	-9.167	-9.191	-0.024
ZnCl+		1.436e-010	1.360e-010	-9.843	-9.867	-0.024
MnCl2		2.780e-013	2.781e-013	-12.556	-12.556	0.000
ZnCl2		1.301e-013	1.302e-013	-12.886	-12.885	0.000
ZnCl3-		1.359e-016	1.286e-016	-15.867	-15.891	-0.024
MnCl3-		8.002e-017	7.575e-017	-16.097	-16.121	-0.024
ZnCl4-2		6.806e-020	5.467e-020	-19.167	-19.262	-0.095
Cu(1)	4.621e-010					
Cu+		4.621e-010	4.368e-010	-9.335	-9.360	-0.024
Cu(2)	3.425e-008					
Cu(OH)2		2.080e-008	2.081e-008	-7.682	-7.682	0.000
Cu+2		1.234e-008	9.976e-009	-7.909	-8.001	-0.092
CuOH+		1.053e-009	9.968e-010	-8.978	-9.001	-0.024
CuSO4		6.369e-011	6.373e-011	-10.196	-10.196	0.000
Cu(OH)3-		1.323e-014	1.253e-014	-13.878	-13.902	-0.024
Cu(OH)4-2		3.110e-020	2.498e-020	-19.507	-19.602	-0.095
F	5.120e-006					
F-		5.045e-006	4.774e-006	-5.297	-5.321	-0.024
MgF+		6.815e-008	6.451e-008	-7.167	-7.190	-0.024
CaF+		3.290e-009	3.114e-009	-8.483	-8.507	-0.024
NaF		2.743e-009	2.744e-009	-8.562	-8.562	0.000
HF		5.224e-010	5.227e-010	-9.282	-9.282	0.000
MnF+		5.580e-012	5.283e-012	-11.253	-11.277	-0.024
HF2-		8.407e-015	7.959e-015	-14.075	-14.099	-0.024
SiF6-2		5.778e-031	4.641e-031	-30.238	-30.333	-0.095
H(0)	1.716e-025					
H2		8.581e-026	8.587e-026	-25.066	-25.066	0.000

K		1.923e-005				
	K+	1.923e-005	1.820e-005	-4.716	-4.740	-0.024
	KSO4-	3.445e-009	3.261e-009	-8.463	-8.487	-0.024
	KOH	6.301e-013	6.305e-013	-12.201	-12.200	0.000
Mg		3.642e-004				
	Mg+2	3.597e-004	2.906e-004	-3.444	-3.537	-0.093
	MgHCO3+	2.940e-006	2.783e-006	-5.532	-5.556	-0.024
	MgSO4	1.478e-006	1.479e-006	-5.830	-5.830	0.000
	MgF+	6.815e-008	6.451e-008	-7.167	-7.190	-0.024
	MgCO3	5.293e-008	5.296e-008	-7.276	-7.276	0.000
	MgOH+	1.935e-009	1.831e-009	-8.713	-8.737	-0.024
Mn(2)		2.154e-007				
	Mn+2	1.979e-007	1.600e-007	-6.704	-6.796	-0.092
	MnHCO3+	1.288e-008	1.219e-008	-7.890	-7.914	-0.024
	MnCO3	3.208e-009	3.210e-009	-8.494	-8.494	0.000
	MnSO4	7.026e-010	7.031e-010	-9.153	-9.153	0.000
	MnCl+	6.807e-010	6.444e-010	-9.167	-9.191	-0.024
	MnOH+	8.938e-012	8.462e-012	-11.049	-11.073	-0.024
	MnF+	5.580e-012	5.283e-012	-11.253	-11.277	-0.024
	MnCl2	2.780e-013	2.781e-013	-12.556	-12.556	0.000
	MnCl3-	8.002e-017	7.575e-017	-16.097	-16.121	-0.024
Mn(3)		4.772e-030				
	Mn+3	4.772e-030	2.915e-030	-29.321	-29.535	-0.214
Na		1.056e-003				
	Na+	1.055e-003	9.990e-004	-2.977	-3.000	-0.024
	NaHCO3	4.801e-007	4.804e-007	-6.319	-6.318	0.000
	NaSO4-	1.673e-007	1.584e-007	-6.776	-6.800	-0.024
	NaF	2.743e-009	2.744e-009	-8.562	-8.562	0.000
	NaCO3-	1.867e-009	1.768e-009	-8.729	-8.753	-0.024
	NaOH	6.591e-011	6.595e-011	-10.181	-10.181	0.000
O(0)		0.000e+000				
	O2	0.000e+000	0.000e+000	-48.504	-48.504	0.000
S(6)		4.676e-005				
	SO4-2	4.441e-005	3.577e-005	-4.353	-4.446	-0.094
	MgSO4	1.478e-006	1.479e-006	-5.830	-5.830	0.000
	CaSO4	7.007e-007	7.011e-007	-6.154	-6.154	0.000
	NaSO4-	1.673e-007	1.584e-007	-6.776	-6.800	-0.024
	KSO4-	3.445e-009	3.261e-009	-8.463	-8.487	-0.024
	ZnSO4	8.669e-010	8.674e-010	-9.062	-9.062	0.000
	MnSO4	7.026e-010	7.031e-010	-9.153	-9.153	0.000
	HSO4-	2.550e-010	2.414e-010	-9.593	-9.617	-0.024
	BaSO4	9.314e-011	9.320e-011	-10.031	-10.031	0.000
	CuSO4	6.369e-011	6.373e-011	-10.196	-10.196	0.000
	Zn(SO4)2-2	3.645e-013	2.928e-013	-12.438	-12.533	-0.095
	CaHSO4+	3.609e-013	3.417e-013	-12.443	-12.466	-0.024
Si		4.339e-002				
	H4SiO4	4.336e-002	4.338e-002	-1.363	-1.363	0.000
	H3SiO4-	3.303e-005	3.127e-005	-4.481	-4.505	-0.024
	H2SiO4-2	7.061e-012	5.672e-012	-11.151	-11.246	-0.095
	SiF6-2	5.778e-031	4.641e-031	-30.238	-30.333	-0.095
Zn		1.703e-007				
	Zn+2	1.490e-007	1.201e-007	-6.827	-6.921	-0.094
	ZnHCO3+	1.366e-008	1.293e-008	-7.865	-7.888	-0.024
	ZnCO3	6.048e-009	6.052e-009	-8.218	-8.218	0.000
	ZnSO4	8.669e-010	8.674e-010	-9.062	-9.062	0.000
	ZnOH+	3.194e-010	3.024e-010	-9.496	-9.519	-0.024
	Zn(OH)2	1.508e-010	1.509e-010	-9.821	-9.821	0.000
	ZnCl+	1.436e-010	1.360e-010	-9.843	-9.867	-0.024
	Zn(CO3)2-2	4.069e-011	3.268e-011	-10.391	-10.486	-0.095
	Zn(SO4)2-2	3.645e-013	2.928e-013	-12.438	-12.533	-0.095
	ZnCl2	1.301e-013	1.302e-013	-12.886	-12.885	0.000
	Zn(OH)3-	5.038e-015	4.769e-015	-14.298	-14.322	-0.024
	ZnCl3-	1.359e-016	1.286e-016	-15.867	-15.891	-0.024
	ZnCl4-2	6.806e-020	5.467e-020	-19.167	-19.262	-0.095
	Zn(OH)4-2	9.402e-021	7.552e-021	-20.027	-20.122	-0.095

------------------------------Saturation indices-------------------------------

Phase	SI	log IAP	log KT	
Anhydrite	-4.03	-8.38	-4.34	CaSO4
Aragonite	-2.28	-10.53	-8.24	CaCO3
Barite	-2.41	-12.73	-10.32	BaSO4
Calcite	-2.13	-10.53	-8.40	CaCO3
Chalcedony	2.41	-1.36	-3.78	SiO2
Chrysotile	-5.93	28.66	34.60	Mg3Si2O5(OH)4
CO2(g)	-2.35	-3.57	-1.22	CO2
Dolomite	-4.02	-20.66	-16.64	CaMg(CO3)2
Fluorite	-3.73	-14.57	-10.85	CaF2
Gypsum	-3.78	-8.38	-4.60	CaSO4:2H2O
H2(g)	-22.00	-25.07	-3.07	H2
H2O(g)	-2.01	-0.00	2.01	H2O
Halite	-7.54	-6.01	1.54	NaCl
Hausmannite	-22.22	43.61	65.83	Mn3O4
Manganite	-7.14	18.20	25.34	MnOOH
O2(g)	-45.63	-48.50	-2.87	O2
Pyrochroite	-8.00	7.20	15.20	Mn(OH)2
Pyrolusite	-15.28	29.20	44.48	MnO2
Quartz	2.90	-1.36	-4.27	SiO2
Rhodochrosite	-2.33	-13.39	-11.06	MnCO3
Sepiolite	0.57	16.84	16.27	Mg2Si3O7.5OH:3H2O
Sepiolite(d)	-1.82	16.84	18.66	Mg2Si3O7.5OH:3H2O
SiO2(a)	1.51	-1.36	-2.87	SiO2
Smithsonite	-3.73	-13.52	-9.79	ZnCO3
Talc	2.33	25.94	23.61	Mg3Si4O10(OH)2
Willemite	-4.12	12.80	16.92	Zn2SiO4
Witherite	-6.22	-14.88	-8.66	BaCO3
Zn(OH)2(e)	-4.42	7.08	11.50	Zn(OH)2

Initial solution 35.   4LCecilia-1

----------------------------Solution composition----------------------------

Elements	Molality	Moles
Alkalinity	1.122e-004	1.122e-004
Ba	2.191e-009	2.191e-009
Ca	2.202e-004	2.202e-004
Cl	3.280e-003	3.280e-003
Cu	1.736e-008	1.736e-008
F	6.492e-006	6.492e-006
K	5.924e-005	5.924e-005
Mg	3.477e-004	3.477e-004
Mn	5.841e-008	5.841e-008
Na	2.325e-003	2.325e-003
S(6)	8.894e-005	8.894e-005
Si	4.339e-002	4.339e-002
Zn	1.365e-007	1.365e-007

----------------------------Description of solution----------------------------

pH	=	7.000
pe	=	4.000
Activity of water	=	0.999
Ionic strength	=	4.186e-003
Mass of water (kg)	=	1.000e+000
Total carbon (mol/kg)	=	1.003e-004
Total CO2 (mol/kg)	=	1.003e-004
Temperature (deg C)	=	7.400
Electrical balance (eq)	=	-5.631e-005
Percent error, 100*(Cat-|An|)/(Cat+|An|)	=	-0.80
Iterations	=	9
Total H	=	1.111861e+002
Total O	=	5.568043e+001

----------------------------Distribution of species----------------------------

Species		Molality	Activity	Log Molality	Log Activity	Log Gamma
H+		1.064e-007	1.000e-007	-6.973	-7.000	-0.027
OH-		2.477e-008	2.312e-008	-7.606	-7.636	-0.030
H2O		5.551e+001	9.992e-001	1.744	-0.000	0.000
Ba	2.191e-009					
Ba+2		2.137e-009	1.636e-009	-8.670	-8.786	-0.116
BaSO4		5.264e-011	5.269e-011	-10.279	-10.278	0.000
BaHCO3+		6.987e-013	6.529e-013	-12.156	-12.185	-0.030
BaCO3		1.293e-014	1.294e-014	-13.889	-13.888	0.000
BaOH+		5.928e-016	5.539e-016	-15.227	-15.257	-0.030
C(4)	1.003e-004					
HCO3-		7.741e-005	7.245e-005	-4.111	-4.140	-0.029
CO2		2.237e-005	2.240e-005	-4.650	-4.650	0.000
MgHCO3+		2.303e-007	2.152e-007	-6.638	-6.667	-0.030
CaHCO3+		1.115e-007	1.043e-007	-6.953	-6.982	-0.029
NaHCO3		8.843e-008	8.851e-008	-7.053	-7.053	0.000
CO3-2		2.842e-008	2.180e-008	-7.546	-7.662	-0.115
CaCO3		4.918e-009	4.923e-009	-8.308	-8.308	0.000
MgCO3		4.206e-009	4.210e-009	-8.376	-8.376	0.000
ZnHCO3+		9.935e-010	9.282e-010	-9.003	-9.032	-0.030
ZnCO3		4.423e-010	4.427e-010	-9.354	-9.354	0.000
NaCO3-		3.675e-010	3.434e-010	-9.435	-9.464	-0.030
MnHCO3+		3.035e-010	2.836e-010	-9.518	-9.547	-0.030
MnCO3		7.598e-011	7.605e-011	-10.119	-10.119	0.000
BaHCO3+		6.987e-013	6.529e-013	-12.156	-12.185	-0.030
Zn(CO3)2-2		2.707e-013	2.063e-013	-12.567	-12.685	-0.118
BaCO3		1.293e-014	1.294e-014	-13.889	-13.888	0.000
Ca	2.202e-004					
Ca+2		2.183e-004	1.673e-004	-3.661	-3.776	-0.115
CaSO4		1.800e-006	1.802e-006	-5.745	-5.744	0.000
CaHCO3+		1.115e-007	1.043e-007	-6.953	-6.982	-0.029
CaF+		6.023e-009	5.628e-009	-8.220	-8.250	-0.030
CaCO3		4.918e-009	4.923e-009	-8.308	-8.308	0.000
CaOH+		2.970e-010	2.775e-010	-9.527	-9.557	-0.030
CaHSO4+		9.442e-013	8.821e-013	-12.025	-12.054	-0.030
Cl	3.280e-003					
Cl-		3.280e-003	3.062e-003	-2.484	-2.514	-0.030
MnCl+		5.864e-010	5.479e-010	-9.232	-9.261	-0.030
ZnCl+		3.936e-010	3.677e-010	-9.405	-9.434	-0.030
ZnCl2		1.093e-012	1.094e-012	-11.961	-11.961	0.000
MnCl2		7.318e-013	7.325e-013	-12.136	-12.135	0.000
ZnCl3-		3.596e-015	3.359e-015	-14.444	-14.474	-0.030
MnCl3-		6.613e-016	6.178e-016	-15.180	-15.209	-0.030
ZnCl4-2		5.834e-018	4.446e-018	-17.234	-17.352	-0.118
Cu(1)	2.312e-010					
Cu+		2.312e-010	2.155e-010	-9.636	-9.667	-0.030
Cu(2)	1.713e-008					
Cu(OH)2		1.019e-008	1.020e-008	-7.992	-7.991	0.000
Cu+2		6.357e-009	4.891e-009	-8.197	-8.311	-0.114
CuOH+		5.231e-010	4.887e-010	-9.281	-9.311	-0.030
CuSO4		5.634e-011	5.640e-011	-10.249	-10.249	0.000
Cu(OH)3-		6.573e-015	6.141e-015	-14.182	-14.212	-0.030
Cu(OH)4-2		1.607e-020	1.224e-020	-19.794	-19.912	-0.118
F	6.492e-006					
F-		6.398e-006	5.973e-006	-5.194	-5.224	-0.030
MgF+		7.983e-008	7.459e-008	-7.098	-7.127	-0.030
NaF		7.461e-009	7.468e-009	-8.127	-8.127	0.000
CaF+		6.023e-009	5.628e-009	-8.220	-8.250	-0.030
HF		6.600e-010	6.606e-010	-9.180	-9.180	0.000
MnF+		1.942e-012	1.815e-012	-11.712	-11.741	-0.030
HF2-		1.357e-014	1.268e-014	-13.867	-13.897	-0.030
SiF6-2		2.197e-030	1.674e-030	-29.658	-29.776	-0.118

H(0)	1.704e-025					
H2		8.521e-026	8.529e-026	-25.070	-25.069	0.000
K	5.924e-005					
K+		5.922e-005	5.529e-005	-4.228	-4.257	-0.030
KSO4-		1.928e-008	1.802e-008	-7.715	-7.744	-0.030
KOH		1.914e-012	1.916e-012	-11.718	-11.718	0.000
Mg	3.477e-004					
Mg+2		3.449e-004	2.652e-004	-3.462	-3.576	-0.114
MgSO4		2.466e-006	2.468e-006	-5.608	-5.608	0.000
MgHCO3+		2.303e-007	2.152e-007	-6.638	-6.667	-0.030
MgF+		7.983e-008	7.459e-008	-7.098	-7.127	-0.030
MgCO3		4.206e-009	4.210e-009	-8.376	-8.376	0.000
MgOH+		1.902e-009	1.777e-009	-8.721	-8.750	-0.030
Mn(2)	5.841e-008					
Mn+2		5.709e-008	4.392e-008	-7.243	-7.357	-0.114
MnCl+		5.864e-010	5.479e-010	-9.232	-9.261	-0.030
MnSO4		3.509e-010	3.513e-010	-9.455	-9.454	0.000
MnHCO3+		3.035e-010	2.836e-010	-9.518	-9.547	-0.030
MnCO3		7.598e-011	7.605e-011	-10.119	-10.119	0.000
MnOH+		2.628e-012	2.455e-012	-11.580	-11.610	-0.030
MnF+		1.942e-012	1.815e-012	-11.712	-11.741	-0.030
MnCl2		7.318e-013	7.325e-013	-12.136	-12.135	0.000
MnCl3-		6.613e-016	6.178e-016	-15.180	-15.209	-0.030
Mn(3)	1.629e-030					
Mn+3		1.629e-030	8.837e-031	-29.788	-30.054	-0.266
Na	2.325e-003					
Na+		2.324e-003	2.173e-003	-2.634	-2.663	-0.029
NaSO4-		6.652e-007	6.215e-007	-6.177	-6.207	-0.030
NaHCO3		8.843e-008	8.851e-008	-7.053	-7.053	0.000
NaF		7.461e-009	7.468e-009	-8.127	-8.127	0.000
NaCO3-		3.675e-010	3.434e-010	-9.435	-9.464	-0.030
NaOH		1.433e-010	1.434e-010	-9.844	-9.843	0.000
O(0)	0.000e+000					
O2		0.000e+000	0.000e+000	-48.279	-48.279	0.000
S(6)	8.894e-005					
SO4-2		8.399e-005	6.427e-005	-4.076	-4.192	-0.116
MgSO4		2.466e-006	2.468e-006	-5.608	-5.608	0.000
CaSO4		1.800e-006	1.802e-006	-5.745	-5.744	0.000
NaSO4-		6.652e-007	6.215e-007	-6.177	-6.207	-0.030
KSO4-		1.928e-008	1.802e-008	-7.715	-7.744	-0.030
ZnSO4		1.326e-009	1.328e-009	-8.877	-8.877	0.000
HSO4-		4.693e-010	4.385e-010	-9.329	-9.358	-0.030
MnSO4		3.509e-010	3.513e-010	-9.455	-9.454	0.000
CuSO4		5.634e-011	5.640e-011	-10.249	-10.249	0.000
BaSO4		5.264e-011	5.269e-011	-10.279	-10.278	0.000
Zn(SO4)2-2		1.051e-012	8.009e-013	-11.978	-12.096	-0.118
CaHSO4+		9.442e-013	8.821e-013	-12.025	-12.054	-0.030
Si	4.339e-002					
H4SiO4		4.336e-002	4.340e-002	-1.363	-1.362	0.000
H3SiO4-		3.438e-005	3.213e-005	-4.464	-4.493	-0.030
H2SiO4-2		8.023e-012	6.114e-012	-11.096	-11.214	-0.118
SiF6-2		2.197e-030	1.674e-030	-29.658	-29.776	-0.118
Zn	1.365e-007					
Zn+2		1.330e-007	1.018e-007	-6.876	-6.992	-0.116
ZnSO4		1.326e-009	1.328e-009	-8.877	-8.877	0.000
ZnHCO3+		9.935e-010	9.282e-010	-9.003	-9.032	-0.030
ZnCO3		4.423e-010	4.427e-010	-9.354	-9.354	0.000
ZnCl+		3.936e-010	3.677e-010	-9.405	-9.434	-0.030
ZnOH+		2.888e-010	2.698e-010	-9.539	-9.569	-0.030
Zn(OH)2		1.278e-010	1.279e-010	-9.894	-9.893	0.000
ZnCl2		1.093e-012	1.094e-012	-11.961	-11.961	0.000
Zn(SO4)2-2		1.051e-012	8.009e-013	-11.978	-12.096	-0.118
Zn(CO3)2-2		2.707e-013	2.063e-013	-12.567	-12.685	-0.118
Zn(OH)3-		4.325e-015	4.041e-015	-14.364	-14.393	-0.030
ZnCl3-		3.596e-015	3.359e-015	-14.444	-14.474	-0.030
ZnCl4-2		5.834e-018	4.446e-018	-17.234	-17.352	-0.118
Zn(OH)4-2		8.398e-021	6.400e-021	-20.076	-20.194	-0.118

----------------------------Saturation indices-----------------------------

Phase	SI	log IAP	log KT	
Anhydrite	-3.63	-7.97	-4.34	CaSO4
Aragonite	-3.19	-11.44	-8.24	CaCO3
Barite	-2.68	-12.98	-10.30	BaSO4
Calcite	-3.04	-11.44	-8.40	CaCO3
Chalcedony	2.41	-1.36	-3.77	SiO2
Chrysotile	-5.97	28.55	34.51	Mg3Si2O5(OH)4
CO2(g)	-3.42	-4.65	-1.23	CO2
Dolomite	-6.02	-22.68	-16.66	CaMg(CO3)2
Fluorite	-3.39	-14.22	-10.84	CaF2
Gypsum	-3.37	-7.97	-4.60	CaSO4:2H2O
H2(g)	-22.00	-25.07	-3.07	H2
H2O(g)	-1.99	-0.00	1.99	H2O
Halite	-6.72	-5.18	1.54	NaCl
Hausmannite	-23.73	41.93	65.66	Mn3O4
Manganite	-7.70	17.64	25.34	MnOOH
O2(g)	-45.40	-48.28	-2.88	O2
Pyrochroite	-8.56	6.64	15.20	Mn(OH)2
Pyrolusite	-15.73	28.64	44.37	MnO2
Quartz	2.89	-1.36	-4.26	SiO2
Rhodochrosite	-3.95	-15.02	-11.06	MnCO3
Sepiolite	0.51	16.76	16.25	Mg2Si3O7.5OH:3H2O
Sepiolite(d)	-1.90	16.76	18.66	Mg2Si3O7.5OH:3H2O
SiO2(a)	1.50	-1.36	-2.87	SiO2
Smithsonite	-4.85	-14.65	-9.80	ZnCO3
Talc	2.29	25.82	23.53	Mg3Si4O10(OH)2
Willemite	-4.21	12.65	16.86	Zn2SiO4
Witherite	-7.79	-16.45	-8.65	BaCO3
Zn(OH)2(e)	-4.49	7.01	11.50	Zn(OH)2

Initial solution 36.   4HFlorencia-1

----------------------------Solution composition----------------------------

Elements	Molality	Moles
Alkalinity	9.012e-004	9.012e-004
Ba	1.459e-009	1.459e-009
Ca	6.748e-005	6.748e-005
Cl	7.651e-005	7.651e-005
Cu	1.734e-008	1.734e-008
F	4.271e-006	4.271e-006
K	1.537e-005	1.537e-005
Mg	6.798e-005	6.798e-005
Mn	4.376e-008	4.376e-008
Na	7.189e-004	7.189e-004
S(6)	5.839e-006	5.839e-006
Si	2.667e-002	2.667e-002
Zn	8.428e-008	8.428e-008

----------------------------Description of solution----------------------------

pH = 7.000
pe = 4.000
Activity of water = 1.000
Ionic strength = 1.139e-003
Mass of water (kg) = 1.000e+000
Total carbon (mol/kg) = 1.162e-003
Total CO2 (mol/kg) = 1.162e-003
Temperature (deg C) = 4.900
Electrical balance (eq) = 1.181e-005
Percent error, 100*(Cat-|An|)/(Cat+|An|) = 0.59
Iterations = 9

Total H = 1.111200e+002
Total O = 5.561614e+001

----------------------------Distribution of species----------------------------

Species		Molality	Activity	Log Molality	Log Activity	Log Gamma
H+		1.036e-007	1.000e-007	-6.985	-7.000	-0.015
OH-		1.903e-008	1.834e-008	-7.720	-7.737	-0.016
H2O		5.551e+001	9.995e-001	1.744	-0.000	0.000
Ba	1.459e-009					
Ba+2		1.450e-009	1.253e-009	-8.839	-8.902	-0.063
BaHCO3+		5.626e-012	5.423e-012	-11.250	-11.266	-0.016
BaSO4		3.115e-012	3.116e-012	-11.507	-11.506	0.000
BaCO3		1.022e-013	1.022e-013	-12.991	-12.991	0.000
BaOH+		4.404e-016	4.245e-016	-15.356	-15.372	-0.016
C(4)	1.162e-003					
HCO3-		8.811e-004	8.497e-004	-3.055	-3.071	-0.016
CO2		2.798e-004	2.799e-004	-3.553	-3.553	0.000
MgHCO3+		5.756e-007	5.548e-007	-6.240	-6.256	-0.016
CaHCO3+		4.050e-007	3.906e-007	-6.393	-6.408	-0.016
NaHCO3		3.309e-007	3.310e-007	-6.480	-6.480	0.000
CO3-2		2.732e-007	2.363e-007	-6.564	-6.627	-0.063
CaCO3		1.840e-008	1.840e-008	-7.735	-7.735	0.000
MgCO3		9.651e-009	9.653e-009	-8.015	-8.015	0.000
ZnHCO3+		7.087e-009	6.832e-009	-8.150	-8.165	-0.016
ZnCO3		3.010e-009	3.011e-009	-8.521	-8.521	0.000
MnHCO3+		2.743e-009	2.644e-009	-8.562	-8.578	-0.016
NaCO3-		1.066e-009	1.028e-009	-8.972	-8.988	-0.016
MnCO3		6.552e-010	6.553e-010	-9.184	-9.184	0.000
Zn(CO3)2-2		1.762e-011	1.521e-011	-10.754	-10.818	-0.064
BaHCO3+		5.626e-012	5.423e-012	-11.250	-11.266	-0.016
BaCO3		1.022e-013	1.022e-013	-12.991	-12.991	0.000
Ca	6.748e-005					
Ca+2		6.701e-005	5.795e-005	-4.174	-4.237	-0.063
CaHCO3+		4.050e-007	3.906e-007	-6.393	-6.408	-0.016
CaSO4		4.688e-008	4.689e-008	-7.329	-7.329	0.000
CaCO3		1.840e-008	1.840e-008	-7.735	-7.735	0.000
CaF+		1.299e-009	1.252e-009	-8.886	-8.902	-0.016
CaOH+		9.973e-011	9.613e-011	-10.001	-10.017	-0.016
CaHSO4+		2.339e-014	2.254e-014	-13.631	-13.647	-0.016
Cl	7.651e-005					
Cl-		7.651e-005	7.374e-005	-4.116	-4.132	-0.016
MnCl+		1.088e-011	1.049e-011	-10.963	-10.979	-0.016
ZnCl+		5.083e-012	4.900e-012	-11.294	-11.310	-0.016
ZnCl2		3.468e-016	3.469e-016	-15.460	-15.460	0.000
MnCl2		3.375e-016	3.376e-016	-15.472	-15.472	0.000
ZnCl3-		2.617e-020	2.522e-020	-19.582	-19.598	-0.016
MnCl3-		7.113e-021	6.856e-021	-20.148	-20.164	-0.016
ZnCl4-2		9.102e-025	7.857e-025	-24.041	-24.105	-0.064
Cu(1)	2.278e-010					
Cu+		2.278e-010	2.194e-010	-9.642	-9.659	-0.016
Cu(2)	1.711e-008					
Cu(OH)2		1.067e-008	1.067e-008	-7.972	-7.972	0.000
Cu+2		5.906e-009	5.114e-009	-8.229	-8.291	-0.063
CuOH+		5.303e-010	5.111e-010	-9.276	-9.291	-0.016
CuSO4		4.461e-012	4.462e-012	-11.351	-11.350	0.000
Cu(OH)3-		6.669e-015	6.428e-015	-14.176	-14.192	-0.016
Cu(OH)4-2		1.485e-020	1.282e-020	-19.828	-19.892	-0.064
F	4.271e-006					
F-		4.256e-006	4.102e-006	-5.371	-5.387	-0.016
MgF+		1.109e-008	1.069e-008	-7.955	-7.971	-0.016
NaF		1.635e-009	1.635e-009	-8.787	-8.786	0.000
CaF+		1.299e-009	1.252e-009	-8.886	-8.902	-0.016
HF		4.352e-010	4.353e-010	-9.361	-9.361	0.000
MnF+		1.028e-012	9.908e-013	-11.988	-12.004	-0.016

HF2-	5.765e-015	5.557e-015	-14.239	-14.255	-0.016
SiF6-2	1.622e-031	1.400e-031	-30.790	-30.854	-0.064
H(0)	1.754e-025				
H2	8.772e-026	8.774e-026	-25.057	-25.057	0.000
K	1.537e-005				
K+	1.537e-005	1.481e-005	-4.813	-4.829	-0.016
KSO4-	3.677e-010	3.544e-010	-9.435	-9.451	-0.016
KOH	5.132e-013	5.134e-013	-12.290	-12.290	0.000
Mg	6.798e-005				
Mg+2	6.735e-005	5.830e-005	-4.172	-4.234	-0.063
MgHCO3+	5.756e-007	5.548e-007	-6.240	-6.256	-0.016
MgSO4	3.889e-008	3.891e-008	-7.410	-7.410	0.000
MgF+	1.109e-008	1.069e-008	-7.955	-7.971	-0.016
MgCO3	9.651e-009	9.653e-009	-8.015	-8.015	0.000
MgOH+	3.134e-010	3.021e-010	-9.504	-9.520	-0.016
Mn(2)	4.376e-008				
Mn+2	4.033e-008	3.492e-008	-7.394	-7.457	-0.063
MnHCO3+	2.743e-009	2.644e-009	-8.562	-8.578	-0.016
MnCO3	6.552e-010	6.553e-010	-9.184	-9.184	0.000
MnSO4	2.041e-011	2.041e-011	-10.690	-10.690	0.000
MnCl+	1.088e-011	1.049e-011	-10.963	-10.979	-0.016
MnOH+	1.606e-012	1.548e-012	-11.794	-11.810	-0.016
MnF+	1.028e-012	9.908e-013	-11.988	-12.004	-0.016
MnCl2	3.375e-016	3.376e-016	-15.472	-15.472	0.000
MnCl3-	7.113e-021	6.856e-021	-20.148	-20.164	-0.016
Mn(3)	6.451e-031				
Mn+3	6.451e-031	4.634e-031	-30.190	-30.334	-0.144
Na	7.189e-004				
Na+	7.186e-004	6.928e-004	-3.144	-3.159	-0.016
NaHCO3	3.309e-007	3.310e-007	-6.480	-6.480	0.000
NaSO4-	1.558e-008	1.502e-008	-7.807	-7.823	-0.016
NaF	1.635e-009	1.635e-009	-8.787	-8.786	0.000
NaCO3-	1.066e-009	1.028e-009	-8.972	-8.988	-0.016
NaOH	4.574e-011	4.575e-011	-10.340	-10.340	0.000
O(0)	0.000e+000				
O2	0.000e+000	0.000e+000	-49.223	-49.223	0.000
S(6)	5.839e-006				
SO4-2	5.737e-006	4.960e-006	-5.241	-5.305	-0.063
CaSO4	4.688e-008	4.689e-008	-7.329	-7.329	0.000
MgSO4	3.889e-008	3.891e-008	-7.410	-7.410	0.000
NaSO4-	1.558e-008	1.502e-008	-7.807	-7.823	-0.016
KSO4-	3.677e-010	3.544e-010	-9.435	-9.451	-0.016
ZnSO4	6.288e-011	6.290e-011	-10.201	-10.201	0.000
HSO4-	3.356e-011	3.235e-011	-10.474	-10.490	-0.016
MnSO4	2.041e-011	2.041e-011	-10.690	-10.690	0.000
CuSO4	4.461e-012	4.462e-012	-11.351	-11.350	0.000
BaSO4	3.115e-012	3.116e-012	-11.507	-11.506	0.000
CaHSO4+	2.339e-014	2.254e-014	-13.631	-13.647	-0.016
Zn(SO4)2-2	3.467e-015	2.993e-015	-14.460	-14.524	-0.064
Si	2.667e-002				
H4SiO4	2.666e-002	2.666e-002	-1.574	-1.574	0.000
H3SiO4-	1.831e-005	1.765e-005	-4.737	-4.753	-0.016
H2SiO4-2	3.175e-012	2.741e-012	-11.498	-11.562	-0.064
SiF6-2	1.622e-031	1.400e-031	-30.790	-30.854	-0.064
Zn	8.428e-008				
Zn+2	7.387e-008	6.387e-008	-7.132	-7.195	-0.063
ZnHCO3+	7.087e-009	6.832e-009	-8.150	-8.165	-0.016
ZnCO3	3.010e-009	3.011e-009	-8.521	-8.521	0.000
ZnOH+	1.416e-010	1.365e-010	-9.849	-9.865	-0.016
Zn(OH)2	8.030e-011	8.032e-011	-10.095	-10.095	0.000
ZnSO4	6.288e-011	6.290e-011	-10.201	-10.201	0.000
Zn(CO3)2-2	1.762e-011	1.521e-011	-10.754	-10.818	-0.064
ZnCl+	5.083e-012	4.900e-012	-11.294	-11.310	-0.016
Zn(SO4)2-2	3.467e-015	2.993e-015	-14.460	-14.524	-0.064
Zn(OH)3-	2.634e-015	2.539e-015	-14.579	-14.595	-0.016
ZnCl2	3.468e-016	3.469e-016	-15.460	-15.460	0.000
ZnCl3-	2.617e-020	2.522e-020	-19.582	-19.598	-0.016

Zn(OH)4-2	4.659e-021	4.022e-021	-20.332	-20.396	-0.064
ZnCl4-2	9.102e-025	7.857e-025	-24.041	-24.105	-0.064

------------------------------Saturation indices-------------------------------

Phase	SI	log IAP	log KT	
Anhydrite	-5.20	-9.54	-4.35	CaSO4
Aragonite	-2.63	-10.86	-8.23	CaCO3
Barite	-3.85	-14.21	-10.36	BaSO4
Calcite	-2.47	-10.86	-8.39	CaCO3
Chalcedony	2.23	-1.57	-3.80	SiO2
Chrysotile	-8.72	26.15	34.87	Mg3Si2O5(OH)4
CO2(g)	-2.36	-3.55	-1.19	CO2
Dolomite	-5.13	-21.72	-16.59	CaMg(CO3)2
Fluorite	-4.14	-15.01	-10.88	CaF2
Gypsum	-4.94	-9.54	-4.60	CaSO4:2H2O
H2(g)	-22.00	-25.06	-3.06	H2
H2O(g)	-2.07	-0.00	2.07	H2O
Halite	-8.83	-7.29	1.53	NaCl
Hausmannite	-24.73	41.63	66.36	Mn3O4
Manganite	-7.80	17.54	25.34	MnOOH
O2(g)	-46.36	-49.22	-2.86	O2
Pyrochroite	-8.66	6.54	15.20	Mn(OH)2
Pyrolusite	-16.29	28.54	44.83	MnO2
Quartz	2.72	-1.57	-4.30	SiO2
Rhodochrosite	-3.03	-14.08	-11.05	MnCO3
Sepiolite	-1.52	14.81	16.33	Mg2Si3O7.5OH:3H2O
Sepiolite(d)	-3.85	14.81	18.66	Mg2Si3O7.5OH:3H2O
SiO2(a)	1.32	-1.57	-2.89	SiO2
Smithsonite	-4.05	-13.82	-9.77	ZnCO3
Talc	-0.85	23.00	23.86	Mg3Si4O10(OH)2
Willemite	-5.06	12.04	17.10	Zn2SiO4
Witherite	-6.85	-15.53	-8.68	BaCO3
Zn(OH)2(e)	-4.70	6.80	11.50	Zn(OH)2

Initial solution 37.   4LFlorencia-1

-----------------------------Solution composition------------------------------

Elements	Molality	Moles
Alkalinity	5.607e-005	5.607e-005
Ba	6.556e-009	6.556e-009
Ca	3.245e-004	3.245e-004
Cl	6.263e-004	6.263e-004
Cu	3.149e-008	3.149e-008
F	7.214e-006	7.214e-006
K	7.676e-006	7.676e-006
Mg	9.465e-006	9.465e-006
Mn	1.657e-007	1.657e-007
Na	6.528e-005	6.528e-005
S(6)	2.541e-005	2.541e-005
Si	6.660e-003	6.660e-003
Zn	1.852e-007	1.852e-007

----------------------------Description of solution----------------------------

pH	=	7.000
pe	=	4.000
Activity of water	=	1.000
Ionic strength	=	1.097e-003
Mass of water (kg)	=	1.000e+000
Total carbon (mol/kg)	=	7.001e-005
Total CO2 (mol/kg)	=	7.001e-005
Temperature (deg C)	=	2.000
Electrical balance (eq)	=	1.193e-006

Percent error, 100*(Cat-|An|)/(Cat+|An|) = 0.08
Iterations = 7
Total H = 1.110391e+002
Total O = 5.553315e+001

----------------------------Distribution of species----------------------------

Species		Molality	Activity	Log Molality	Log Activity	Log Gamma
H+		1.035e-007	1.000e-007	-6.985	-7.000	-0.015
OH-		1.442e-008	1.391e-008	-7.841	-7.857	-0.016
H2O		5.551e+001	9.999e-001	1.744	-0.000	0.000
Ba	6.556e-009					
Ba+2		6.495e-009	5.632e-009	-8.187	-8.249	-0.062
BaSO4		5.981e-011	5.983e-011	-10.223	-10.223	0.000
BaHCO3+		1.361e-012	1.313e-012	-11.866	-11.882	-0.016
BaCO3		2.321e-014	2.321e-014	-13.634	-13.634	0.000
BaOH+		1.978e-015	1.908e-015	-14.704	-14.719	-0.016
C(4)	7.001e-005					
HCO3-		5.198e-005	5.017e-005	-4.284	-4.300	-0.015
CO2		1.790e-005	1.790e-005	-4.747	-4.747	0.000
CaHCO3+		1.042e-007	1.006e-007	-6.982	-6.997	-0.015
CO3-2		1.459e-008	1.266e-008	-7.836	-7.898	-0.062
CaCO3		4.799e-009	4.800e-009	-8.319	-8.319	0.000
MgHCO3+		4.782e-009	4.613e-009	-8.320	-8.336	-0.016
NaHCO3		1.776e-009	1.776e-009	-8.751	-8.750	0.000
ZnHCO3+		1.036e-009	9.997e-010	-8.985	-9.000	-0.016
MnHCO3+		6.609e-010	6.376e-010	-9.180	-9.195	-0.016
ZnCO3		3.998e-010	3.999e-010	-9.398	-9.398	0.000
MnCO3		1.434e-010	1.434e-010	-9.844	-9.843	0.000
MgCO3		6.950e-011	6.952e-011	-10.158	-10.158	0.000
NaCO3-		4.378e-012	4.223e-012	-11.359	-11.374	-0.016
BaHCO3+		1.361e-012	1.313e-012	-11.866	-11.882	-0.016
Zn(CO3)2-2		1.250e-013	1.082e-013	-12.903	-12.966	-0.062
BaCO3		2.321e-014	2.321e-014	-13.634	-13.634	0.000
Ca	3.245e-004					
Ca+2		3.234e-004	2.806e-004	-3.490	-3.552	-0.062
CaSO4		9.399e-007	9.401e-007	-6.027	-6.027	0.000
CaHCO3+		1.042e-007	1.006e-007	-6.982	-6.997	-0.015
CaF+		9.839e-009	9.491e-009	-8.007	-8.023	-0.016
CaCO3		4.799e-009	4.800e-009	-8.319	-8.319	0.000
CaOH+		4.827e-010	4.656e-010	-9.316	-9.332	-0.016
CaHSO4+		4.598e-013	4.435e-013	-12.337	-12.353	-0.016
Cl	6.263e-004					
Cl-		6.263e-004	6.041e-004	-3.203	-3.219	-0.016
MnCl+		3.638e-010	3.509e-010	-9.439	-9.455	-0.016
ZnCl+		8.890e-011	8.576e-011	-10.051	-10.067	-0.016
MnCl2		9.252e-014	9.254e-014	-13.034	-13.034	0.000
ZnCl2		4.907e-014	4.908e-014	-13.309	-13.309	0.000
ZnCl3-		2.970e-017	2.865e-017	-16.527	-16.543	-0.016
MnCl3-		1.596e-017	1.540e-017	-16.797	-16.813	-0.016
ZnCl4-2		8.220e-021	7.119e-021	-20.085	-20.148	-0.062
Cu(1)	4.006e-010					
Cu+		4.006e-010	3.862e-010	-9.397	-9.413	-0.016
Cu(2)	3.109e-008					
Cu(OH)2		1.940e-008	1.940e-008	-7.712	-7.712	0.000
Cu+2		1.069e-008	9.288e-009	-7.971	-8.032	-0.061
CuOH+		9.627e-010	9.287e-010	-9.017	-9.032	-0.016
CuSO4		3.383e-011	3.384e-011	-10.471	-10.471	0.000
Cu(OH)3-		1.212e-014	1.169e-014	-13.917	-13.932	-0.016
Cu(OH)4-2		2.693e-020	2.332e-020	-19.570	-19.632	-0.062
F	7.214e-006					
F-		7.201e-006	6.945e-006	-5.143	-5.158	-0.016
CaF+		9.839e-009	9.491e-009	-8.007	-8.023	-0.016
MgF+		2.481e-009	2.394e-009	-8.605	-8.621	-0.016
HF		7.031e-010	7.033e-010	-9.153	-9.153	0.000

NaF		2.516e-010	2.517e-010	-9.599	-9.599	0.000
MnF+		7.103e-012	6.852e-012	-11.149	-11.164	-0.016
HF2-		1.514e-014	1.461e-014	-13.820	-13.835	-0.016
SiF6-2		1.295e-030	1.122e-030	-29.888	-29.950	-0.062
H(0)	1.814e-025					
H2		9.071e-026	9.074e-026	-25.042	-25.042	0.000
K	7.676e-006					
K+		7.675e-006	7.402e-006	-5.115	-5.131	-0.016
KSO4-		7.397e-010	7.136e-010	-9.131	-9.147	-0.016
KOH		2.566e-013	2.566e-013	-12.591	-12.591	0.000
Mg	9.465e-006					
Mg+2		9.436e-006	8.193e-006	-5.025	-5.087	-0.061
MgSO4		2.142e-008	2.142e-008	-7.669	-7.669	0.000
MgHCO3+		4.782e-009	4.613e-009	-8.320	-8.336	-0.016
MgF+		2.481e-009	2.394e-009	-8.605	-8.621	-0.016
MgCO3		6.950e-011	6.952e-011	-10.158	-10.158	0.000
MgOH+		3.248e-011	3.133e-011	-10.488	-10.504	-0.016
Mn(2)	1.657e-007					
Mn+2		1.642e-007	1.426e-007	-6.785	-6.846	-0.061
MnHCO3+		6.609e-010	6.376e-010	-9.180	-9.195	-0.016
MnCl+		3.638e-010	3.509e-010	-9.439	-9.455	-0.016
MnSO4		3.340e-010	3.341e-010	-9.476	-9.476	0.000
MnCO3		1.434e-010	1.434e-010	-9.844	-9.843	0.000
MnF+		7.103e-012	6.852e-012	-11.149	-11.164	-0.016
MnOH+		4.982e-012	4.806e-012	-11.303	-11.318	-0.016
MnCl2		9.252e-014	9.254e-014	-13.034	-13.034	0.000
MnCl3-		1.596e-017	1.540e-017	-16.797	-16.813	-0.016
Mn(3)	1.599e-030					
Mn+3		1.599e-030	1.157e-030	-29.796	-29.937	-0.141
Na	6.528e-005					
Na+		6.527e-005	6.297e-005	-4.185	-4.201	-0.016
NaSO4-		5.920e-009	5.711e-009	-8.228	-8.243	-0.016
NaHCO3		1.776e-009	1.776e-009	-8.751	-8.750	0.000
NaF		2.516e-010	2.517e-010	-9.599	-9.599	0.000
NaCO3-		4.378e-012	4.223e-012	-11.359	-11.374	-0.016
NaOH		4.159e-012	4.160e-012	-11.381	-11.381	0.000
O(0)	0.000e+000					
O2		0.000e+000	0.000e+000	-50.339	-50.339	0.000
S(6)	2.541e-005					
SO4-2		2.444e-005	2.119e-005	-4.612	-4.674	-0.062
CaSO4		9.399e-007	9.401e-007	-6.027	-6.027	0.000
MgSO4		2.142e-008	2.142e-008	-7.669	-7.669	0.000
NaSO4-		5.920e-009	5.711e-009	-8.228	-8.243	-0.016
KSO4-		7.397e-010	7.136e-010	-9.131	-9.147	-0.016
ZnSO4		6.490e-010	6.491e-010	-9.188	-9.188	0.000
MnSO4		3.340e-010	3.341e-010	-9.476	-9.476	0.000
HSO4-		1.363e-010	1.315e-010	-9.866	-9.881	-0.016
BaSO4		5.981e-011	5.983e-011	-10.223	-10.223	0.000
CuSO4		3.383e-011	3.384e-011	-10.471	-10.471	0.000
CaHSO4+		4.598e-013	4.435e-013	-12.337	-12.353	-0.016
Zn(SO4)2-2		1.564e-013	1.355e-013	-12.806	-12.868	-0.062
Si	6.660e-003					
H4SiO4		6.656e-003	6.658e-003	-2.177	-2.177	0.000
H3SiO4-		3.992e-006	3.851e-006	-5.399	-5.414	-0.016
H2SiO4-2		5.417e-013	4.691e-013	-12.266	-12.329	-0.062
SiF6-2		1.295e-030	1.122e-030	-29.888	-29.950	-0.062
Zn	1.852e-007					
Zn+2		1.825e-007	1.583e-007	-6.739	-6.801	-0.062
ZnHCO3+		1.036e-009	9.997e-010	-8.985	-9.000	-0.016
ZnSO4		6.490e-010	6.491e-010	-9.188	-9.188	0.000
ZnCO3		3.998e-010	3.999e-010	-9.398	-9.398	0.000
ZnOH+		2.716e-010	2.620e-010	-9.566	-9.582	-0.016
Zn(OH)2		1.992e-010	1.992e-010	-9.701	-9.701	0.000
ZnCl+		8.890e-011	8.576e-011	-10.051	-10.067	-0.016
Zn(SO4)2-2		1.564e-013	1.355e-013	-12.806	-12.868	-0.062
Zn(CO3)2-2		1.250e-013	1.082e-013	-12.903	-12.966	-0.062
ZnCl2		4.907e-014	4.908e-014	-13.309	-13.309	0.000

```
   Zn(OH)3-             6.530e-015  6.299e-015   -14.185   -14.201    -0.016
   ZnCl3-               2.970e-017  2.865e-017   -16.527   -16.543    -0.016
   Zn(OH)4-2            1.153e-020  9.982e-021   -19.938   -20.001    -0.062
   ZnCl4-2              8.220e-021  7.119e-021   -20.085   -20.148    -0.062

------------------------------Saturation indices-------------------------------

      Phase                SI  log IAP  log KT

      Anhydrite         -3.87   -8.23   -4.36  CaSO4
      Aragonite         -3.23  -11.45   -8.22  CaCO3
      Barite            -2.49  -12.92  -10.43  BaSO4
      Calcite           -3.06  -11.45   -8.39  CaCO3
      Chalcedony         1.66   -2.18   -3.84  SiO2
      Chrysotile       -12.89   22.39   35.28  Mg3Si2O5(OH)4
      CO2(g)            -3.60   -4.75   -1.14  CO2
      Dolomite          -7.92  -24.43  -16.51  CaMg(CO3)2
      Fluorite          -2.94  -13.87  -10.92  CaF2
      Gypsum            -3.62   -8.23   -4.61  CaSO4:2H2O
      H2(g)            -22.00  -25.04   -3.04  H2
      H2O(g)            -2.15   -0.00    2.15  H2O
      Halite            -8.95   -7.42    1.53  NaCl
      Hausmannite      -23.73   43.46   67.20  Mn3O4
      Manganite         -7.19   18.15   25.34  MnOOH
      O2(g)            -47.49  -50.34   -2.85  O2
      Pyrochroite       -8.05    7.15   15.20  Mn(OH)2
      Pyrolusite       -16.22   29.15   45.37  MnO2
      Quartz             2.17   -2.18   -4.35  SiO2
      Rhodochrosite     -3.70  -14.74  -11.04  MnCO3
      Sepiolite         -5.12   11.30   16.42  Mg2Si3O7.5OH:3H2O
      Sepiolite(d)      -7.36   11.30   18.66  Mg2Si3O7.5OH:3H2O
      SiO2(a)            0.74   -2.18   -2.92  SiO2
      Smithsonite       -4.97  -14.70   -9.73  ZnCO3
      Talc              -6.21   18.03   24.24  Mg3Si4O10(OH)2
      Willemite         -5.15   12.22   17.37  Zn2SiO4
      Witherite         -7.43  -16.15   -8.71  BaCO3
      Zn(OH)2(e)        -4.30    7.20   11.50  Zn(OH)2

------------------
End of simulation.
------------------

------------------------------------
Reading input data for simulation 2.
------------------------------------

-----------
End of run.
-----------
```
